# The drug:H^+^ antiporters of family 2 (DHA2), siderophore transporters (ARN) and glutathione:H^+^ antiporters (GEX) have a common evolutionary origin in hemiascomycete yeasts

**DOI:** 10.1186/1471-2164-14-901

**Published:** 2013-12-18

**Authors:** Paulo Jorge Dias, Isabel Sá-Correia

**Affiliations:** 1 IBB – Institute for Biotechnology and Bioengineering, Centre for Biological and Chemical Engineering, Instituto Superior Técnico, Universidade de Lisboa, Av. Rovisco Pais, 1049-001, Lisboa, Portugal; 2 Department of Bioengineering, Instituto Superior Técnico, Universidade de Lisboa, Av. Rovisco Pais, 1049-001, Lisboa, Portugal

**Keywords:** Multidrug resistance (MDR), Hemiascomycete yeasts, Major facilitator superfamily (MFS), 14-spanner MFS transporters, DHA2 transporters, ARN transporters, GEX transporters, Comparative genomics, Phylogenetic analysis, Gene neighbourhood analysis

## Abstract

**Background:**

The *Saccharomyces cerevisiae* 14-spanner Drug:H^+^ Antiporter family 2 (DHA2) are transporters of the Major Facilitator Superfamily (MFS) involved in multidrug resistance (MDR). Although poorly characterized, DHA2 family members were found to participate in the export of structurally and functionally unrelated compounds or in the uptake of amino acids into the vacuole or the cell. In *S. cerevisiae*, the four ARN/SIT family members encode siderophore transporters and the two GEX family members encode glutathione extrusion pumps. The evolutionary history of DHA2, ARN and GEX genes, encoding 14-spanner MFS transporters, is reconstructed in this study.

**Results:**

The translated ORFs of 31 strains from 25 hemiascomycetous species, including 10 pathogenic *Candida* species, were compared using a local sequence similarity algorithm. The constraining and traversing of a network representing the pairwise similarity data gathered 355 full size proteins and retrieved ARN and GEX family members together with DHA2 transporters, suggesting the existence of a close phylogenetic relationship among these 14-spanner major facilitators. Gene neighbourhood analysis was combined with tree construction methodologies to reconstruct their evolutionary history and 7 DHA2 gene lineages, 5 ARN gene lineages, and 1 GEX gene lineage, were identified. The *S. cerevisiae* DHA2 proteins Sge1, Azr1, Vba3 and Vba5 co-clustered in a large phylogenetic branch, the *ATR1* and YMR279C genes were proposed to be paralogs formed during the Whole Genome Duplication (WGD) whereas the closely related ORF YOR378W resides in its own lineage. Homologs of *S. cerevisiae* DHA2 vacuolar proteins Vba1, Vba2 and Vba4 occur widespread in the Hemiascomycetes. Arn1/Arn2 homologs were only found in species belonging to the *Saccharomyces* complex and are more abundant in the pre-WGD species. Arn4 homologs were only found in sub-telomeric regions of species belonging to the *Sacharomyces sensu strictu* group (SSSG). Arn3 type siderophore transporters are abundant in the Hemiascomycetes and form an ancient gene lineage extending to the filamentous fungi.

**Conclusions:**

The evolutionary history of DHA2, ARN and GEX genes was reconstructed and a common evolutionary root shared by the encoded proteins is hypothesized. A new protein family, denominated DAG, is proposed to span these three phylogenetic subfamilies of 14-spanner MFS transporters.

## Background

The *Saccharomyces cerevisiae* major facilitator superfamily (MFS) of transporters involved in multidrug resistance (MDR), i.e., in the simultaneous resistance to a wide range of structurally and functionally unrelated cytotoxic chemicals [1], were classified in three protein families as deduced from the sequence analysis of the open reading frames identified after the release of the whole genome sequence [2-6]. Two of these families are the 12-spanner drug:H^+^ antiporter family 1 (DHA1) and the 14-spanner drug:H^+^ antiporter family 2 (DHA2) [7,8] while the third was called the Unknown Major Facilitator (UMF) family [6]. Following the demonstration that four *S. cerevisiae* UMF members encoded siderophore transporters [9-12], these proteins were reassigned to a new protein family, denominated the ARN family (also known as the SIT family) [13,14]. Arn2p and Arn4p (also known as Enb1p) have high siderophore substrate specificity for the bacterial catecholate enterobactin and for triacetylfusarinine C, respectively, while Arn1 and Arn3p (also known as Sit1p) show a broad and overlapping siderophore substrate specificity [14]. Although the other two proteins of the UMF family are highly similar to the ARN transporters [15,16], no experimental evidence has been obtained supporting their involvement in siderophore transport [17,18]. More recently, these two proteins were demonstrated to function as glutathione extrusion pumps (GEX) and designated Gex1p and Gex2p [18].

The *S. cerevisiae* DHA2 family comprises ten proteins encoded by *ATR1*, YMR279C, YOR378W, *SGE1*, *AZR1*, *VBA1*, *VBA2*, *VBA3*, *VBA4* and *VBA5* genes [19], but the physiological functions of the majority of these proteins are still unclear. *ATR1* was the first *S. cerevisiae* DHA2 gene to be biochemically characterized and found to confer resistance to aminotriazole and 4-nitroquinoline-1-oxide (4-NQO) [20-22]. Later, a screen for yeast genes conferring resistance to boron revealed that the plasma membrane Atr1p was the main exporter for this element [23]. The ORF YMR279C was proposed to encode a back-up boron pump [24] while ORF YOR378W is not required for boron tolerance [23,24] but determines yeast resistance to cycloheximide and streptomycin and sensitivity to rapamycin [25,26]. Vba1p, Vba2p and Vba3p were found to be involved in vacuolar uptake of basic amino acids, mediating the transport of histidine and lysine into the vacuole [27] and Vba2p was also found to catalyze the vacuolar transport of arginine [27]. Although Vba4p was localized at the vacuolar membrane [28], no significant differences in the vacuolar uptake of basic amino acids were registered in a *∆vba4* mutant compared with the parental strain [27] and the physiological function of Vba4p remains unknown [19]. With the exception of five amino acid residues and the presence of an extra peptide of 124 amino acids in the N terminus, Vba5p exhibits the same sequence as Vba3p [29]. However, differently from Vba3p, Vba5p localizes exclusively at the plasma membrane where it catalyzes the uptake of lysine and arginine into the cell [29] and *VBA5* overexpression was shown to lead to increased susceptibility of yeast cells to 4-NQO and quinidine [29]. The transcription level of *S. cerevisiae VBA3* gene was found to be highly induced under low-iron conditions [30,31]. The *AZR1* gene encodes a plasma membrane transporter required for yeast adaptation to low-molecular-weight organic acids, in particular to acetic acid, and to the antifungals ketoconazole and fluconazole and to polymyxin B [32,33]. The *SGE1* gene encodes a plasma membrane transporter presumably involved in the expulsion of dye molecules possessing a large unsaturated domain that stabilizes a permanent positive charge, such as 10-N-nonyl acridine orange, crystal violet, ethidium bromide and malachite green [34-36]. *SGE1* gene also confers resistance to methylmethane sulfonate [36] and was reported to be present in multiple copies in the genomes of *S. cerevisiae* strains involved in industrial production of fuel ethanol or saké [37,38]. Knq1p, a functionally characterized DHA2 transporter of *Kluyveromyces lactis*, was found to be involved in oxidative stress response and iron homeostasis [39,40]. This protein was found to define a new branch in a phylogenetic tree constructed using the DHA2 proteins encoded in the genomes of 5 hemiascomycete yeasts [41].

The evolutionary history of the DHA1 genes present in the genome of 13 hemiascomycete yeast species was reconstructed by combining building tree methodologies with gene neighbourhood analysis [42]. Gene neighbourhood analysis is a comparative genome based-approach used to infer gene lineages [42,43]. In the present study, we undertook the clustering of the amino acid sequences of a total of 172,422 translated ORFs obtained from 31 sequenced yeast strains from 25 different hemiascomycetous species to construct a homogenous protein classification system which was used to trace back the evolutionary history of genes encoding DHA2 transporters in the Hemiascomycetes. Combined with tree construction methods, this approach allowed the most comprehensive phylogenetic characterization of the hemiascomycetous DHA2 proteins available to date. The results obtained during this study also suggest that the DHA2, ARN and GEX transporters are closely related families.

## Methods

### Hemiascomycete yeast genomes

The translated ORFs of the 31 sequenced hemiascomycetous strains analysed in this work were retrieved from the genome databases indicated in Table 1. These 31 hemiascomycetous strains correspond to 25 different species, 14 of which belong to the *Saccharomyces* complex, 9 to the CTG complex and 2 are the early-divergent hemiascomycetes, *Pichia pastoris* and *Yarrowia lipolytica*. Henceforth, the four letters code shown in Table 1 for species abbreviation will be used to designate both yeast genes and species. The letter displayed after the first four letters is used to abbreviate the strain name when the genome of more than one strain from a given species is available or when the genome of the same strain was sequenced by different research centres. To uniformize the annotation used, translated ORFs are always represented with small letters.

**Table 1:** Hemiascomycetous strains examined during this work

**Species**	**Strain**	**Acronym**	**Phylogenetic complex**	**Speciation in relation to WGD**	**Coverage**	**Genome size (Mb)**	**Database**
*Saccharomyces cerevisiae*	S288C	sace_a	Saccharomyces complex	Post	Complete	12.2	1
	EC1118	sace_b		Post	24X	11.7	2
	JAY291	sace_c		Post	165X	11.5	2
	RM11-1A	sace_d		Post	10X	11.7	3
	YJM789	sace_e		Post	10X	12.0	2
*Saccharomyces paradoxus*	NRRL Y-17217	sapa		Post	7.7X	11.9	4
*Saccharomyces mikatae*	IFO 1815	sami_a		Post	5.9X	11.5	5
	IFO 1815	sami_b		Post	2.8X	10.8	6
*Saccharomyces bayanus*	623-6C	saba_a		Post	2.9X	11.9	7
	MCYC 623	saba_b		Post	6.4X	11.5	8
*Saccharomyces kudriavzevii*	IFO 1802	saku		Post	3.4X	11.2	9
*Saccharomyces castelli*	NRRL Y-12630	saca		Post	3.9X	11.2	7
*Candida glabrata*	CBS138	cagl		Post	Complete	12.3	10
*Kluyveromyces polysporus*	DSM 70294	klpo		Post	7.8X	14.7	7
*Zygosaccharomyces rouxii*	CBS 732	zyro		Pre	Complete	9.8	10
*Saccharomyces kluyveri*	CBS3082	sakl		Pre	Complete	11.5	10
*Kluyveromyces waltii*	NRRL Y-12651	klwa		Pre	8X	10.9	10
*Kluyveromyces thermotolerans*	CBS6340	klth		Pre	Complete	10.4	10
*Kluyveromyces lactis*	CLIB210	klla		Pre	Complete	10.7	10
*Eremothecium gossypii*	ATCC10895	ergo		Pre	Complete	9.1	10
*Candida albicans*	SC5314	caal_a	CTG complex	Pre	10.4X	27.6	11
	WO-1	caal_b			10X	21.7	11
*Candida dubliniensis*	CD36	cadu			11X	14.6	11
*Candida tropicalis*	MYA-3404	catr			10X	14.6	11
*Candida parapsilosis*	CDC 317	capa			9.2X	13.1	11
*Lodderomyces elongisporus*	NRLL YB-4239	loel			8.7X	15.5	11
*Candida guilliermondii*	ATCC 6260	cagu			12X	10.6	11
*Debaryomyces hansenii*	CBS767	deha			Complete	12.2	10
*Pichia stipitis*	CBS 6054	pist			-	15.4	12
*Candida lusitaniae*	ATCC 42720	calu			9X	12.1	11
*Pichia pastoris*	GS115	pipa	Early-divergent	Pre	20X	9.2	13
*Yarrowia lipolytica*	CLIB122	yali	Early-divergent	Pre	Complete	20.6	10

Information regarding strain acronyms, the corresponding phylogenetic complex, phylogenetic position concerning the WGD event, genome coverage, estimated genome size and database from where the genome sequence was retrieved is shown.

http://1-http://downloads.yeastgenome.org/sequence/S288C_reference/orf_protein/orf_trans_all.fasta.gz.

http://2-http://www.ncbi.nlm.nih.gov/.

http://3-http://www.broadinstitute.org/annotation/genome/saccharomyces_cerevisiae.3.

http://4-http://downloads.yeastgenome.org/sequence/fungi/S_paradoxus/archive/MIT/orf_protein/.

http://5-http://downloads.yeastgenome.org/sequence/fungi/S_mikatae/archive/MIT/orf_protein/.

http://6-http://downloads.yeastgenome.org/sequence/fungi/S_mikatae/archive/WashU/orf_protein/.

http://7-http://wolfe.gen.tcd.ie/ygob/.

http://8-http://downloads.yeastgenome.org/sequence/fungi/S_bayanus/archive/MIT/orf_protein/.

http://9-http://downloads.yeastgenome.org/sequence/fungi/S_kudriavzevii/archive/WashU/orf_protein/.

http://10-http://www.genolevures.org/download.html#.

http://11-http://www.ebi.ac.uk/integr8.

http://12-http://srs.ebi.ac.uk/srsbin/cgi-bin/wgetz.

http://13-https://bioinformatics.psb.ugent.be/gdb/pichia/pipas_genes-1009.pep.

### Sequence clustering of the translated ORFs

The comparative genomic approach used in this study is based on the sequence clustering of all translated ORFs of the 31 sequenced yeast strains. This required the compilation and organization of a total of 172,422 translated ORFs. These translated ORFs were organized into a blast database and compared all-against-all using blastp algorithm made available in blast2 package [44]. The blastp algorithm used gapped alignment with the following parameter: expectation value (10^-30^), open gap (−1), extend gap (−1), threshold for extending hits (11) and word size (3). This approach generated a total of 31 million pairwise alignments. In order to handle this amount of data, sequence clustering was formulated as a graph traversal problem, where the nodes are the translated ORFs and the edges indicate the existence of pairwise sequence similarity between amino acid sequences. Classification of the translated ORFs into clusters was achieved by breadth-first traversing this network at different e-value thresholds, ranging from E-30 to E-12.

### Gene neighbourhood analysis

During this work, a MySQL genome database was built compiling a series of genomic information regarding the previously mentioned translated ORFs. This genomic information includes gene name, chromosome/contig, sequence clustering classification, gene start position, gene end position, amino acid sequence length and encoded amino acid sequences. The package “sqldf” [45] and complementing scripting in R language was used to retrieve fifteen neighbour genes on each side of the query genes as well as the corresponding sequence clustering classification from this hemiascomycetous genome database. The rationale of synteny analysis resides on the assumption that two genes of different yeast species whose translation products belong to the same sequence cluster (homologues by similarity) will be members of the same gene lineage if they share at least one pair of neighbours that are also homologous to each other by similarity [42,43]. The process is reiterated for all possible heterospecific pairwise comparisons of homologues deduced from the sequence clusters. The sequence clustering classification of the thirty genes neighbouring each query gene was done using a conservative blastp e-value of E-30 to limit the number of false positive sequences incorporated together with true cluster members. When further evidences were needed to corroborate dubious synteny connections between genes, sequence clustering was performed at a less restrictive e-value threshold (E-15).

The chromosome neighbourhood of the query genes was converted into a format adequate for import into the Cytoscape environment [46]. In the resulting networks, nodes represent query genes and edges represent pairs of neighbouring genes classified in the same sequence cluster. Useful biological information indicated below was imported into the Cytoscape network as edges attributes. The existence of synteny between query genes was verified through the analysis of network topology (number of shared neighbour pairs) and the biological information associated with the corresponding edges. The advantage of this framework is that it allows scrutinizing the synteny relationships established between genes in a simple mathematical context of network topology exploration. Three sources of biological information were used to assess the strength of each neighbour pair connection [42]: i) closeness of the connecting neighbours in relation to the query genes, ii) sequence similarity between connecting neighbours and iii) dimension of the sequence cluster to which the homologous neighbours belong; small dimension of the sequence cluster indicates that it is small the probability that two homologous neighbours are in the vicinity of two query genes by chance.

### Topology prediction, sequence alignment and phylogenetic tree building

The topology of the amino acid sequences was analysed using HMMTOP and TMHMM 2.0 bioinformatics tools [47,48]. For each amino acid sequence not showing 14 TMS and with less than 490 residues in length, the TMS range was predicted by visual analysis of the topology probability and protein hydrophobicity plots generated by TMHMM 2.0 and TOPPRED 2 [49,50], respectively.

Multiple alignments of the amino acid sequences were calculated by MUSCLE [51] and processed using the PHYLIP package [52]. PROTDIST/NEIGHBOUR and PROML packages were used to generate the phylogenetic trees based on distance and maximum likelihood methods, respectively. The Dendroscope application was used for tree visualization [53].

Sequence identity and similarity shared between protein pairs was assessed using an all-against-all Needleman-Wunsch alignment approach. This algorithm was run using the needle package available in the EMBOSS suite [54]. All needle pairwise alignments made in this work used default values for the gap open and gap extension parameters, 10.0 and 0.5, respectively. After the construction of the DHA2 phylogenetic tree, all-against-all Needleman-Wunsch alignments were also constructed for the members of each phylogenetic cluster.

## Results

### Identification of the DHA2 proteins in 31 hemiascomycetous strains

**Figure 1:**
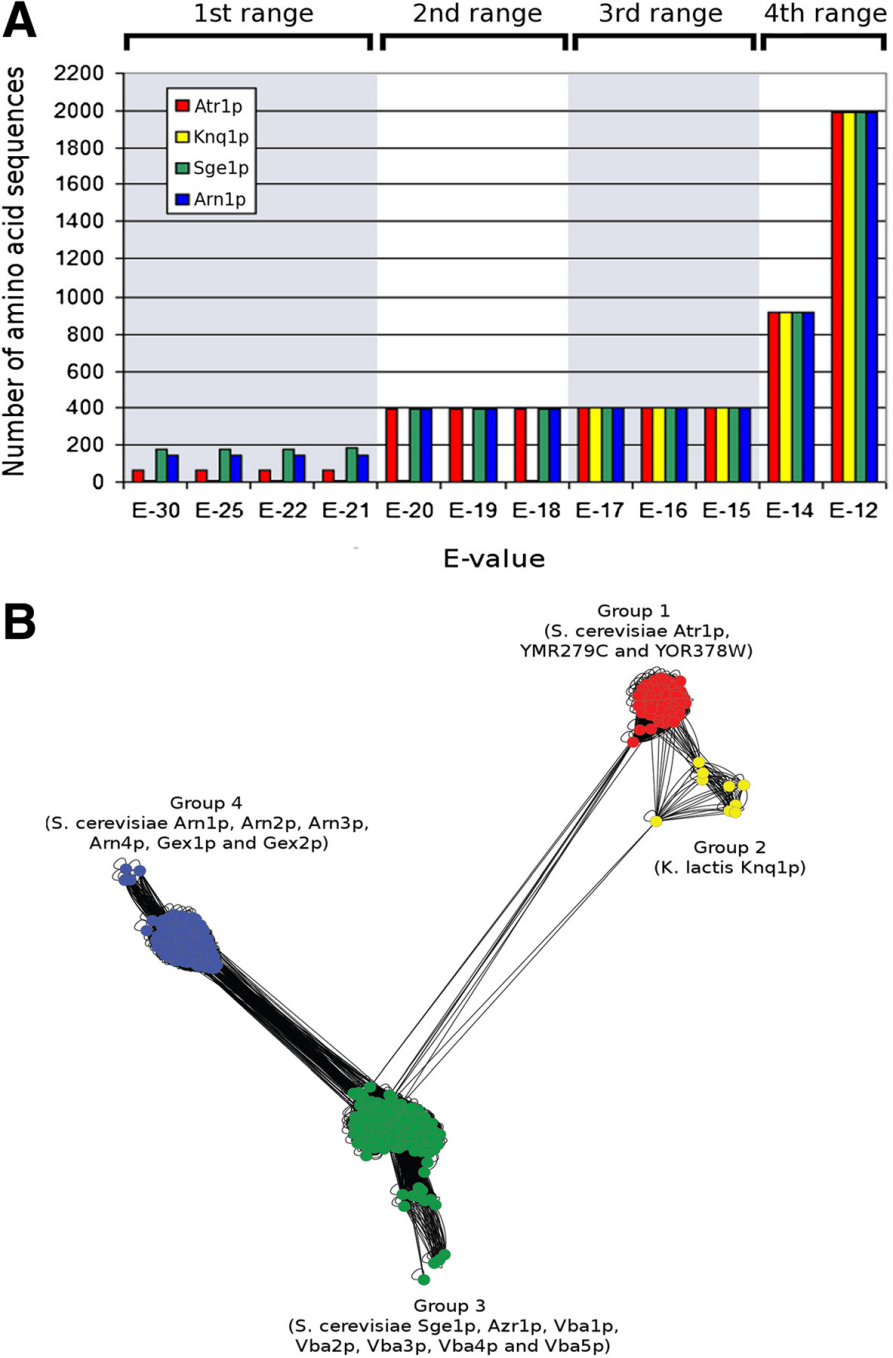
**Identification of the 14-spanner MFS-MDR proteins encoded in 31 hemiascomycetous genomes. A)** Plot representing the number of sequences retrieved after constraining and traversing the pairwise similarity network at different e-values using Atr1p, Knq1p, Sge1p and Arn1p as starting nodes. **B)** Network representing the blastp relationships linking the 14-spanner MFS-MDR proteins gathered at an e-value level of E-15 (starting node Atr1p). The distances separating the amino acid sequences in this network were calculated based on the pairwise sequence alignment e-values and using the Cytoscape layout option “Edge-weighted spring embedded”.

The constraining and traversing of a pairwise similarity network allowed the identification of the DHA2 proteins encoded in the genomes of 31 hemiascomycetous strains. The functionally characterized Atr1 protein was used as starting node for the network traversal. Analysis of the plot representing the number of sequences retrieved at different e-values shows the existence of four distinct blastp clustering ranges. The first range occurs between e-values E-30 to E-21, gathering 68 amino acid sequences highly similar to the starting node Atr1p (group 1), including *S. cerevisiae* ORFs YMR279C and YOR378W (Figure 1B). In the second blastp clustering range (e-values from E-20 to E-18) occurs the merge of the amino acid sequences comprised in groups 3 and 4 (182 and 143 members, respectively) with those of group 1 (Figure 1B), originating a total of 393 sequences. Group 3 comprises the *S. cerevisiae* DHA2 transporters Sge1, Azr1, Vba1, Vba2, Vba3, Vba4 and Vba5. Group 4 comprises the amino acid sequences of *S. cerevisiae* ARN and GEX 14-spanner MFS transporters. Members of clusters 3 and 4 are linked by many connections, indicating the existence of strong homology between amino acid segments of these transporters. In the third blastp clustering range (e-values from E-17 to E-15), group 2, comprising the biochemically characterized DHA2 transporter of *K. lactis* species, Knq1p, is merged with groups 1, 3 and 4. The resulting supergroup contains a total of 402 amino acid sequences. Analysis of the number of TMS shown by these amino acid sequences (see Additional file 1) together with the construction of a phylogenetic tree (see Additional file 2) confirmed that all true 14-spanner transporters were gathered at this blastp clustering range. In the fourth blastp clustering range (e-values equal or bellow E-14), false positive amino acid sequences are incorporated with the true 14-spanner MFS proteins.

The joint retrieval of the DHA2, ARN and GEX proteins by this *in silico* approach suggests the existence of a close phylogenetic relationship linking these transporters. Consistent with this indication, the DHA2, ARN and GEX proteins encoded in the genome of *S. cerevisiae* S288C strain were clustered in a single protein family, CL3C0009, by the Génolevures Consortium [55]. These results were confirmed by breadth-first traversing this network using the functionally characterized Sge1, Vba1, Vba4, Arn1, Arn3, Arn4, Gex1 and Knq1 proteins as starting nodes. For the sake of clarity, only the results of Atr1p, Knq1p, Sge1p and Arn1p are shown in Figure 1A since the remaining amino acid sequences clustered with one of these four proteins (Figure 1B).

### Phylogenetic analysis of the hemiascomycetous DHA2, ARN and GEX transporters

**Figure 2:**
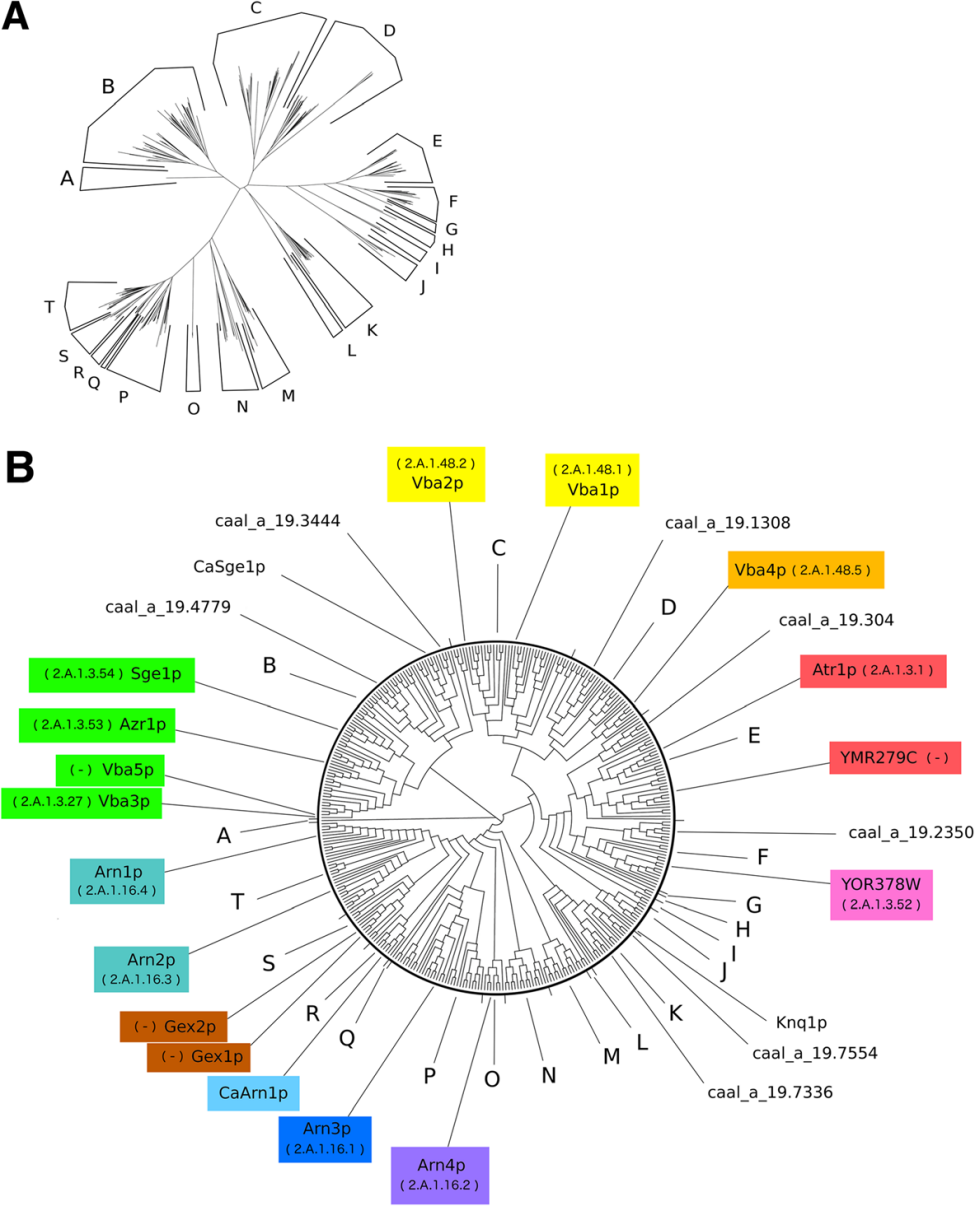
**Phylogenetic analysis of DHA2, ARN and GEX transporters gathered from 31 yeast strains from 25 hemiascomycetous species. A)** Radial phylogram showing the amino acid sequence similarity distances between these 355 full-size 14-spanner MFS transporters. **B)** Circular cladogram showing the tree topology. PROTDIST/NEIGHBOR packages of PHYLIP suite were used in the analysis. Protein and translated ORF names can be consulted in Additional file 2. The name of the *S. cerevisiae* and *C. albicans* members is indicated as well as the biochemically characterized Knq1 transporter of *K. lactis*. The gene and species annotation adopted in this study uses the four letters code described in Table 1. The TCDB protein family classification of *S. cerevisiae* proteins is also indicated inside parenthesis.

The analysis of the hydrophobicity and topology of the 14-spanner proteins gathered in the third blastp clustering range revealed that 355 of these comprised full-size transporters while 47 were fragments (see Additional file 1). A phylogenetic tree representing the full-size DHA2, ARN and GEX proteins was built and divided into 20 clusters, labelled from A to T (Figure 2A,B and Additional file 3 for protein/translated ORFs names). Since the distance and the maximum likelihood methods originated similar phylogenetic trees regarding cluster composition (see Additional file 4 and Additional file 5), only the tree obtained using the distance method is shown (Figure 2). The stability of cluster composition of the phylogenetic trees obtained by the distance and maximum likelihood methods supported the division of the phylogenetic tree into the 20 clusters. To avoid tree artefacts resulting from root positioning, the pist_igi19985888 protein was manually chosen as root for the phylogenetic tree (cluster A) since this protein does not cluster together with any of the other amino acid sequences. The neddle package of EMBOSS suite was used to make all possible pairwise alignment combinations between the full-size 14-spanner MFS-MDR proteins. Pairwise sequence comparisons revealed that the sequence identity of transporters residing in the same phylogenetic cluster ranged from 36.5% to 94.2% whereas sequence similarity ranged from 53.0% to 97.1%.

Using as reference the DHA2, ARN and GEX transporters encoded in the *S. cerevisiae* S288C genome, this phylogenetic tree (Figure 2) was used to assign sequence homology to the 14-spanner transporters encoded in the genomes of the remaining hemiascomycetous strains (Table 2 and Additional file 1). For example, regarding the siderophore transporters arsenal present in the genome of the ten *Candida* pathogenic species considered in this study, the analysis of Table 2 shows that *C. albicans* and *C. dubliniensis* only possess *Ca*Arn1 type of siderophore transporters (cluster R), *C. tropicalis* and *C. parapsilosis* exhibit both *Ca*Arn1 and Arn3 types of siderophore transporters (cluster R and cluster P, respectively) and *C. glabrata*, an opportunistic yeast pathogen belonging to the *Saccharomyces* complex, only exhibits one siderophore transporter (the ortholog of Arn1 gene). Eight translated ORFs in the genome of *C. albicans* SC5314 were iden-tified as *bona fide* DHA2 transporters: caal_a_19.304, caal_a_19.2350, caal_a_19.1942, caal_a_19.4779, caal_a_19.3444, caal_a_19.1308, caal_a_19.7554 and caal_a_19.7336. An additional ORF, orf19.2923, was proposed before as encoding a DHA2 protein [56] but the corresponding amino acid sequence shares high similarity with *S. cerevisiae* ORF YMR155W, a putative protein of unknown function, identified as interacting with Hsp82p [57]. Consistent with our proposal, the protein classification system developed by the Génolevures consortium also clustered YMR155W amino acid sequence in the GL3C0730 family while the *S. cerevisiae* DHA2 proteins are grouped in the GL3R0009 family. No association could be found between the presence of particular genes encoding DHA2, ARN and GEX transporters in the yeast genomes examined and the corresponding species pathogenicity (Table 2 and Additional file 6 and Additional file 7).

**Table 2:** Number of full size DHA2, ARN and GEX proteins in each cluster for a specific yeast strain

**Subfamily**				**DHA2**													**ARN**								**GEX**
**Acronym**					**(Sge1/Azr1/Vba3/Vba5)**	**(Vba1/Vba2)**	**(Vba4)**	**(Atr1/YMR279C)**	**(YOR378W)**				**(Knq1)**						**(Arn4)**	**(Arn3)**		**( *Ca *Arn1)**	**(Arn1/Arn2)**		**(Gex1/Gex2)**
**Cluster**				**A**	**B**	**C**	**D**	**E**	**F**	**G**	**H**	**I**	**J**	**K**	**L**		**M**	**N**	**O**	**P**	**Q**	**R**	**T**		**S**
Saccharom. complex		sace_a			4	2	1	2	1										1	1			2		2
		sace_b			2	2	1	2											1	1			2		1
		sace_c			2	2	1	2	1				1					1		1			1		1
		sace_d			3	2	1	2	1										1	1			1		1
		sace_e			4	1	1	2	1											1			1		1
		sapa			4	2		2	1				1					1	1	1			1		2
		sami_a			2	2	1	2	1				1						1	1			1		
		sami_b			2				1																1
		saba_a				2		2	1				1					1	2	1			1		
		saba_b				1		2	1				1					1		1			1		
		saku			1	2	1	1	1									1					1		
		saca			1	1		3			1									2			1		
		cagl			1	1		2			1												1		
		klpo					1	1												4					
		zyro				4	1																1		
		sakl			5	2	3	1					1							2			4		
		klwa			2	3	2	1	1				1							2			3		1
		klth			6	3	1	1	1				1							2			5		1
		klla			2	1	2		1				1										4		1
		ergo				1	1																		
CTG complex		caal_a			3		1	1	1					2								1			
		caal_b			3		1	1						2								1			
		cadu			3		1	1	1					2								1			
		catr			4			1	1					2						1		1			
		capa			3		1		1					1						3		2			
		loel			2		1		1					1								1			
		cagu			2	2	1	1	2	1				2				3		2	1				
		deha			2	1	1	1	1					2				4		1					
		pist		1	7	1		1	2					2						1		1			
		calu			4		1	1						1						1					
Early-div.		pipa			1	1	2					1						2		1					
Early-div.		yali					1					2		1	2		13								
Total				1	75	39	29	36	23	1	2	3	9	18	2		13	14	7	31	1	8	31		12
Identity (%)				-	36.5	40.8	38.6	55.7	58.8	-	65.1	63.6	65.1	53.4	70.1		44.0	42.6	94.2	52.4	-	74.0	60.9		67.8
Similarity (%)				-	53.0	56.0	53.2	70.0	72.3	-	74.9	73.8	76.5	69.8	77.5		59.5	58.7	97.1	67.7	-	84.8	75.5		78.3

Information regarding total number of full size transporters and their average percentage of sequence identity and similarity is shown.

The family functional classification of each *S. cerevisiae* 14-spanner MFS transporter was retrieved from the Transporter Classification Database (TCDB) [7] and the information was added to Figure 2. The TCDB classification divides the genes that have been considered as encoding the *S. cerevisiae* DHA2 transporters into two families. “The Drug:H^+^ Antiporter-2 (14 Spanner) (DHA2) Family” (2.A.1.3), comprising *ATR1*, *SGE1*, *AZR1* and *VBA3* genes and ORF YOR378W, and “The Vacuolar Basic Amino Acid Transporter (V-BAAT) Family” (2.A.1.48), comprising *VBA1*, *VBA2* and *VBA4* genes. The *VBA5* gene and the ORF YMR279C, historically considered as members of the DHA2 protein family [19], do not have family classification in TCDB database. The proteins encoded by *ARN1*, *ARN2*, *ARN3* and *ARN4* genes reside in a single TCDB family, “The Siderophore-Iron Transporter (SIT) Family” (2.A.1.16). The *GEX1* and *GEX2* encoded transporters are also not included in TCDB database.

### Identification of DHA2, ARN and GEX gene lineages in the Hemiascomycetes

Gene neighbourhood analysis of the chromosome environment where the DHA2, ARN and GEX genes reside allowed the identification of thirteen gene lineages (see Additional file 8). This analysis involved the representation of synteny between genes in a network framework and, subsequently, the exploitation of the network topology in Cytoscape software environment. DHA2 genes encoding transporters present in phylogenetic clusters A, G, H, I and L did not reside in a conserved chromosome environment, as it is the case of the genes encoding siderophore transporters residing in clusters M and Q. However, in general, genes belonging to a given lineage encode transporters present in the same phylogenetic cluster. The DHA2, ARN and GEX gene lineages spanning the species of the *Saccharomyces* complex, CTG complex and the early-divergent hemiascomycetes, *P. pastoris* and *Y. lipolytica*, are detailed bellow. The order of speciation of the yeasts belonging to the CTG complex adopted in this work was based on the order used in previous phylogenetic studies on Hemiascomycetes [58-61].

### DHA2 gene lineages

**Figure 3:**
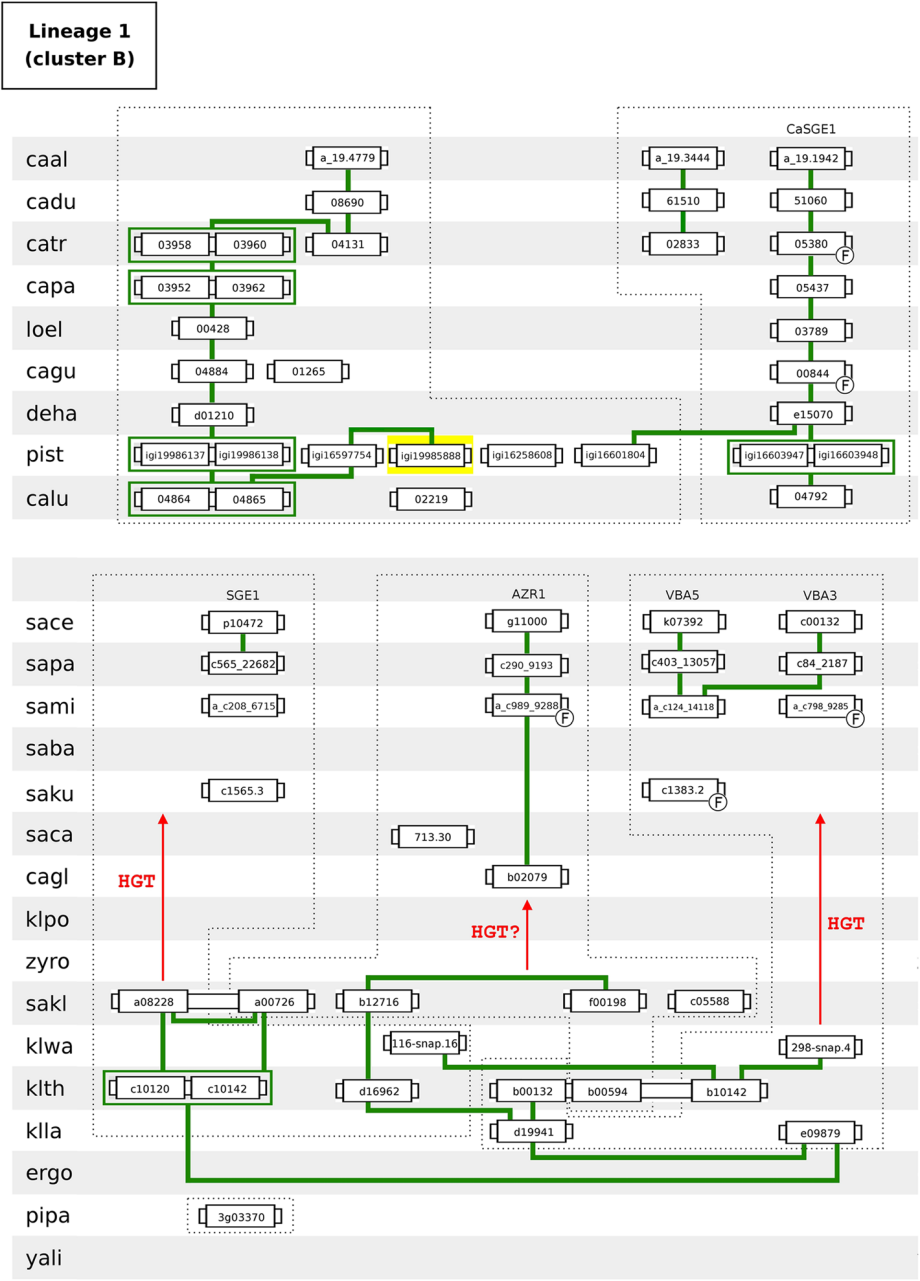
**Lineage 1 (homologs of *S. cerevisiae SGE1*/*AZR1*/*VBA3*/*VBA5 *genes).** Each box represents a gene. Lines connect genes sharing common neighbours. Yellow background represents DHA2 genes not belonging to the phylogenetic cluster associated to the lineage. F indicates that the corresponding gene was classified as a fragment. The broken line encompasses groups of proteins more similar in amino acid sequence (inferred from the analysis of the phylogenetic tree). HGT represents the plausible occurrence of events of horizontal gene transfer between species.

**Figure 4:**
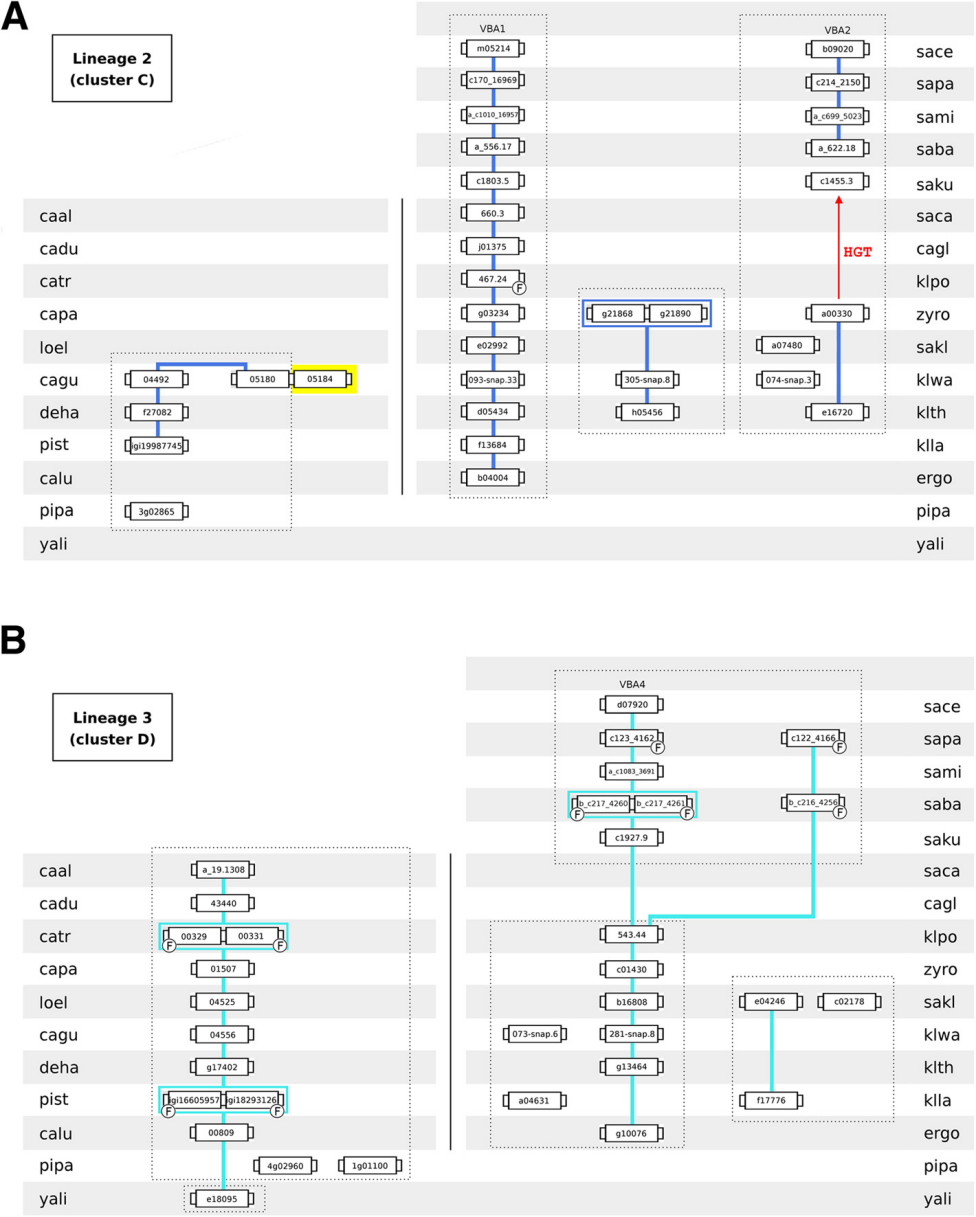
**Lineages 2 (homologs of *S. cerevisiae VBA1*/*VBA2 *genes) and 3 (homologs of *S. cerevisiae VBA4 *gene).** Conventions as in Figure 3.

**Figure 5:**
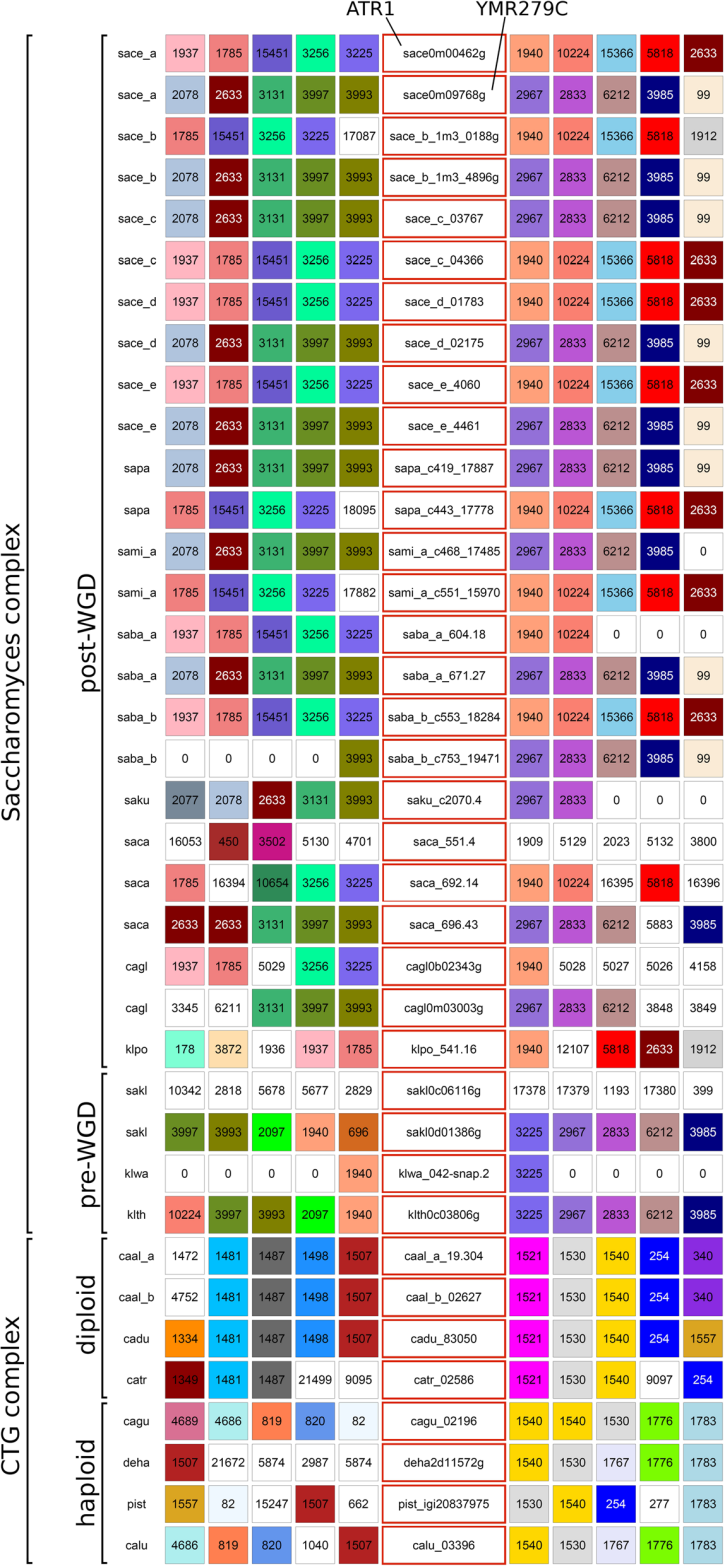
**Gene neighbourhood of *ATR1 *gene, ORF YMR279C and corresponding homolog genes (central boxes).** Adjacent boxes represent gene neighbours. The yellow background represents genes not belonging to the phylogenetic cluster associated to the lineage. Homologous neighbours are highlighted in the same color. A white box represents genes with no homologous neighbours in the represented chromosome region. The synteny was assessed with 15 neighbours on each side but, for the sake of clarity, this representation was truncated to 5 neighbours (see Additional file 8 for full neighbourhood details).

**Figure 6:**
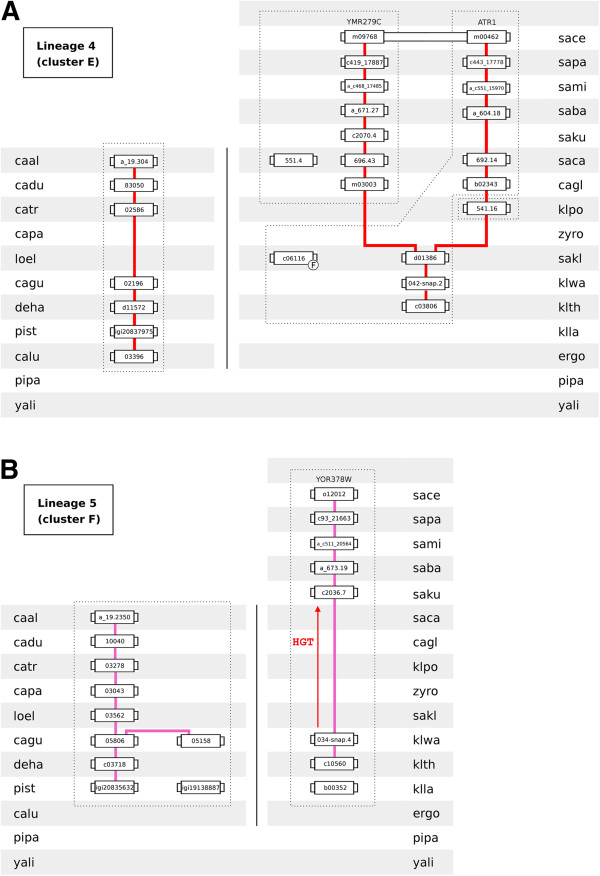
**Lineages 4 (homologs of *S. cerevisiae ATR1*/YMR279C genes) and 5 (homologs of *S. cerevisiae *ORF YOR378W).** Conventions as in Figure 3.

**Figure 7:**
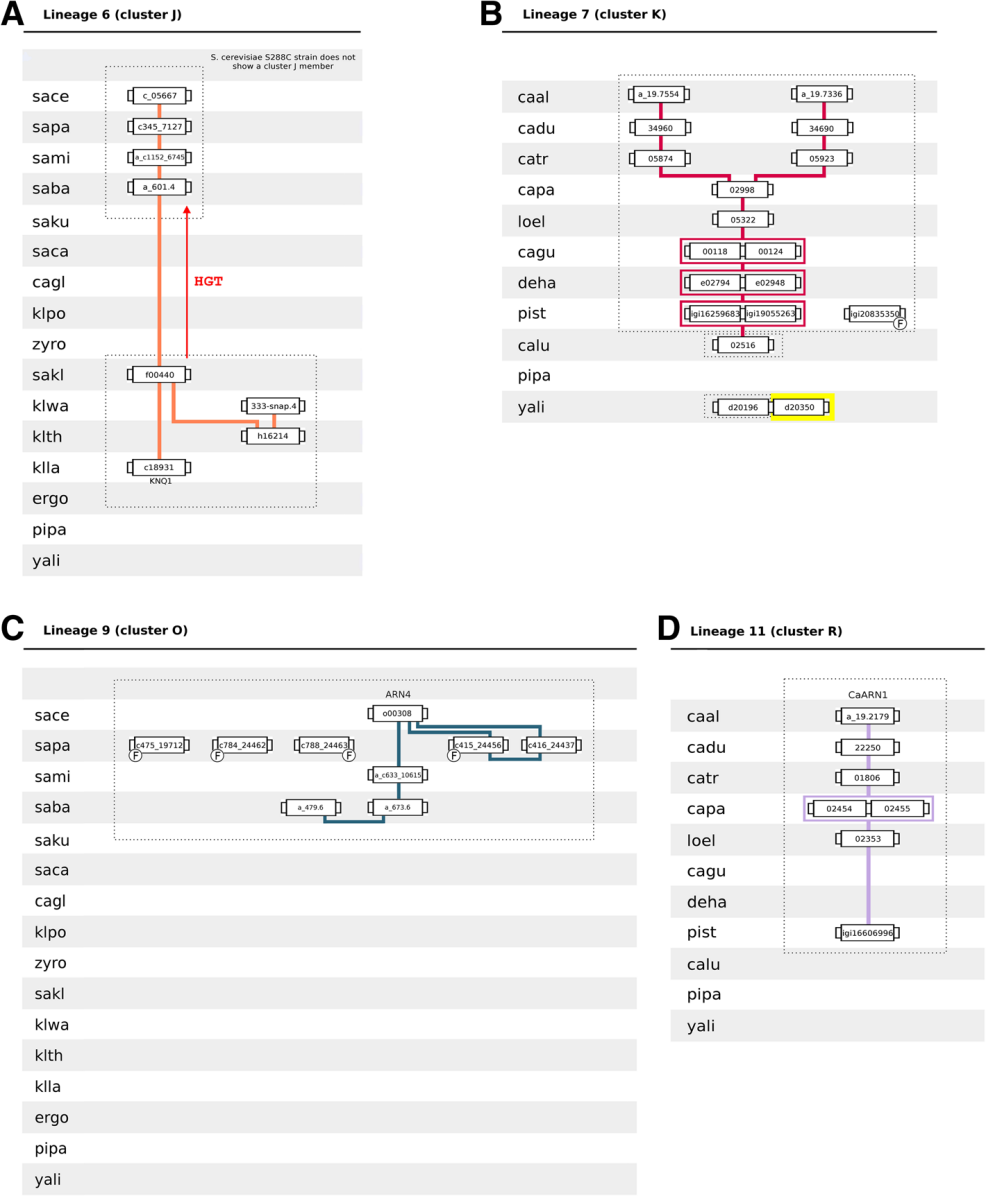
**Lineages 6 (homologs of *K. lactis KNQ1 *gene), 7 (cluster K proteins), 9 (homologs of *S. cerevisiae ARN4 *gene) and 11 (homologs of *C. albicans CaARN1 * gene).** Conventions as in Figure 3.

The gene neighbourhood analysis allowed the identification of seven DHA2 gene lineages in the 31 hemiascomycetous strains examined (Figures 3, 4, 5, 6, 7A and 7B). Five of these lineages include the ten DHA2 genes encoded by the genome of *S. cerevisiae* S288C reference strain.

Lineage 1 comprises seven sublineages (Figure 3). With the exception of pist_igi19985888 gene, a member of cluster A, lineage 1 comprises cluster B-encoding genes. In the *Saccharomyces* complex, three sublineages converge on *S. cerevisiae SGE1*, *AZR1* and *VBA3*/*VBA5* genes. The *S. cerevisiae VBA3* and *VBA5* genes are paralogs originated in a duplication event occurring after *S. mikatae* speciation. The sequenced genomes of *Z. rouxii*, *K. polysporus*, *C. glabrata* and *S. castellii* species do not possess *SGE1* or *VBA3*/*VBA5* homologs, suggesting that these genes were acquired by the ancestral of the *Saccharomyces sensu strictu* group (SSSG) by lateral transference. In addition, no synteny was observed between the *AZR1* homologs of Lachancea and those of SSSG species. In the CTG complex, three sublineages converge on *C. albicans CaSGE1* gene and on ORFs caal_a_19.3444 and caal_a_19.4779. Two sublineages encompass DHA2 genes belonging to CTG haploid sub-group species while the origin of the third sublineage is more recent. The cluster B ORF of the early-divergent species *P. pastoris* (pipa_3g03370) resides in a distinct chromosome environment.

With the exception of five amino acid residues and the presence of an extra peptide of 124 amino acids in the N terminus, the amino acid sequences encoded by *VBA5* and *VBA3* genes are identical. Due to this fact, the plasma membrane localization of Vba5p was hypothesized to be dependent on the presence of the extra N-terminal amino acid sequence [29]. The analysis of the amino acid sequences of the *VBA3* and *VBA5* homologs showed that, with exception of *VBA3* gene (encoded in the *S. cerevisiae* S288C genome) and ORF sace_e_3474 (encoded in the *S. cerevisiae* YJM789 genome), all these genes carry a similar extra N-terminal peptide (see Additional file 9). In addition, the analysis of translated DNA sequence of the upstream region of *VBA3* gene and ORF sace_e_3474 showed that their N-terminal peptides are still encoded in the genomes of the corresponding *S. cerevisiae* strains. The N-terminal peptide of ORF sace_e_3474 is miss-predicted due to the localization of this ORF in the extremity of the DNA contig (its sequence is partly truncated) while the coding sequence of the N-terminal peptide in the *VBA3* gene is disrupted by a stop codon (see Additional file 10).

Lineage 2 comprises the homologs of *S. cerevisiae VBA1*/*VBA2* genes (cluster C). This lineage is divided into four sublineages (Figure 4A). One extends from ergo2b04004 to the *S. cerevisiae VBA1* gene, encompassing genes from all species belonging to the *Saccharomyces* complex considered in this study. The genomes of *K. polysporus, C. glabrata* and *S. castellii* lack a *VBA2* homolog, resulting in a lineage discontinuity occurring in the transition from pre- to post-WGD species. The third sublineage is composed by two *K. waltii* and *K. thermotolerans* genes and by a tandem repeat present in *Z. rouxii* genome. The last sublineage spans genes of the CTG haploid species. The *VBA1*/*VBA2* homolog of the early-divergent hemiascomycetes *P. pastoris* (pipa_3g02865) does not share common neighbours with the remaining cluster C members.

Lineage 3 comprises the homologs of *S. cerevisiae VBA4* gene (cluster D). Only the genomes of *C. glabrata* and *S. castellii* lack a cluster D-encoding gene (or fragment) in the 31 hemiascomycetous strains considered in this study. The sublineage encompassing the *Saccharomyces* complex species extends from ergo2g10076 to the *S. cerevisiae VBA4* gene (Figure 4B). The paralog of *VBA4* gene was quickly lost after the WGD event. The chromosome environment where the *VBA4* homologs reside in the species of the CTG complex is highly conserved, encompassing also ORF yali0e18095 belonging to the early-divergent hemiascomycete yeast species *Y. lipolytica*. Two ORFs classified in cluster D present in the *P. pastoris* genome (pipa_4g02960 and pipa_1g01100) do not share common neighbours with the remaining lineage 3 genes.

Lineage 4 comprises the homologs of *S. cerevisiae ATR1* gene and ORF YMR279C (cluster E). The chromosome environment where *ATR1* and YMR279C homologs reside is conserved (Figure 5). The evolutionary history of these genes reproduces a typical WGD pattern, where a pre-WGD lineage splits into two sublineages, each of which gave rise to the *S. cerevisiae ATR1* and YMR279C paralogs (Figure 6A). Regarding the CTG complex, cluster E-encoding genes show a linear evolutionary history converging on ORF caal_a_19.304. The early-divergent hemiascomycetes *P. pastoris* and *Y. lipolytica* do not possess cluster E members.

Lineage 5 comprises the homologs of *S. cerevisiae* ORF YOR378W (cluster F). This lineage divides into two sublineages (Figure 6B). The sublineage spanning the species of the *Saccharomyces* complex shows a discontinuity occurring in the transition from pre- to post-WGD species. Although klwa_034-snap.4 and klth0c10560 share two common neighbours with the post-WGD YOR378W homologs, the size of this discontinuity suggests that the species of the SSSG have acquired their cluster F-encoding genes by lateral gene transfer, plausibly from a pre-WGD yeast. The sublineage spanning the species of the CTG complex converges on *C. albicans* ORF caal_a_19.2350. The genomes of *C. lusitaniae* and the early-divergent hemiascomycetes *P. pastoris* and *Y. lipolytica* lack a cluster F-encoding gene.

Lineage 6 comprises the homologs of *K. lactis KNQ1* gene (cluster J). Besides *K. lactis*, only the genomes of SSSG and Lachancea species possess cluster J-encoding genes. However, with the exception of JAY-291 strain, the *S. cerevisiae* strains considered in this work lack a cluster J-encoding gene. The lack of *KNQ1* homologs by *Z. rouxii*, *K. polysporus*, *C. glabrata*, *S. castellii* and *S. kudriavzevii* species suggests that the ancestral of the SSSG have acquired their cluster F-encoding genes by lateral gene transfer, plausibly from a pre-WGD donor species (Figure 7A). The genomes of both early-divergent hemiascomycetes lack a cluster F-encoding gene.

Lineage 7 comprises genes of species belonging to the CTG complex (cluster K). A duplication event occurring after the speciation of *C. parapsilosis* originated two sublineages, converging each on *C. albicans* ORFs caal_a_19.7554 and on caal_a_19.7336 (Figure 7B). With the exception of yali0d20196, cluster K-encoding genes reside in a conserved chromosome environment.

### ARN gene lineages

**Figure 8:**
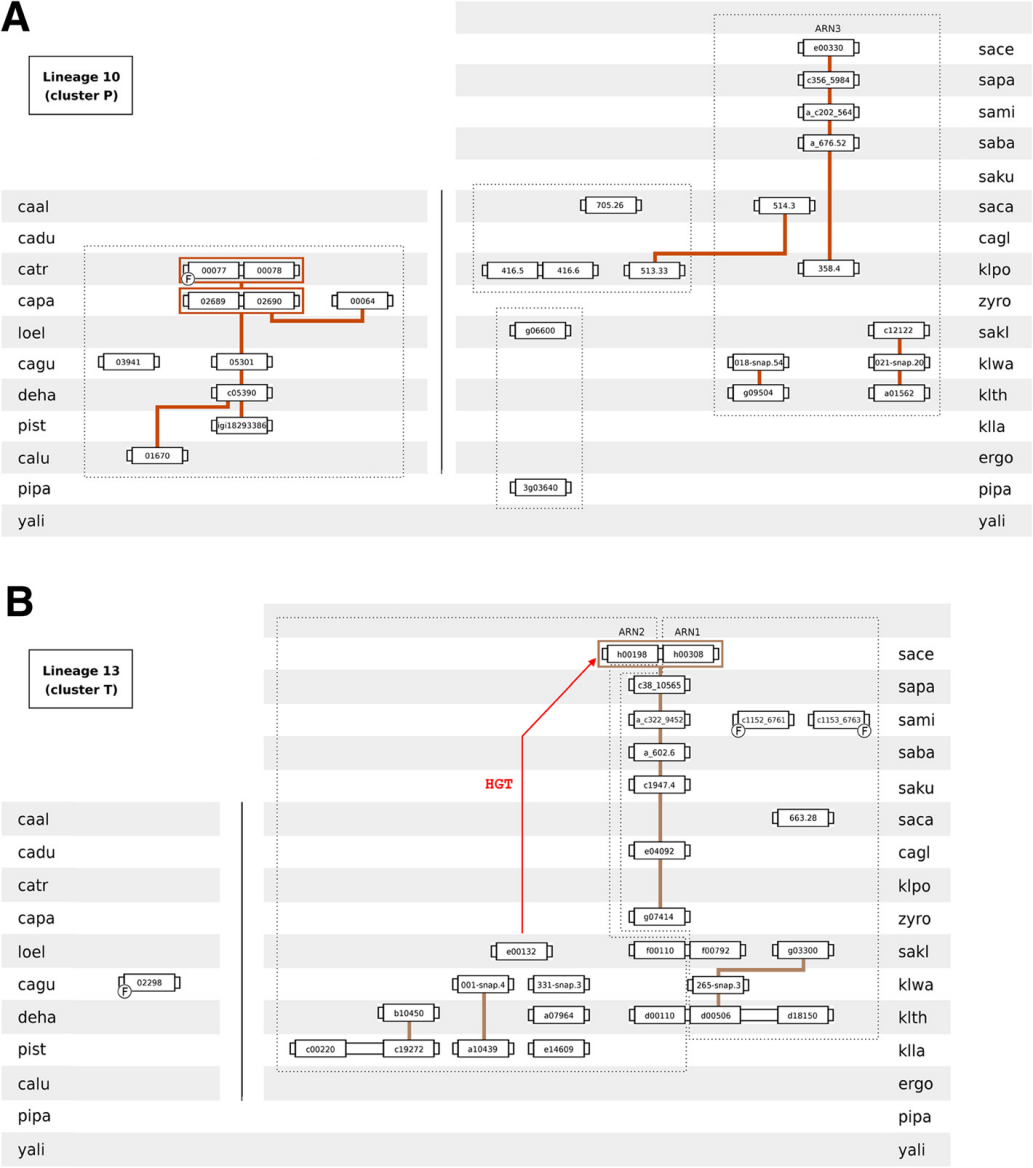
**Lineages 10 (homologs of *S. cerevisiae ARN3 *gene) and 13 (homologs of *S. cerevisiae ARN1/ARN2 *genes).** Conventions as in Figure 3.

This study identified four lineages comprising genes encoding siderophore transporters (Figures 7C,D and 8). Three of these lineages comprise the four ARN genes encoded in the genome of *S. cerevisiae* S288C reference strain [9-12] and one additional lineage comprises the sole *C. albicans* gene encoding a siderophore transporter [62,63]. The members of the ARN gene lineages, as described in [16], reside in the phylogenetic cluster 2.A.1.16.Z1.

Lineage 9 comprises the homologs of *S. cerevisiae ARN4* gene (cluster O). These genes are only found in the genomes of SSSG species (Figure 7C). Of the five *S. cerevisiae* strains considered in this work, only the genomes of S288C reference strain and of two wine yeast isolates, the RM11-1A and EC1118 strains, exhibit *ARN4* homologs.

Lineage 10 comprises the homologs of *S. cerevisiae ARN3* gene (cluster P). The chromosome environment where cluster P-encoding genes reside is sparsely conserved, splitting lineage 10 into five different sublineages (Figure 8A). One sublineage spans the species belonging to the CTG, although *C. albicans*, *C. dubliniensis* and *L. elongisporus* lack cluster P-encoding genes. Regarding species belonging to the *Saccharomyces* complex, two sublineages encompass genes from post-WGD species while the remaining two encompass genes from pre-WGD species. The origin of the sublineage containing the *S. cerevisiae ARN3* gene can be traced back to klpo_358.4. The amino acid sequence of pipa_3g03640 is similar to sakl0g06600 protein, although it does not share common neighbours with the remaining cluster P members.

Lineage 11 comprises the homologs of *CaARN1* gene (cluster R). These genes are not related to the *S. cerevisiae ARN1* gene since they do not share common neighbours and do not group in the same phylogenetic cluster. Although this lineage spans mainly genes of CTG diploid species, the haploid species *P. stipitis* also possesses one cluster R ORF (Figure 7D).

Lineage 13 comprises the homologs of *S. cerevisiae ARN1*/*ARN2* genes (cluster T). Four sublineages exist within lineage 13 (Figure 8B), all spanning genes of species belonging to the *Saccharomyces* complex*.* The origin of the sublineage containing the *S. cerevisiae ARN1* gene could be retraced to a *Z. rouxii* ORF (zyro0g07414). The *K. polysporus* genome lacks a cluster T-encoding gene suggesting that, after the WGD event, the ancestral of the post-WGD species quickly lost one gene duplicate. The genomes of Lachancea species and of *K. lactis* show an abundant number of cluster T-encoding genes, sparsely syntenic. Although the species of the CTG complex lack full-size cluster T-encoding genes, the genome of *C. guilliermondii* show a gene fragment whose amino acid sequence is highly similar to these transporters. The genomes of the early-divergent hemiascomycetes *P. pastoris* and *Y. lipolytica* lack cluster T-encoding genes.

The phylogenetic cluster M comprises proteins showing sequence similarity to siderophore transporters (Figure 2). As described by Diffels et al. [16], genes encoding cluster M transporters are only present in *Y. lipolytica* genome and reside in the phylogenetic cluster 2.A.1.16.Z2. Pairwise similarity searches using cluster M amino acid sequences against the Aspergillus Genome Database showed that these proteins share high sequence similarity with *Aspergillus nidulans* MirC (e-value 4E-76), MirA (e-value 5E-58) and MirB (e-value 5E-58) proteins, three biochemically characterized siderophore transporters [14,64,65].

**Figure 9:**
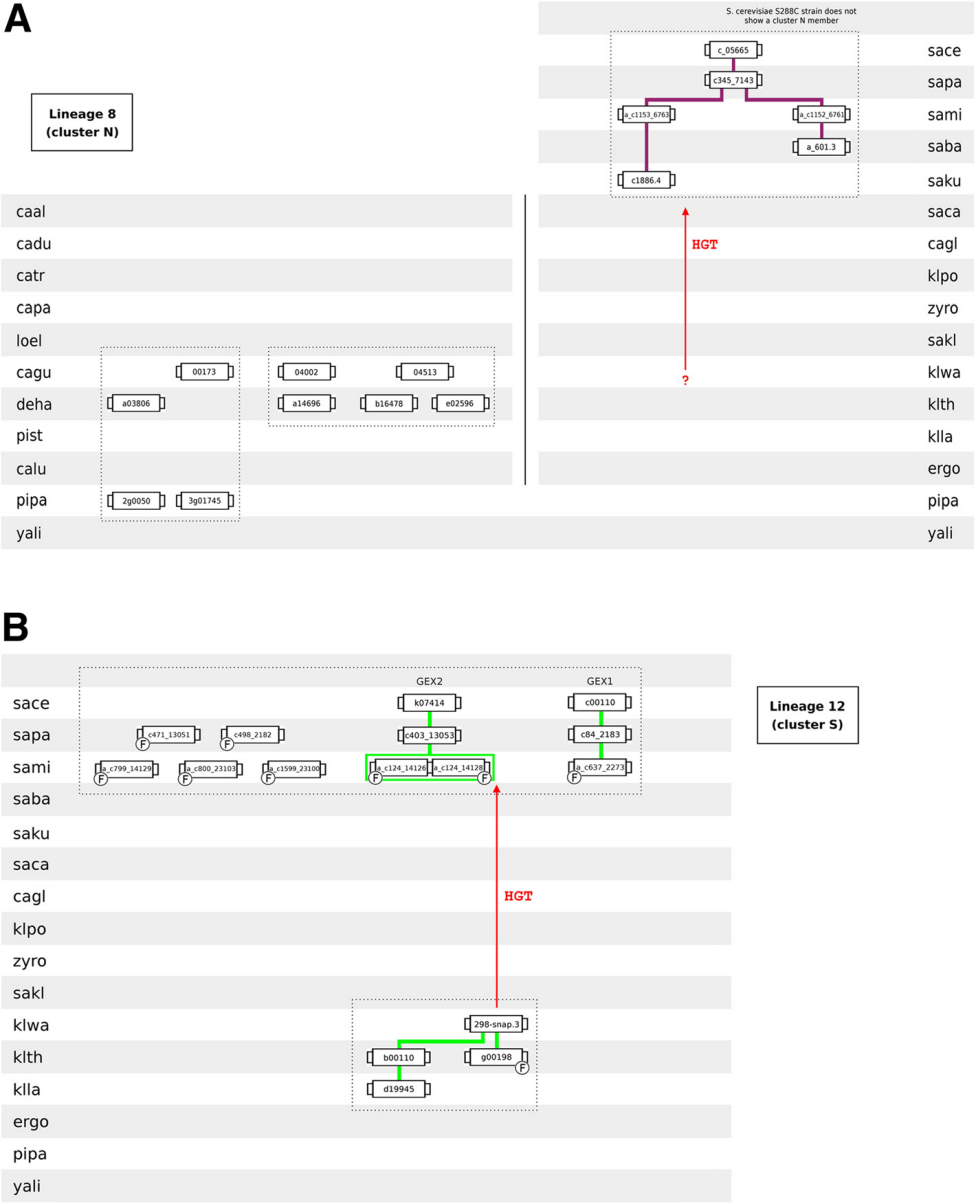
** Lineages 8 (cluster N proteins) and 12 (homologs of *S. cerevisiae GEX1/GEX2 *genes).** Conventions as in Figure 3.

The amino acid sequence of members of phylogenetic cluster N are closely related to those of siderophore transporters (Figure 2). Diffels et al. [16] reported that these proteins group in two different phylogenetic clusters (2.A.1.16.Z3 and 2.A.1.16.Z4). The gene neighbourhood analysis allowed the reconstruction of the evolutionary history of cluster N-encoding genes (lineage 8). All species belonging to the SSSG possess a cluster N transporter and the corresponding genes reside in a conserved chromosome environment (Figure 9A). However, with exception of the JAY-291 strain, all *S. cerevisiae* strains considered in this work lack a cluster N-encoding gene. Cluster N members are also found in species belonging to the CTG haploid sub-group and in the early-divergent hemiascomycetes *P. pastoris*.

### GEX gene lineages

Lineage 12 comprises the homologs of *S. cerevisiae GEX1*/*GEX2* genes (cluster S). The members of the GEX gene lineage reside in the phylogenetic cluster 2.A.1.16.Z1 (as described in [16]). Several previous studies indicated that the amino acid sequences of Gex1p and Gex2p are highly similar to those of Arn1 and Arn2 proteins [15,16,18] and the analysis of the phylogenetic tree representing the 14-spanner DHA2, ARN and GEX sub-families confirmed this observation (Figure 2). The close resemblance between the members of cluster T (Arn1/Arn2 homologs) and cluster S (Gex1/Gex2 homologs) suggests that these two functionally distinct groups of 14-spanner MFS transporters have been differentiated from the same ancestral gene. Interestingly, only the genomes of three species belonging to the SSSG and of three pre-WGD species were found to encode cluster S-encoding genes in the Hemiascomycetes clade (Figure 9B). This lineage divides into three sublineages, two containing each *S. cerevisiae* GEX gene while the third one comprises the GEX homologs present in the genomes of *K. lactis*, *K. thermotolerans* and *K. waltii*. The discontinuity occurring in lineage 12 in the transition from pre- to post-WGD species suggests that the cluster S-encoding genes were acquired by the ancestral of the SSSG by lateral gene transfer, presumably from a pre-WGD species.

## Discussion

A combined approach using classical phylogenetic tree building methods and gene neighbourhood analysis was used to reconstruct the evolution of the DHA2 genes in the Hemiascomycetes. This study considered twenty additional hemiascomycetous species to those examined by Gbelska *et al.*[41], which did not included gene neighbourhood analysis. The 12 cluster classification of the DHA2 subfamily proposed in our phylogenetic study considerably expands the previous study [41]. Members of the phylogenetic clusters B (Sge1/Azr1/Vba3/Vba5), C (Vba1/Vba2), D (Vba4), E (Atr1/YMR279C) and F (YOR378W) were found in the majority of the hemiascomycetous species analysed in this study, strongly suggesting that these DHA2 proteins may sustain important biological functions.

The comparative genomics approach adopted in this work allowed understanding the evolutionary relationships between three *S. cerevisiae* DHA2 genes encoding related amino acid sequences: *ATR1* and the uncharacterized ORFs YMR279C and YOR378W. This approach revealed that the *ATR1* and YMR279C genes are ohnolog genes (lineage 4) while YOR378W resides in its own lineage (lineage 5). Although the genes comprised in these two lineages do not share common neighbours, this does not exclude the existence of a common evolutionary origin rooting deep in the Fungi phylogenetic tree. The close phylogenetic relationship of ORF YMR279C with the *ATR1* gene and the fact that its constitutive expression confers resistance to boron in yeast cells through the decrease of the intracellular levels of this element [24], is consistent with the hypothesis that ORF YMR279C is a boron extrusion pump acting as a back-up of the Atr1p transporter [24]. The proteins belonging to cluster E present in the genomes of the pre-WGD yeast species are more similar to Atr1p than to the ORF YMR279C encoded protein. This may suggest that the ancestral function carried out by these two genes was preserved by Atr1p while the putative ORF YMR279C boron back-up function is, presumably, a new metabolic feature acquired through functional divergence of one of the redundant gene copies produced at the WGD event.

Although a few DHA2 family transporters have been described as recognizing substrates of biological significance, most of those known to be required for resistance to several drugs and other xenobiotic compounds do not have an assigned biological role yet [19]. Remarkably, the expression of a number of DHA2 transporters has been found to confer increased susceptibility, rather than resistance, to specific chemical compounds as well. This is the case of the *VBA5* gene whose overexpression in *S. cerevisiae* sensitizes the cells to the action of 4-NQO and quinidine [29] or of the ORF YOR378W whose overexpression leads to increased yeast susceptibility to rapamycin [25]. These observations reinforces the idea that the drug pump model used to explain the physiological functions associated to the Major Facilitator Superfamily Multidrug Resistance (MFS-MDR) transporters is too simplistic [19].

The phylogenetic study described here strongly suggests that the previous cluster classification of the ARN and GEX members [16] should be revised. Since the time of the previous phylogenetic study, the *GEX1* and *GEX2* genes were shown to encode glutathione exchangers, a biological role that makes them physiologically apart from the ARN proteins. The functional classification of the ARN and GEX proteins into distinct subfamilies cannot ignore the fact that the encoding genes share highly related amino acid sequences (Figure 2) and that their expression is activated under conditions of iron depletion, although GEX gene expression only occurs under extreme iron scarcity [17]. The main transcription regulator of the expression of the four ARN genes is Aft1p [17] while the expression of *GEX1* gene is under control of the transcription factor Aft2p [18]. The *S. cerevisiae AFT1* and *AFT2* genes are paralogs [66] that specialized during yeast evolution to perform overlapping but not redundant functions [67]. The close amino acid sequence similarity between the ARN and GEX proteins suggests that the encoding genes may share a common evolutionary origin and that posterior divergence led to their differentiation, both in sequence and regulation, to fulfill different physiological functions.

Consistent with the notion that siderophore uptake is strongly dependent on the genetic background of the yeast strain [68], the present study also uncovered important variations in the arsenal of siderophore transporters encoded in genomes of different *S. cerevisiae* strains. While S288C and EC1118 strains do possess both *ARN1* and *ARN2* genes, the genomes of the remaining *S. cerevisiae* strains examined in this study only have the *ARN1* gene suggesting that the former strains may have acquired the *ARN2* gene by lateral gene transfer, presumably from a pre-WGD donor species. The genomes of *S. cerevisiae* strains JAY291 and YJM789 also lack an *ARN4* homolog. Interestingly, *ARN4* homologs only exist in SSSG species, all residing in sub-telomeric regions. These chromosomal regions are thought to serve as nursery for new genes and to provide a reservoir where new haplotypes and new gene functions can be created [69]. However, the inspection of the chromosome neighbourhood where each *ARN4* homolog resides did not provide any clue regarding the origin of this hypothetical primordial gene.

The synteny and similarity data suggests that lateral gene transfer and gene duplication were the main evolutionary forces responsible for the expansion of genes encoding DHA2, ARN and GEX transporters in the Hemiascomycetes. Lateral gene transfer is proposed to have occur in lineage 1 (*SGE1* and *VBA3*/*VBA5* homologs), lineage 2 (*VBA2* homologs), lineage 5 (YOR378W homologs), lineage 6 (*KNQ1* homologs), lineage 8 (cluster N members), lineage 12 (*GEX1*/*GEX2* homologs) and lineage 13 (*ARN2* homologs). Gene duplication, the primary source of new genes necessary for the evolution of functional novelty [70], was found to be a frequent event in the majority of DHA2, ARN and GEX gene lineages reconstructed in this study. The consistent evolutionary pattern of a surplus of duplicate genes belonging to the same phylogenetic cluster found to occur in certain hemiascomycetous genomes raises the question of how many of these genes are still functionally redundant and how many have already been co-opted through neofunctionalization or sub-functionalization to fulfil new physiological functions in the corresponding yeast strains. Although the majority of the genes encoding DHA2, ARN and GEX transporters are not essential in laboratorial optimal conditions, the widespread occurrence of lateral transfer and duplication events of these genes during the evolution of the hemiascomycetes suggest that the encoded proteins may sustain important physiological functions in the diverse range of ecological niches occupied by these yeasts in nature. Considering that multidrug resistance and iron uptake are major determinants of yeast virulence, the identification of the complete set of DHA2 and ARN transporters present in the genomes of ten *Candida* pathogenic species provides potential new molecular targets for antifungal drug development.

The phylogenetic results emerging from this work together with new experimental results recently reported in the literature concerning the DHA2, ARN and GEX transporters should be considered for the eventual revision of protein family classification used in TCDB database [7]. In specific, the biochemically characterized *S. cerevisiae* Gex1, Gex2, Vba5, *C. albicans Ca*Arn1 and *K. lactis* Knp1 transporters with a demonstrated function in yeast physiology should be included in TCDB database. Moreover, the finding that ORF YMR279C is the paralog of *ATR1* gene with origin in the WGD event and that it is also involved in boron homeostasis suggest its classification in the same TCDB family of ATr1p.

This study provides evidence for a close amino acid sequence similarity between the DHA2, ARN and GEX proteins. The fact that these 14-spanner transporters of the Major Facilitator Superfamily are hypothesized to recognize distinct substrates and may have different subcellular localization, at the plasma membrane, the vacuole membrane, post-Golgi vesicles and late endosomal vesicles [14,18,19,71], suggests that it is unlikely that their sequence similarity may result from convergent evolution. The hypothesis that DHA2, ARN and GEX transporters share a common evolutionary root is the explanation that better fits the results of this phylogenetic study and we propose a new family to accommodate the DHA2, ARN and GEX proteins, DAG, spanning these three phylogenetic subfamilies of 14-spanner MFS transporters. This hypothesis is corroborated by the fact that these three subfamilies appeared during the evolutionary transition giving birth to the Dikarya fungi (unpublished results). Subsequently, selection, radiation and neofunctionalization of the initial ancestral genes encoding these 14-spanner MFS transporters gave rise to the functions associated with them, spanning the MDR phenomenon, amino acid transport, boron homeostasis, siderophore transport and glutathione exchange.

## Conclusions

A total of 172,422 translated ORFs encoded in the genomes of 31 sequenced yeast strains from 25 hemiascomycetous species were gathered in this study. The corresponding amino acid sequences were compared using the blastp algorithm, generating a total of 31 million pairwise alignments, represented as a network. A functionally characterized DHA2 protein, Atr1p, was used as starting node to breadth-first traverse this network at different e-value thresholds. 14-spanner Major Facilitator Superfamily transporters involved in siderophore import [14] and glutathione export [18] were gathered together with the DHA2 proteins, supporting the concept that the genes encoding the DHA2, ARN and GEX proteins share a common evolutionary origin. The new protein family spanning these three phylogenetic subfamilies was denominated the DAG protein family and a phylogenetic tree representing the full-size DAG proteins was built. Gene neighbourhood analysis of the chromosome environment where the DHA2, ARN and GEX genes reside allowed the identification of seven DHA2 gene lineages, five ARN gene lineages and one GEX gene lineage. Lateral gene transfer and gene duplication were important mechanisms underlying the evolution of the DAG genes in the Hemiascomycetes.

### Availability of supporting data

The data sets supporting the results of this article are available in the TreeBASE Repository with a study Accession URL http://purl.org/phylo/treebase/phylows/study/TB2:S15039.

## Abbreviations

WGD: Whole genome duplication; Saccharomyces complex: *Saccharomyces sensu lato* group; CTG complex: Group of species that translate the CUG codon into serine instead of leucine; MDR: Multiple drug resistance; ABC: ATP-binding cassette; MFS: Major facilitator superfamily; TMS: Transmembrane span; DHA1: Drug:H^+^ antiporters of family 1; DHA2: Drug:H^+^ antiporters of family 2; UMF: Unknown major facilitator; ARN: Anhydromevalonyl residues linked to N^δ^-ornithine; SIT: Siderophore iron transport; GEX: Glutathione exchangers; 4-NQO: 4-nitroquinoline-1-oxide; TCDB: Transporter classification database; DAG: Group comprising the DHA2, ARN and GEX transporters; SSSG: *Saccharomyces sensu strictu* group; HGT: Horizontal gene transfer.

## Competing interests

The authors declare that they have no competing interests.

## Authors’ contributions

PJD carried out phylogenetic tree construction and gene neighbourhood analysis and built the pairwise similarity network. ISC conceived and supervised this study and together with PJD wrote the manuscript. All authors read and approved the final manuscript.

## Supplementary Material

Additional file 1 **Potential 14-spanner MFS-MDR proteins gathered from the 31 hemiascomycetous yeasts analysed during this work.** This table shows the gene/ORF name, acronym, protein sequence, protein sequence length and phylogenetic cluster classification. It also indicates whether the translated ORF was considered to comprise a true 14-spanner MFS-MDR protein and if the corresponding amino acid sequence was used in the construction of the phylogenetic tree. Both HMMTOP 2.1 and TMHMM 2.0 were used for topology prediction of the amino acid sequences under analysis (HMMTOP_pred indicates number of predicted TMS, HMMTOP_N_top indicates the N-terminal topology prediction, TMHMM_pred indicates number of predicted TMS, TMHMM_topology details where topology changes along the protein sequence, TMHMM_First60 indicates the expected number of amino acids in transmembrane helices in the first 60 amino acids of the protein). For amino acid sequences showing less than 490 amino acids in length, this table also indicates whether the protein sequence was gathered or not at blastp e-values of E-15, E-17, E-20, E-25 and E-30 (Y = yes, N = no) and the TMS range predicted by visual analysis of the topology probability and protein hydrophobicity plots generated by TMHMM 2.0 and TOPPRED 2, respectively, for each amino acid sequence. Click here for file

Additional file 2 **Radial phylogram representing the 920 amino acid sequences gathered at an e-value level of E-14 as described in Figure **1**.** Besides the 14-spanner MFS-MDR transporters, 508 DHA1 amino acid sequences and 10 non-membrane proteins were recovered at this similarity threshold. Click here for file

Additional file 3 **Radial phylogram showing the amino acid sequence similarity distances between the 355 full-size 14-spanner MFS transporters.** Protein and translated ORF names are shown. The name of the *S. cerevisiae* and *C. albicans* members is indicated as well as the biochemically characterized Knq1 transporter of *K. lactis*. The gene and species annotation adopted in this study uses the four letters code described in Table 1. Click here for file

Additional file 4 Radial phylogram of the DHA2, ARN and GEX transporters gathered from 31 hemiascomycetous yeasts using the PROML package of PHYLIP suite. Click here for file

Additional file 5 Circular cladogram of the DHA2, ARN and GEX transporters gathered from 31 hemiascomycetous yeasts using the PROML package of PHYLIP suite. Click here for file

Additional file 6 **Homology relationships established between the *S. cerevisiae *** **DHA2, ARN and GEX genes and genes present in the genomes of the most virulent *Candida* species.** Click here for file

Additional file 7 **Homology relationships established between the *S. cerevisiae *** **DHA2, ARN and GEX genes and genes present in the genomes of the less virulent *Candida *** **species.** Click here for file

Additional file 8 **Chromosome environment of the DHA2, ARN and GEX genes gathered from thirty-one hemiascomycetous yeasts.** Gene neighbourhood is shown with a 30-gene window. The following genomic information is displayed in two tables: a) gene name and b) protein family name. Each box framed in red represents a gene. Adjacent boxes represent the gene neighbours. Yellow background represents genes not belonging to the phylogenetic cluster associated to the lineage. Homologous neighbours, based on our protein family classification, are highlighted in the same colour. Click here for file

Additional file 9 **Multiple alignment of the amino acid sequences encoded by the full size homologs of the *S. cerevisiae VBA3***/***VBA5 *** **genes and by the fragments sami_a_c798_9285 and saku_c1383.2.** Click here for file

Additional file 10 **Analysis of the DNA upstream regions of *S. cerevisiae VBA3 *** **gene and ORF sace_e_3474.** Click here for file
