# Supplementary figures and images for: The drug:H+ antiporters of family 2 (DHA2), siderophore transporters (ARN) and glutathione:H+ antiporters (GEX) have a common evolutionary origin in hemiascomycete yeasts
Source: BMC Genomics. 2013 Dec 18;14:901. doi: 10.1186/1471-2164-14-901 (PMC3890622; doi:10.1186/1471-2164-14-901)

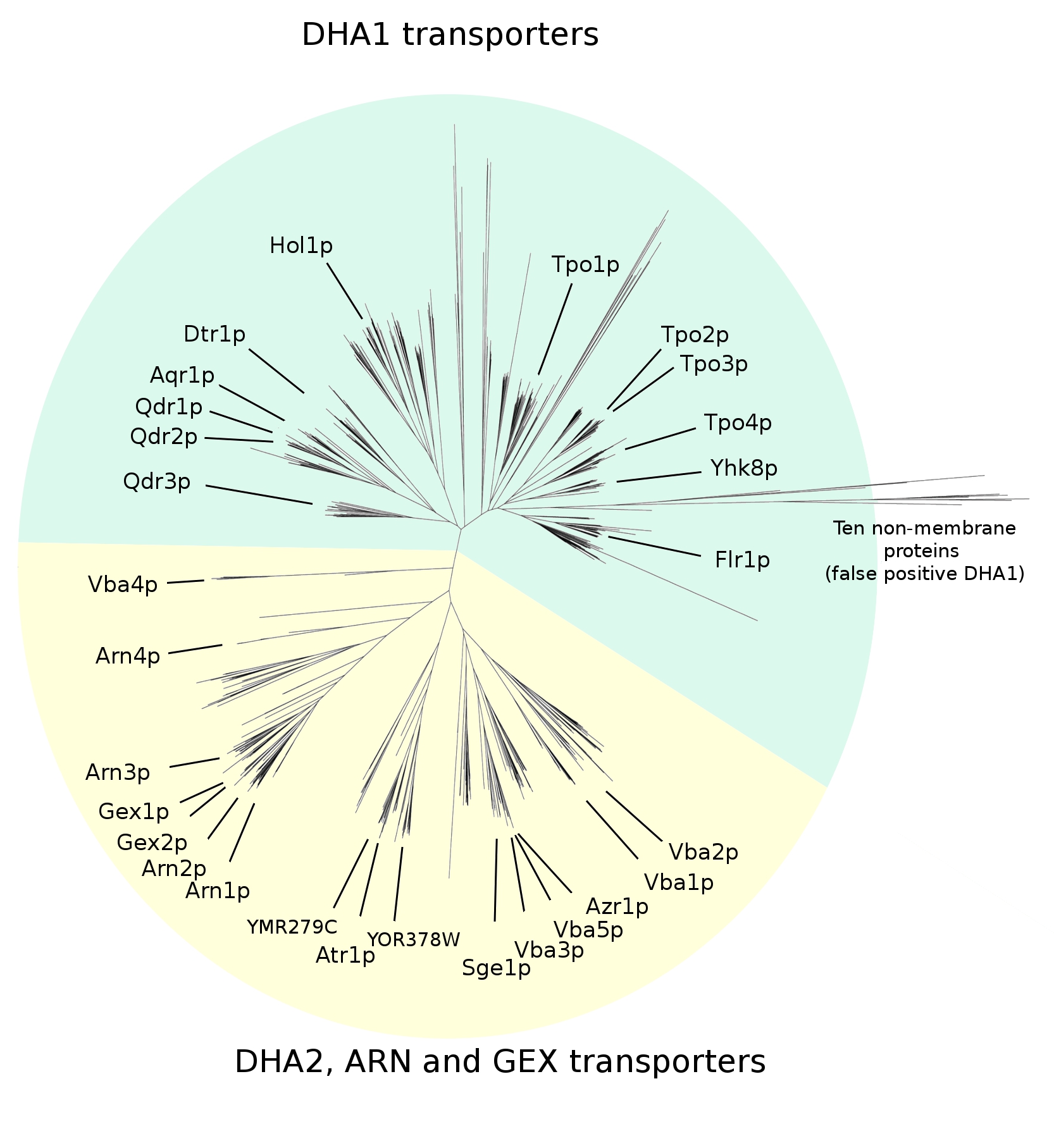

Supplement: Additional file 2 — Radial phylogram representing the 920 amino acid sequences gathered at an e-value level of E-14 as described in Figure 1. Besides the 14-spanner MFS-MDR transporters, 508 DHA1 amino acid sequences and 10 non-membrane proteins were recovered at this similarity threshold. [file 1471-2164-14-901-S2.jpeg]

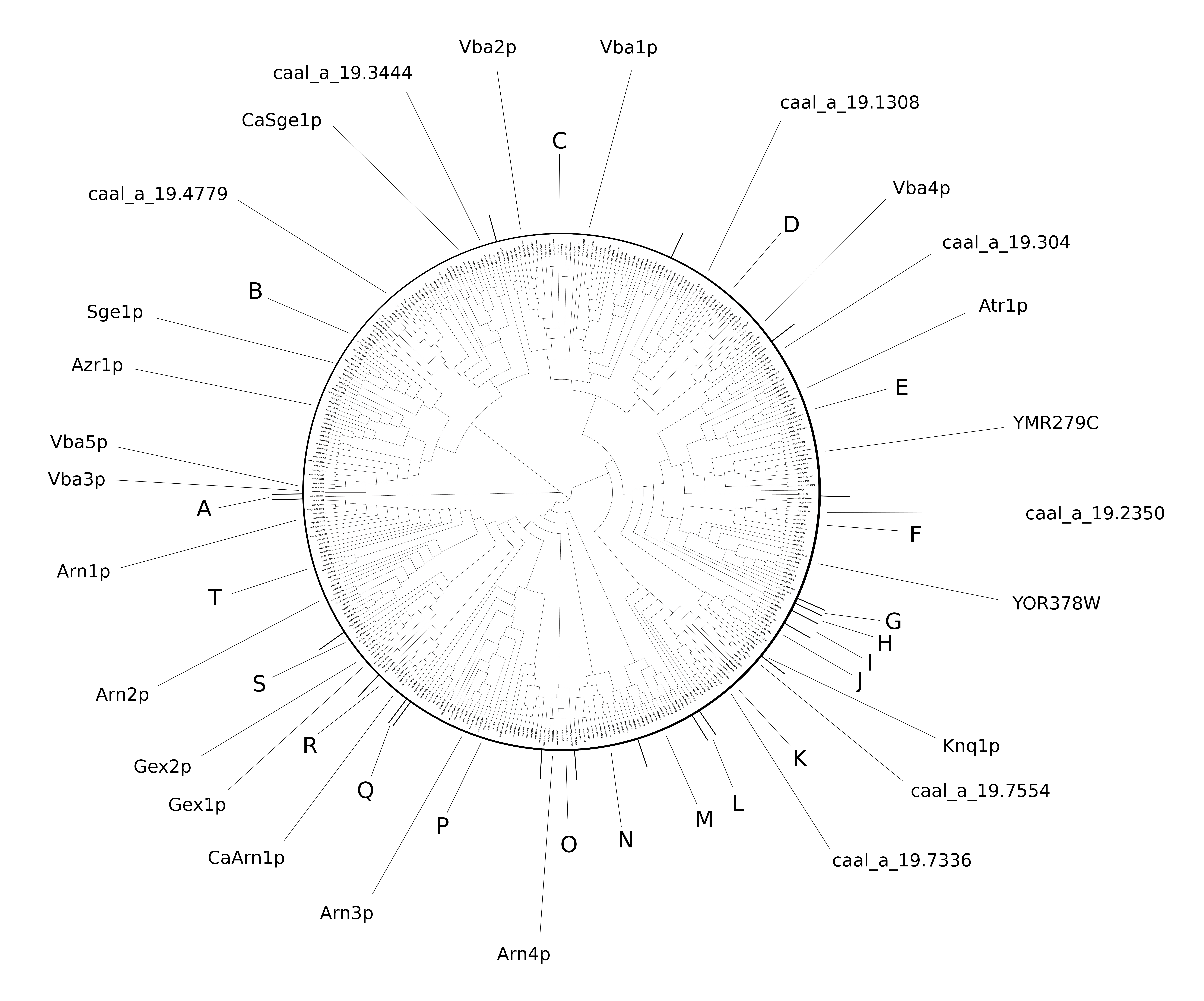

Supplement: Additional file 3 — Radial phylogram showing the amino acid sequence similarity distances between the 355 full-size 14-spanner MFS transporters. Protein and translated ORF names are shown. The name of the S. cerevisiae and C. albicans members is indicated as well as the biochemically characterized Knq1 transporter of K. lactis. The gene and species annotation adopted in this study uses the four letters code described in Table 1. [file 1471-2164-14-901-S3.jpeg]

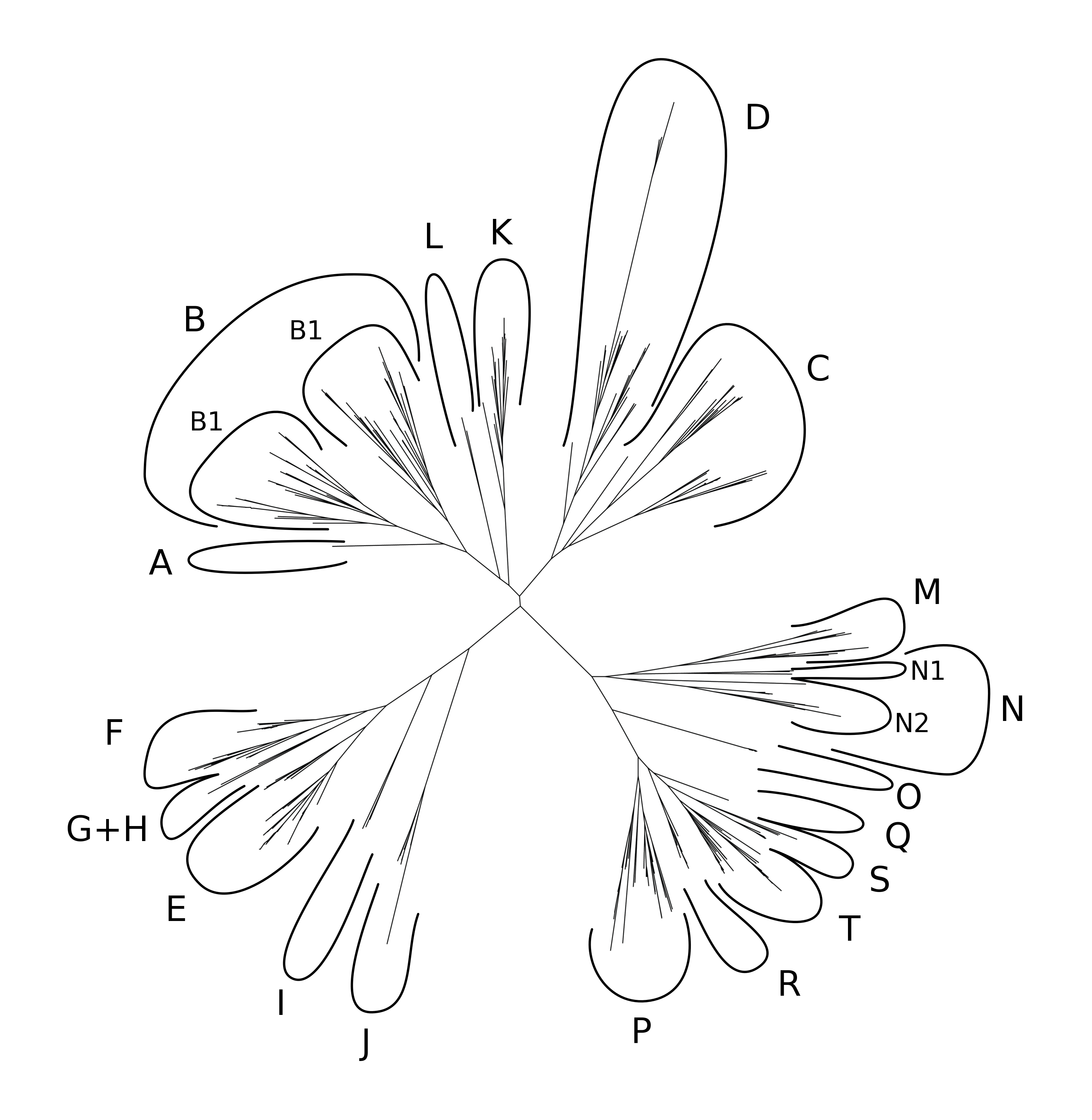

Supplement: Additional file 4 — Radial phylogram of the DHA2, ARN and GEX transporters gathered from 31 hemiascomycetous yeasts using the PROML package of PHYLIP suite. [file 1471-2164-14-901-S4.jpeg]

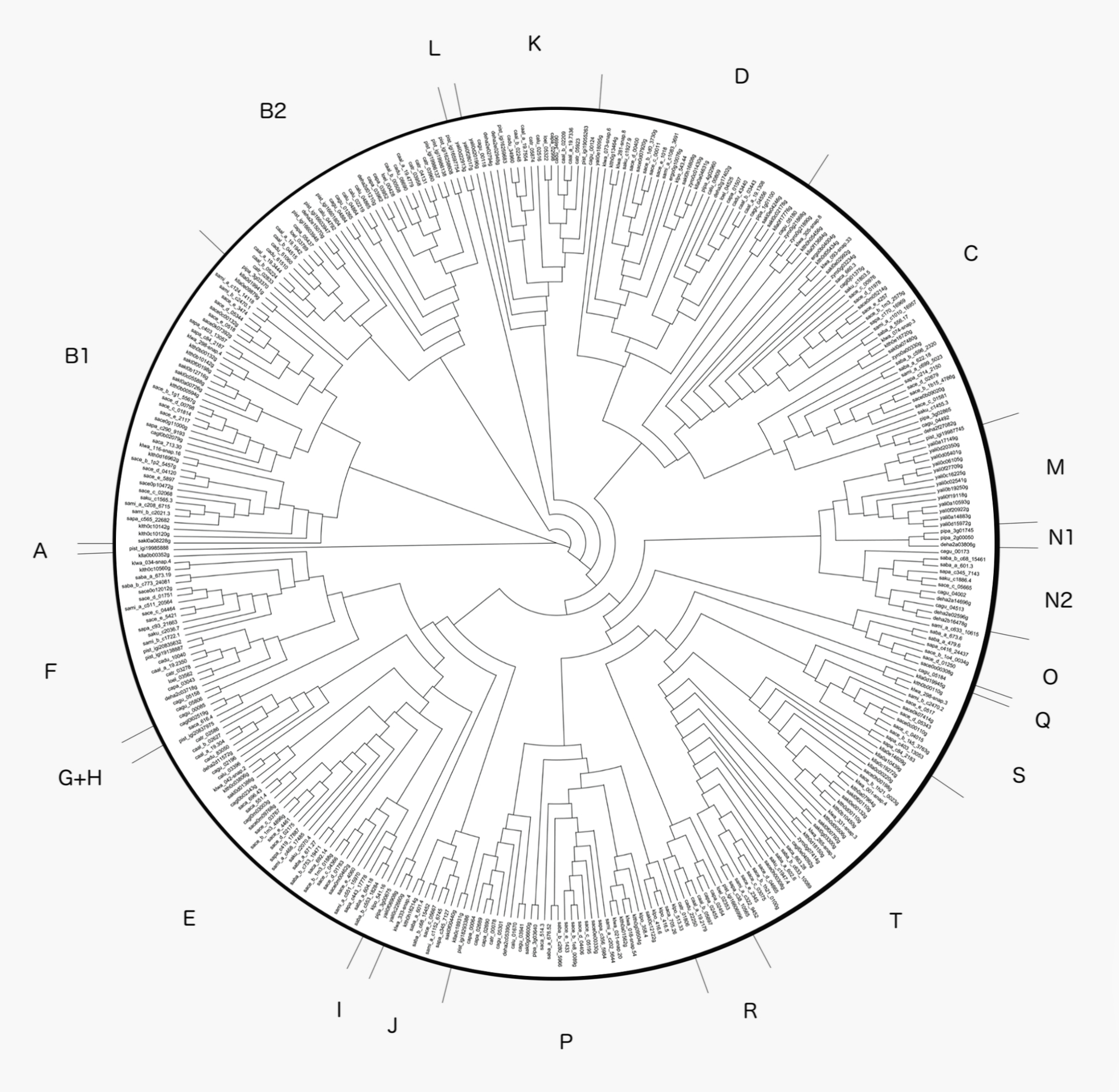

Supplement: Additional file 5 — Circular cladogram of the DHA2, ARN and GEX transporters gathered from 31 hemiascomycetous yeasts using the PROML package of PHYLIP suite. [file 1471-2164-14-901-S5.jpeg]
