# Supplementary material for: The drug:H+ antiporters of family 2 (DHA2), siderophore transporters (ARN) and glutathione:H+ antiporters (GEX) have a common evolutionary origin in hemiascomycete yeasts
Source: BMC Genomics. 2013 Dec 18;14:901. doi: 10.1186/1471-2164-14-901 (PMC3890622; doi:10.1186/1471-2164-14-901)
Supplement: Additional file 6 — Homology relationships established between the S. cerevisiae DHA2, ARN and GEX genes and genes present in the genomes of the most virulent Candida species. [file 1471-2164-14-901-S6.pdf]

| Subfamily | Cluster | S. cerevisiae ( S288C ) | C. glabrata ( CBS138 ) | C. albicans ( SC5314 ) | C. dubliniensis ( CD36 ) | C. tropicalis ( MYA-3404 ) | C. parapsilosis ( CDC 317 ) |
|---|---|---|---|---|---|---|---|
| DHA2 | B | SGE1<br>AZR1<br>VBA3<br>VBA5 | cagl0b02079 | caal_a_19.1942<br>caal_a_19.3444<br>caal_a_19.4779 | cadu_51060<br>cadu_61510<br>cadu08690 | catr_02833<br>catr_04131<br>catr_03958<br>catr_03960 | capa_05437<br>capa_03962<br>capa_03952 |
| | C | VBA1<br>VBA2 | cagl0j01375 | - | - | - | - |
| | D | VBA4 | - | caal_a_19.1308 | cadu_43440 | - | capa_01507 |
| | E | ATR1<br>YMR279C | cagl0b02343<br>cagl0m03003 | caal_a_19.304 | cadu_83050 | catr_02586 | - |
| | F | YOR378W | - | caal_a_19.2350 | cadu_10040 | catr_03278 | capa_03043 |
| | K | - | - | caal_a_19.7554<br>caal_a_19.7336 | cadu_34960<br>cadu_34690 | catr_05874<br>catr_05923 | capa_02923 |
| ARN | O | ARN4 | - | - | - | - | - |
| | P | ARN3 | - | - | - | catr_00078 | capa_00064<br>capa_02689<br>capa_02690 |
| | R | - | - | CaARN1 | cadu_22250 | catr_01806 | capa_02454<br>capa_02455 |
| | T | ARN1<br>ARN2 | cagl0e04092 | - | - | - | - |
| GEX | S | GEX1<br>GEX2 | - | - | - | - | - |
