# Supplementary material for: The drug:H+ antiporters of family 2 (DHA2), siderophore transporters (ARN) and glutathione:H+ antiporters (GEX) have a common evolutionary origin in hemiascomycete yeasts
Source: BMC Genomics. 2013 Dec 18;14:901. doi: 10.1186/1471-2164-14-901 (PMC3890622; doi:10.1186/1471-2164-14-901)
Supplement: Additional file 7 — Homology relationships established between the S. cerevisiae DHA2, ARN and GEX genes and genes present in the genomes of the less virulent Candida species. [file 1471-2164-14-901-S7.pdf]

Subfamily	Cluster	S. cerevisiae (S288C)	L. elongisporus (NRLL YB-4239)	C. guilliermondii (ATCC 6260)	D. hansenii (CBS767)	C. lusitaniae (ATCC 42720)	Y. lipolytica (CLIB122)
	B	SGE1<br>AZR1<br>VBA3<br>VBA5	loel_00428<br>loel_03789	cagu_01265<br>cagu_04884	deha2d01210g<br>deha2e15070g	calu_02219<br>calu_04865<br>calu_04864<br>calu_04792	-
	C	VBA1<br>VBA2	-	cagu_04492<br>cagu_05180	deha2f27082g	-	-
	D	VBA4	loel_04525	cagu_04556	deha2g17402g	calu_00809	yali0e18095g
	E	ATR1<br>YMR279C	-	cagu_02196	deha2d11572g	calu_03396	-
DHA2	F	YOR378W	loel_03562	cagu_05158<br>cagu_05806	deha2c03718g	-	-
	G	-	-	cagu_00085	-	-	-
	I	-	-	-	-	-	yali0f06908g<br>yali0c22660g
	K	-	loel_05322	cagu_00118<br>cagu_00124	deha2e02948g<br>deha2e02794g	calu_02516	yali0d20196g
	L	-	-	-	-	-	yali0f28017g<br>yali0d22913g
	M	-	-	-	-	-	yali0d15972g<br>yali0a14883g<br>yali0b19250g<br>yali0c02541g<br>yali0f19118g<br>yali0f20922g<br>yali0a10593g<br>yali0c16225g<br>yali0f27709g<br>yali0c06105g<br>yali0d05401g<br>yali0d20350g<br>yali0a17149g
ARN	N	-	-	cagu_00173<br>cagu_04513<br>cagu_04002	deha2a03806g<br>deha2b16478g<br>deha2e02596g<br>deha2a14696g	-	-
	O	ARN4	-	-	-	-	-
	P	ARN3	-	cagu_05301<br>cagu_03941	deha2c05390g	calu_01670	-
	Q	-	-	cagu_05184	-	-	-
	R	-	loel_02353	-	-	-	-
	T	ARN1<br>ARN2	-	-	-	-	-
GEX	S	GEX1<br>GEX2	-	-	-	-	-
