# Supplementary material for: The drug:H+ antiporters of family 2 (DHA2), siderophore transporters (ARN) and glutathione:H+ antiporters (GEX) have a common evolutionary origin in hemiascomycete yeasts
Source: BMC Genomics. 2013 Dec 18;14:901. doi: 10.1186/1471-2164-14-901 (PMC3890622; doi:10.1186/1471-2164-14-901)
Supplement: Additional file 8 — Chromosome environment of the DHA2, ARN and GEX genes gathered from thirty-one hemiascomycetous yeasts. Gene neighbourhood is shown with a 30-gene window. The following genomic information is displayed in two tables: a) gene name and b) protein family name. Each box framed in red represents a gene. Adjacent boxes represent the gene neighbours. Yellow background represents genes not belonging to the phylogenetic cluster associated to the lineage. Homologous neighbours, based on our protein family classification, are highlighted in the same colour. [file 1471-2164-14-901-S8.pdf]

Cluster A

pist	4266	4264	3356	1476	3352	5811	3351	3348	236	3349	3350	5862	1804	1539	9117	pist_igi19985888	1919	0	0	0	0	0	0	0	0	0	0	0	0	0	0
pist	4196	4197	3883	3884	4269	4268	2570	4266	4264	3356	1476	3352	5811	3351	3348	pist_igi16597754	3349	3350	5862	1804	1539	9117	236	1919	0	0	0	0	0	0	0

Homologs of ORF pist_igi19985888. The protein family classification of the adjacent genes is shown and the homologous neighbours are highlighted in the same colour.

pist	pist_igi16597747	pist_igi16597748	pist_igi16597749	pist_igi19139403	pist_igi16597750	pist_igi16597751	pist_igi16597752	pist_igi16597753	pist_igi16597754	pist_igi19139404
pist	pist_igi16597744	pist_igi20834536	pist_igi18291041	pist_igi16597745	pist_igi18291042	pist_igi16597746	pist_igi19139402	pist_igi16597747	pist_igi16597748	pist_igi16597749

pist	pist_igi18291043	pist_igi20834537	pist_igi19139405	pist_igi20834538	pist_igi16597755	pist_igi19985888	pist_igi18291044	Null	Null	Null
pist	pist_igi19139403	pist_igi16597750	pist_igi16597751	pist_igi16597752	pist_igi16597753	pist_igi16597754	pist_igi19139404	pist_igi18291043	pist_igi20834537	pist_igi19139405

pist	Null	Null	Null	Null	Null	Null	Null	Null	Null	Null	Null
pist	pist_igi20834538	pist_igi16597755	pist_igi19985888	pist_igi18291044	Null	Null	Null	Null	Null	Null	Null

Homologs of ORF pist_igi19985888. The names of the adjacent genes are shown and the homologous neighbours are highlighted in the same colour.

Cluster B (lineage 1): homologs of SGE1, AZR1, VBA3 and VBA5 genes

Sheet1

sace_a	0	0	0	0	0	0	0	0	0	0	0	0	0	0	896	sace0c00132g	662	4998	15495	764	4999	3894	3918	15744	15494	146	15493	2025	4003	5003	5004
sace_a	3431	4541	2790	1714	5875	5874	4156	5026	4158	1712	4464	4466	5025	5024	626	sace0g11000g	164	4155	2942	15596	1906	3507	2151	2007	5360	15597	5362	3603	15598	2179	16670
sace_a	15395	1381	1187	1549	2670	16693	2672	4486	607	2069	6057	15388	75	57	57	sace0k07392g	896	0	0	0	0	0	0	0	0	0	0	0	0	0	0
sace_a	192	15601	5492	4435	1373	2285	1228	5493	2792	1372	1229	1374	3732	601	4369	sace0p10472g	16736	16737	1572	11717	11717	11717	0	0	0	0	0	0	0	0	0
sace_b	3431	4541	2790	1714	5874	4156	5026	4158	1712	4464	16939	4466	5025	5024	626	sace_b_1g1_5567g	164	16940	4155	23149	2942	15596	1906	3507	2151	2007	5360	15597	5362	15598	2179
sace_b	5492	4435	1373	2285	1228	5493	2792	1372	1229	1374	3732	601	17212	4369	17213	sace_b_1p2_5457g	16736	16737	1572	11717	0	0	0	0	0	0	0	0	0	0	0
sace_c	0	0	0	0	0	0	0	0	0	0	4464	4466	5025	5024	626	sace_c_01814	164	4155	2942	15596	1906	3507	2151	2007	5360	15597	5362	3603	15598	2179	16670
sace_c	4382	1363	192	15601	5492	4435	1373	2285	5493	2792	1372	1229	1374	3732	4369	sace_c_02068	16736	16737	1572	0	0	0	0	0	0	0	0	0	0	0	0
sace_d	3920	3431	4541	2790	1714	5875	5874	4156	5026	4158	4464	4466	5025	5024	626	sace_d_00798	164	4155	2942	15596	1906	3507	2151	2007	5360	15597	5362	3603	15598	2179	16670
sace_d	402	1858	15395	1381	1187	1549	2670	16693	2672	4486	607	2069	6057	15388	4369	sace_d_04120	16736	16737	1572	0	0	0	0	0	0	0	0	0	0	0	0
sace_d	0	0	0	0	0	0	0	0	0	0	0	0	0	1040	896	sace_d_05344	662	4998	15495	764	4999	3894	3918	15744	15494	146	15493	2025	4003	5003	5004
sace_e	0	0	0	0	0	0	0	0	0	0	0	0	0	0	896	sace_e_0518	662	4998	0	0	0	0	0	0	0	0	0	0	0	0	0
sace_e	3431	4541	2790	1714	5875	5874	4156	5026	4158	1712	4464	4466	5025	5024	626	sace_e_2117	164	4155	2942	15596	1906	3507	2151	2007	5360	15597	5362	3603	15598	2179	16670
sace_e	1858	15395	1381	1187	1549	2670	16693	2672	4486	607	2069	6057	15388	75	57	sace_e_3474	0	0	0	0	0	0	0	0	0	0	0	0	0	0	0
sace_e	192	15601	5492	4435	1373	2285	1228	5493	2792	1372	1229	1374	3732	601	4369	sace_e_5897	16736	16737	1572	23742	0	0	0	0	0	0	0	0	0	0	0
sapa	1714	5875	5874	24522	5026	4158	1712	18026	4464	24523	4466	5025	5024	626	18027	sapa_c290_9193	164	164	18028	4155	24524	2942	15596	1906	3507	2151	24525	2007	5360	15597	5362
sapa	0	0	0	0	0	0	0	0	0	0	0	0	0	75	57	sapa_c403_13057	896	0	0	0	0	0	0	0	0	0	0	0	0	0	0
sapa	5492	4435	1373	2285	1228	5493	2792	1372	1229	1374	3732	601	4369	24734	17213	sapa_c565_22682	15657	1040	0	0	0	0	0	0	0	0	0	0	0	0	0
sapa	0	0	0	0	0	0	0	0	0	0	0	0	0	0	896	sapa_c84_2187	662	4998	16623	18044	764	4999	3894	3918	15494	146	15493	2025	4003	5003	5004
sami_a	0	0	0	0	0	0	0	0	0	0	0	0	75	15962	57	sami_a_c124_14118	896	896	0	0	0	0	0	0	0	0	0	0	0	0	0
sami_a	0	0	0	0	0	0	0	0	0	0	0	5510	1448	12787	24011	sami_a_c208_6715	24012	12787	0	0	0	0	0	0	0	0	0	0	0	0	0
sami_a	0	0	0	0	0	0	0	0	0	0	0	0	0	0	0	sami_a_c798_9285	164	24241	24240	0	0	0	0	0	0	0	0	0	0	0	0

sami_a	0	0	0	0	0	0	0	0	0	4464	4466	5025	5024	626	626	sami_a_c989_9288	0	0	0	0	0	0	0	0	0	0	0	0	0	0	0
sami_b	0	0	0	0	0	0	0	0	0	0	0	0	0	0	0	sami_b_c2470.1	896	0	0	0	0	0	0	0	0	0	0	0	0	0	0
saku	0	0	0	0	0	0	0	0	0	0	0	0	0	0	2191	saku_c1565.3	0	0	0	0	0	0	0	0	0	0	0	0	0	0	0
saca	5218	12123	2385	1730	1703	1671	3828	147	22846	6314	1303	2867	2674	1294	6313	saca_713.30	5709	722	716	1332	1619	35	525	1617	978	4354	5116	832	458	280	16511
cagl	5029	3256	3225	1023	1940	5028	5027	5026	4158	1712	4464	4466	5025	5024	626	cagl0b02079g	5023	3267	1663	1684	1557	5022	147	5021	1682	5020	5019	2567	3358	5018	5017
sakl	340	64	1883	63	1184	147	1918	5102	5206	17250	1267	5207	5208	1831	6077	sakl0a00726g	3609	4563	5104	5209	12166	2043	4533	4392	525	2004	2005	1084	1083	2058	11503
sakl	17283	17282	2369	2368	880	5947	2744	2745	4111	10810	6078	688	687	689	6077	sakl0a08228g	17281	5319	700	3217	525	3216	5489	5320	5321	3809	2463	2121	3908	3907	23744
sakl	17348	4699	747	5374	2560	4170	3496	468	1192	1579	3732	280	23748	5582	8514	sakl0b12716g	465	23749	17349	405	0	0	0	0	0	0	0	0	0	0	0
sakl	2542	4628	3352	147	1933	9783	6273	147	17375	4157	3063	10484	12438	915	915	sakl0c05588g	2472	242	915	4246	3799	992	4092	1044	1057	17376	17377	5680	6242	2282	10342
sakl	0	0	0	0	0	0	0	0	0	0	0	0	896	291	2042	sakl0f00198g	5582	1579	2682	465	17516	17517	4522	12627	11088	881	870	4996	1555	5583	12075
klwa	0	0	0	0	0	0	0	0	0	0	0	0	0	12337	12275	klwa_116-snap.16	82	2466	616	615	612	618	1125	1995	1995	2082	2961	57	10705	2637	2584
klwa	0	0	0	0	0	0	0	0	0	0	0	0	11717	12204	896	klwa_298-snap.4	12416	199	6068	1040	1040	1040	1040	1040	12755	6964	340	262	21	286	2914
klth	3521	468	912	5098	2737	57	11328	2987	468	2191	4088	12169	1192	134	82	klth0b00132g	896	0	0	0	0	0	0	0	0	0	0	0	0	0	0
klth	12169	4088	2191	468	2987	11328	57	2737	5098	912	468	3521	286	2914	340	klth0b00594g	12170	12171	12172	1328	316	12173	4238	2774	296	3062	41	1823	5267	2356	57
klth	0	0	6991	12218	896	11717	340	3888	11717	12217	12216	12215	12215	2611	9576	klth0b10142g	7616	6964	340	262	2466	616	615	612	618	1125	1995	1995	2082	2961	10704
klth	1793	1792	1790	4570	4569	790	1127	285	10678	1184	262	5359	462	5704	6077	klth0c10120g	236	2744	2369	2368	880	12268	4211	10808	10807	4210	21998	945	3072	4277	6816
klth	1792	1790	4570	4569	790	1127	285	10678	1184	262	5359	462	5704	6077	236	klth0c10142g	2744	2369	2368	880	12268	4211	10808	10807	4210	21998	945	3072	4277	6816	12269
klth	2066	1454	3128	1761	24	147	4699	747	5374	2560	4170	12343	75	75	1566	klth0d16962g	12342	442	443	5541	23	12341	12340	19	18	1491	12339	660	6160	1953	2016
klla	0	0	0	0	0	0	0	234	465	10828	10829	2191	468	11208	896	klla0d19941g	11207	2412	1566	2280	4469	2719	2722	2720	2721	264	2522	5826	12	5825	440
klla	11273	725	11272	1516	5690	657	11271	535	2143	2145	3702	3703	11270	10709	6816	klla0e09879g	11207	2919	2400	2917	220	11269	11268	314	75	280	364	2094	340	5744	248
caal_a	354	736	735	734	733	732	731	730	729	728	727	726	725	724	723	caal_a_19.1942	722	721	719	718	147	717	716	715	199	199	713	712	2290	20961	2291
caal_a	246	252	261	269	280	292	302	310	1829	1828	1827	1826	1825	1824	1823	caal_a_19.3444	187	187	167	167	1821	1820	1819	1818	20949	1817	187	1816	1815	1814	1813

Sheet1

caal_a	2726	2727	2728	2729	2730	2731	725	1398	2733	2734	2734	2735	2736	2737	2736	caal_a_19.4779	1643	662	2739	2440	2740	2741	2742	2743	2744	2746	2747	2748	2749	2750	2751
caal_b	2726	2727	2728	2729	2730	2731	725	1398	2733	2734	2734	2735	2736	2737	2736	caal_b_00508	1643	662	2739	2440	2740	2741	2742	2743	2744	2746	2747	2748	2749	2750	2751
caal_b	736	4791	734	733	732	731	730	730	729	728	727	726	725	724	723	caal_b_04515	722	721	719	718	147	4792	716	715	199	199	713	712	2290	4793	2291
caal_b	246	269	280	292	302	310	310	1829	1828	1827	1826	3551	1825	1824	1823	caal_b_05224	187	187	167	167	1821	1820	1819	1818	21108	1817	187	1816	1815	1814	1813
cadu	716	2726	2727	2728	2729	725	1398	2733	4849	2734	2734	2735	2736	2737	2736	cadu_08690	1643	662	2739	2440	2740	2741	2742	2743	2744	2746	2747	2748	2749	2750	2751
cadu	736	734	733	732	731	730	729	728	1040	1040	727	726	725	724	723	cadu_51060	722	721	719	718	147	716	715	199	199	713	712	2290	21182	2291	2292
cadu	238	239	246	269	280	292	302	310	1829	1828	1827	1826	1825	1824	1823	cadu_61510	187	187	167	167	1821	1820	1819	1818	1817	187	1816	1815	1814	1813	1812
catr	227	238	9114	246	269	280	292	302	310	310	1827	9115	1825	1824	1823	catr_02833	187	167	187	167	90	90	9116	1821	1820	1819	1817	150	1816	1815	1814
catr	2082	2083	2084	8697	9217	147	147	677	4435	2516	2515	314	490	1075	128	catr_03958	662	236	2731	9218	348	2520	82	3655	3656	3657	9219	525	174	173	171
catr	9220	170	171	173	174	525	9219	3657	3656	3655	82	2520	348	9218	2731	catr_03960	662	236	128	1075	490	314	2515	2516	4435	677	147	147	9217	8697	2084
catr	9229	4009	4013	4027	4036	4042	980	558	981	982	985	1398	725	8972	2731	catr_04131	2497	1643	662	2739	126	129	2497	2496	9230	9231	9232	9233	9234	9235	2950
catr	21546	734	733	732	731	730	730	729	728	727	726	725	9264	724	723	catr_05380	722	721	719	718	147	716	715	3280	3281	3282	3283	21547	3284	3286	8972
capa	4219	4218	4217	4216	4192	8686	314	8687	1677	4231	8392	8688	4233	4237	4236	capa_03952	2766	2765	2764	8689	2713	2715	2716	2718	992	236	280	2719	147	8690	455
capa	1253	8691	3010	8691	601	475	8478	2659	432	454	455	8690	147	2719	280	capa_03962	992	2718	2716	2715	2713	8689	2764	2765	2766	236	4236	4237	4233	8688	8392
capa	3943	3727	1221	8832	8831	1223	1226	978	1229	2447	8830	2445	190	3950	845	capa_05437	722	849	2419	2420	746	745	8829	8828	8827	741	740	739	739	1205	3385
loel	3078	591	12845	22078	3082	12844	12843	3086	3086	1407	2709	2709	2710	12842	2767	loel_00428	2766	2765	2764	12841	2762	2761	2760	2758	2757	1921	241	7437	2793	2795	2796
loel	1040	73	2451	2450	147	13198	2449	13197	727	726	725	22114	13196	13195	724	loel_03789	722	721	13194	147	716	715	13193	712	2290	2291	2292	3453	3445	3438	3412
cagu	3454	3307	3452	6476	5348	813	812	6475	811	692	816	82	6474	724	724	cagu_00844	722	721	719	6473	147	716	715	6472	712	2290	4621	4619	4618	2481	2064
cagu	4494	4494	4493	147	4492	4491	473	4489	1569	6529	6530	2412	6531	6532	2410	cagu_01265	2409	2408	2405	2404	2403	2402	1740	2398	6533	6534	3284	3901	3902	6535	6536
cagu	301	2979	959	2831	468	468	442	1314	3828	3829	57	3830	3831	3832	7008	cagu_04884	1643	280	199	7063	7065	7064	468	662	4238	4241	4240	4239	3226	235	4429
deha	1137	9659	2121	21651	6348	7948	97	912	340	340	4165	9660	291	4434	2611	deha2d01210g	280	9661	7064	9662	4165	912	9663	3164	340	7063	199	468	662	4241	4240
deha	1528	1529	3923	781	782	5110	814	190	813	812	811	692	816	82	724	deha2e15070g	722	721	719	9843	147	716	715	9844	712	2290	21724	2291	2292	3344	4674

pist	1298	15200	1816	1816	15183	1112	1289	1288	1305	2589	2588	2586	397	972	440	pist_igi16258608	14809	14827	728	1262	1261	1260	15249	1257	22273	1322	1323	1324	1325	2582	2583
pist	0	0	0	0	0	0	0	1919	236	9117	1539	1804	5862	3350	3349	pist_igi16597754	3348	3351	5811	3352	1476	3356	4264	4266	2570	4268	4269	3884	3883	4197	4196
pist	14952	90	376	375	374	373	1398	14953	15278	2090	14954	4629	2914	1398	2987	pist_igi16601804	4622	4624	4623	4625	4626	1644	147	4628	4621	4619	4618	24	2242	4617	147
pist	1761	3127	601	601	1201	6830	2451	2450	147	22286	2449	15288	726	725	724	pist_igi16603947	236	722	14916	721	719	9843	147	716	715	14984	712	2290	15167	2291	2292
pist	3127	601	601	1201	6830	2451	2450	147	22286	2449	15288	726	725	724	236	pist_igi16603948	722	14916	721	719	9843	147	716	715	14984	712	2290	15167	2291	2292	3344
pist	3715	525	2305	902	351	490	314	2515	2516	129	128	126	2739	2731	236	pist_igi19986137	287	340	662	1398	725	1075	82	3655	15269	3657	14909	2520	348	14908	525
pist	14908	348	2520	14909	3657	15269	3655	82	1075	725	1398	662	340	287	236	pist_igi19986138	2731	2739	126	128	129	2516	2515	314	490	351	902	2305	525	3715	3714
pist	0	0	0	0	0	0	0	0	0	0	0	0	0	0	1919	pist_igi19985888	9117	1539	1804	5862	3350	3349	236	3348	3351	5811	3352	1476	3356	4264	4266
calu	4373	277	4368	7598	2360	2370	2379	2386	2393	2399	2406	351	2414	945	2281	calu_02219	2267	7599	7600	354	4287	515	7601	516	517	580	7602	572	525	7603	7604
calu	3558	3535	1028	1028	670	670	669	8	44	668	667	4149	2252	82	724	calu_04792	722	721	719	8069	147	696	716	715	712	2290	21379	2291	147	1520	4672
calu	1569	8083	3137	1202	3138	3139	3140	3141	8082	3142	3143	8081	8080	418	236	calu_04864	3145	3146	8079	885	1995	8078	3321	1570	3331	2995	525	4257	1	7623	4241
calu	7623	1	4257	525	2995	3331	1570	3321	8078	1995	885	8079	3146	3145	236	calu_04865	418	8080	8081	3143	3142	8082	3141	3140	3139	3138	1202	3137	8083	1569	4489
pipa	1545	14306	545	14313	22238	876	881	14330	2155	4135	14341	1170	14354	1390	4022	pipa_3g03370	2699	14373	771	3586	14377	622	2795	14393	4355	14398	14402	5329	14407	4257	1972

Homologs of SGE1, AZR1, VBA3 and VBA5 genes. The protein family classification of the adjacent genes is shown and the homologous neighbours are highlighted in the same colour.

sace_a	Null	Null	Null	Null	Null	Null	Null	Null	Null	Null	Null	Null	Null	Null	sace0c00110g	sace0c00132g	sace0c00154g	sace0c00176g	sace0c00198g	sace0c00220g	sace0c00242g	sace0c00264g	sace0c00286g	sace0c00308g	sace0c00330g	sace0c00352g	sace0c00374g	sace0c00396g	sace0c00418g	sace0c00440g	sace0c00462g
sace_a	sace0g10604g	sace0g10626g	sace0g10648g	sace0g10670g	sace0g10692g	sace0g10714g	sace0g10736g	sace0g10758g	sace0g10780g	sace0g10802g	sace0g10846g	sace0g10868g	sace0g10890g	sace0g10956g	sace0g10978g	sace0g11000g	sace0g11022g	sace0g11044g	sace0g11066g	sace0g11088g	sace0g11110g	sace0g11132g	sace0g11154g	sace0g11176g	sace0g11198g	sace0g11220g	sace0g11242g	sace0g11264g	sace0g11286g	sace0g11308g	sace0g11330g
sace_a	sace0k07062g	sace0k07084g	sace0k07106g	sace0k07128g	sace0k07150g	sace0k07172g	sace0k07194g	sace0k07216g	sace0k07238g	sace0k07260g	sace0k07282g	sace0k07304g	sace0k07326g	sace0k07348g	sace0k07370g	sace0k07392g	sace0k07414g	Null	Null	Null	Null	Null	Null	Null	Null	Null	Null	Null	Null	Null	Null
sace_a	sace0p10142g	sace0p10164g	sace0p10186g	sace0p10208g	sace0p10230g	sace0p10252g	sace0p10274g	sace0p10296g	sace0p10318g	sace0p10340g	sace0p10362g	sace0p10384g	sace0p10406g	sace0p10428g	sace0p10450g	sace0p10472g	sace0p10494g	sace0p10516g	sace0p10538g	sace0p10560g	sace0p10582g	sace0p10604g	Null	Null	Null	Null	Null	Null	Null	Null	Null
sace_b	sace_b_1g1_5391g	sace_b_1g1_5402g	sace_b_1g1_5413g	sace_b_1g1_5424g	sace_b_1g1_5446g	sace_b_1g1_5457g	sace_b_1g1_5468g	sace_b_1g1_5479g	sace_b_1g1_5490g	sace_b_1g1_5501g	sace_b_1g1_5512g	sace_b_1g1_5523g	sace_b_1g1_5534g	sace_b_1g1_5545g	sace_b_1g1_5556g	sace_b_1g1_5567g	sace_b_1g1_5578g	sace_b_1g1_5589g	sace_b_1g1_5600g	sace_b_1g1_5611g	sace_b_1g1_5622g	sace_b_1g1_5633g	sace_b_1g1_5644g	sace_b_1g1_5655g	sace_b_1g1_5666g	sace_b_1g1_5677g	sace_b_1g1_5688g	sace_b_1g1_5699g	sace_b_1g1_5710g	sace_b_1g1_5732g	sace_b_1g1_5743g
sace_b	sace_b_1p2_5292g	sace_b_1p2_5303g	sace_b_1p2_5314g	sace_b_1p2_5325g	sace_b_1p2_5336g	sace_b_1p2_5347g	sace_b_1p2_5358g	sace_b_1p2_5369g	sace_b_1p2_5380g	sace_b_1p2_5391g	sace_b_1p2_5402g	sace_b_1p2_5413g	sace_b_1p2_5424g	sace_b_1p2_5435g	sace_b_1p2_5446g	sace_b_1p2_5457g	sace_b_1p2_5468g	sace_b_1p2_5479g	sace_b_1p2_5490g	sace_b_1p2_5512g	Null	Null	Null	Null	Null	Null	Null	Null	Null	Null	Null
sace_c	Null	Null	Null	Null	Null	Null	Null	Null	Null	Null	sace_c_01836	sace_c_01807	sace_c_01842	sace_c_01806	sace_c_01821	sace_c_01814	sace_c_01803	sace_c_01852	sace_c_01816	sace_c_01865	sace_c_01808	sace_c_01831	sace_c_01802	sace_c_01826	sace_c_01825	sace_c_01847	sace_c_01830	sace_c_01845	sace_c_01841	sace_c_01804	sace_c_01864
sace_c	sace_c_02092	sace_c_02077	sace_c_02070	sace_c_02090	sace_c_02075	sace_c_02082	sace_c_02055	sace_c_02091	sace_c_02083	sace_c_02080	sace_c_02059	sace_c_02074	sace_c_02056	sace_c_02088	sace_c_02079	sace_c_02068	sace_c_02072	sace_c_02060	sace_c_02069	Null	Null	Null	Null	Null	Null	Null	Null	Null	Null	Null	Null
sace_d	sace_d_00814	sace_d_00813	sace_d_00812	sace_d_00811	sace_d_00810	sace_d_00809	sace_d_00808	sace_d_00807	sace_d_00806	sace_d_00805	sace_d_00803	sace_d_00802	sace_d_00801	sace_d_00800	sace_d_00799	sace_d_00798	sace_d_00797	sace_d_00796	sace_d_00795	sace_d_00794	sace_d_00793	sace_d_00792	sace_d_00791	sace_d_00790	sace_d_00789	sace_d_00788	sace_d_00787	sace_d_00786	sace_d_00785	sace_d_00784	sace_d_00783
sace_d	sace_d_04105	sace_d_04106	sace_d_04107	sace_d_04108	sace_d_04109	sace_d_04110	sace_d_04111	sace_d_04112	sace_d_04113	sace_d_04114	sace_d_04115	sace_d_04116	sace_d_04117	sace_d_04118	sace_d_04119	sace_d_04120	sace_d_04121	sace_d_04122	sace_d_04123	Null	Null	Null	Null	Null	Null	Null	Null	Null	Null	Null	Null
sace_d	Null	Null	Null	Null	Null	Null	Null	Null	Null	Null	Null	Null	Null	sace_d_05342	sace_d_05343	sace_d_05344	sace_d_05345	sace_d_05346	sace_d_05347	sace_d_05348	sace_d_05349	sace_d_05350	sace_d_05351	sace_d_05352	sace_d_05353	sace_d_05354	sace_d_05355	sace_d_05356	sace_d_05357	sace_d_05358	sace_d_05359
sace_e	Null	Null	Null	Null	Null	Null	Null	Null	Null	Null	Null	Null	Null	Null	sace_e_0517	sace_e_0518	sace_e_0519	sace_e_0520	Null	Null	Null	Null	Null	Null	Null	Null	Null	Null	Null	Null	Null
sace_e	sace_e_2102	sace_e_2103	sace_e_2104	sace_e_2105	sace_e_2106	sace_e_2107	sace_e_2108	sace_e_2109	sace_e_2110	sace_e_2111	sace_e_2112	sace_e_2113	sace_e_2114	sace_e_2115	sace_e_2116	sace_e_2117	sace_e_2118	sace_e_2119	sace_e_2120	sace_e_2121	sace_e_2122	sace_e_2123	sace_e_2124	sace_e_2125	sace_e_2126	sace_e_2127	sace_e_2128	sace_e_2129	sace_e_2130	sace_e_2131	sace_e_2132
sace_e	sace_e_3459	sace_e_3460	sace_e_3461	sace_e_3462	sace_e_3463	sace_e_3464	sace_e_3465	sace_e_3466	sace_e_3467	sace_e_3468	sace_e_3469	sace_e_3470	sace_e_3471	sace_e_3472	sace_e_3473	sace_e_3474	Null	Null	Null	Null	Null	Null	Null	Null	Null	Null	Null	Null	Null	Null	Null
sace_e	sace_e_5882	sace_e_5883	sace_e_5884	sace_e_5885	sace_e_5886	sace_e_5887	sace_e_5888	sace_e_5889	sace_e_5890	sace_e_5891	sace_e_5892	sace_e_5893	sace_e_5894	sace_e_5895	sace_e_5896	sace_e_5897	sace_e_5898	sace_e_5899	sace_e_5900	sace_e_5901	Null	Null	Null	Null	Null	Null	Null	Null	Null	Null	Null
sapa	sapa_c290_9150	sapa_c290_9154	sapa_c290_9156	sapa_c290_9160	sapa_c290_9161	sapa_c290_9162	sapa_c290_9164	sapa_c290_9169	sapa_c290_9171	sapa_c290_9180	sapa_c290_9179	sapa_c290_9182	sapa_c290_9187	sapa_c290_9190	sapa_c290_9194	sapa_c290_9193	sapa_c290_9200	sapa_c290_9202	sapa_c290_9203	sapa_c290_9204	sapa_c290_9207	sapa_c290_9208	sapa_c290_9212	sapa_c290_9215	sapa_c290_9218	sapa_c290_9220	sapa_c290_9224	sapa_c290_9231	sapa_c290_9233	sapa_c290_9237	sapa_c290_9242
sapa	Null	Null	Null	Null	Null	Null	Null	Null	Null	Null	Null	Null	Null	sapa_c403_13072	sapa_c403_13063	sapa_c403_13057	sapa_c403_13053	Null	Null	Null	Null	Null	Null	Null	Null	Null	Null	Null	Null	Null	Null
sapa	sapa_c565_22754	sapa_c565_22745	sapa_c565_22739	sapa_c565_22737	sapa_c565_22734	sapa_c565_22730	sapa_c565_22725	sapa_c565_22719	sapa_c565_22718	sapa_c565_22711	sapa_c565_22708	sapa_c565_22704	sapa_c565_22689	sapa_c565_22687	sapa_c565_22686	sapa_c565_22682	sapa_c565_22678	sapa_c565_22674	Null	Null	Null	Null	Null	Null	Null	Null	Null	Null	Null	Null	Null
sapa	Null	Null	Null	Null	Null	Null	Null	Null	Null	Null	Null	Null	Null	Null	sapa_c84_2183	sapa_c84_2187	sapa_c84_2195	sapa_c84_2197	sapa_c84_2199	sapa_c84_2201	sapa_c84_2205	sapa_c84_2209	sapa_c84_2210	sapa_c84_2217	sapa_c84_2222	sapa_c84_2224	sapa_c84_2229	sapa_c84_2230	sapa_c84_2238	sapa_c84_2243	sapa_c84_2247
sami_a	Null	Null	Null	Null	Null	Null	Null	Null	Null	Null	Null	Null	sami_a_c124_14108	sami_a_c124_14109	sami_a_c124_14112	sami_a_c124_14118	sami_a_c124_14126	sami_a_c124_14128	Null	Null	Null	Null	Null	Null	Null	Null	Null	Null	Null	Null	Null
sami_a	Null	Null	Null	Null	Null	Null	Null	Null	Null	Null	Null	sami_b_c208.1	sami_a_c208_6702	sami_a_c208_6712	sami_a_c208_6716	sami_a_c208_6715	sami_a_c208_6723	sami_a_c208_6726	Null	Null	Null	Null	Null	Null	Null	Null	Null	Null	Null	Null	Null
sami_a	Null	Null	Null	Null	Null	Null	Null	Null	Null	Null	Null	Null	Null	Null	Null	sami_a_c798_9285	sami_a_c798_9284	sami_a_c798_9283	sami_a_c798_9281	Null	Null	Null	Null	Null	Null	Null	Null	Null	Null	Null	Null
sami_a	Null	Null	Null	Null	Null	Null	Null	Null	Null	sami_a_c989_9304	sami_a_c989_9302	sami_a_c989_9299	sami_a_c989_9294	sami_a_c989_9292	sami_a_c989_9291	sami_a_c989_9288	Null	Null	Null	Null	Null	Null	Null	Null	Null	Null	Null	Null	Null	Null	Null
sami_b	Null	Null	Null	Null	Null	Null	Null	Null	Null	Null	Null	Null	Null	Null	Null	sami_b_c2470.1	sami_b_c2470.2	Null	Null	Null	Null	Null	Null	Null	Null	Null	Null	Null	Null	Null	Null
saku	Null	Null	Null	Null	Null	Null	Null	Null	Null	Null	Null	Null	Null	Null	saku_c1565.2	saku_c1565.3	Null	Null	Null	Null	Null	Null	Null	Null	Null	Null	Null	Null	Null	Null	Null
saca	saca_713.45	saca_713.44	saca_713.43	saca_713.42	saca_713.41	saca_713.40	saca_713.39	saca_713.38	saca_713.37	saca_713.36	saca_713.35	saca_713.34	saca_713.33	saca_713.32	saca_713.31	saca_713.30	saca_713.29	saca_713.28	saca_713.27	saca_713.26	saca_713.25	saca_713.24	saca_713.23	saca_713.22	saca_713.21d	saca_713.21	saca_713.20	saca_713.19	saca_713.18	saca_713.17	saca_713.16
cagl	cagl0b02409g	cagl0b02387g	cagl0b02365g	cagl0b02343g	cagl0b02321g	cagl0b02299g	cagl0b02277g	cagl0b02255g	cagl0b02233g	cagl0b02211g	cagl0b02189g	cagl0b02167g	cagl0b02145g	cagl0b02123g	cagl0b02101g	cagl0b02079g	cagl0b02057g	cagl0b02035g	cagl0b02013g	cagl0b01991g	cagl0b01969g	cagl0b01947g	cagl0b01925g	cagl0b01903g	cagl0b01881g	cagl0b01875g	cagl0b01859g	cagl0b01837g	cagl0b01815g	cagl0b01793g	cagl0b01771g
sakl	sakl0a00374g	sakl0a00396g	sakl0a00418g	sakl0a00440g	sakl0a00462g	sakl0a00484g	sakl0a00506g	sakl0a00528g	sakl0a00550g	sakl0a00572g	sakl0a00594g	sakl0a00616g	sakl0a00638g	sakl0a00660g	sakl0a00704g	sakl0a00726g	sakl0a00748g	sakl0a00770g	sakl0a00792g	sakl0a00814g	sakl0a00836g	sakl0a00858g	sakl0a00880g	sakl0a00902g	sakl0a00924g	sakl0a00946g	sakl0a00968g	sakl0a00990g	sakl0a01012g	sakl0a01034g	sakl0a01056g
sakl	sakl0a08646g	sakl0a08624g	sakl0a08602g	sakl0a08580g	sakl0a08558g	sakl0a08536g	sakl0a08514g	sakl0a08492g	sakl0a08470g	sakl0a08448g	sakl0a08426g	sakl0a08404g	sakl0a08382g	sakl0a08360g	sakl0a08250g	sakl0a08228g	sakl0a08206g	sakl0a08184g	sakl0a08162g	sakl0a08140g	sakl0a08118g	sakl0a08096g	sakl0a08074g	sakl0a08052g	sakl0a08030g	sakl0a08008g	sakl0a07986g	sakl0a07964g	sakl0a07942g	sakl0a07920g	sakl0a07898g
sakl	sakl0b12386g	sakl0b12408g	sakl0b12430g	sakl0b12452g	sakl0b12474g	sakl0b12496g	sakl0b12518g	sakl0b12540g	sakl0b12562g	sakl0b12584g	sakl0b12606g	sakl0b12628g	sakl0b12650g	sakl0b12672g	sakl0b12694g	sakl0b12716g	sakl0b12738g	sakl0b12760g	sakl0b12804g	sakl0b12826g	Null	Null	Null	Null	Null	Null	Null	Null	Null	Null	Null
sakl	sakl0c05214g	sakl0c05236g	sakl0c05258g	sakl0c05280g	sakl0c05302g	sakl0c05324g	sakl0c05346g	sakl0c05368g	sakl0c05390g	sakl0c05412g	sakl0c05434g	sakl0c05456g	sakl0c05522g	sakl0c05544g	sakl0c05566g	sakl0c05588g	sakl0c05632g	sakl0c05654g	sakl0c05676g	sakl0c05698g	sakl0c05720g	sakl0c05742g	sakl0c05764g	sakl0c05786g	sakl0c05808g	sakl0c05830g	sakl0c05852g	sakl0c05896g	sakl0c05918g	sakl0c05940g	sakl0c06006g
sakl	Null	Null	Null	Null	Null	Null	Null	Null	Null	Null	Null	Null	sakl0f00110g	sakl0f00132g	sakl0f00176g	sakl0f00198g	sakl0f00242g	sakl0f00286g	sakl0f00308g	sakl0f00330g	sakl0f00352g	sakl0f00374g	sakl0f00396g	sakl0f00418g	sakl0f00440g	sakl0f00462g	sakl0f00484g	sakl0f00506g	sakl0f00528g	sakl0f00550g	sakl0f00572g
klwa	Null	Null	Null	Null	Null	Null	Null	Null	Null	Null	Null	Null	Null	klwa_116-snap.18	klwa_116-snap.17	klwa_116-snap.16	klwa_116-snap.15	klwa_116-snap.14	klwa_116-snap.13	klwa_116-snap.12	klwa_116-snap.11	klwa_116-snap.10	klwa_116-snap.9	klwa_116-snap.8	klwa_116-snap.7	klwa_116-snap.6	klwa_116-snap.5	klwa_116-snap.4	klwa_116-snap.3	klwa_116-snap.2	klwa_116-snap.1
klwa	Null	Null	Null	Null	Null	Null	Null	Null	Null	Null	Null	Null	klwa_298-snap.1	klwa_298-snap.2	klwa_298-snap.3	klwa_298-snap.4	klwa_298-snap.5	klwa_298-snap.6	klwa_298-snap.7	klwa_298-snap.8	klwa_298-snap.9	klwa_298-snap.10	klwa_298-snap.11	klwa_298-snap.12	klwa_298-snap.13	klwa_298-snap.14	klwa_298-snap.15	klwa_298-snap.16	klwa_298-snap.17	klwa_298-snap.18	klwa_298-snap.19
klth	klth0b00506g	klth0b00484g	klth0b00462g	klth0b00440g	klth0b00418g	klth0b00396g	klth0b00374g	klth0b00352g	klth0b00330g	klth0b00308g	klth0b00286g	klth0b00264g	klth0b00220g	klth0b00176g	klth0b00154g	klth0b00132g	klth0b00110g	Null	Null	Null	Null	Null	Null	Null	Null	Null	Null	Null	Null	Null	Null
klth	klth0b00264g	klth0b00286g	klth0b00308g	klth0b00330g	klth0b00352g	klth0b00374g	klth0b00396g	klth0b00418g	klth0b00440g	klth0b00462g	klth0b00484g	klth0b00506g	klth0b00528g	klth0b00550g	klth0b00572g	klth0b00594g	klth0b00616g	klth0b00704g	klth0b00770g	klth0b00836g	klth0b00858g	klth0b00880g	klth0b00902g	klth0b00924g	klth0b00946g	klth0b00968g	klth0b00990g	klth0b01034g	klth0b01056g	klth0b01078g	klth0b01100g
klth	Null	Null	klth0b10494g	klth0b10472g	klth0b10450g	klth0b10428g	klth0b10384g	klth0b10340g	klth0b10318g	klth0b10296g	klth0b10274g	klth0b10252g	klth0b10230g	klth0b10186g	klth0b10164g	klth0b10142g	klth0b10098g	klth0b10076g	klth0b10054g	klth0b10032g	klth0b09966g	klth0b09944g	klth0b09922g	klth0b09900g	klth0b09878g	klth0b09856g	klth0b09834g	klth0b09812g	klth0b09790g	klth0b09768g	klth0b09746g
klth	klth0c09790g	klth0c09812g	klth0c09834g	klth0c09856g	klth0c09878g	klth0c09900g	klth0c09922g	klth0c09944g	klth0c09966g	klth0c09988g	klth0c10010g	klth0c10032g	klth0c10054g	klth0c10076g	klth0c10098g	klth0c10120g	klth0c10142g	klth0c10164g	klth0c10186g	klth0c10208g	klth0c10230g	klth0c10252g	klth0c10274g	klth0c10296g	klth0c10318g	klth0c10340g	klth0c10362g	klth0c10384g	klth0c10406g	klth0c10428g	klth0c10450g
klth	klth0c09812g	klth0c09834g	klth0c09856g	klth0c09878g	klth0c09900g	klth0c09922g	klth0c09944g	klth0c09966g	klth0c09988g	klth0c10010g	klth0c10032g	klth0c10054g	klth0c10076g	klth0c10098g	klth0c10120g	klth0c10142g	klth0c10164g	klth0c10186g	klth0c10208g	klth0c10230g	klth0c10252g	klth0c10274g	klth0c10296g	klth0c10318g	klth0c10340g	klth0c10362g	klth0c10384g	klth0c10406g	klth0c10428g	klth0c10450g	klth0c10494g
klth	klth0d17336g	klth0d17314g	klth0d17270g	klth0d17248g	klth0d17226g	klth0d17204g	klth0d17182g	klth0d17160g	klth0d17138g	klth0d17116g	klth0d17094g	klth0d17072g	klth0d17050g	klth0d17006g	klth0d16984g	klth0d16962g	klth0d16940g	klth0d16918g	klth0d16896g	klth0d16874g	klth0d16852g	klth0d16830g	klth0d16808g	klth0d16786g	klth0d16764g	klth0d16698g	klth0d16676g	klth0d16654g	klth0d16632g	klth0d16610g	klth0d16588g
klla	Null	Null	Null	Null	Null	Null	Null	klla0d20031g	klla0d20009g	klla0d19987g	klla0d19965g	klla0d19957g	klla0d19953g	klla0d19949g	klla0d19945g	klla0d19941g	klla0d19933g	klla0d19929g	klla0d19921g	klla0d19899g	klla0d19877g	klla0d19855g	klla0d19833g	klla0d19811g	klla0d19789g	klla0d19767g	klla0d19745g	klla0d19723g	klla0d19701g	klla0d19646g	klla0d19624g
klla	klla0e10209g	klla0e10187g	klla0e10165g	klla0e10143g	klla0e10121g	klla0e10099g	klla0e10077g	klla0e10055g	klla0e10033g	klla0e10011g	klla0e09989g	klla0e09967g	klla0e09945g	klla0e09923g	klla0e09901g	klla0e09879g	klla0e09857g	klla0e09835g	klla0e09813g	klla0e09791g	klla0e09769g	klla0e09747g	klla0e09725g	klla0e09703g	klla0e09681g	klla0e09637g	klla0e09615g	klla0e09593g	klla0e09571g	klla0e09549g	klla0e09527g
caal_a	caal_a_19.1960	caal_a_19.1959	caal_a_19.1958	caal_a_19.1957	caal_a_19.1956	caal_a_19.1955	caal_a_19.1954	caal_a_19.1953	caal_a_19.1950	caal_a_19.1949	caal_a_19.1948	caal_a_19.1946	caal_a_19.1945	caal_a_19.1944	caal_a_19.1943	caal_a_19.1942	caal_a_19.1941	caal_a_19.1940	caal_a_19.1939	caal_a_19.1938	caal_a_19.1936	caal_a_19.1935	caal_a_19.1934	caal_a_19.1933	caal_a_19.1932	caal_a_19.1930	caal_a_19.1927	caal_a_19.1926	caal_a_19.4131	caal_a_19.4132	caal_a_19.4133
caal_a	caal_a_19.132	caal_a_19.133	caal_a_19.134	caal_a_19.135	caal_a_19.136	caal_a_19.137	caal_a_19.138	caal_a_19.139	caal_a_19.3450.1	caal_a_19.3449.2	caal_a_19.3449	caal_a_19.3448	caal_a_19.3447	caal_a_19.3446	caal_a_19.3445	caal_a_19.3444	caal_a_19.3443	caal_a_19.3442	caal_a_19.3441	caal_a_19.3440	caal_a_19.3439	caal_a_19.3438	caal_a_19.3437	caal_a_19.3436	caal_a_19.3435	caal_a_19.3434	caal_a_19.3433	caal_a_19.3432	caal_a_19.3431	caal_a_19.3430	caal_a_19.3429
caal_a	caal_a_19.4763	caal_a_19.4764	caal_a_19.4765	caal_a_19.4766	caal_a_19.4767	caal_a_19.4768	caal_a_19.4769	caal_a_19.4771	caal_a_19.4772	caal_a_19.4773	caal_a_19.4774	caal_a_19.4775	caal_a_19.4776	caal_a_19.4777	caal_a_19.4778	caal_a_19.4779	caal_a_19.4780	caal_a_19.4781	caal_a_19.4783	caal_a_19.4784	caal_a_19.4785	caal_a_19.4786	caal_a_19.4787	caal_a_19.4788	caal_a_19.4789	caal_a_19.4791	caal_a_19.4792	caal_a_19.4793	caal_a_19.4795	caal_a_19.4796	caal_a_19.4798
caal_b	caal_b_00523	caal_b_00522	caal_b_00521	caal_b_00520	caal_b_00519	caal_b_00518	caal_b_00517	caal_b_00516	caal_b_00515	caal_b_00514	caal_b_00513	caal_b_00512	caal_b_00511	caal_b_00510	caal_b_00509	caal_b_00508	caal_b_00507	caal_b_00506	caal_b_00505	caal_b_00504	caal_b_00503	caal_b_00502	caal_b_00501	caal_b_00500	caal_b_00499	caal_b_00498	caal_b_00497	caal_b_00496	caal_b_00495	caal_b_00494	caal_b_00493
caal_b	caal_b_04500	caal_b_04501	caal_b_04502	caal_b_04503	caal_b_04504	caal_b_04505	caal_b_04506	caal_b_04507	caal_b_04508	caal_b_04509	caal_b_04510	caal_b_04511	caal_b_04512	caal_b_04513	caal_b_04514	caal_b_04515	caal_b_04516	caal_b_04517	caal_b_04518	caal_b_04519	caal_b_04520	caal_b_04521	caal_b_04522	caal_b_04523	caal_b_04524	caal_b_04525	caal_b_04526	caal_b_04527	caal_b_04528	caal_b_04529	caal_b_04530
caal_b	caal_b_05239	caal_b_05238	caal_b_05237	caal_b_05236	caal_b_05235	caal_b_05234	caal_b_05233	caal_b_05232	caal_b_05231	caal_b_05230	caal_b_05229	caal_b_05228	caal_b_05227	caal_b_05226	caal_b_05225	caal_b_05224	caal_b_05223	caal_b_05222	caal_b_05221	caal_b_05220	caal_b_05219	caal_b_05218	caal_b_05217	caal_b_05216	caal_b_05215	caal_b_05214	caal_b_05213	caal_b_05212	caal_b_05211	caal_b_05210	caal_b_05209
cadu	cadu_08540	cadu_08550	cadu_08560	cadu_08570	cadu_08580	cadu_08590	cadu_08600	cadu_08610	cadu_08620	cadu_08630	cadu_08640	cadu_08650	cadu_08660	cadu_08670	cadu_08680	cadu_08690	cadu_08700	cadu_08710	cadu_08720	cadu_08730	cadu_08740	cadu_08745	cadu_08750	cadu_08760	cadu_08770	cadu_08780	cadu_08790	cadu_08800	cadu_08810	cadu_08820	cadu_08830
cadu	cadu_51210	cadu_51200	cadu_51190	cadu_51180	cadu_51170	cadu_51160	cadu_51150	cadu_51140	cadu_51130	cadu_51120	cadu_51110	cadu_51100	cadu_51090	cadu_51080	cadu_51070	cadu_51060	cadu_51050	cadu_51040	cadu_51030	cadu_51020	cadu_51010	cadu_51000	cadu_50990	cadu_50980	cadu_50970	cadu_50960	cadu_50950	cadu_50940	cadu_50930	cadu_50920	cadu_50910
cadu	cadu_61330	cadu_61340	cadu_61350	cadu_61370	cadu_61380	cadu_61400	cadu_61410	cadu_61420	cadu_61450	cadu_61455	cadu_61460	cadu_61470	cadu_61480	cadu_61490	cadu_61500	cadu_61510	cadu_61520	cadu_61530	cadu_61540	cadu_61550	cadu_61560	cadu_61570	cadu_61580	cadu_61600	cadu_61610	cadu_61630	cadu_61640	cadu_61650	cadu_61660	cadu_61670	cadu_61690
catr	catr_02818	catr_02819	catr_02820	catr_02821	catr_02822	catr_02823	catr_02824	catr_02825	catr_02826	catr_02827	catr_02828	catr_02829	catr_02830	catr_02831	catr_02832	catr_02833	catr_02834	catr_02835	catr_02836	catr_02837	catr_02838	catr_02839	catr_02840	catr_02841	catr_02842	catr_02843	catr_02844	catr_02845	catr_02846	catr_02847	catr_02848
catr	catr_03943	catr_03944	catr_03945	catr_03946	catr_03947	catr_03948	catr_03949	catr_03950	catr_03951	catr_03952	catr_03953	catr_03954	catr_03955	catr_03956	catr_03957	catr_03958	catr_03959	catr_03960	catr_03961	catr_03962	catr_03963	catr_03964	catr_03965	catr_03966	catr_03967	catr_03968	catr_03969	catr_03970	catr_03971	catr_03972	catr_03973
catr	catr_03975	catr_03974	catr_03973	catr_03972	catr_03971	catr_03970	catr_03969	catr_03968	catr_03967	catr_03966	catr_03965	catr_03964	catr_03963	catr_03962	catr_03961	catr_03960	catr_03959	catr_03958	catr_03957	catr_03956	catr_03955	catr_03954	catr_03953	catr_03952	catr_03951	catr_03950	catr_03949	catr_03948	catr_03947	catr_03946	catr_03945
catr	catr_04116	catr_04117	catr_04118	catr_04119	catr_04120	catr_04121	catr_04122	catr_04123	catr_04124	catr_04125	catr_04126	catr_04127	catr_04128	catr_04129	catr_04130	catr_04131	catr_04132	catr_04133	catr_04134	catr_04135	catr_04136	catr_04137	catr_04138	catr_04139	catr_04140	catr_04141	catr_04142	catr_04143	catr_04144	catr_04145	catr_04146
catr	catr_05365	catr_05366	catr_05367	catr_05368	catr_05369	catr_05370	catr_05371	catr_05372	catr_05373	catr_05374	catr_05375	catr_05376	catr_05377	catr_05378	catr_05379	catr_05380	catr_05381	catr_05382	catr_05383	catr_05384	catr_05385	catr_05386	catr_05387	catr_05388	catr_05389	catr_05390	catr_05391	catr_05392	catr_05393	catr_05394	catr_05395
capa	capa_03937	capa_03938	capa_03939	capa_03940	capa_03941	capa_03942	capa_03943	capa_03944	capa_03945	capa_03946	capa_03947	capa_03948	capa_03949	capa_03950	capa_03951	capa_03952	capa_03953	capa_03954	capa_03955	capa_03956	capa_03957	capa_03958	capa_03959	capa_03960	capa_03961	capa_03962	capa_03963	capa_03964	capa_03965	capa_03966	capa_03967
capa	capa_03977	capa_03976	capa_03975	capa_03974	capa_03973	capa_03972	capa_03971	capa_03970	capa_03969	capa_03968	capa_03967	capa_03966	capa_03965	capa_03964	capa_03963	capa_03962	capa_03961	capa_03960	capa_03959	capa_03958	capa_03957	capa_03956	capa_03955	capa_03954	capa_03953	capa_03952	capa_03951	capa_03950	capa_03949	capa_03948	capa_03947
capa	capa_05452	capa_05451	capa_05450	capa_05449	capa_05448	capa_05447	capa_05446	capa_05445	capa_05444	capa_05443	capa_05442	capa_05441	capa_05440	capa_05439	capa_05438	capa_05437	capa_05436	capa_05435	capa_05434	capa_05433	capa_05432	capa_05431	capa_05430	capa_05429	capa_05428	capa_05427	capa_05426	capa_05425	capa_05424	capa_05423	capa_05422
loel	loel_00443	loel_00442	loel_00441	loel_00440	loel_00439	loel_00438	loel_00437	loel_00436	loel_00435	loel_00434	loel_00433	loel_00432	loel_00431	loel_00430	loel_00429	loel_00428	loel_00427	loel_00426	loel_00425	loel_00424	loel_00423	loel_00422	loel_00421	loel_00420	loel_00419	loel_00418	loel_00417	loel_00416	loel_00415	loel_00414	loel_00413
loel	loel_03804	loel_03803	loel_03802	loel_03801	loel_03800	loel_03799	loel_03798	loel_03797	loel_03796	loel_03795	loel_03794	loel_03793	loel_03792	loel_03791	loel_03790	loel_03789	loel_03788	loel_03787	loel_03786	loel_03785	loel_03784	loel_03783	loel_03782	loel_03781	loel_03780	loel_03779	loel_03778	loel_03777	loel_03776	loel_03775	loel_03774

cagu	cagu_00859	cagu_00858	cagu_00857	cagu_00856	cagu_00855	cagu_00854	cagu_00853	cagu_00852	cagu_00851	cagu_00850	cagu_00849	cagu_00848	cagu_00847	cagu_00846	cagu_00845	cagu_00844	cagu_00843	cagu_00842	cagu_00841	cagu_00840	cagu_00839	cagu_00838	cagu_00837	cagu_00836	cagu_00835	cagu_00834	cagu_00833	cagu_00832	cagu_00831	cagu_00830	cagu_00829
cagu	cagu_01250	cagu_01251	cagu_01252	cagu_01253	cagu_01254	cagu_01255	cagu_01256	cagu_01257	cagu_01258	cagu_01259	cagu_01260	cagu_01261	cagu_01262	cagu_01263	cagu_01264	cagu_01265	cagu_01266	cagu_01267	cagu_01268	cagu_01269	cagu_01270	cagu_01271	cagu_01272	cagu_01273	cagu_01274	cagu_01275	cagu_01276	cagu_01277	cagu_01278	cagu_01279	cagu_01280
cagu	cagu_04869	cagu_04870	cagu_04871	cagu_04872	cagu_04873	cagu_04874	cagu_04875	cagu_04876	cagu_04877	cagu_04878	cagu_04879	cagu_04880	cagu_04881	cagu_04882	cagu_04883	cagu_04884	cagu_04885	cagu_04886	cagu_04887	cagu_04888	cagu_04890	cagu_04889	cagu_04891	cagu_04892	cagu_04893	cagu_04894	cagu_04895	cagu_04896	cagu_04897	cagu_04898	cagu_04899
deha	deha2d00880g	deha2d00902g	deha2d00924g	deha2d00946g	deha2d00968g	deha2d00990g	deha2d01012g	deha2d01034g	deha2d01056g	deha2d01078g	deha2d01100g	deha2d01122g	deha2d01144g	deha2d01166g	deha2d01188g	deha2d01210g	deha2d01232g	deha2d01254g	deha2d01276g	deha2d01298g	deha2d01320g	deha2d01342g	deha2d01364g	deha2d01386g	deha2d01408g	deha2d01430g	deha2d01452g	deha2d01474g	deha2d01496g	deha2d01518g	deha2d01540g
deha	deha2e14740g	deha2e14762g	deha2e14784g	deha2e14806g	deha2e14828g	deha2e14850g	deha2e14872g	deha2e14894g	deha2e14916g	deha2e14938g	deha2e14960g	deha2e14982g	deha2e15004g	deha2e15026g	deha2e15048g	deha2e15070g	deha2e15092g	deha2e15114g	deha2e15136g	deha2e15158g	deha2e15180g	deha2e15202g	deha2e15224g	deha2e15246g	deha2e15268g	deha2e15290g	deha2e15312g	deha2e15334g	deha2e15356g	deha2e15378g	deha2e15400g
pist	pist_igi16274355	pist_igi19905643	pist_igi18210222	pist_igi16258603	pist_igi19901627	pist_igi16258604	pist_igi19901628	pist_igi19901629	pist_igi19901630	pist_igi16258605	pist_igi16258606	pist_igi18206208	pist_igi16258607	pist_igi20749651	pist_igi18206209	pist_igi16258608	pist_igi16258609	pist_igi19055026	pist_igi16258610	pist_igi16258611	pist_igi20749652	pist_igi19901631	pist_igi20749653	pist_igi19055027	pist_igi16258612	pist_igi16258613	pist_igi19901632	pist_igi19901633	pist_igi16258614	pist_igi16258615	pist_igi18206210
pist	Null	Null	Null	Null	Null	Null	Null	pist_igi18291044	pist_igi19985888	pist_igi16597755	pist_igi20834538	pist_igi19139405	pist_igi20834537	pist_igi18291043	pist_igi19139404	pist_igi16597754	pist_igi16597753	pist_igi16597752	pist_igi16597751	pist_igi16597750	pist_igi19139403	pist_igi16597749	pist_igi16597748	pist_igi16597747	pist_igi19139402	pist_igi16597746	pist_igi18291042	pist_igi16597745	pist_igi18291041	pist_igi20834536	pist_igi16597744
pist	pist_igi16601791	pist_igi16601792	pist_igi16601793	pist_igi16601794	pist_igi16601795	pist_igi16601796	pist_igi16601797	pist_igi16601798	pist_igi20835627	pist_igi16601799	pist_igi16601800	pist_igi16601801	pist_igi19986938	pist_igi16601802	pist_igi16601803	pist_igi16601804	pist_igi16601805	pist_igi20835628	pist_igi20835629	pist_igi16601806	pist_igi16601807	pist_igi16601808	pist_igi19140402	pist_igi18292098	pist_igi16601809	pist_igi18292099	pist_igi20835630	pist_igi19986939	pist_igi16601810	pist_igi16601811	pist_igi18292100
pist	pist_igi16603941	pist_igi18292632	pist_igi16603942	pist_igi19140983	pist_igi19987488	pist_igi20836198	pist_igi20836199	pist_igi19140984	pist_igi20836200	pist_igi16603943	pist_igi19140985	pist_igi20836201	pist_igi16603944	pist_igi16603945	pist_igi16603946	pist_igi16603947	pist_igi16603948	pist_igi16603949	pist_igi16603950	pist_igi18292633	pist_igi20836202	pist_igi20836203	pist_igi19987489	pist_igi19140986	pist_igi19140987	pist_igi16603951	pist_igi18292634	pist_igi18292635	pist_igi19140988	pist_igi16603952	pist_igi16603953
pist	pist_igi18292632	pist_igi16603942	pist_igi19140983	pist_igi19987488	pist_igi20836198	pist_igi20836199	pist_igi19140984	pist_igi20836200	pist_igi16603943	pist_igi19140985	pist_igi20836201	pist_igi16603944	pist_igi16603945	pist_igi16603946	pist_igi16603947	pist_igi16603948	pist_igi16603949	pist_igi16603950	pist_igi18292633	pist_igi20836202	pist_igi20836203	pist_igi19987489	pist_igi19140986	pist_igi19140987	pist_igi16603951	pist_igi18292634	pist_igi18292635	pist_igi19140988	pist_igi16603952	pist_igi16603953	pist_igi20836204
pist	pist_igi16598787	pist_igi20834809	pist_igi16598786	pist_igi16598785	pist_igi16598784	pist_igi18291327	pist_igi19139664	pist_igi20834808	pist_igi20834807	pist_igi16598783	pist_igi16598782	pist_igi20834806	pist_igi16598781	pist_igi20834805	pist_igi19986138	pist_igi19986137	pist_igi20834804	pist_igi16598780	pist_igi19139663	pist_igi19986136	pist_igi20834552	pist_igi18291061	pist_igi16597812	pist_igi19985902	pist_igi20834551	pist_igi18291060	pist_igi16597811	pist_igi19139415	pist_igi19139414	pist_igi16597810	pist_igi20834550
pist	pist_igi16597810	pist_igi19139414	pist_igi19139415	pist_igi16597811	pist_igi18291060	pist_igi20834551	pist_igi19985902	pist_igi16597812	pist_igi18291061	pist_igi20834552	pist_igi19986136	pist_igi19139663	pist_igi16598780	pist_igi20834804	pist_igi19986137	pist_igi19986138	pist_igi20834805	pist_igi16598781	pist_igi20834806	pist_igi16598782	pist_igi16598783	pist_igi20834807	pist_igi20834808	pist_igi19139664	pist_igi18291327	pist_igi16598784	pist_igi16598785	pist_igi16598786	pist_igi20834809	pist_igi16598787	pist_igi19986139
pist	Null	Null	Null	Null	Null	Null	Null	Null	Null	Null	Null	Null	Null	Null	pist_igi18291044	pist_igi19985888	pist_igi16597755	pist_igi20834538	pist_igi19139405	pist_igi20834537	pist_igi18291043	pist_igi19139404	pist_igi16597754	pist_igi16597753	pist_igi16597752	pist_igi16597751	pist_igi16597750	pist_igi19139403	pist_igi16597749	pist_igi16597748	pist_igi16597747
calu	calu_02204	calu_02205	calu_02206	calu_02207	calu_02208	calu_02209	calu_02210	calu_02211	calu_02212	calu_02213	calu_02214	calu_02215	calu_02216	calu_02217	calu_02218	calu_02219	calu_02220	calu_02221	calu_02222	calu_02223	calu_02224	calu_02225	calu_02226	calu_02227	calu_02228	calu_02229	calu_02230	calu_02231	calu_02232	calu_02233	calu_02234
calu	calu_04777	calu_04778	calu_04779	calu_04780	calu_04781	calu_04782	calu_04783	calu_04784	calu_04785	calu_04786	calu_04787	calu_04788	calu_04789	calu_04790	calu_04791	calu_04792	calu_04793	calu_04794	calu_04795	calu_04796	calu_04797	calu_04798	calu_04799	calu_04800	calu_04801	calu_04802	calu_04803	calu_04804	calu_04805	calu_04806	calu_04807
calu	calu_04879	calu_04878	calu_04877	calu_04876	calu_04875	calu_04874	calu_04873	calu_04872	calu_04871	calu_04870	calu_04868	calu_04869	calu_04867	calu_04866	calu_04865	calu_04864	calu_04863	calu_04862	calu_04861	calu_04860	calu_04859	calu_04858	calu_04857	calu_04856	calu_04855	calu_04854	calu_04853	calu_04852	calu_04851	calu_04850	calu_04849
calu	calu_04850	calu_04851	calu_04852	calu_04853	calu_04854	calu_04855	calu_04856	calu_04857	calu_04858	calu_04859	calu_04860	calu_04861	calu_04862	calu_04863	calu_04864	calu_04865	calu_04866	calu_04867	calu_04869	calu_04868	calu_04870	calu_04871	calu_04872	calu_04873	calu_04874	calu_04875	calu_04876	calu_04877	calu_04878	calu_04879	calu_04880
pipa	pipa_3g02170	pipa_3g02210	pipa_3g02225	pipa_3g02355	pipa_3g02380	pipa_3g02530	pipa_3g02540	pipa_3g02665	pipa_3g02700	pipa_3g02780	pipa_3g02805	pipa_3g02945	pipa_3g03025	pipa_3g03045	pipa_3g03225	pipa_3g03370	pipa_3g03390	pipa_3g03455	pipa_3g03465	pipa_3g03490	pipa_3g03615	pipa_3g03670	pipa_3g03710	pipa_3g03810	pipa_3g03860	pipa_3g03865	pipa_3g03960	pipa_3g03970	pipa_3g04040	pipa_3g04090	pipa_3g04140

Homologs of SGE1, AZR1, VBA3 and VBA5 genes. The names of the adjacent genes are shown and the homologous neighbours are highlighted in the same colour.

Cluster C (lineage 2): homologs of VBA1 and VBA2 genes

sace_a	2483	15492	1935	3381	1099	1536	2281	4651	15491	1922	3613	155	3454	3452	314	sace0b09020g	877	2440	2611	22883	4369	468	22884	2191	12787	15506	0	0	0	0	0
sace_a	6047	2559	5906	3844	929	5173	1661	4682	145	15374	1205	493	493	5748	5747	sace0m05214g	190	4495	813	2475	1932	5746	1449	1448	1047	1050	726	4424	2223	4318	147
sace_b	15492	1935	3381	1099	1536	2281	4651	15491	1922	3613	155	3454	3452	314	16775	sace_b_1b15_4786g	877	2440	2611	23071	4369	0	0	0	0	0	0	0	0	0	0
sace_b	5906	3844	17100	929	5173	1661	4682	145	15374	17101	1205	493	493	5748	5747	sace_b_1m3_2575g	190	4495	813	1932	5746	1449	1448	1047	1050	726	4424	2223	4318	17102	147
sace_c	2789	6047	2559	5906	3844	929	5173	1661	4682	145	15374	1205	493	5748	5747	sace_c_00976	190	4495	813	2475	1932	5746	1449	1448	1047	1050	0	0	0	0	0
sace_c	2483	15492	1935	3381	1099	1536	2281	4651	15491	1922	3613	155	3454	3452	314	sace_c_01581	877	2440	2611	23339	4369	10579	0	0	0	0	0	0	0	0	0
sace_d	2789	6047	2559	5906	3844	929	5173	1661	4682	145	15374	1205	493	5748	5747	sace_d_01978	190	4495	813	2475	1932	5746	1449	1448	1047	1050	726	4424	2223	4318	147
sace_d	2483	15492	1935	3381	1099	1536	2281	4651	15491	1922	3613	155	3454	3452	314	sace_d_02679	877	2440	2611	23505	4369	468	23504	2191	12787	15506	0	0	0	0	0
sace_e	2483	15492	1935	3381	1099	1536	2281	4651	15491	1922	3613	155	3454	3452	314	sace_e_0500	877	2440	2611	23605	4369	4369	468	23606	2191	12787	15506	0	0	0	0
sace_e	2789	6047	2559	5906	3844	929	5173	1661	4682	145	15374	1205	493	5748	5747	sace_e_4257	190	4495	813	2475	1932	5746	1449	1448	1047	1050	726	4424	2223	4318	147
sapa	0	0	0	0	0	0	0	0	0	145	15374	1205	493	5748	5747	sapa_c170_16969	190	4495	813	2475	1932	5746	1449	1448	1047	1050	726	4424	2223	4318	147
sapa	24449	15492	1935	3381	1099	1536	2281	4651	15491	1922	3613	155	3454	3452	314	sapa_c214_2150	877	2440	2611	4369	24450	0	0	0	0	0	0	0	0	0	0
sami_a	0	0	0	0	0	0	0	0	0	0	0	0	493	5748	5747	sami_a_c1010_16957	190	4495	813	2475	1932	5746	1449	1448	1047	1050	726	4424	2223	4318	4318
sami_a	0	0	0	0	0	0	0	0	0	0	0	0	0	0	0	sami_a_c699_5023	877	2440	2611	2611	4369	24202	0	0	0	0	0	0	0	0	0
saba_a	0	2789	6047	2559	5906	3844	929	5173	1661	145	15374	1205	493	5748	5747	saba_a_556.17	0	0	0	0	0	0	0	0	0	0	0	0	0	0	0
saba_a	2483	15492	1935	3381	1099	1536	2281	4651	15491	1922	3613	155	3454	3452	314	saba_a_622.18	877	2611	5098	2737	12276	15409	0	0	0	0	0	0	0	0	0
saba_b	15889	15492	1935	3381	1099	1536	2281	4651	15491	1922	3613	155	3454	3452	314	saba_b_c596_2320	877	2611	5098	2737	12276	15409	0	0	0	0	0	0	0	0	0
saku	0	0	0	0	0	0	0	0	0	0	0	0	0	0	314	saku_c1455.3	0	0	0	0	0	0	0	0	0	0	0	0	0	0	0
saku	0	0	0	0	0	0	0	0	0	0	0	0	0	0	5747	saku_c1803.5	190	4495	813	0	0	0	0	0	0	0	0	0	0	0	0
saca	0	0	0	0	0	0	0	0	0	0	0	0	0	16300	5747	saca_660.3	190	4495	813	2475	1932	5746	1047	1050	2878	2182	16596	3344	4545	3046	2125
cagl	456	1755	75	75	75	5751	314	5750	3844	929	736	5749	1205	5748	5747	cagl0j01375g	190	4495	813	2475	1932	5746	1047	1050	5745	5744	3604	27	26	525	365
klpo	0	0	0	0	0	0	0	0	0	0	0	0	0	0	0	klpo_467.24	190	4495	2475	1932	5978	5746	1449	1448	1047	1048	1050	5745	877	879	5293
zyro	0	0	0	0	0	0	0	20450	20451	20450	20452	20450	20451	33	82	zyro0a00330g	340	3021	2729	5571	2811	3261	1943	6029	1109	4502	1857	2241	20453	4364	1111

zyro	875	2727	5071	3310	3309	485	535	1205	1205	147	502	493	20812	1893	5747	zyro0g03234g	190	813	2475	1932	5978	5746	1449	1448	20811	24930	1047	1048	1050	5745	696
zyro	1538	1070	1075	1074	10579	468	1386	1076	906	905	2506	418	908	1429	5794	zyro0g21868g	236	3238	20897	3017	3236	413	10578	5072	20898	20899	2090	57	841	1062	1064
zyro	1070	1075	1074	10579	468	1386	1076	906	905	2506	418	908	1429	5794	236	zyro0g21890g	3238	20897	3017	3236	413	10578	5072	20898	20899	2090	57	841	1062	1064	1925
sakl	5530	4454	5347	5954	2925	5953	5348	64	12591	2979	959	4019	683	17279	4024	sakl0a07480g	11020	5617	418	940	98	3574	301	5618	12540	17280	3224	5620	4612	4237	3213
sakl	5355	2806	3205	5355	5355	5356	1049	5977	1205	147	502	493	10236	1893	5747	sakl0e02992g	190	813	2475	1932	5978	5746	1449	1448	12295	1047	1048	1050	5745	3207	4430
klwa	0	0	0	0	0	0	0	0	0	0	0	0	0	2412	468	klwa_074-snap.3	242	0	0	0	0	0	0	0	0	0	0	0	0	0	0
klwa	145	12298	2806	3205	12676	12297	5355	5356	1049	1205	147	502	493	10236	1893	klwa_093-snap.33	190	813	2475	1932	5978	5746	1449	1448	12295	1047	1048	1050	5745	3207	4430
klwa	0	0	0	0	0	0	0	0	0	0	0	0	0	0	5794	klwa_305-snap.8	3238	12587	3017	3236	413	6138	2901	0	0	0	0	0	0	0	0
klth	12298	2806	3205	12297	5355	5356	1049	12296	1205	147	502	493	10236	1893	5747	klth0d05434g	190	813	2475	1932	5978	5746	1449	1448	12295	1047	1048	1050	5745	3207	4430
klth	0	0	0	0	0	0	242	2191	468	12169	33	465	1899	679	12413	klth0e16720g	199	22011	12412	485	340	841	2007	3254	2729	5571	2811	3261	1943	6029	1109
klth	1070	1075	1074	10579	468	1386	1076	906	905	2506	418	908	1429	1260	5794	klth0h05456g	3238	12587	3017	3236	413	2191	468	10578	5072	4556	535	3309	3310	5071	2727
klla	11428	1786	11429	11430	3038	4067	5356	1049	1205	147	502	493	11431	1893	5747	klla0f13684g	190	813	2475	1932	5978	5746	11432	5414	1052	1075	525	6061	4113	11433	348
ergo	10238	1271	710	147	1369	1046	10237	1049	1205	147	502	493	10236	1893	5747	ergo0b04004g	190	813	2475	1932	5978	5746	10235	1047	1048	1050	5745	3207	10234	4430	147
cagu	1552	6989	1398	1398	1511	1510	1509	1508	1506	6990	4289	4488	4488	6991	6991	cagu_04492	340	340	6992	4454	6755	2914	2914	82	6993	2194	6994	2899	677	677	4369
cagu	0	0	0	0	0	0	0	0	6524	340	7112	7113	1260	7113	7113	cagu_05180	7114	7115	280	896	280	340	605	3245	7116	468	662	4411	6373	6373	370
cagu	0	0	0	0	6524	340	7112	7113	1260	7113	7113	236	7114	7115	280	cagu_05184	280	340	605	3245	7116	468	662	4411	6373	6373	370	468	798	95	1796
deha	10013	3655	1075	1398	6734	7008	277	4377	2987	10014	4377	5874	340	4488	6991	deha2f27082g	1374	3112	10015	5874	2987	2037	10016	6068	10017	10018	10019	9393	9393	9762	9467
pist	0	0	0	0	0	0	0	0	0	0	0	465	14994	75	4488	pist_igi19987745	2121	14995	6530	2769	14996	1568	1569	4489	473	4492	147	4493	4494	1031	2850
pipa	14340	284	2679	3013	1271	14342	14343	407	369	14344	14345	1940	466	1566	14346	pipa_3g02865	3788	138	137	3361	3364	14347	130	25	4442	14348	14349	4020	1177	14350	1171

Homologs of VBA1 and VBA2 genes. The protein family classification of the adjacent genes is shown and the homologous neighbours are highlighted in the same colour.

sace_a	sace0b08690g	sace0b08712g	sace0b08734g	sace0b08756g	sace0b08778g	sace0b08800g	sace0b08822g	sace0b08844g	sace0b08866g	sace0b08888g	sace0b08910g	sace0b08932g	sace0b08954g	sace0b08976g	sace0b08998g	sace0b09020g	sace0b09042g	sace0b09064g	sace0b09086g	sace0b09108g	sace0b09130g	sace0b09152g	sace0b09174g	sace0b09196g	sace0b09218g	sace0b09240g	Null	Null	Null	Null	Null
sace_a	sace0m04862g	sace0m04884g	sace0m04906g	sace0m04928g	sace0m04950g	sace0m04994g	sace0m05016g	sace0m05038g	sace0m05060g	sace0m05082g	sace0m05104g	sace0m05126g	sace0m05148g	sace0m05170g	sace0m05192g	sace0m05214g	sace0m05236g	sace0m05258g	sace0m05280g	sace0m05302g	sace0m05324g	sace0m05346g	sace0m05368g	sace0m05390g	sace0m05412g	sace0m05434g	sace0m05478g	sace0m05500g	sace0m05522g	sace0m05544g	sace0m05566g
sace_b	sace_b_1b15_4621g	sace_b_1b15_4632g	sace_b_1b15_4643g	sace_b_1b15_4654g	sace_b_1b15_4665g	sace_b_1b15_4676g	sace_b_1b15_4687g	sace_b_1b15_4698g	sace_b_1b15_4709g	sace_b_1b15_4720g	sace_b_1b15_4731g	sace_b_1b15_4742g	sace_b_1b15_4753g	sace_b_1b15_4764g	sace_b_1b15_4775g	sace_b_1b15_4786g	sace_b_1b15_4797g	sace_b_1b15_4808g	sace_b_1b15_4819g	sace_b_1b15_4830g	sace_b_1b15_4841g	Null	Null	Null	Null	Null	Null	Null	Null	Null	Null
sace_b	sace_b_1m3_2399g	sace_b_1m3_2410g	sace_b_1m3_2421g	sace_b_1m3_2432g	sace_b_1m3_2443g	sace_b_1m3_2454g	sace_b_1m3_2465g	sace_b_1m3_2476g	sace_b_1m3_2487g	sace_b_1m3_2498g	sace_b_1m3_2509g	sace_b_1m3_2520g	sace_b_1m3_2531g	sace_b_1m3_2542g	sace_b_1m3_2564g	sace_b_1m3_2575g	sace_b_1m3_2586g	sace_b_1m3_2597g	sace_b_1m3_2608g	sace_b_1m3_2630g	sace_b_1m3_2641g	sace_b_1m3_2652g	sace_b_1m3_2663g	sace_b_1m3_2674g	sace_b_1m3_2685g	sace_b_1m3_2696g	sace_b_1m3_2707g	sace_b_1m3_2718g	sace_b_1m3_2729g	sace_b_1m3_2740g	sace_b_1m3_2751g
sace_c	sace_c_01023	sace_c_00978	sace_c_00972	sace_c_00950	sace_c_00988	sace_c_00941	sace_c_00947	sace_c_00962	sace_c_00930	sace_c_00986	sace_c_01028	sace_c_00946	sace_c_01026	sace_c_00984	sace_c_00970	sace_c_00976	sace_c_00979	sace_c_00955	sace_c_00939	sace_c_00982	sace_c_01033	sace_c_01009	sace_c_00938	sace_c_00960	sace_c_00977	sace_c_01031	Null	Null	Null	Null	Null
sace_c	sace_c_01610	sace_c_01604	sace_c_01560	sace_c_01592	sace_c_01572	sace_c_01585	sace_c_01589	sace_c_01603	sace_c_01622	sace_c_01626	sace_c_01601	sace_c_01583	sace_c_01557	sace_c_01599	sace_c_01564	sace_c_01581	sace_c_01618	sace_c_01591	sace_c_01619	sace_c_01628	sace_c_01555	sace_c_01600	Null	Null	Null	Null	Null	Null	Null	Null	Null
sace_d	sace_d_01963	sace_d_01964	sace_d_01965	sace_d_01966	sace_d_01967	sace_d_01968	sace_d_01969	sace_d_01970	sace_d_01971	sace_d_01972	sace_d_01973	sace_d_01974	sace_d_01975	sace_d_01976	sace_d_01977	sace_d_01978	sace_d_01979	sace_d_01980	sace_d_01981	sace_d_01982	sace_d_01983	sace_d_01984	sace_d_01985	sace_d_01986	sace_d_01987	sace_d_01988	sace_d_01989	sace_d_01990	sace_d_01991	sace_d_01992	sace_d_01993
sace_d	sace_d_02694	sace_d_02693	sace_d_02692	sace_d_02691	sace_d_02690	sace_d_02689	sace_d_02688	sace_d_02687	sace_d_02686	sace_d_02685	sace_d_02684	sace_d_02683	sace_d_02682	sace_d_02681	sace_d_02680	sace_d_02679	sace_d_02678	sace_d_02677	sace_d_02676	sace_d_02675	sace_d_02674	sace_d_02673	sace_d_02672	sace_d_02671	sace_d_02670	sace_d_02669	Null	Null	Null	Null	Null
sace_e	sace_e_0485	sace_e_0486	sace_e_0487	sace_e_0488	sace_e_0489	sace_e_0490	sace_e_0491	sace_e_0492	sace_e_0493	sace_e_0494	sace_e_0495	sace_e_0496	sace_e_0497	sace_e_0498	sace_e_0499	sace_e_0500	sace_e_0501	sace_e_0502	sace_e_0503	sace_e_0504	sace_e_0505	sace_e_0506	sace_e_0507	sace_e_0508	sace_e_0509	sace_e_0510	sace_e_0511	Null	Null	Null	Null
sace_e	sace_e_4242	sace_e_4243	sace_e_4244	sace_e_4245	sace_e_4246	sace_e_4247	sace_e_4248	sace_e_4249	sace_e_4250	sace_e_4251	sace_e_4252	sace_e_4253	sace_e_4254	sace_e_4255	sace_e_4256	sace_e_4257	sace_e_4258	sace_e_4259	sace_e_4260	sace_e_4261	sace_e_4262	sace_e_4263	sace_e_4264	sace_e_4265	sace_e_4266	sace_e_4267	sace_e_4268	sace_e_4269	sace_e_4270	sace_e_4271	sace_e_4272
sapa	Null	Null	Null	Null	Null	Null	Null	Null	Null	sapa_c170_17004	sapa_c170_16999	sapa_c170_16989	sapa_c170_16984	sapa_c170_16980	sapa_c170_16974	sapa_c170_16969	sapa_c170_16962	sapa_c170_16958	sapa_c170_16957	sapa_c170_16953	sapa_c170_16948	sapa_c170_16946	sapa_c170_16945	sapa_c170_16941	sapa_c170_16940	sapa_c170_16937	sapa_c170_16934	sapa_c170_16926	sapa_c170_16923	sapa_c170_16919	sapa_c170_16914
sapa	sapa_c214_2101	sapa_c214_2102	sapa_c214_2104	sapa_c214_2106	sapa_c214_2112	sapa_c214_2116	sapa_c214_2118	sapa_c214_2125	sapa_c214_2128	sapa_c214_2130	sapa_c214_2133	sapa_c214_2136	sapa_c214_2139	sapa_c214_2142	sapa_c214_2147	sapa_c214_2150	sapa_c214_2153	sapa_c214_2157	sapa_c214_2160	sapa_c214_2166	sapa_c214_2168	Null	Null	Null	Null	Null	Null	Null	Null	Null	Null
sami_a	Null	Null	Null	Null	Null	Null	Null	Null	Null	Null	Null	Null	sami_a_c1010_16969	sami_a_c1010_16963	sami_a_c1010_16960	sami_a_c1010_16957	sami_a_c1010_16953	sami_a_c1010_16950	sami_a_c1010_16947	sami_a_c1010_16944	sami_a_c1010_16943	sami_a_c1010_16939	sami_a_c1010_16938	sami_a_c1010_16935	sami_a_c1010_16932	sami_a_c1010_16929	sami_a_c1010_16926	sami_a_c1010_16920	sami_a_c1010_16918	sami_a_c1010_16916	sami_a_c1010_16914
sami_a	Null	Null	Null	Null	Null	Null	Null	Null	Null	Null	Null	Null	Null	Null	Null	sami_a_c699_5023	sami_a_c699_5028	sami_a_c699_5033	sami_a_c699_5041	sami_a_c699_5043	sami_a_c699_5049	sami_a_c699_5052	Null	Null	Null	Null	Null	Null	Null	Null	Null
saba_a	Null	saba_a_556.2	saba_a_556.3	saba_a_556.4	saba_a_556.5	saba_a_556.6	saba_a_556.7	saba_a_556.8	saba_a_556.9	saba_a_556.11	saba_a_556.12	saba_a_556.13	saba_a_556.14	saba_a_556.15	saba_a_556.16	saba_a_556.17	Null	Null	Null	Null	Null	Null	Null	Null	Null	Null	Null	Null	Null	Null	Null
saba_a	saba_a_622.2	saba_a_622.3	saba_a_622.4	saba_a_622.5	saba_a_622.6	saba_a_622.7	saba_a_622.8	saba_a_622.9	saba_a_622.10	saba_a_622.11	saba_a_622.12	saba_a_622.13	saba_a_622.14	saba_a_622.15	saba_a_622.16	saba_a_622.18	saba_a_622.19	saba_a_622.20	saba_a_622.22	saba_a_622.23	saba_a_622.24	saba_a_622.26	Null	Null	Null	Null	Null	Null	Null	Null	Null
saba_b	saba_b_c596_2257	saba_b_c596_2258	saba_b_c596_2261	saba_b_c596_2264	saba_b_c596_2269	saba_b_c596_2276	saba_b_c596_2277	saba_b_c596_2281	saba_b_c596_2289	saba_b_c596_2291	saba_b_c596_2296	saba_b_c596_2300	saba_b_c596_2303	saba_b_c596_2311	saba_b_c596_2313	saba_b_c596_2320	saba_b_c596_2323	saba_b_c596_2329	saba_b_c596_2333	saba_b_c596_2338	saba_b_c596_2349	saba_b_c596_2356	Null	Null	Null	Null	Null	Null	Null	Null	Null
saku	Null	Null	Null	Null	Null	Null	Null	Null	Null	Null	Null	Null	Null	Null	saku_c1455.2	saku_c1455.3	Null	Null	Null	Null	Null	Null	Null	Null	Null	Null	Null	Null	Null	Null	Null
saku	Null	Null	Null	Null	Null	Null	Null	Null	Null	Null	Null	Null	Null	Null	saku_c1803.6	saku_c1803.5	saku_c1803.4	saku_c1803.3	saku_c1803.2	Null	Null	Null	Null	Null	Null	Null	Null	Null	Null	Null	Null
saca	Null	Null	Null	Null	Null	Null	Null	Null	Null	Null	Null	Null	Null	saca_660.1	saca_660.2	saca_660.3	saca_660.4	saca_660.5	saca_660.6	saca_660.7	saca_660.8	saca_660.9	saca_660.10	saca_660.11	saca_660.12	saca_660.13	saca_ydr461c-a	saca_660.14	saca_660.15	saca_660.16	saca_660.17
cagl	cagl0j01870g	cagl0j01848g	cagl0j01800g	cagl0j01774g	cagl0j01727g	cagl0j01699g	cagl0j01661g	cagl0j01595g	cagl0j01529g	cagl0j01507g	cagl0j01485g	cagl0j01463g	cagl0j01441g	cagl0j01419g	cagl0j01397g	cagl0j01375g	cagl0j01353g	cagl0j01331g	cagl0j01309g	cagl0j01287g	cagl0j01265g	cagl0j01243g	cagl0j01221g	cagl0j01199g	cagl0j01177g	cagl0j01155g	cagl0j01111g	cagl0j01089g	cagl0j01067g	cagl0j01045g	cagl0j01023g
klpo	Null	Null	Null	Null	Null	Null	Null	Null	Null	Null	Null	Null	Null	Null	Null	klpo_467.24	klpo_467.23	klpo_467.22	klpo_467.21	klpo_467.20	klpo_467.19	klpo_467.18	klpo_467.17	klpo_467.16	klpo_467.15	klpo_467.14	klpo_467.13	klpo_467.12	klpo_467.11	klpo_467.10	klpo_467.9
zyro	Null	Null	Null	Null	Null	Null	Null	zyro0a00132g	zyro0a00154g	zyro0a00198g	zyro0a00220g	zyro0a00242g	zyro0a00264g	zyro0a00286g	zyro0a00308g	zyro0a00330g	zyro0a00374g	zyro0a00396g	zyro0a00440g	zyro0a00462g	zyro0a00484g	zyro0a00506g	zyro0a00528g	zyro0a00550g	zyro0a00572g	zyro0a00594g	zyro0a00616g	zyro0a00638g	zyro0a00682g	zyro0a00704g	zyro0a00726g
zyro	zyro0g03586g	zyro0g03564g	zyro0g03542g	zyro0g03520g	zyro0g03498g	zyro0g03476g	zyro0g03454g	zyro0g03410g	zyro0g03388g	zyro0g03366g	zyro0g03344g	zyro0g03322g	zyro0g03300g	zyro0g03278g	zyro0g03256g	zyro0g03234g	zyro0g03212g	zyro0g03190g	zyro0g03168g	zyro0g03146g	zyro0g03124g	zyro0g03102g	zyro0g03080g	zyro0g03058g	zyro0g03036g	zyro0g03014g	zyro0g02992g	zyro0g02970g	zyro0g02948g	zyro0g02926g	zyro0g02904g
zyro	zyro0g21538g	zyro0g21560g	zyro0g21582g	zyro0g21604g	zyro0g21626g	zyro0g21648g	zyro0g21670g	zyro0g21692g	zyro0g21714g	zyro0g21736g	zyro0g21758g	zyro0g21780g	zyro0g21802g	zyro0g21824g	zyro0g21846g	zyro0g21868g	zyro0g21890g	zyro0g21912g	zyro0g21934g	zyro0g21956g	zyro0g21978g	zyro0g22000g	zyro0g22022g	zyro0g22044g	zyro0g22110g	zyro0g22132g	zyro0g22154g	zyro0g22176g	zyro0g22198g	zyro0g22264g	zyro0g22286g
zyro	zyro0g21560g	zyro0g21582g	zyro0g21604g	zyro0g21626g	zyro0g21648g	zyro0g21670g	zyro0g21692g	zyro0g21714g	zyro0g21736g	zyro0g21758g	zyro0g21780g	zyro0g21802g	zyro0g21824g	zyro0g21846g	zyro0g21868g	zyro0g21890g	zyro0g21912g	zyro0g21934g	zyro0g21956g	zyro0g21978g	zyro0g22000g	zyro0g22022g	zyro0g22044g	zyro0g22110g	zyro0g22132g	zyro0g22154g	zyro0g22176g	zyro0g22198g	zyro0g22264g	zyro0g22286g	zyro0g22308g
sakl	sakl0a07106g	sakl0a07128g	sakl0a07150g	sakl0a07172g	sakl0a07194g	sakl0a07216g	sakl0a07238g	sakl0a07260g	sakl0a07282g	sakl0a07304g	sakl0a07326g	sakl0a07348g	sakl0a07370g	sakl0a07392g	sakl0a07414g	sakl0a07480g	sakl0a07502g	sakl0a07524g	sakl0a07546g	sakl0a07568g	sakl0a07612g	sakl0a07634g	sakl0a07656g	sakl0a07678g	sakl0a07700g	sakl0a07722g	sakl0a07744g	sakl0a07766g	sakl0a07788g	sakl0a07810g	sakl0a07832g
sakl	sakl0e02640g	sakl0e02662g	sakl0e02684g	sakl0e02706g	sakl0e02728g	sakl0e02772g	sakl0e02794g	sakl0e02816g	sakl0e02838g	sakl0e02860g	sakl0e02882g	sakl0e02904g	sakl0e02926g	sakl0e02948g	sakl0e02970g	sakl0e02992g	sakl0e03014g	sakl0e03036g	sakl0e03058g	sakl0e03080g	sakl0e03102g	sakl0e03124g	sakl0e03146g	sakl0e03168g	sakl0e03190g	sakl0e03212g	sakl0e03234g	sakl0e03256g	sakl0e03278g	sakl0e03300g	sakl0e03322g
klwa	Null	Null	Null	Null	Null	Null	Null	Null	Null	Null	Null	Null	Null	klwa_074-snap.1	klwa_074-snap.2	klwa_074-snap.3	klwa_074-snap.4	Null	Null	Null	Null	Null	Null	Null	Null	Null	Null	Null	Null	Null	Null
klwa	klwa_093-snap.48	klwa_093-snap.47	klwa_093-snap.46	klwa_093-snap.45	klwa_093-snap.44	klwa_093-snap.43	klwa_093-snap.42	klwa_093-snap.41	klwa_093-snap.40	klwa_093-snap.39	klwa_093-snap.38	klwa_093-snap.37	klwa_093-snap.36	klwa_093-snap.35	klwa_093-snap.34	klwa_093-snap.33	klwa_093-snap.32	klwa_093-snap.31	klwa_093-snap.30	klwa_093-snap.29	klwa_093-snap.28	klwa_093-snap.27	klwa_093-snap.26	klwa_093-snap.25	klwa_093-snap.24	klwa_093-snap.23	klwa_093-snap.22	klwa_093-snap.21	klwa_093-snap.20	klwa_093-snap.19	klwa_093-snap.18
klwa	Null	Null	Null	Null	Null	Null	Null	Null	Null	Null	Null	Null	Null	Null	klwa_305-snap.9	klwa_305-snap.8	klwa_305-snap.7	klwa_305-snap.6	klwa_305-snap.5	klwa_305-snap.4	klwa_305-snap.3	klwa_305-snap.2	klwa_305-snap.1	Null	Null	Null	Null	Null	Null	Null	Null
kith	kith0d05764g	kith0d05742g	kith0d05720g	kith0d05698g	kith0d05676g	kith0d05654g	kith0d05632g	kith0d05610g	kith0d05588g	kith0d05566g	kith0d05544g	kith0d05522g	kith0d05500g	kith0d05478g	kith0d05456g	kith0d05434g	kith0d05412g	kith0d05390g	kith0d05368g	kith0d05346g	kith0d05324g	kith0d05302g	kith0d05280g	kith0d05258g	kith0d05236g	kith0d05214g	kith0d05192g	kith0d05170g	kith0d05148g	kith0d05126g	kith0d05104g
kith	Null	Null	Null	Null	Null	Null	kith0e17138g	kith0e17006g	kith0e16984g	kith0e16874g	kith0e16852g	kith0e16808g	kith0e16786g	kith0e16764g	kith0e16742g	kith0e16720g	kith0e16676g	kith0e16654g	kith0e16632g	kith0e16610g	kith0e16588g	kith0e16566g	kith0e16544g	kith0e16522g	kith0e16500g	kith0e16478g	kith0e16456g	kith0e16434g	kith0e16412g	kith0e16390g	kith0e16368g
kith	kith0h05786g	kith0h05764g	kith0h05742g	kith0h05720g	kith0h05698g	kith0h05676g	kith0h05654g	kith0h05632g	kith0h05610g	kith0h05588g	kith0h05566g	kith0h05544g	kith0h05522g	kith0h05500g	kith0h05478g	kith0h05456g	kith0h05434g	kith0h05412g	kith0h05390g	kith0h05368g	kith0h05346g	kith0h05324g	kith0h05280g	kith0h05258g	kith0h05236g	kith0h05214g	kith0h05148g	kith0h05126g	kith0h05104g	kith0h05082g	kith0h05060g
klla	klla0f13354g	klla0f13376g	klla0f13398g	klla0f13420g	klla0f13442g	klla0f13464g	klla0f13486g	klla0f13508g	klla0f13530g	klla0f13552g	klla0f13574g	klla0f13596g	klla0f13618g	klla0f13640g	klla0f13662g	klla0f13684g	klla0f13706g	klla0f13728g	klla0f13750g	klla0f13772g	klla0f13794g	klla0f13816g	klla0f13838g	klla0f13860g	klla0f13882g	klla0f13904g	klla0f13926g	klla0f13948g	klla0f13970g	klla0f13981g	klla0f13992g
ergo	ergo0b04356g	ergo0b04334g	ergo0b04312g	ergo0b04290g	ergo0b04268g	ergo0b04246g	ergo0b04202g	ergo0b04180g	ergo0b04158g	ergo0b04136g	ergo0b04114g	ergo0b04092g	ergo0b04070g	ergo0b04048g	ergo0b04026g	ergo0b04004g	ergo0b03982g	ergo0b03960g	ergo0b03938g	ergo0b03916g	ergo0b03894g	ergo0b03872g	ergo0b03850g	ergo0b03828g	ergo0b03806g	ergo0b03784g	ergo0b03762g	ergo0b03740g	ergo0b03718g	ergo0b03696g	ergo0b03674g
cagu	cagu_04477	cagu_04478	cagu_04479	cagu_04480	cagu_04481	cagu_04482	cagu_04483	cagu_04484	cagu_04485	cagu_04486	cagu_04487	cagu_04488	cagu_04489	cagu_04490	cagu_04491	cagu_04492	cagu_04493	cagu_04494	cagu_04495	cagu_04496	cagu_04497	cagu_04498	cagu_04499	cagu_04500	cagu_04501	cagu_04502	cagu_04503	cagu_04504	cagu_04505	cagu_04506	cagu_04507
cagu	Null	Null	Null	Null	Null	Null	Null	Null	cagu_05173	cagu_05174	cagu_05175	cagu_05176	cagu_05177	cagu_05178	cagu_05179	cagu_05180	cagu_05181	cagu_05182	cagu_05183	cagu_05184	cagu_05185	cagu_05186	cagu_05187	cagu_05188	cagu_05189	cagu_05190	cagu_05191	cagu_05192	cagu_05193	cagu_05194	cagu_05195
cagu	Null	Null	Null	Null	cagu_05173	cagu_05174	cagu_05175	cagu_05176	cagu_05177	cagu_05178	cagu_05179	cagu_05180	cagu_05181	cagu_05182	cagu_05183	cagu_05184	cagu_05185	cagu_05186	cagu_05187	cagu_05188	cagu_05189	cagu_05190	cagu_05191	cagu_05192	cagu_05193	cagu_05194	cagu_05195	cagu_05196	cagu_05197	cagu_05198	cagu_05199
deha	deha2f26730g	deha2f26752g	deha2f26774g	deha2f26796g	deha2f26818g	deha2f26840g	deha2f26862g	deha2f26884g	deha2f26906g	deha2f26950g	deha2f26972g	deha2f26994g	deha2f27016g	deha2f27038g	deha2f27060g	deha2f27082g	deha2f27104g	deha2f27126g	deha2f27170g	deha2f27192g	deha2f27214g	deha2f27236g	deha2f27258g	deha2f27280g	deha2f27302g	deha2f27324g	deha2f27346g	deha2f27368g	deha2f27390g	deha2f27412g	deha2f27434g
pist	Null	Null	Null	Null	Null	Null	Null	Null	Null	Null	Null	pist_igi19141289	pist_igi16604988	pist_igi19141290	pist_igi20836448	pist_igi19987745	pist_igi19987746	pist_igi16604989	pist_igi16604990	pist_igi16604991	pist_igi16604992	pist_igi16604993	pist_igi19141291	pist_igi19141292	pist_igi16604994	pist_igi19141293	pist_igi20836449	pist_igi19141294	pist_igi19141295	pist_igi16604995	pist_igi20836450
pipa	pipa_3g02785	pipa_3g02790	pipa_3g02795	pipa_3g02800	pipa_3g02810	pipa_3g02815	pipa_3g02820	pipa_3g02825	pipa_3g02830	pipa_3g02835	pipa_3g02840	pipa_3g02845	pipa_3g02850	pipa_3g02855	pipa_3g02860	pipa_3g02865	pipa_3g02870	pipa_3g02875	pipa_3g02880	pipa_3g02885	pipa_3g02890	pipa_3g02895	pipa_3g02900	pipa_3g02905	pipa_3g02910	pipa_3g02915	pipa_3g02920	pipa_3g02925	pipa_3g02930	pipa_3g02935	pipa_3g02940

Homologs of VBA1 and VBA2 genes. The names of the adjacent genes are shown and the homologous neighbours are highlighted in the same colour.

Cluster D (lineage 3): homologs of VBA4 gene

sace_a	1503	6105	869	2035	15306	1300	2040	547	5017	1833	15331	15332	3358	2567	5019	sace0d07920g	16630	1682	5021	147	15421	10767	1557	1684	2679	1663	3267	10765	5680	1303	57
sace_b	869	2035	15306	1300	2040	547	5017	1833	16819	15331	16820	15332	3358	5019	23095	sace_b_1d0_3730g	1682	5021	147	15421	10767	1557	1684	2679	1663	3267	10765	5680	1303	23096	23097
sace_c	0	0	0	0	0	0	0	547	5017	1833	15331	15332	3358	2567	5019	sace_c_00011	16630	1682	5021	147	15421	10767	1557	1684	2679	1663	3267	10765	5680	1303	57
sace_d	1503	6105	869	2035	15306	1300	2040	547	5017	1833	15331	15332	3358	2567	5019	sace_d_00400	16630	1682	5021	147	15421	10767	1557	1684	2679	1663	3267	5680	1303	23439	57
sace_e	1503	6105	869	2035	15306	1300	2040	547	5017	1833	15331	15332	3358	2567	5019	sace_e_1018	16630	1682	5021	147	15421	10767	1557	1684	2679	1663	3267	5680	1303	23623	57
sapa	0	0	0	0	0	0	0	0	0	0	0	0	0	0	0	sapa_c122_4166	1682	5021	147	15421	10767	1557	1684	2679	1663	1663	3267	10765	5680	1303	24374
sapa	6105	869	2035	15306	1300	2040	547	5017	1833	17765	15331	15332	3358	2567	5019	sapa_c123_4162	0	0	0	0	0	0	0	0	0	0	0	0	0	0	0
sami_a	0	0	0	0	0	0	0	0	0	0	0	0	0	0	5019	sami_a_c1083_3691	0	0	0	0	0	0	0	0	0	0	0	0	0	0	0
saba_b	0	0	0	0	0	0	0	0	0	0	0	0	0	0	0	saba_b_c216_4256	1682	5021	0	0	0	0	0	0	0	0	0	0	0	0	0
saba_b	0	0	1300	2040	547	5017	5017	1833	15781	15331	15332	3358	2567	5019	236	saba_b_c217_4260	0	0	0	0	0	0	0	0	0	0	0	0	0	0	0
saba_b	0	0	0	1300	2040	547	5017	5017	1833	15781	15331	15332	3358	2567	5019	saba_b_c217_4261	236	0	0	0	0	0	0	0	0	0	0	0	0	0	0
saku	0	0	0	0	0	0	0	0	5017	1833	15331	15332	3358	2567	5019	saku_c1927.9	0	0	0	0	0	0	0	0	0	0	0	0	0	0	0
klpo	5924	4646	11644	12118	12119	1438	1300	978	3356	5017	1833	1820	12120	12121	877	klpo_543.44	12130	1682	5021	5058	2639	1557	12122	3267	1294	5680	21983	147	5016	5014	1606
zyro	547	978	1213	4692	3356	5017	1833	1820	20562	10772	20561	3358	2567	5019	877	zyro0c01430g	2961	2637	2584	2583	6342	2594	1274	6343	402	1857	2633	5779	2219	4983	518
sakl	749	5834	3889	5012	2694	3955	3952	3940	2194	12229	17361	3164	2191	468	17362	sakl0c02178g	3962	3088	57	116	1211	3927	10686	1221	1412	17363	90	873	2704	354	17364
sakl	2555	1508	2454	146	4115	248	5148	5147	12292	147	692	382	17472	3566	3565	sakl0e04246g	1893	3807	2846	2853	17473	23759	5914	2104	5915	418	836	54	826	824	834
sakl	978	5218	1213	4692	3356	5017	1833	1820	17663	10772	17662	3358	2567	5019	877	sakl0h16808g	17661	1682	5021	5058	912	5057	5056	147	10766	2639	10767	4165	1192	1557	17660
klwa	0	0	0	0	0	0	0	0	0	0	12337	12275	1260	340	11165	klwa_073-snap.6	4170	468	0	0	0	0	0	0	0	0	0	0	0	0	0
klwa	0	0	0	0	0	3356	5017	1833	1820	12536	10772	3358	2567	5019	877	klwa_281-snap.8	1682	5021	5058	912	5057	5056	147	0	0	0	0	0	0	0	0
klth	547	978	1213	4692	3356	5017	1833	1820	12536	10772	12535	3358	2567	5019	877	klth0g13464g	22024	1682	5021	5058	912	5057	5056	147	10766	2639	10767	4165	1557	12534	12533
klla	2245	1597	10856	10857	10858	5340	4237	4612	3224	10859	10860	5618	301	10861	10862	klla0a04631g	3958	1062	1925	190	5199	4938	849	851	857	843	177	845	2090	377	379
klla	280	2987	5425	2341	1703	1730	11452	1722	1606	2385	5670	314	11451	3566	3565	klla0f17776g	1893	3807	2846	2853	11450	21900	11449	11448	2104	5915	418	836	54	826	824

ergo	978	10774	1213	4692	3356	5017	1833	1820	10773	10772	10771	3358	2567	5019	877	ergo0g10076g	1682	2674	1294	6313	758	10770	3267	1662	1663	2679	1684	10769	10768	1557	4165
caal_a	1782	1784	251	3	250	249	248	247	245	244	243	242	241	240	237	caal_a_19.1308	235	234	233	232	231	230	229	228	226	225	224	47	2927	2928	2930
caal_b	1782	1784	251	3	250	249	248	247	245	244	243	242	241	240	237	caal_b_03443	235	234	233	232	231	230	229	228	226	4775	224	224	47	2927	2928
cadu	237	240	241	242	243	244	245	247	248	249	250	251	1784	1782	4897	cadu_43440	235	234	233	232	231	230	229	228	226	225	224	47	2927	2928	2930
catr	243	1270	1267	1266	1265	1265	1264	8893	440	230	241	240	237	8901	8900	catr_00329	235	234	233	232	231	229	228	226	225	224	47	2927	2928	2930	2931
catr	245	244	243	1270	1267	1266	1265	1265	1264	8893	440	230	241	240	237	catr_00331	8900	236	235	234	233	232	231	229	228	226	225	224	47	2927	2928
capa	0	0	0	251	248	249	250	245	244	243	8	242	241	240	237	capa_01507	235	234	82	7000	232	233	82	231	229	228	226	225	8480	2931	2930
loel	2487	1203	2488	2489	2490	2491	2492	2492	2493	2495	2688	2687	241	240	237	loel_04525	235	234	233	232	7000	231	13269	228	226	225	13270	13271	2931	2930	2928
cagu	1243	1242	349	347	803	1248	6679	340	340	250	243	244	124	912	340	cagu_04556	235	2412	3564	662	291	2744	340	477	234	7001	240	233	232	7000	231
deha	10115	10114	1321	1247	1244	1243	1242	349	347	803	10113	1248	250	243	244	deha2g17402g	235	234	237	240	241	233	232	7000	231	10112	228	226	225	224	47
pist	607	1777	1778	187	15006	248	249	250	245	244	243	242	241	240	15114	pist_igi16605957	236	235	234	233	232	7000	231	15232	228	226	225	224	47	178	633
pist	1777	1778	187	15006	248	249	250	245	244	243	242	241	240	15114	236	pist_igi18293126	235	234	233	232	7000	231	15232	228	226	225	224	47	178	633	4362
calu	7362	7363	3257	3256	3887	3886	3885	3890	1242	349	347	803	7364	243	244	calu_00809	235	234	7365	241	233	232	7000	244	2710	2709	1407	3086	3727	4294	915
pipa	2765	13493	2762	2766	2480	1607	1608	1580	13496	7160	13497	13498	2281	13500	13501	pipa_1g01100	2417	2418	13502	500	501	1561	22168	22169	280	2822	6248	2247	2071	2070	444
pipa	14669	2600	2601	22261	1265	1264	631	1490	1489	1488	3324	14671	14673	147	3579	pipa_4g02960	178	14675	3562	3570	3757	3758	1933	14676	3002	85	3006	3005	442	3963	14677
yali	41	1205	19747	3278	243	19748	19749	1069	2673	4037	1749	19750	2299	1664	19751	yali0e18095g	1659	407	19752	19753	19754	1192	597	1705	1075	1052	262	127	3807	19755	3259

Homologs of VBA4 gene. The protein family classification of the adjacent genes is shown and the homologous neighbours are highlighted in the same colour.

sace_a	sace0d07568g	sace0d07590g	sace0d07612g	sace0d07634g	sace0d07656g	sace0d07700g	sace0d07722g	sace0d07744g	sace0d07766g	sace0d07788g	sace0d07810g	sace0d07832g	sace0d07854g	sace0d07876g	sace0d07898g	sace0d07920g	sace0d07942g	sace0d07964g	sace0d07986g	sace0d08008g	sace0d08030g	sace0d08052g	sace0d08074g	sace0d08096g	sace0d08118g	sace0d08140g	sace0d08162g	sace0d08184g	sace0d08206g	sace0d08228g	sace0d08250g
sace_b	sace_b_1d0_3554g	sace_b_1d0_3565g	sace_b_1d0_3576g	sace_b_1d0_3587g	sace_b_1d0_3598g	sace_b_1d0_3609g	sace_b_1d0_3620g	sace_b_1d0_3631g	sace_b_1d0_3642g	sace_b_1d0_3653g	sace_b_1d0_3664g	sace_b_1d0_3675g	sace_b_1d0_3686g	sace_b_1d0_3708g	sace_b_1d0_3719g	sace_b_1d0_3730g	sace_b_1d0_3752g	sace_b_1d0_3763g	sace_b_1d0_3774g	sace_b_1d0_3785g	sace_b_1d0_3796g	sace_b_1d0_3807g	sace_b_1d0_3818g	sace_b_1d0_3829g	sace_b_1d0_3840g	sace_b_1d0_3851g	sace_b_1d0_3862g	sace_b_1d0_3873g	sace_b_1d0_3884g	sace_b_1d0_3895g	sace_b_1d0_3906g
sace_c	Null	Null	Null	Null	Null	Null	Null	sace_c_00008	sace_c_00029	sace_c_00026	sace_c_00009	sace_c_00042	sace_c_00035	sace_c_00025	sace_c_00024	sace_c_00011	sace_c_00043	sace_c_00017	sace_c_00023	sace_c_00020	sace_c_00030	sace_c_00016	sace_c_00039	sace_c_00040	sace_c_00021	sace_c_00036	sace_c_00010	sace_c_00037	sace_c_00041	sace_c_00038	sace_c_00033
sace_d	sace_d_00415	sace_d_00414	sace_d_00413	sace_d_00412	sace_d_00411	sace_d_00410	sace_d_00409	sace_d_00408	sace_d_00407	sace_d_00406	sace_d_00405	sace_d_00404	sace_d_00403	sace_d_00402	sace_d_00401	sace_d_00400	sace_d_00399	sace_d_00398	sace_d_00397	sace_d_00396	sace_d_00395	sace_d_00394	sace_d_00393	sace_d_00392	sace_d_00391	sace_d_00390	sace_d_05699	sace_d_00387	sace_d_00386	sace_d_00385	sace_d_00384
sace_e	sace_e_1003	sace_e_1004	sace_e_1005	sace_e_1006	sace_e_1007	sace_e_1008	sace_e_1009	sace_e_1010	sace_e_1011	sace_e_1012	sace_e_1013	sace_e_1014	sace_e_1015	sace_e_1016	sace_e_1017	sace_e_1018	sace_e_1019	sace_e_1020	sace_e_1021	sace_e_1022	sace_e_1023	sace_e_1024	sace_e_1025	sace_e_1026	sace_e_1027	sace_e_1028	sace_e_1029	sace_e_1030	sace_e_1031	sace_e_1032	sace_e_1033
sapa	Null	Null	Null	Null	Null	Null	Null	Null	Null	Null	Null	Null	Null	Null	Null	sapa_c122_4166	sapa_c122_4172	sapa_c122_4175	sapa_c122_4177	sapa_c122_4185	sapa_c122_4189	sapa_c122_4192	sapa_c122_4194	sapa_c122_4195	sapa_c122_4207	sapa_c122_4211	sapa_c122_4212	sapa_c122_4217	sapa_c122_4221	sapa_c122_4225	sapa_c122_4228
sapa	sapa_c123_4111	sapa_c123_4115	sapa_c123_4120	sapa_c123_4123	sapa_c123_4126	sapa_c123_4130	sapa_c123_4133	sapa_c123_4138	sapa_c123_4142	sapa_c123_4146	sapa_c123_4147	sapa_c123_4150	sapa_c123_4152	sapa_c123_4154	sapa_c123_4158	sapa_c123_4162	Null	Null	Null	Null	Null	Null	Null	Null	Null	Null	Null	Null	Null	Null	Null
sami_a	Null	Null	Null	Null	Null	Null	Null	Null	Null	Null	Null	Null	Null	Null	sami_a_c1083_3696	sami_a_c1083_3691	Null	Null	Null	Null	Null	Null	Null	Null	Null	Null	Null	Null	Null	Null	Null
saba_b	Null	Null	Null	Null	Null	Null	Null	Null	Null	Null	Null	Null	Null	Null	Null	saba_b_c216_4256	saba_b_c216_4250	saba_b_c216_4248	Null	Null	Null	Null	Null	Null	Null	Null	Null	Null	Null	Null	Null
saba_b	Null	Null	saba_b_c217_4298	saba_b_c217_4293	saba_b_c217_4288	saba_b_c217_4285	saba_b_c217_4284	saba_b_c217_4280	saba_b_c217_4279	saba_b_c217_4277	saba_b_c217_4275	saba_b_c217_4274	saba_b_c217_4266	saba_b_c217_4264	saba_b_c217_4261	saba_b_c217_4260	Null	Null	Null	Null	Null	Null	Null	Null	Null	Null	Null	Null	Null	Null	Null
saba_b	Null	Null	Null	saba_b_c217_4298	saba_b_c217_4293	saba_b_c217_4288	saba_b_c217_4285	saba_b_c217_4284	saba_b_c217_4280	saba_b_c217_4279	saba_b_c217_4277	saba_b_c217_4275	saba_b_c217_4274	saba_b_c217_4266	saba_b_c217_4264	saba_b_c217_4261	saba_b_c217_4260	Null	Null	Null	Null	Null	Null	Null	Null	Null	Null	Null	Null	Null	Null
saku	Null	Null	Null	Null	Null	Null	Null	Null	saku_c1927.2	saku_c1927.3	saku_c1927.4	saku_c1927.5	saku_c1927.6	saku_c1927.7	saku_c1927.8	saku_c1927.9	Null	Null	Null	Null	Null	Null	Null	Null	Null	Null	Null	Null	Null	Null	Null
klpo	klpo_543.29	klpo_543.30	klpo_543.31	klpo_543.32	klpo_543.33	klpo_543.34	klpo_543.35	klpo_543.36	klpo_543.37	klpo_543.38	klpo_543.39	klpo_543.40	klpo_543.41	klpo_543.42	klpo_543.43	klpo_543.44	klpo_ydr119w-a	klpo_543.45	klpo_543.46	klpo_543.47	klpo_543.48	klpo_543.49	klpo_543.50	klpo_543.51	klpo_543.52	klpo_543.53	klpo_543.54	klpo_543.55	klpo_543.56	klpo_543.57	klpo_543.58
zyro	zyro0c01782g	zyro0c01738g	zyro0c01716g	zyro0c01694g	zyro0c01672g	zyro0c01650g	zyro0c01628g	zyro0c01606g	zyro0c01584g	zyro0c01562g	zyro0c01540g	zyro0c01518g	zyro0c01496g	zyro0c01474g	zyro0c01452g	zyro0c01430g	zyro0c01408g	zyro0c01320g	zyro0c01298g	zyro0c01276g	zyro0c01254g	zyro0c01232g	zyro0c01210g	zyro0c01188g	zyro0c01166g	zyro0c01144g	zyro0c01100g	zyro0c01078g	zyro0c01056g	zyro0c01034g	zyro0c01012g
sakl	sakl0c01826g	sakl0c01848g	sakl0c01870g	sakl0c01892g	sakl0c01914g	sakl0c01936g	sakl0c01958g	sakl0c02002g	sakl0c02024g	sakl0c02046g	sakl0c02068g	sakl0c02090g	sakl0c02112g	sakl0c02134g	sakl0c02156g	sakl0c02178g	sakl0c02200g	sakl0c02222g	sakl0c02244g	sakl0c02266g	sakl0c02288g	sakl0c02310g	sakl0c02332g	sakl0c02354g	sakl0c02376g	sakl0c02398g	sakl0c02420g	sakl0c02442g	sakl0c02464g	sakl0c02486g	sakl0c02508g
sakl	sakl0e03872g	sakl0e03894g	sakl0e03916g	sakl0e03938g	sakl0e03960g	sakl0e04004g	sakl0e04026g	sakl0e04048g	sakl0e04070g	sakl0e04092g	sakl0e04114g	sakl0e04136g	sakl0e04158g	sakl0e04202g	sakl0e04224g	sakl0e04246g	sakl0e04268g	sakl0e04312g	sakl0e04334g	sakl0e04356g	sakl0e04378g	sakl0e04400g	sakl0e04422g	sakl0e04444g	sakl0e04466g	sakl0e04488g	sakl0e04510g	sakl0e04532g	sakl0e04554g	sakl0e04576g	sakl0e04598g
sakl	sakl0h17138g	sakl0h17116g	sakl0h17094g	sakl0h17072g	sakl0h17050g	sakl0h17028g	sakl0h17006g	sakl0h16984g	sakl0h16962g	sakl0h16940g	sakl0h16918g	sakl0h16896g	sakl0h16874g	sakl0h16852g	sakl0h16830g	sakl0h16808g	sakl0h16786g	sakl0h16764g	sakl0h16742g	sakl0h16720g	sakl0h16698g	sakl0h16676g	sakl0h16654g	sakl0h16632g	sakl0h16610g	sakl0h16588g	sakl0h16566g	sakl0h16544g	sakl0h16522g	sakl0h16500g	sakl0h16478g
klwa	Null	Null	Null	Null	Null	Null	Null	Null	Null	Null	klwa_073-snap.1	klwa_073-snap.2	klwa_073-snap.3	klwa_073-snap.4	klwa_073-snap.5	klwa_073-snap.6	klwa_073-snap.7	klwa_073-snap.8	Null	Null	Null	Null	Null	Null	Null	Null	Null	Null	Null	Null	Null
klwa	Null	Null	Null	Null	Null	klwa_281-snap.18	klwa_281-snap.17	klwa_281-snap.16	klwa_281-snap.15	klwa_281-snap.14	klwa_281-snap.13	klwa_281-snap.12	klwa_281-snap.11	klwa_281-snap.10	klwa_281-snap.9	klwa_281-snap.8	klwa_281-snap.7	klwa_281-snap.6	klwa_281-snap.5	klwa_281-snap.4	klwa_281-snap.3	klwa_281-snap.2	klwa_281-snap.1	Null	Null	Null	Null	Null	Null	Null	Null
kith	kith0g13794g	kith0g13772g	kith0g13750g	kith0g13728g	kith0g13706g	kith0g13684g	kith0g13662g	kith0g13640g	kith0g13618g	kith0g13596g	kith0g13574g	kith0g13552g	kith0g13530g	kith0g13508g	kith0g13486g	kith0g13464g	kith0g13442g	kith0g13420g	kith0g13398g	kith0g13376g	kith0g13354g	kith0g13332g	kith0g13310g	kith0g13288g	kith0g13266g	kith0g13244g	kith0g13222g	kith0g13200g	kith0g13178g	kith0g13156g	kith0g13134g
klla	klla0a04235g	klla0a04301g	klla0a04323g	klla0a04345g	klla0a04367g	klla0a04389g	klla0a04411g	klla0a04433g	klla0a04499g	klla0a04521g	klla0a04543g	klla0a04565g	klla0a04587g	klla0a04609g	klla0a04620g	klla0a04631g	klla0a04653g	klla0a04708g	klla0a04730g	klla0a04752g	klla0a04774g	klla0a04796g	klla0a04818g	klla0a04840g	klla0a04862g	klla0a04884g	klla0a04906g	klla0a04928g	klla0a04961g	klla0a04983g	klla0a05005g
klla	klla0f18106g	klla0f18084g	klla0f18062g	klla0f18040g	klla0f18018g	klla0f17996g	klla0f17974g	klla0f17952g	klla0f17930g	klla0f17908g	klla0f17886g	klla0f17864g	klla0f17842g	klla0f17820g	klla0f17798g	klla0f17776g	klla0f17754g	klla0f17677g	klla0f17655g	klla0f17633g	klla0f17611g	klla0f17589g	klla0f17567g	klla0f17545g	klla0f17523g	klla0f17501g	klla0f17479g	klla0f17457g	klla0f17435g	klla0f17413g	klla0f17391g
ergo	ergo0g10406g	ergo0g10384g	ergo0g10362g	ergo0g10340g	ergo0g10318g	ergo0g10296g	ergo0g10274g	ergo0g10252g	ergo0g10230g	ergo0g10208g	ergo0g10186g	ergo0g10164g	ergo0g10142g	ergo0g10120g	ergo0g10098g	ergo0g10076g	ergo0g10054g	ergo0g10032g	ergo0g09988g	ergo0g09966g	ergo0g09944g	ergo0g09922g	ergo0g09900g	ergo0g09878g	ergo0g09856g	ergo0g09834g	ergo0g09812g	ergo0g09790g	ergo0g09768g	ergo0g09746g	ergo0g09724g
caal_a	caal_a_19.3378	caal_a_19.3380	caal_a_19.1327	caal_a_19.1326	caal_a_19.1325	caal_a_19.1324	caal_a_19.1323	caal_a_19.1321	caal_a_19.1318	caal_a_19.1317	caal_a_19.1314	caal_a_19.1313	caal_a_19.1311	caal_a_19.1310	caal_a_19.1309	caal_a_19.1308	caal_a_19.1307	caal_a_19.1306	caal_a_19.1305	caal_a_19.1304	caal_a_19.1303	caal_a_19.1302	caal_a_19.1301	caal_a_19.1300	caal_a_19.1299	caal_a_19.1298	caal_a_19.1297	caal_a_19.5037	caal_a_19.5038	caal_a_19.5039	caal_a_19.5040
caal_b	caal_b_03458	caal_b_03457	caal_b_03456	caal_b_03455	caal_b_03454	caal_b_03453	caal_b_03452	caal_b_03451	caal_b_03450	caal_b_03449	caal_b_03448	caal_b_03447	caal_b_03446	caal_b_03445	caal_b_03444	caal_b_03443	caal_b_03442	caal_b_03441	caal_b_03440	caal_b_03439	caal_b_03438	caal_b_03437	caal_b_03436	caal_b_03435	caal_b_03434	caal_b_03433	caal_b_03432	caal_b_03431	caal_b_03430	caal_b_03429	caal_b_03428
cadu	cadu_43290	cadu_43300	cadu_43310	cadu_43320	cadu_43330	cadu_43340	cadu_43350	cadu_43360	cadu_43370	cadu_43380	cadu_43390	cadu_43400	cadu_43420	cadu_43425	cadu_43430	cadu_43440	cadu_43450	cadu_43460	cadu_43470	cadu_43480	cadu_43490	cadu_43500	cadu_43510	cadu_43520	cadu_43530	cadu_43540	cadu_43550	cadu_43560	cadu_43570	cadu_43580	cadu_43590
catr	catr_00344	catr_00343	catr_00342	catr_00341	catr_00340	catr_00339	catr_00338	catr_00337	catr_00336	catr_00335	catr_00334	catr_00333	catr_00332	catr_00331	catr_00330	catr_00329	catr_00328	catr_00327	catr_00326	catr_00325	catr_00324	catr_00323	catr_00322	catr_00321	catr_00320	catr_00319	catr_00318	catr_00317	catr_00316	catr_00315	catr_00314
catr	catr_00346	catr_00345	catr_00344	catr_00343	catr_00342	catr_00341	catr_00340	catr_00339	catr_00338	catr_00337	catr_00336	catr_00335	catr_00334	catr_00333	catr_00332	catr_00331	catr_00330	catr_00329	catr_00328	catr_00327	catr_00326	catr_00325	catr_00324	catr_00323	catr_00322	catr_00321	catr_00320	catr_00319	catr_00318	catr_00317	catr_00316
capa	Null	Null	Null	capa_01519	capa_01518	capa_01517	capa_01516	capa_01515	capa_01514	capa_01513	capa_01512	capa_01511	capa_01510	capa_01509	capa_01508	capa_01507	capa_01506	capa_01505	capa_01504	capa_01503	capa_01502	capa_01501	capa_01500	capa_01499	capa_01498	capa_01497	capa_01496	capa_01495	capa_01494	capa_01493	capa_01492
loel	loel_04510	loel_04511	loel_04512	loel_04513	loel_04514	loel_04515	loel_04516	loel_04517	loel_04518	loel_04519	loel_04520	loel_04521	loel_04522	loel_04523	loel_04524	loel_04525	loel_04526	loel_04527	loel_04528	loel_04529	loel_04530	loel_04531	loel_04532	loel_04533	loel_04534	loel_04535	loel_04536	loel_04537	loel_04538	loel_04539	loel_04540
cagu	cagu_04571	cagu_04570	cagu_04569	cagu_04568	cagu_04567	cagu_04566	cagu_04565	cagu_04564	cagu_04563	cagu_04562	cagu_04561	cagu_04560	cagu_04559	cagu_04558	cagu_04557	cagu_04556	cagu_04555	cagu_04554	cagu_04553	cagu_04552	cagu_04551	cagu_04550	cagu_04549	cagu_04548	cagu_04547	cagu_04546	cagu_04545	cagu_04544	cagu_04543	cagu_04542	cagu_04541
deha	deha2g17776g	deha2g17754g	deha2g17732g	deha2g17710g	deha2g17688g	deha2g17666g	deha2g17644g	deha2g17622g	deha2g17600g	deha2g17578g	deha2g17556g	deha2g17534g	deha2g17512g	deha2g17468g	deha2g17446g	deha2g17402g	deha2g17380g	deha2g17358g	deha2g17336g	deha2g17314g	deha2g17292g	deha2g17270g	deha2g17248g	deha2g17226g	deha2g17204g	deha2g17182g	deha2g17160g	deha2g17138g	deha2g17116g	deha2g17094g	deha2g17072g
pist	pist_igi16605948	pist_igi19141556	pist_igi19988011	pist_igi18293124	pist_igi16605949	pist_igi16605950	pist_igi16605951	pist_igi20836702	pist_igi16605952	pist_igi16605953	pist_igi16605954	pist_igi16605955	pist_igi16605956	pist_igi20836703	pist_igi18293125	pist_igi16605957	pist_igi18293126	pist_igi16605958	pist_igi20836704	pist_igi20836705	pist_igi16605959	pist_igi19141557	pist_igi16605960	pist_igi19988012	pist_igi18293127	pist_igi18293128	pist_igi16605961	pist_igi18293129	pist_igi16605962	pist_igi16605963	pist_igi19141558
pist	pist_igi19141556	pist_igi19988011	pist_igi18293124	pist_igi16605949	pist_igi16605950	pist_igi16605951	pist_igi20836702	pist_igi16605952	pist_igi16605953	pist_igi16605954	pist_igi16605955	pist_igi16605956	pist_igi20836703	pist_igi18293125	pist_igi16605957	pist_igi18293126	pist_igi16605958	pist_igi20836704	pist_igi20836705	pist_igi16605959	pist_igi19141557	pist_igi16605960	pist_igi19988012	pist_igi18293127	pist_igi18293128	pist_igi16605961	pist_igi18293129	pist_igi16605962	pist_igi16605963	pist_igi19141558	pist_igi18293130
calu	calu_00794	calu_00795	calu_00796	calu_00797	calu_00798	calu_00799	calu_00800	calu_00801	calu_00802	calu_00803	calu_00804	calu_00805	calu_00806	calu_00807	calu_00808	calu_00809	calu_00810	calu_00811	calu_00812	calu_00813	calu_00814	calu_00815	calu_00816	calu_00817	calu_00818	calu_00819	calu_00820	calu_00821	calu_00822	calu_00823	calu_00824
pipa	pipa_1g01005	pipa_1g01010	pipa_1g01015	pipa_1g01020	pipa_1g01025	pipa_1g01035	pipa_1g01040	pipa_1g01050	pipa_1g01055	pipa_1g01060	pipa_1g01065	pipa_1g01070	pipa_1g01075	pipa_1g01085	pipa_1g01095	pipa_1g01100	pipa_1g01105	pipa_1g01110	pipa_1g01115	pipa_1g01120	pipa_1g01125	pipa_1g01130	pipa_1g01135	pipa_1g01140	pipa_1g01145	pipa_1g01150	pipa_1g01155	pipa_1g01160	pipa_1g01165	pipa_1g01170	pipa_1g01175
pipa	pipa_4g02855	pipa_4g02860	pipa_4g02865	pipa_4g02870	pipa_4g02875	pipa_4g02880	pipa_4g02885	pipa_4g02890	pipa_4g02895	pipa_4g02900	pipa_4g02905	pipa_4g02915	pipa_4g02925	pipa_4g02945	pipa_4g02950	pipa_4g02960	pipa_4g02965	pipa_4g02970	pipa_4g02975	pipa_4g02980	pipa_4g02985	pipa_4g02990	pipa_4g02995	pipa_4g03000	pipa_4g03005	pipa_4g03010	pipa_4g03015	pipa_4g03020	pipa_4g03025	pipa_4g03030	pipa_4g03035
yali	yali0e17765g	yali0e17787g	yali0e17809g	yali0e17831g	yali0e17853g	yali0e17875g	yali0e17897g	yali0e17919g	yali0e17941g	yali0e17963g	yali0e17985g	yali0e18007g	yali0e18029g	yali0e18051g	yali0e18073g	yali0e18095g	yali0e18117g	yali0e18139g	yali0e18161g	yali0e18194g	yali0e18216g	yali0e18238g	yali0e18260g	yali0e18282g	yali0e18304g	yali0e18326g	yali0e18348g	yali0e18414g	yali0e18436g	yali0e18458g	yali0e18480g

Homologs of VBA4 gene. The names of the adjacent genes are shown and the homologous neighbours are highlighted in the same colour.

Cluster E (lineage 4): homologs of ATR1 gene and ORF YMR279C

Sheet1

sace_a	15506	1586	2811	22984	4364	1111	4366	633	178	3872	1937	1785	15451	3256	3225	sace0m00462g	1940	10224	15366	5818	2633	1912	2077	5817	15886	1170	1171	1177	2757	845	15307
sace_a	5519	241	1921	1175	1176	5302	1170	2076	23002	2077	2078	2633	3131	3997	3993	sace0m09768g	2967	2833	6212	3985	99	3256	10654	1941	1250	3430	525	147	2185	2171	10265
sace_b	2811	23217	4364	1111	4366	633	178	3872	17086	1937	1785	15451	3256	3225	17087	sace_b_1m3_0188g	1940	10224	15366	5818	1912	2077	5817	15886	1170	1171	1177	2757	845	17088	15307
sace_b	5519	241	1921	1175	1176	1170	2076	23237	23238	2077	2078	2633	3131	3997	3993	sace_b_1m3_4896g	2967	2833	6212	3985	99	3256	10654	1941	1250	3430	525	17113	147	2185	2171
sace_c	0	0	0	0	0	0	0	2076	23387	2077	2078	2633	3131	3997	3993	sace_c_03767	2967	2833	6212	3985	99	3256	10654	1941	1250	3430	525	147	2185	2171	10265
sace_c	0	1586	2811	23400	4364	1111	4366	633	178	3872	1937	1785	15451	3256	3225	sace_c_04366	1940	10224	15366	5818	2633	1912	2077	5817	15886	1170	1171	1177	2757	845	15307
sace_d	15506	1586	2811	23479	4364	1111	4366	633	178	3872	1937	1785	15451	3256	3225	sace_d_01783	1940	10224	15366	5818	2633	1912	2077	5817	15886	1170	1171	2757	845	15307	1516
sace_d	5519	241	1921	1175	1176	5302	1170	2076	23495	2077	2078	2633	3131	3997	3993	sace_d_02175	2967	2833	6212	3985	99	3256	10654	1941	1250	3430	525	147	2185	2171	10265
sace_e	15506	1586	2811	23690	4364	1111	4366	633	178	3872	1937	1785	15451	3256	3225	sace_e_4060	1940	10224	15366	5818	2633	1912	2077	5817	15886	1170	1171	1177	2757	845	15307
sace_e	5519	241	1921	1175	1176	5302	1170	2076	23708	2077	2078	2633	3131	3997	3993	sace_e_4461	2967	2833	6212	3985	99	3256	10654	1941	1250	3430	525	147	2185	2171	10265
sapa	0	0	0	0	0	0	0	2076	2077	2078	2078	2633	3131	3997	3993	sapa_c419_17887	2967	2833	6212	3985	99	3256	10654	1941	1250	3430	525	147	2171	10265	1403
sapa	1586	2811	24679	4364	1111	4366	633	178	3872	1937	1785	15451	3256	3225	18095	sapa_c443_17778	1940	10224	15366	5818	2633	1912	2077	5817	15886	1170	1171	1177	2757	845	15307
sami_a	0	0	0	0	0	0	0	0	2076	2077	2078	2633	3131	3997	3993	sami_a_c468_17485	2967	2833	6212	3985	0	0	0	0	0	0	0	0	0	0	0
sami_a	0	0	0	0	1111	4366	633	178	3872	1937	1785	15451	3256	3225	17882	sami_a_c551_15970	1940	10224	15366	5818	2633	0	0	0	0	0	0	0	0	0	0
saba_a	15452	12416	1586	2811	22351	4364	1111	633	178	3872	1937	1785	15451	3256	3225	saba_a_604.18	1940	10224	0	0	0	0	0	0	0	0	0	0	0	0	0
saba_a	5521	5520	5519	241	1921	1175	5302	1170	2076	2077	2078	2633	3131	3997	3993	saba_a_671.27	2967	2833	6212	3985	99	3256	10654	1941	1250	3430	525	147	2185	2171	10265
saba_b	12416	1586	2811	22607	4364	1111	4366	633	178	3872	1937	1785	15451	3256	3225	saba_b_c553_18284	1940	10224	15366	5818	2633	1912	2077	5817	15886	1170	1171	1177	2757	0	0
saba_b	0	0	0	0	0	0	0	0	0	0	0	0	0	0	3993	saba_b_c753_19471	2967	2833	6212	3985	99	3256	10654	1941	1250	3430	0	0	0	0	0
saku	1516	3998	5521	5520	5519	241	1921	1175	5302	1170	2077	2078	2633	3131	3993	saku_c2070.4	2967	2833	0	0	0	0	0	0	0	0	0	0	0	0	0
saca	0	0	0	0	22791	3561	5128	458	453	22792	16053	450	3502	5130	4701	saca_551.4	1909	5129	2023	5132	3800	3796	0	0	0	0	0	0	0	0	0
saca	0	4502	1857	22833	4364	1111	633	178	3872	1937	1785	16394	10654	3256	3225	saca_692.14	1940	10224	16395	5818	16396	1580	5094	16397	5095	5096	147	3165	143	2760	16186
saca	5519	241	1145	1921	1175	1176	5302	1170	2076	2078	2633	2633	3131	3997	3993	saca_696.43	2967	2833	6212	5883	3985	0	0	0	0	0	0	0	0	0	0

cagl	4070	5030	101	1200	2064	4364	1111	633	178	3872	1937	1785	5029	3256	3225	cagl0b02343g	1940	5028	5027	5026	4158	1712	4464	4466	5025	5024	626	236	5023	3267	1663
cagl	1075	332	3868	6209	4593	4596	6210	1262	4156	9	3345	6211	3131	3997	3993	cagl0m03003g	2967	2833	6212	3848	3849	3301	4146	4315	978	3094	489	6213	6214	21270	6215
klpo	418	6138	5739	5740	12109	4364	4364	1111	5684	4366	178	3872	1936	1937	1785	klpo_541.16	1940	12107	5818	2633	1912	2077	2076	1171	5302	2757	5519	5521	3143	391	12108
sakl	3799	992	4092	1044	1057	17376	17377	5680	6242	2282	10342	2818	5678	5677	2829	sakl0c06116g	17378	17379	1193	17380	399	147	359	8	430	525	1735	1736	1737	921	3534
sakl	5817	2077	2078	17419	1912	2633	5818	17418	3131	10224	3997	3993	2097	1940	696	sakl0d01386g	3225	2967	2833	6212	3985	17417	407	17398	99	3256	10654	17416	4939	1941	1785
klwa	0	0	0	0	0	0	0	0	0	0	0	0	0	0	1940	klwa_042-snap.2	3225	0	0	0	0	0	0	0	0	0	0	0	0	0	0
klth	1170	12238	2076	5817	2077	2078	2633	5818	12237	3131	10224	3997	3993	2097	1940	klth0c03806g	3225	2967	2833	6212	3985	1941	12236	12235	3256	99	5629	456	700	12234	1810
caal_a	1375	1382	1390	1397	1415	1423	1368	1349	1343	1334	1472	1481	1487	1498	1507	caal_a_19.304	1521	1530	1540	254	340	1557	1579	1587	1595	905	1609	1618	1625	1293	1638
caal_b	1423	1415	1397	1390	1382	1375	1368	1349	1343	1334	4752	1481	1487	1498	1507	caal_b_02627	1521	1530	1540	254	340	1557	4753	1579	1899	1906	1899	124	1881	1873	1868
cadu	1368	1375	1382	1390	1397	1415	1423	1368	1349	1343	1334	1481	1487	1498	1507	cadu_83050	1521	1530	1540	254	1557	1587	1595	905	1609	1618	1625	1293	1638	21207	1255
catr	1423	1415	1397	1135	1135	242	1390	1382	1375	1368	1349	1481	1487	21499	9095	catr_02586	1521	1530	1540	9097	254	1346	1347	1579	1587	1595	905	1609	1618	1625	1293
cagu	4702	322	1213	1213	6657	6658	4692	6659	1820	33	4689	4686	819	820	82	cagu_02196	1540	1540	1530	1776	1783	6660	3502	6661	4152	4150	4650	3538	3537	468	3536
deha	453	452	451	450	449	9720	447	446	444	9721	1507	21672	5874	2987	5874	deha2d11572g	1540	1530	1767	1776	1783	6660	9722	3502	6661	4152	9723	4150	4650	3538	3537
pist	147	4135	262	1348	1618	905	696	15182	1587	1579	1557	82	15247	1507	662	pist_igi20837975	1530	1540	254	277	1783	1776	1767	1756	6649	14827	14827	2133	2132	1256	15062
calu	7843	4692	4692	7842	7841	7840	82	7839	1820	4689	4686	819	820	1040	1507	calu_03396	1540	1530	1767	1776	1783	1411	1480	6838	1620	3404	3409	7838	191	2517	7157

Homologs of ATR1 gene and of ORF YR279C. The protein family classification of the adjacent genes is shown and the homologous neighbours are highlighted in the same colour.

sace_a	sace0m00132g	sace0m00154g	sace0m00176g	sace0m00198g	sace0m00220g	sace0m00242g	sace0m00264g	sace0m00286g	sace0m00308g	sace0m00330g	sace0m00352g	sace0m00374g	sace0m00396g	sace0m00418g	sace0m00440g	sace0m00462g	sace0m00484g	sace0m00506g	sace0m00528g	sace0m00550g	sace0m00572g	sace0m00594g	sace0m00616g	sace0m00638g	sace0m00660g	sace0m00682g	sace0m00704g	sace0m00726g	sace0m00748g	sace0m00792g	sace0m00814g
sace_a	sace0m09416g	sace0m09438g	sace0m09460g	sace0m09482g	sace0m09504g	sace0m09526g	sace0m09548g	sace0m09592g	sace0m09614g	sace0m09636g	sace0m09658g	sace0m09680g	sace0m09702g	sace0m09724g	sace0m09746g	sace0m09768g	sace0m09790g	sace0m09812g	sace0m09834g	sace0m09856g	sace0m09900g	sace0m09922g	sace0m09944g	sace0m09966g	sace0m09988g	sace0m10010g	sace0m10032g	sace0m10054g	sace0m10076g	sace0m10098g	sace0m10120g
sace_b	sace_b_1m3_0023g	sace_b_1m3_0034g	sace_b_1m3_0045g	sace_b_1m3_0056g	sace_b_1m3_0067g	sace_b_1m3_0078g	sace_b_1m3_0089g	sace_b_1m3_0100g	sace_b_1m3_0111g	sace_b_1m3_0122g	sace_b_1m3_0133g	sace_b_1m3_0144g	sace_b_1m3_0155g	sace_b_1m3_0166g	sace_b_1m3_0177g	sace_b_1m3_0188g	sace_b_1m3_0199g	sace_b_1m3_0210g	sace_b_1m3_0221g	sace_b_1m3_0232g	sace_b_1m3_0254g	sace_b_1m3_0265g	sace_b_1m3_0276g	sace_b_1m3_0287g	sace_b_1m3_0298g	sace_b_1m3_0309g	sace_b_1m3_0320g	sace_b_1m3_0331g	sace_b_1m3_0342g	sace_b_1m3_0353g	sace_b_1m3_0364g
sace_b	sace_b_1m3_4720g	sace_b_1m3_4731g	sace_b_1m3_4742g	sace_b_1m3_4753g	sace_b_1m3_4764g	sace_b_1m3_4786g	sace_b_1m3_4797g	sace_b_1m3_4808g	sace_b_1m3_4819g	sace_b_1m3_4830g	sace_b_1m3_4841g	sace_b_1m3_4852g	sace_b_1m3_4863g	sace_b_1m3_4874g	sace_b_1m3_4885g	sace_b_1m3_4896g	sace_b_1m3_4907g	sace_b_1m3_4918g	sace_b_1m3_4929g	sace_b_1m3_4940g	sace_b_1m3_4951g	sace_b_1m3_4962g	sace_b_1m3_4973g	sace_b_1m3_4984g	sace_b_1m3_4995g	sace_b_1m3_5006g	sace_b_1m3_5017g	sace_b_1m3_5028g	sace_b_1m3_5039g	sace_b_1m3_5050g	sace_b_1m3_5061g
sace_c	Null	Null	Null	Null	Null	Null	Null	sace_c_03752	sace_c_03773	sace_c_03742	sace_c_03745	sace_c_03758	sace_c_03753	sace_c_03772	sace_c_03765	sace_c_03767	sace_c_03757	sace_c_03754	sace_c_03764	sace_c_03761	sace_c_03746	sace_c_03748	sace_c_03756	sace_c_03771	sace_c_03770	sace_c_03768	sace_c_03750	sace_c_03759	sace_c_03743	sace_c_03769	sace_c_03760
sace_c	Null	sace_c_04383	sace_c_04395	sace_c_04375	sace_c_04388	sace_c_04389	sace_c_04399	sace_c_04377	sace_c_04361	sace_c_04362	sace_c_04372	sace_c_04374	sace_c_04369	sace_c_04364	sace_c_04386	sace_c_04366	sace_c_04387	sace_c_04401	sace_c_04391	sace_c_04400	sace_c_04370	sace_c_04384	sace_c_04363	sace_c_04390	sace_c_04392	sace_c_04376	sace_c_04385	sace_c_04393	sace_c_04365	sace_c_04397	sace_c_04373
sace_d	sace_d_01768	sace_d_01769	sace_d_01770	sace_d_01771	sace_d_01772	sace_d_01773	sace_d_01774	sace_d_01775	sace_d_01776	sace_d_01777	sace_d_01778	sace_d_01779	sace_d_01780	sace_d_01781	sace_d_01782	sace_d_01783	sace_d_01784	sace_d_01785	sace_d_01786	sace_d_01787	sace_d_01788	sace_d_01789	sace_d_01790	sace_d_01791	sace_d_01792	sace_d_01793	sace_d_01794	sace_d_01796	sace_d_01797	sace_d_01798	sace_d_01800
sace_d	sace_d_02160	sace_d_02161	sace_d_02162	sace_d_02163	sace_d_02164	sace_d_02165	sace_d_02166	sace_d_02167	sace_d_02168	sace_d_02169	sace_d_02170	sace_d_02171	sace_d_02172	sace_d_02173	sace_d_02174	sace_d_02175	sace_d_02176	sace_d_02177	sace_d_02178	sace_d_02179	sace_d_02181	sace_d_02182	sace_d_02183	sace_d_02184	sace_d_02185	sace_d_02186	sace_d_02187	sace_d_02188	sace_d_02189	sace_d_02190	sace_d_02191
sace_e	sace_e_4045	sace_e_4046	sace_e_4047	sace_e_4048	sace_e_4049	sace_e_4050	sace_e_4051	sace_e_4052	sace_e_4053	sace_e_4054	sace_e_4055	sace_e_4056	sace_e_4057	sace_e_4058	sace_e_4059	sace_e_4060	sace_e_4061	sace_e_4062	sace_e_4063	sace_e_4064	sace_e_4065	sace_e_4066	sace_e_4067	sace_e_4068	sace_e_4069	sace_e_4070	sace_e_4071	sace_e_4072	sace_e_4073	sace_e_4074	sace_e_4075
sace_e	sace_e_4446	sace_e_4447	sace_e_4448	sace_e_4449	sace_e_4450	sace_e_4451	sace_e_4452	sace_e_4453	sace_e_4454	sace_e_4455	sace_e_4456	sace_e_4457	sace_e_4458	sace_e_4459	sace_e_4460	sace_e_4461	sace_e_4462	sace_e_4463	sace_e_4464	sace_e_4465	sace_e_4466	sace_e_4467	sace_e_4468	sace_e_4469	sace_e_4470	sace_e_4471	sace_e_4472	sace_e_4473	sace_e_4474	sace_e_4475	sace_e_4476
sapa	Null	Null	Null	Null	Null	Null	Null	sapa_c419_17852	sapa_c419_17856	sapa_c419_17862	sapa_c419_17864	sapa_c419_17867	sapa_c419_17874	sapa_c419_17879	sapa_c419_17884	sapa_c419_17887	sapa_c419_17891	sapa_c419_17898	sapa_c419_17900	sapa_c419_17904	sapa_c419_17907	sapa_c419_17911	sapa_c419_17915	sapa_c419_17919	sapa_c419_17921	sapa_c419_17925	sapa_c419_17930	sapa_c419_17933	sapa_c419_17941	sapa_c419_17947	sapa_c419_17949
sapa	sapa_c443_17832	sapa_c443_17828	sapa_c443_17826	sapa_c443_17822	sapa_c443_17818	sapa_c443_17814	sapa_c443_17813	sapa_c443_17809	sapa_c443_17805	sapa_c443_17801	sapa_c443_17794	sapa_c443_17788	sapa_c443_17786	sapa_c443_17782	sapa_c443_17781	sapa_c443_17778	sapa_c443_17776	sapa_c443_17773	sapa_c443_17771	sapa_c443_17769	sapa_c443_17759	sapa_c443_17757	sapa_c443_17752	sapa_c443_17750	sapa_c443_17744	sapa_c443_17742	sapa_c443_17741	sapa_c443_17736	sapa_c443_17728	sapa_c443_17724	sapa_c443_17723
sami_a	Null	Null	Null	Null	Null	Null	Null	Null	sami_a_c468_17450	sami_a_c468_17454	sami_a_c468_17465	sami_a_c468_17468	sami_a_c468_17474	sami_a_c468_17479	sami_a_c468_17483	sami_a_c468_17485	sami_a_c468_17487	sami_a_c468_17492	sami_a_c468_17495	sami_a_c468_17497	Null	Null	Null	Null	Null	Null	Null	Null	Null	Null	Null
sami_a	Null	Null	Null	Null	sami_a_c551_15930	sami_a_c551_15934	sami_a_c551_15938	sami_a_c551_15940	sami_a_c551_15944	sami_a_c551_15947	sami_a_c551_15950	sami_a_c551_15954	sami_a_c551_15959	sami_a_c551_15962	sami_a_c551_15967	sami_a_c551_15970	sami_a_c551_15975	sami_a_c551_15977	sami_a_c551_15981	sami_a_c551_15983	sami_a_c551_15987	Null	Null	Null	Null	Null	Null	Null	Null	Null	Null
saba_a	saba_a_604.2	saba_a_604.3	saba_a_604.4	saba_a_604.5	saba_a_604.6	saba_a_604.7	saba_a_604.8	saba_a_604.10	saba_a_604.11	saba_a_604.12	saba_a_604.13	saba_a_604.14	saba_a_604.15	saba_a_604.16	saba_a_604.17	saba_a_604.18	saba_a_604.19	saba_a_604.20	Null	Null	Null	Null	Null	Null	Null	Null	Null	Null	Null	Null	Null
saba_a	saba_a_671.43	saba_a_671.42	saba_a_671.41	saba_a_671.40	saba_a_671.39	saba_a_671.38	saba_a_671.36	saba_a_671.35	saba_a_671.34	saba_a_671.33	saba_a_671.32	saba_a_671.31	saba_a_671.30	saba_a_671.29	saba_a_671.28	saba_a_671.27	saba_a_671.26	saba_a_671.25	saba_a_671.24	saba_a_671.23	saba_a_671.22	saba_a_671.21	saba_a_671.20	saba_a_671.19	saba_a_671.18	saba_a_671.17	saba_a_671.16	saba_a_671.14	saba_a_671.13	saba_a_671.12	saba_a_671.11
saba_b	saba_b_c553_18357	saba_b_c553_18349	saba_b_c553_18343	saba_b_c553_18338	saba_b_c553_18335	saba_b_c553_18330	saba_b_c553_18328	saba_b_c553_18326	saba_b_c553_18321	saba_b_c553_18316	saba_b_c553_18309	saba_b_c553_18300	saba_b_c553_18294	saba_b_c553_18291	saba_b_c553_18287	saba_b_c553_18284	saba_b_c553_18281	saba_b_c553_18278	saba_b_c553_18273	saba_b_c553_18269	saba_b_c553_18263	saba_b_c553_18260	saba_b_c553_18253	saba_b_c553_18250	saba_b_c553_18247	saba_b_c553_18245	saba_b_c553_18242	saba_b_c553_18236	saba_b_c553_18231	Null	Null
saba_b	Null	Null	Null	Null	Null	Null	Null	Null	Null	Null	Null	Null	Null	Null	saba_b_c753_19476	saba_b_c753_19471	saba_b_c753_19463	saba_b_c753_19457	saba_b_c753_19453	saba_b_c753_19450	saba_b_c753_19447	saba_b_c753_19442	saba_b_c753_19440	saba_b_c753_19436	saba_b_c753_19431	saba_b_c753_19427	Null	Null	Null	Null	Null
saku	saku_c2070.22	saku_c2070.21	saku_c2070.20	saku_c2070.19	saku_c2070.18	saku_c2070.17	saku_c2070.16	saku_c2070.15	saku_c2070.13	saku_c2070.12	saku_c2070.10	saku_c2070.9	saku_c2070.8	saku_c2070.7	saku_c2070.5	saku_c2070.4	saku_c2070.3	saku_c2070.2	Null	Null	Null	Null	Null	Null	Null	Null	Null	Null	Null	Null	Null
saca	Null	Null	Null	Null	saca_513.0d	saca_513.1	saca_513.2	saca_513.3	saca_513.4	saca_513.5	saca_513.5d	saca_513.6	saca_551.1	saca_551.2	saca_551.3	saca_551.4	saca_551.5	saca_551.6	saca_551.7	saca_551.8	saca_551.9	saca_208.1	Null	Null	Null	Null	Null	Null	Null	Null	Null
saca	Null	saca_692.1	saca_692.2	saca_692.3	saca_692.4	saca_692.5	saca_692.6	saca_692.7	saca_692.8	saca_692.9	saca_692.10	saca_692.10d	saca_692.11	saca_692.12	saca_692.13	saca_692.14	saca_692.15	saca_692.16	saca_692.17	saca_692.18	saca_692.19	saca_692.20	saca_692.21	saca_692.21d	saca_692.22	saca_692.23	saca_692.24	saca_692.25	saca_692.26	saca_692.27	saca_692.28
saca	saca_696.28	saca_696.29	saca_696.30	saca_696.31	saca_696.32	saca_696.33	saca_696.34	saca_696.35	saca_696.36	saca_696.37	saca_696.38	saca_696.39	saca_696.40	saca_696.41	saca_696.42	saca_696.43	saca_696.44	saca_696.45	saca_696.46	saca_696.47	saca_696.48	Null	Null	Null	Null	Null	Null	Null	Null	Null	Null
cagl	cagl0b02673g	cagl0b02651g	cagl0b02629g	cagl0b02607g	cagl0b02585g	cagl0b02563g	cagl0b02541g	cagl0b02519g	cagl0b02497g	cagl0b02475g	cagl0b02453g	cagl0b02431g	cagl0b02409g	cagl0b02387g	cagl0b02365g	cagl0b02343g	cagl0b02321g	cagl0b02299g	cagl0b02277g	cagl0b02255g	cagl0b02233g	cagl0b02211g	cagl0b02189g	cagl0b02167g	cagl0b02145g	cagl0b02123g	cagl0b02101g	cagl0b02079g	cagl0b02057g	cagl0b02035g	cagl0b02013g
cagl	cagl0m02651g	cagl0m02673g	cagl0m02695g	cagl0m02717g	cagl0m02739g	cagl0m02761g	cagl0m02783g	cagl0m02805g	cagl0m02849g	cagl0m02871g	cagl0m02893g	cagl0m02915g	cagl0m02937g	cagl0m02959g	cagl0m02981g	cagl0m03003g	cagl0m03025g	cagl0m03047g	cagl0m03069g	cagl0m03091g	cagl0m03113g	cagl0m03135g	cagl0m03157g	cagl0m03179g	cagl0m03201g	cagl0m03223g	cagl0m03245g	cagl0m03267g	cagl0m03289g	cagl0m03305g	cagl0m03311g
klpo	klpo_541.31	klpo_541.30	klpo_541.29	klpo_541.28	klpo_541.27	klpo_541.26	klpo_541.25	klpo_541.24	klpo_541.23	klpo_541.22	klpo_541.21	klpo_541.20	klpo_541.19	klpo_541.18	klpo_541.17	klpo_541.16	klpo_541.15	klpo_541.14	klpo_541.13	klpo_541.12	klpo_541.11	klpo_541.10	klpo_541.9	klpo_541.8	klpo_541.7	klpo_541.6	klpo_541.5	klpo_541.4	klpo_541.3	klpo_541.1	klpo_541.2
sakl	sakl0c05720g	sakl0c05742g	sakl0c05764g	sakl0c05786g	sakl0c05808g	sakl0c05830g	sakl0c05852g	sakl0c05896g	sakl0c05918g	sakl0c05940g	sakl0c06006g	sakl0c06028g	sakl0c06050g	sakl0c06072g	sakl0c06094g	sakl0c06116g	sakl0c06138g	sakl0c06160g	sakl0c06182g	sakl0c06204g	sakl0c06226g	sakl0c06248g	sakl0c06270g	sakl0c06292g	sakl0c06314g	sakl0c06336g	sakl0c06358g	sakl0c06380g	sakl0c06402g	sakl0c06424g	sakl0c06446g
sakl	sakl0d01738g	sakl0d01716g	sakl0d01694g	sakl0d01672g	sakl0d01628g	sakl0d01606g	sakl0d01584g	sakl0d01562g	sakl0d01540g	sakl0d01518g	sakl0d01496g	sakl0d01474g	sakl0d01452g	sakl0d01430g	sakl0d01408g	sakl0d01386g	sakl0d01364g	sakl0d01342g	sakl0d01320g	sakl0d01298g	sakl0d01276g	sakl0d01254g	sakl0d01232g	sakl0d01210g	sakl0d01188g	sakl0d01166g	sakl0d01144g	sakl0d01122g	sakl0d01100g	sakl0d01078g	sakl0d01056g
klwa	Null	Null	Null	Null	Null	Null	Null	Null	Null	Null	Null	Null	Null	Null	klwa_042-snap.3	klwa_042-snap.2	klwa_042-snap.1	Null	Null	Null	Null	Null	Null	Null	Null	Null	Null	Null	Null	Null	Null
klth	klth0c04158g	klth0c04136g	klth0c04114g	klth0c04070g	klth0c04048g	klth0c04026g	klth0c04004g	klth0c03982g	klth0c03960g	klth0c03938g	klth0c03916g	klth0c03894g	klth0c03872g	klth0c03850g	klth0c03828g	klth0c03806g	klth0c03784g	klth0c03762g	klth0c03740g	klth0c03718g	klth0c03696g	klth0c03674g	klth0c03652g	klth0c03630g	klth0c03608g	klth0c03586g	klth0c03564g	klth0c03542g	klth0c03520g	klth0c03498g	klth0c03476g
caal_a	caal_a_19.285	caal_a_19.286	caal_a_19.287	caal_a_19.288	caal_a_19.290	caal_a_19.291	caal_a_19.292	caal_a_19.295	caal_a_19.296	caal_a_19.297	caal_a_19.298	caal_a_19.299	caal_a_19.300	caal_a_19.301	caal_a_19.302	caal_a_19.304	caal_a_19.305	caal_a_19.306	caal_a_19.307	caal_a_19.308	caal_a_19.309	caal_a_19.310	caal_a_19.313	caal_a_19.314	caal_a_19.315	caal_a_19.316	caal_a_19.317	caal_a_19.318	caal_a_19.319	caal_a_19.320	caal_a_19.321
caal_b	caal_b_02612	caal_b_02613	caal_b_02614	caal_b_02615	caal_b_02616	caal_b_02617	caal_b_02618	caal_b_02619	caal_b_02620	caal_b_02621	caal_b_02622	caal_b_02623	caal_b_02624	caal_b_02625	caal_b_02626	caal_b_02627	caal_b_02628	caal_b_02629	caal_b_02630	caal_b_02631	caal_b_02632	caal_b_02633	caal_b_02634	caal_b_02635	caal_b_02636	caal_b_02637	caal_b_02638	caal_b_02639	caal_b_02640	caal_b_02641	caal_b_02642
cadu	cadu_82890	cadu_82900	cadu_82910	cadu_82920	cadu_82930	cadu_82940	cadu_82950	cadu_82960	cadu_82970	cadu_82980	cadu_82990	cadu_83010	cadu_83020	cadu_83030	cadu_83040	cadu_83050	cadu_83060	cadu_83070	cadu_83080	cadu_83090	cadu_83100	cadu_83110	cadu_83120	cadu_83130	cadu_83140	cadu_83150	cadu_83160	cadu_83180	cadu_83190	cadu_83195	cadu_83200
catr	catr_02571	catr_02572	catr_02573	catr_02574	catr_02575	catr_02576	catr_02577	catr_02578	catr_02579	catr_02580	catr_02581	catr_02582	catr_02583	catr_02584	catr_02585	catr_02586	catr_02587	catr_02588	catr_02589	catr_02590	catr_02591	catr_02592	catr_02593	catr_02594	catr_02595	catr_02596	catr_02597	catr_02598	catr_02599	catr_02600	catr_02601
cagu	cagu_02181	cagu_02182	cagu_02183	cagu_02184	cagu_02185	cagu_02186	cagu_02187	cagu_02188	cagu_02189	cagu_02190	cagu_02191	cagu_02192	cagu_02193	cagu_02194	cagu_02195	cagu_02196	cagu_02197	cagu_02198	cagu_02199	cagu_02200	cagu_02201	cagu_02202	cagu_02203	cagu_02204	cagu_02205	cagu_02206	cagu_02207	cagu_02208	cagu_02209	cagu_02210	cagu_02211
deha	deha2d11242g	deha2d11264g	deha2d11286g	deha2d11308g	deha2d11330g	deha2d11352g	deha2d11374g	deha2d11396g	deha2d11418g	deha2d11440g	deha2d11462g	deha2d11484g	deha2d11506g	deha2d11528g	deha2d11550g	deha2d11572g	deha2d11594g	deha2d11616g	deha2d11638g	deha2d11660g	deha2d11682g	deha2d11704g	deha2d11726g	deha2d11748g	deha2d11770g	deha2d11792g	deha2d11814g	deha2d11836g	deha2d11858g	deha2d11880g	deha2d11902g
pist	pist_igi16612221	pist_igi18294605	pist_igi16612220	pist_igi16612219	pist_igi16612218	pist_igi20838222	pist_igi16612217	pist_igi19143081	pist_igi18294604	pist_igi16612216	pist_igi19989565	pist_igi16612215	pist_igi19989564	pist_igi19143080	pist_igi16612214	pist_igi20837975	pist_igi18294386	pist_igi19989318	pist_igi19989317	pist_igi16611220	pist_igi19142825	pist_igi19142824	pist_igi16611219	pist_igi19142823	pist_igi16611218	pist_igi16611217	pist_igi19989316	pist_igi16611216	pist_igi19142822	pist_igi16611215	pist_igi16611214
calu	calu_03411	calu_03410	calu_03406	calu_03409	calu_03408	calu_03407	calu_03405	calu_03404	calu_03403	calu_03402	calu_03401	calu_03400	calu_03399	calu_03398	calu_03397	calu_03396	calu_03395	calu_03394	calu_03393	calu_03392	calu_03391	calu_03390	calu_03389	calu_03388	calu_03387	calu_03386	calu_03385	calu_03384	calu_03383	calu_03381	calu_03382

Homologs of ATR1 gene and of ORF YR279C. The names of the adjacent genes are shown and the homologous neighbours are highlighted in the same colour.

Cluster F (lineage 5): homologs of ORF YOR378W

sace_a	3898	1857	2987	640	5821	1966	4022	4020	5823	5408	4026	677	2686	23032	5194	sace0o12012g	12270	199	23033	23034	23035	199	11570	7206	12416	90	11328	4238	1137	2164	12787
sace_c	0	0	0	0	0	0	0	0	0	0	0	0	0	23403	5194	sace_c_04464	12270	199	23404	23401	23402	199	11570	7206	12416	0	0	0	0	0	0
sace_d	1467	3898	1857	2987	640	5821	1966	4022	4020	5823	5408	4026	2686	23475	5194	sace_d_01751	12270	199	23476	23477	23478	199	11570	7206	12416	11328	4238	1137	2164	12787	11717
sace_e	3898	1857	2987	640	5821	1966	4022	4020	5823	5408	4026	677	2686	23732	5194	sace_e_5421	12270	199	23733	23734	23735	199	11570	7206	12416	0	0	0	0	0	0
sapa	18132	2987	640	18131	5821	1966	4022	4020	5823	5408	4026	677	2686	24776	5194	sapa_c93_21663	12270	199	24775	24774	199	11570	7206	12416	90	11328	11328	4238	1137	2164	18130
sami_a	0	0	0	0	0	0	0	0	0	0	0	0	0	0	0	sami_a_c511_20564	24126	12270	199	11570	7206	0	0	0	0	0	0	0	0	0	0
saba_a	3898	1857	280	2987	640	5821	1966	4022	4020	5823	5408	4026	677	2686	5194	saba_a_673.19	12270	199	22385	22384	199	4372	1448	1449	468	2191	468	2191	896	12787	11132
saba_b	1857	280	2987	640	15942	5821	1966	4022	4020	5823	5408	4026	677	2686	5194	saba_b_c773_24081	12270	199	22691	22690	199	22689	0	0	0	0	0	0	0	0	0
saku	0	0	0	0	0	0	0	0	0	0	0	0	677	2686	5194	saku_c2036.7	12270	199	17742	23832	199	11570	0	0	0	0	0	0	0	0	0
klwa	0	0	0	0	0	0	12137	12169	12337	12275	753	12416	3982	12278	199	klwa_034-snap.4	12270	9576	3496	0	0	0	0	0	0	0	0	0	0	0	0
klth	147	4693	5224	4675	10310	3948	4110	4196	691	690	351	37	1679	4288	4282	klth0c10560g	12271	12270	12269	6816	4277	3072	945	21998	4210	10807	10808	4211	12268	880	2368
klla	0	0	0	0	0	0	0	465	10828	10829	10913	2191	468	1212	75	klla0b00352g	407	1191	1192	2412	133	10914	10915	1740	54	2252	5087	10916	945	5086	5085
caal_a	2831	3	3	20917	652	277	653	654	655	656	657	658	660	1025	1024	caal_a_19.2350	9	1022	1021	1020	1019	1018	1017	190	82	696	502	1015	1014	1013	1012
caal_b	3	21026	4716	277	653	654	655	656	657	658	660	660	1025	1024	1023	caal_b_00361	9	1022	1021	1019	1018	1017	190	82	696	502	1015	1014	1013	1012	1009
caal_b	3	3	21026	4716	277	653	654	655	656	657	658	660	660	1025	1024	caal_b_00362	1023	9	1022	1021	1019	1018	1017	190	82	696	502	1015	1014	1013	1012
cadu	4709	4710	4711	2831	4840	277	653	654	655	656	657	658	660	1025	1024	cadu_10040	9	1022	1021	21137	1019	1018	1017	190	82	696	502	1015	1014	1013	1012
catr	277	9148	2633	3747	653	654	655	1024	1025	660	658	658	9147	657	656	catr_03278	9146	9	1022	9145	262	2733	2734	2734	2735	2736	6386	2737	2736	2729	1022
capa	2908	2906	978	21422	2903	2902	418	2901	2900	2899	3555	2859	2913	1025	1024	capa_03043	2895	2252	3752	3751	3750	3749	8598	3748	3747	3746	3744	2843	2843	8599	4354
loel	2906	13174	978	2904	2903	2902	418	2901	2900	2899	3555	2859	2913	1025	1024	loel_03562	13175	2895	2252	3752	3751	3750	3749	3748	12802	3747	3747	3746	13176	3744	2843
cagu	463	301	345	21323	1112	7106	407	260	1111	1110	1109	1107	2413	465	2682	cagu_05158	662	4541	512	199	1092	82	124	340	7107	7108	328	7109	7110	7111	0
cagu	7200	159	2734	2733	262	262	6352	340	2007	662	2682	2682	7201	1025	1024	cagu_05806	9	1022	7202	2633	277	7203	2400	3010	301	4263	4262	7204	4260	2430	3857
deha	1836	1837	534	1838	1839	9392	9392	161	160	159	2734	2733	242	1025	1024	deha2c03718g	9	2679	1022	9591	2633	2633	277	9590	2400	3010	301	4263	4262	9589	4260

pist	3307	15202	3869	3868	4280	4281	1857	50	15090	4284	4285	14916	4286	876	4289	pist_igi19138887	296	2823	280	2044	316	6611	7160	7160	2828	2827	2826	2825	147	147	147
pist	2811	14956	14955	2727	15215	2729	2736	2737	6386	2736	2735	2734	262	1025	1024	pist_igi20835632	9	2679	1022	2633	2633	277	2400	2511	2510	2799	1	2803	2804	2805	187

Homologs of ORF YOR378W. The protein family classification of the adjacent genes is shown and the homologous neighbours are highlighted in the same colour.

sace_a	sace0o11682g	sace0o11704g	sace0o11726g	sace0o11748g	sace0o11770g	sace0o11792g	sace0o11814g	sace0o11836g	sace0o11858g	sace0o11880g	sace0o11902g	sace0o11924g	sace0o11946g	sace0o11968g	sace0o11990g	sace0o12012g	sace0o12034g	sace0o12056g	sace0o12078g	sace0o12100g	sace0o12122g	sace0o12144g	sace0o12166g	sace0o12188g	sace0o12210g	sace0o12232g	sace0o12254g	sace0o12276g	sace0o12298g	sace0o12320g	sace0o12342g
sace_c	Null	Null	Null	Null	Null	Null	Null	Null	Null	Null	Null	Null	Null	sace_c_04466	sace_c_04463	sace_c_04464	sace_c_04460	sace_c_04456	sace_c_04467	sace_c_04457	sace_c_04462	sace_c_04458	sace_c_04465	sace_c_04459	sace_c_04461	Null	Null	Null	Null	Null	Null
sace_d	sace_d_01735	sace_d_01736	sace_d_01737	sace_d_01738	sace_d_01739	sace_d_01740	sace_d_01741	sace_d_01742	sace_d_01743	sace_d_01744	sace_d_01745	sace_d_01746	sace_d_01748	sace_d_01749	sace_d_01750	sace_d_01751	sace_d_01752	sace_d_01753	sace_d_01754	sace_d_01755	sace_d_01756	sace_d_01757	sace_d_01758	sace_d_01759	sace_d_01760	sace_d_05710	sace_d_01763	sace_d_01764	sace_d_01765	sace_d_01766	sace_d_01767
sace_e	sace_e_5406	sace_e_5407	sace_e_5408	sace_e_5409	sace_e_5410	sace_e_5411	sace_e_5412	sace_e_5413	sace_e_5414	sace_e_5415	sace_e_5416	sace_e_5417	sace_e_5418	sace_e_5419	sace_e_5420	sace_e_5421	sace_e_5422	sace_e_5423	sace_e_5424	sace_e_5425	sace_e_5426	sace_e_5427	sace_e_5428	sace_e_5429	sace_e_5430	Null	Null	Null	Null	Null	Null
sapa	sapa_c93_21729	sapa_c93_21720	sapa_c93_21717	sapa_c93_21718	sapa_c93_21716	sapa_c93_21714	sapa_c93_21712	sapa_c93_21709	sapa_c93_21702	sapa_c93_21696	sapa_c93_21692	sapa_c93_21686	sapa_c93_21684	sapa_c93_21683	sapa_c93_21676	sapa_c93_21663	sapa_c93_21658	sapa_c93_21650	sapa_c93_21646	sapa_c93_21642	sapa_c93_21639	sapa_c93_21633	sapa_c93_21630	sapa_c93_21626	sapa_c93_21621	sapa_c93_21616	sapa_c93_21613	sapa_c93_21610	sapa_c93_21608	sapa_c93_21606	sapa_c93_21605
sami_a	Null	Null	Null	Null	Null	Null	Null	Null	Null	Null	Null	Null	Null	Null	Null	sami_a_c511_20564	sami_a_c511_20565	sami_a_c511_20556	sami_a_c511_20544	sami_a_c511_20540	sami_a_c511_20539	Null	Null	Null	Null	Null	Null	Null	Null	Null	Null
saba_a	saba_a_673.35	saba_a_673.34	saba_a_673.33	saba_a_673.32	saba_a_673.31	saba_a_673.30	saba_a_673.29	saba_a_673.28	saba_a_673.27	saba_a_673.26	saba_a_673.25	saba_a_673.24	saba_a_673.23	saba_a_673.22	saba_a_673.20	saba_a_673.19	saba_a_673.18	saba_a_673.17	saba_a_673.16	saba_a_673.15	saba_a_673.14	saba_a_673.13	saba_a_673.12	saba_a_673.11	saba_a_673.10	saba_a_673.9	saba_a_673.8	saba_a_673.7	saba_a_673.6	saba_a_673.5	saba_a_673.4
saba_b	saba_b_c773_24152	saba_b_c773_24144	saba_b_c773_24140	saba_b_c773_24134	saba_b_c773_24135	saba_b_c773_24131	saba_b_c773_24127	saba_b_c773_24125	saba_b_c773_24122	saba_b_c773_24117	saba_b_c773_24115	saba_b_c773_24109	saba_b_c773_24102	saba_b_c773_24096	saba_b_c773_24088	saba_b_c773_24081	saba_b_c773_24077	saba_b_c773_24064	saba_b_c773_24052	saba_b_c773_24048	saba_b_c773_24043	saba_b_c773_24040	Null	Null	Null	Null	Null	Null	Null	Null	Null
saku	Null	Null	Null	Null	Null	Null	Null	Null	Null	Null	Null	Null	saku_c2036.11	saku_c2036.10	saku_c2036.8	saku_c2036.7	saku_c2036.6	saku_c2036.5	saku_c2036.4	saku_c2036.3	saku_c2036.2	saku_c2036.1	Null	Null	Null	Null	Null	Null	Null	Null	Null
klwa	Null	Null	Null	Null	Null	Null	klwa_034-snap.13	klwa_034-snap.12	klwa_034-snap.11	klwa_034-snap.10	klwa_034-snap.9	klwa_034-snap.8	klwa_034-snap.7	klwa_034-snap.6	klwa_034-snap.5	klwa_034-snap.4	klwa_034-snap.3	klwa_034-snap.2	klwa_034-snap.1	Null	Null	Null	Null	Null	Null	Null	Null	Null	Null	Null	Null
klth	klth0c10912g	klth0c10890g	klth0c10868g	klth0c10846g	klth0c10824g	klth0c10802g	klth0c10780g	klth0c10758g	klth0c10736g	klth0c10714g	klth0c10670g	klth0c10648g	klth0c10626g	klth0c10604g	klth0c10582g	klth0c10560g	klth0c10538g	klth0c10516g	klth0c10494g	klth0c10450g	klth0c10428g	klth0c10406g	klth0c10384g	klth0c10362g	klth0c10340g	klth0c10318g	klth0c10296g	klth0c10274g	klth0c10252g	klth0c10230g	klth0c10208g
klla	Null	Null	Null	Null	Null	Null	Null	klla0b00143g	klla0b00166g	klla0b00187g	klla0b00220g	klla0b00242g	klla0b00264g	klla0b00286g	klla0b00308g	klla0b00352g	klla0b00385g	klla0b00407g	klla0b00429g	klla0b00451g	klla0b00473g	klla0b00495g	klla0b00517g	klla0b00539g	klla0b00561g	klla0b00583g	klla0b00627g	klla0b00649g	klla0b00671g	klla0b00693g	klla0b00715g
caal_a	caal_a_19.999	caal_a_19.1002	caal_a_19.1005	caal_a_19.1830	caal_a_19.1831	caal_a_19.1832	caal_a_19.1833	caal_a_19.1834	caal_a_19.1835	caal_a_19.1836	caal_a_19.1837	caal_a_19.1839	caal_a_19.1840	caal_a_19.2352	caal_a_19.2351	caal_a_19.2350	caal_a_19.2347	caal_a_19.2346	caal_a_19.2344	caal_a_19.2343.1	caal_a_19.2343	caal_a_19.2342	caal_a_19.2341	caal_a_19.2340	caal_a_19.2337	caal_a_19.2336	caal_a_19.2335	caal_a_19.2334	caal_a_19.2333	caal_a_19.2332	caal_a_19.2331
caal_b	caal_b_00376	caal_b_00375	caal_b_00374	caal_b_00373	caal_b_00372	caal_b_00371	caal_b_00370	caal_b_00369	caal_b_00368	caal_b_00367	caal_b_00366	caal_b_00365	caal_b_00364	caal_b_00363	caal_b_00362	caal_b_00361	caal_b_00360	caal_b_00359	caal_b_00358	caal_b_00357	caal_b_00356	caal_b_00355	caal_b_00354	caal_b_00353	caal_b_00352	caal_b_00351	caal_b_00350	caal_b_00349	caal_b_00348	caal_b_00347	caal_b_00346
caal_b	caal_b_00377	caal_b_00376	caal_b_00375	caal_b_00374	caal_b_00373	caal_b_00372	caal_b_00371	caal_b_00370	caal_b_00369	caal_b_00368	caal_b_00367	caal_b_00366	caal_b_00365	caal_b_00364	caal_b_00363	caal_b_00362	caal_b_00361	caal_b_00360	caal_b_00359	caal_b_00358	caal_b_00357	caal_b_00356	caal_b_00355	caal_b_00354	caal_b_00353	caal_b_00352	caal_b_00351	caal_b_00350	caal_b_00349	caal_b_00348	caal_b_00347
cadu	cadu_09890	cadu_09900	cadu_09910	cadu_09920	cadu_09930	cadu_09940	cadu_09950	cadu_09960	cadu_09970	cadu_09980	cadu_09990	cadu_10000	cadu_10010	cadu_10020	cadu_10030	cadu_10040	cadu_10050	cadu_10060	cadu_10070	cadu_10080	cadu_10090	cadu_10100	cadu_10110	cadu_10120	cadu_10130	cadu_10140	cadu_10150	cadu_10160	cadu_10170	cadu_10180	cadu_10190
catr	catr_03293	catr_03292	catr_03291	catr_03290	catr_03289	catr_03288	catr_03287	catr_03286	catr_03285	catr_03284	catr_03283	catr_03281	catr_03282	catr_03280	catr_03279	catr_03278	catr_03277	catr_03276	catr_03275	catr_03274	catr_03273	catr_03272	catr_03271	catr_03270	catr_03269	catr_03268	catr_03267	catr_03266	catr_03265	catr_03264	catr_03263
capa	capa_03058	capa_03057	capa_03056	capa_03055	capa_03054	capa_03053	capa_03052	capa_03051	capa_03050	capa_03049	capa_03048	capa_03047	capa_03046	capa_03045	capa_03044	capa_03043	capa_03042	capa_03041	capa_03040	capa_03039	capa_03038	capa_03037	capa_03036	capa_03035	capa_03034	capa_03033	capa_03032	capa_03031	capa_03030	capa_03029	capa_03028
loel	loel_03547	loel_03548	loel_03549	loel_03550	loel_03551	loel_03552	loel_03553	loel_03554	loel_03555	loel_03556	loel_03557	loel_03558	loel_03559	loel_03560	loel_03561	loel_03562	loel_03563	loel_03564	loel_03565	loel_03566	loel_03567	loel_03568	loel_03569	loel_03570	loel_03571	loel_03572	loel_03573	loel_03574	loel_03575	loel_03576	loel_03577
cagu	cagu_05143	cagu_05144	cagu_05145	cagu_05146	cagu_05147	cagu_05148	cagu_05149	cagu_05150	cagu_05151	cagu_05152	cagu_05153	cagu_05154	cagu_05155	cagu_05156	cagu_05157	cagu_05158	cagu_05159	cagu_05160	cagu_05161	cagu_05162	cagu_05163	cagu_05164	cagu_05165	cagu_05166	cagu_05167	cagu_05168	cagu_05169	cagu_05170	cagu_05171	cagu_05172	Null
cagu	cagu_05791	cagu_05792	cagu_05793	cagu_05794	cagu_05795	cagu_05796	cagu_05797	cagu_05798	cagu_05799	cagu_05800	cagu_05801	cagu_05802	cagu_05803	cagu_05804	cagu_05805	cagu_05806	cagu_05807	cagu_05808	cagu_05809	cagu_05810	cagu_05811	cagu_05812	cagu_05813	cagu_05814	cagu_05815	cagu_05816	cagu_05817	cagu_05818	cagu_05819	cagu_05820	cagu_05821
deha	deha2c04048g	deha2c04026g	deha2c04004g	deha2c03982g	deha2c03960g	deha2c03938g	deha2c03916g	deha2c03894g	deha2c03872g	deha2c03850g	deha2c03828g	deha2c03806g	deha2c03784g	deha2c03762g	deha2c03740g	deha2c03718g	deha2c03696g	deha2c03674g	deha2c03652g	deha2c03630g	deha2c03608g	deha2c03586g	deha2c03564g	deha2c03520g	deha2c03476g	deha2c03454g	deha2c03432g	deha2c03410g	deha2c03388g	deha2c03366g	deha2c03344g
pist	pist_igi19985372	pist_igi19985373	pist_igi19138885	pist_igi19985374	pist_igi16595596	pist_igi16595597	pist_igi19985375	pist_igi16595598	pist_igi18290484	pist_igi19138886	pist_igi16595599	pist_igi20834009	pist_igi19985376	pist_igi18290485	pist_igi20834010	pist_igi19138887	pist_igi16595600	pist_igi16595601	pist_igi16595602	pist_igi16595603	pist_igi16595604	pist_igi19138888	pist_igi18290486	pist_igi20834011	pist_igi18290487	pist_igi19138889	pist_igi19985377	pist_igi16595605	pist_igi20834012	pist_igi16595606	pist_igi19985378
pist	pist_igi16601833	pist_igi16601832	pist_igi16601831	pist_igi16601830	pist_igi19986945	pist_igi18292105	pist_igi20835634	pist_igi16601829	pist_igi19986944	pist_igi19140408	pist_igi16601828	pist_igi16601827	pist_igi16601826	pist_igi16601825	pist_igi20835633	pist_igi20835632	pist_igi16601824	pist_igi18292104	pist_igi16601823	pist_igi16601822	pist_igi16601821	pist_igi19986943	pist_igi16601820	pist_igi18292103	pist_igi19140407	pist_igi19986942	pist_igi18292102	pist_igi16601819	pist_igi19140406	pist_igi16601818	pist_igi19140405

Homologs of ORF YOR378W. The names of the adjacent genes are shown and the homologous neighbours are highlighted in the same colour.

Cluster J (lineage 6): homologs of *K. lactis* KNQ1 gene

sace_c	0	0	0	0	0	0	0	0	0	33	5098	2737	4369	896	12627	sace_c_05667	484	6028	4363	4351	1445	1443	1439	2496	23424	2357	468	945	178	3567	1182
sapa	0	0	0	33	82	5098	2737	4369	24589	2191	2191	2191	18051	468	896	sapa_c345_7127	18050	75	484	6028	4363	4351	1445	1443	1439	2496	2412	2357	468	0	0
sami_a	0	0	0	0	0	0	0	0	0	0	0	0	0	0	896	sami_a_c1152_6745	15320	17776	0	0	0	0	0	0	0	0	0	0	0	0	0
saba_a	0	0	0	0	0	0	0	0	0	0	0	0	15446	21	896	saba_a_601.4	124	12755	340	75	2987	1374	199	15447	0	0	0	0	0	0	0
saba_b	0	0	0	0	0	0	0	0	0	0	0	0	15446	21	896	saba_b_c68_15452	124	12755	340	22663	75	2987	1374	199	15447	57	0	0	0	0	0
sakl	0	0	0	896	291	2042	236	5582	1579	2682	465	17516	17517	4522	12627	sakl0f00440g	881	870	4996	1555	5583	12075	2377	2371	2376	4995	6948	262	3675	4210	1600
klwa	0	0	0	0	0	0	0	0	0	0	0	0	3010	277	11098	klwa_333-snap.4	12626	2899	4374	4690	5240	1638	57	0	0	0	0	0	0	0	0
klth	0	0	0	0	0	0	0	0	0	0	0	6344	12416	1579	12627	klth0h16214g	465	12626	2899	4374	4690	12625	5240	57	2200	6113	2930	4084	4084	1570	1532
klla	75	896	11095	11094	11093	11092	11091	6680	4372	11090	7616	340	11089	262	6964	klla0c18931g	2914	3254	1604	1429	3375	5100	3064	11087	4442	11086	21870	262	340	64	1883

Homologs of K. lactis KNQ1 gene. The protein family classification of the adjacent genes is shown and the homologous neighbours are highlighted in the same colour.

sace_c	Null	Null	Null	Null	Null	Null	Null	Null	Null	sace_c_05497	sace_c_05500	sace_c_05483	sace_c_05486	sace_c_05665	sace_c_05666	sace_c_05667
sapa	Null	Null	Null	sapa_c345_7189	sapa_c345_7185	sapa_c345_7178	sapa_c345_7176	sapa_c345_7166	sapa_c345_7162	sapa_c345_7161	sapa_c345_7159	sapa_c345_7158	sapa_c345_7157	sapa_c345_7151	sapa_c345_7143	sapa_c345_7127
sami_a	Null	Null	Null	Null	Null	Null	Null	Null	Null	Null	Null	Null	Null	Null	sami_a_c1152_6761	sami_a_c1152_6745
saba_a	Null	Null	Null	Null	Null	Null	Null	Null	Null	Null	Null	Null	saba_a_601.1	saba_a_601.2	saba_a_601.3	saba_a_601.4
saba_b	Null	Null	Null	Null	Null	Null	Null	Null	Null	Null	Null	Null	saba_b_c68_15483	saba_b_c68_15469	saba_b_c68_15461	saba_b_c68_15452
sakl	Null	Null	Null	sakl0f00110g	sakl0f00132g	sakl0f00176g	sakl0f00198g	sakl0f00242g	sakl0f00286g	sakl0f00308g	sakl0f00330g	sakl0f00352g	sakl0f00374g	sakl0f00396g	sakl0f00418g	sakl0f00440g
klwa	Null	Null	Null	Null	Null	Null	Null	Null	Null	Null	Null	Null	klwa_333-snap.1	klwa_333-snap.2	klwa_333-snap.3	klwa_333-snap.4
klth	Null	Null	Null	Null	Null	Null	Null	Null	Null	Null	Null	klth0h16456g	klth0h16390g	klth0h16258g	klth0h16236g	klth0h16214g
klla	klla0c19316g	klla0c19272g	klla0c19250g	klla0c19228g	klla0c19206g	klla0c19184g	klla0c19132g	klla0c19107g	klla0c19085g	klla0c19063g	klla0c19041g	klla0c19019g	klla0c18997g	klla0c18975g	klla0c18953g	klla0c18931g

sace_c	sace_c_05510	sace_c_05513	sace_c_05493	sace_c_05506	sace_c_05505	sace_c_05485	sace_c_05492	sace_c_05508	sace_c_05516	sace_c_05503	sace_c_05494	sace_c_05491	sace_c_05514	sace_c_05495	sace_c_05498
sapa	sapa_c345_7124	sapa_c345_7119	sapa_c345_7108	sapa_c345_7103	sapa_c345_7099	sapa_c345_7095	sapa_c345_7093	sapa_c345_7089	sapa_c345_7085	sapa_c345_7078	sapa_c345_7076	sapa_c345_7073	sapa_c345_7068	Null	Null
sami_a	sami_b_c1152.1	sami_a_c1152_6739	Null	Null	Null	Null	Null	Null	Null	Null	Null	Null	Null	Null	Null
saba_a	saba_a_601.5	saba_a_601.6	saba_a_601.7	saba_a_601.8	saba_a_601.9	saba_a_601.10	saba_a_601.11	saba_a_601.13	Null	Null	Null	Null	Null	Null	Null
saba_b	saba_b_c68_15444	saba_b_c68_15440	saba_b_c68_15430	saba_b_c68_15426	saba_b_c68_15419	saba_b_c68_15411	saba_b_c68_15408	saba_b_c68_15399	saba_b_c68_15395	saba_b_c68_15393	Null	Null	Null	Null	Null
sakl	sakl0f00462g	sakl0f00484g	sakl0f00506g	sakl0f00528g	sakl0f00550g	sakl0f00572g	sakl0f00594g	sakl0f00616g	sakl0f00638g	sakl0f00660g	sakl0f00682g	sakl0f00704g	sakl0f00726g	sakl0f00748g	sakl0f00770g
klwa	klwa_333-snap.5	klwa_333-snap.6	klwa_333-snap.7	klwa_333-snap.8	klwa_333-snap.9	klwa_333-snap.10	klwa_333-snap.11	Null	Null	Null	Null	Null	Null	Null	Null
klth	klth0h16192g	klth0h16170g	klth0h16148g	klth0h16126g	klth0h16104g	klth0h16082g	klth0h16060g	klth0h16038g	klth0h16016g	klth0h15994g	klth0h15972g	klth0h15950g	klth0h15928g	klth0h15906g	klth0h15884g
klla	klla0c18909g	klla0c18887g	klla0c18865g	klla0c18843g	klla0c18821g	klla0c18799g	klla0c18777g	klla0c18755g	klla0c18744g	klla0c18722g	klla0c18711g	klla0c18700g	klla0c18678g	klla0c18656g	klla0c18634g

Homologs of K. lactis KNQ1 gene. The names of the adjacent genes are shown and the homologous neighbours are highlighted in the same colour.

Cluster K (lineage 7): homologs of C. albicans ORFs caal_a_19.7554
and caal_a_19.7336

caal_a	4369	266	4370	4371	2602	4372	4373	4374	4375	4376	64	4377	277	902	1857	caal_a_19.7336	4378	527	4379	4380	21011	3278	4381	4382	4383	3920	3919	1367	3917	3916	3915
caal_a	983	351	3892	3905	3904	3903	525	4498	4499	2066	3	4501	4502	4503	4504	caal_a_19.7554	4505	4506	190	4507	2934	4508	82	82	4509	4510	149	3564	64	4512	4513
caal_b	4369	266	4370	4371	2602	4372	4373	4374	4375	4376	64	4377	277	902	1857	caal_b_02209	4378	527	4379	4380	3278	4381	4382	4383	3920	3919	1367	3917	3916	3915	3914
caal_b	983	351	3892	3905	3904	3903	525	4498	4499	2066	3	4501	4502	4503	4504	caal_b_02248	4505	4506	190	4507	2934	4508	82	82	4509	4510	149	3564	64	4512	4513
cadu	4369	266	4370	4371	2602	4372	4373	4374	4375	4376	64	4377	277	902	1857	cadu_34690	4378	527	4380	3278	4381	4382	4383	3920	3919	1367	3917	4836	3915	3914	3913
cadu	3906	983	351	3892	3905	3904	3903	525	4498	4499	2066	3	4501	4502	4503	cadu_34960	4505	21170	190	4889	2934	4508	82	82	4509	4510	149	3564	64	4512	4513
catr	351	3906	3907	3905	3904	3903	525	4498	4499	2066	4501	4502	9359	190	4505	catr_05874	9358	82	4508	8972	2934	4509	4510	149	3564	64	4512	2007	4517	4513	4513
catr	3916	4370	4371	2602	64	4376	4375	4374	4373	4372	4377	4377	277	902	1857	catr_05923	527	527	4378	4380	9360	3278	266	4369	3564	187	187	679	4366	4361	4361
capa	4509	8590	82	3913	6745	8591	3910	82	1024	8592	190	8593	2934	4508	8594	capa_02998	527	4378	4380	8595	6366	3278	3916	4381	4382	1363	4383	3920	3919	3917	3915
loel	8598	4344	4343	13383	13382	82	1024	2934	4508	13381	13380	13378	13379	190	13377	loel_05322	13376	4378	13375	13374	13373	3278	3916	4381	4382	1363	12982	13372	4383	3920	3919
cagu	6374	3108	814	3112	3112	200	6373	6372	202	236	190	6371	2934	4508	6370	cagu_00118	527	527	6369	4378	6368	6367	1596	6366	3278	4381	4382	1363	192	193	193
cagu	2072	2073	2080	2079	3104	3105	6374	3108	814	3112	3112	200	6373	6372	202	cagu_00124	190	6371	2934	4508	6370	236	527	527	6369	4378	6368	6367	1596	6366	3278
deha	3105	3106	3108	814	200	9789	203	202	236	9788	190	9787	2934	4508	9786	deha2e02794g	527	4378	9785	9784	1596	6366	896	346	21699	9783	6500	3278	3916	4381	4382
deha	2069	607	2072	2073	2080	2079	3104	3105	3106	3108	814	200	9789	203	202	deha2e02948g	9788	190	9787	2934	4508	9786	236	527	4378	9785	9784	1596	6366	896	346
pist	4466	4467	4468	4469	4470	2257	187	4473	4472	662	536	2090	189	4474	33	pist_igi16259683	14823	190	14822	2934	4508	14821	236	527	4378	9785	15251	6366	3278	3916	4381
pist	4473	4472	662	536	2090	189	4474	33	236	14823	190	14822	2934	4508	14821	pist_igi19055263	527	4378	9785	15251	6366	3278	3916	4381	4382	1363	4383	14820	3920	3919	3917
pist	3298	57	3396	3395	3394	3393	3391	3390	3389	3388	3360	14938	14939	187	1579	pist_igi20835350	14834	3363	3362	3361	3364	3365	3309	3310	3278	3276	3275	3376	3384	535	242
calu	1925	190	1923	1922	90	895	1538	692	693	917	916	913	7656	203	202	calu_02516	7655	190	2281	7654	3644	3646	2864	2578	3653	3650	3650	6918	3648	7653	2987
yali	3943	1580	1608	19376	19377	19378	18168	492	2751	280	2752	3112	531	4621	24	yali0d20196g	19379	249	1560	4140	321	896	3564	1577	101	19380	19381	19382	19383	19384	321
yali	18168	492	2751	280	2752	3112	531	4621	24	236	19379	249	1560	4140	321	yali0d20350g	3564	1577	101	19380	19381	19382	19383	19384	321	19385	4182	19386	19387	618	1460

Homologs of C. albicans ORFs caal_a_19.7554 and caal_a_19.7336. The protein family classification of the adjacent genes is shown and the homologous neighbours are highlighted in the same colour.

caal_a	caal_a_19.7319	caal_a_19.7320	caal_a_19.7321	caal_a_19.7322	caal_a_19.7323	caal_a_19.7324	caal_a_19.7325	caal_a_19.7326	caal_a_19.7327	caal_a_19.7328	caal_a_19.7329	caal_a_19.7330	caal_a_19.7331	caal_a_19.7332	caal_a_19.7335	caal_a_19.7336
caal_a	caal_a_19.6595	caal_a_19.6594	caal_a_19.6592	caal_a_19.6591	caal_a_19.6590	caal_a_19.6589	caal_a_19.7546	caal_a_19.7547	caal_a_19.7548	caal_a_19.7549	caal_a_19.7550	caal_a_19.7551	caal_a_19.7552	caal_a_19.7553	caal_a_19.7555	caal_a_19.7554
caal_b	caal_b_02194	caal_b_02195	caal_b_02196	caal_b_02197	caal_b_02198	caal_b_02199	caal_b_02200	caal_b_02201	caal_b_02202	caal_b_02203	caal_b_02204	caal_b_02205	caal_b_02206	caal_b_02207	caal_b_02208	caal_b_02209
caal_b	caal_b_02233	caal_b_02234	caal_b_02235	caal_b_02236	caal_b_02237	caal_b_02238	caal_b_02239	caal_b_02240	caal_b_02241	caal_b_02242	caal_b_02243	caal_b_02244	caal_b_02245	caal_b_02246	caal_b_02247	caal_b_02248
cadu	cadu_34590	cadu_34600	cadu_34610	cadu_34615	cadu_34620	cadu_34630	cadu_34640	cadu_34650	cadu_34655	cadu_34660	cadu_34665	cadu_34667	cadu_34670	cadu_34680	cadu_34685	cadu_34690
cadu	cadu_34840	cadu_34850	cadu_34860	cadu_34870	cadu_34880	cadu_34885	cadu_34890	cadu_34900	cadu_34910	cadu_34915	cadu_34920	cadu_34930	cadu_34940	cadu_34950	cadu_34955	cadu_34960
catr	catr_05889	catr_05888	catr_05887	catr_05886	catr_05885	catr_05884	catr_05883	catr_05882	catr_05881	catr_05880	catr_05879	catr_05878	catr_05877	catr_05876	catr_05875	catr_05874
catr	catr_05908	catr_05909	catr_05910	catr_05911	catr_05912	catr_05913	catr_05914	catr_05915	catr_05916	catr_05917	catr_05918	catr_05919	catr_05920	catr_05921	catr_05922	catr_05923
capa	capa_02983	capa_02984	capa_02985	capa_02986	capa_02987	capa_02988	capa_02989	capa_02990	capa_02991	capa_02992	capa_02993	capa_02994	capa_02995	capa_02996	capa_02997	capa_02998
loel	loel_05337	loel_05336	loel_05335	loel_05334	loel_05333	loel_05332	loel_05331	loel_05330	loel_05329	loel_05328	loel_05327	loel_05325	loel_05326	loel_05324	loel_05323	loel_05322
cagu	cagu_00133	cagu_00132	cagu_00131	cagu_00130	cagu_00129	cagu_00128	cagu_00127	cagu_00126	cagu_00125	cagu_00124	cagu_00123	cagu_00122	cagu_00121	cagu_00120	cagu_00119	cagu_00118
cagu	cagu_00139	cagu_00138	cagu_00137	cagu_00136	cagu_00135	cagu_00134	cagu_00133	cagu_00132	cagu_00131	cagu_00130	cagu_00129	cagu_00128	cagu_00127	cagu_00126	cagu_00125	cagu_00124
deha	deha2e03190g	deha2e03168g	deha2e03146g	deha2e03124g	deha2e03058g	deha2e03036g	deha2e02992g	deha2e02970g	deha2e02948g	deha2e02926g	deha2e02904g	deha2e02882g	deha2e02860g	deha2e02838g	deha2e02816g	deha2e02794g
deha	deha2e03344g	deha2e03322g	deha2e03300g	deha2e03278g	deha2e03256g	deha2e03234g	deha2e03212g	deha2e03190g	deha2e03168g	deha2e03146g	deha2e03124g	deha2e03058g	deha2e03036g	deha2e02992g	deha2e02970g	deha2e02948g
pist	pist_igi16259690	pist_igi19055267	pist_igi16259689	pist_igi18206493	pist_igi16259688	pist_igi19055266	pist_igi20749922	pist_igi16259687	pist_igi16259686	pist_igi18206492	pist_igi16259685	pist_igi18206491	pist_igi20749921	pist_igi16259684	pist_igi19055265	pist_igi16259683
pist	pist_igi16259687	pist_igi16259686	pist_igi18206492	pist_igi16259685	pist_igi18206491	pist_igi20749921	pist_igi16259684	pist_igi19055265	pist_igi16259683	pist_igi16259682	pist_igi16259681	pist_igi16259680	pist_igi19901909	pist_igi19055264	pist_igi16259679	pist_igi19055263
pist	pist_igi18291846	pist_igi19986663	pist_igi19986664	pist_igi16600789	pist_igi16600790	pist_igi16600791	pist_igi18291847	pist_igi18291848	pist_igi19140173	pist_igi19986665	pist_igi19140174	pist_igi16600792	pist_igi16600793	pist_igi20835349	pist_igi18291849	pist_igi20835350
calu	calu_02531	calu_02530	calu_02529	calu_02528	calu_02527	calu_02526	calu_02525	calu_02524	calu_02523	calu_02522	calu_02521	calu_02520	calu_02519	calu_02518	calu_02517	calu_02516
yali	yali0d19866g	yali0d19888g	yali0d19910g	yali0d19932g	yali0d19954g	yali0d19976g	yali0d19998g	yali0d20020g	yali0d20042g	yali0d20064g	yali0d20086g	yali0d20108g	yali0d20130g	yali0d20152g	yali0d20174g	yali0d20196g
yali	yali0d19998g	yali0d20020g	yali0d20042g	yali0d20064g	yali0d20086g	yali0d20108g	yali0d20130g	yali0d20152g	yali0d20174g	yali0d20196g	yali0d20218g	yali0d20240g	yali0d20262g	yali0d20306g	yali0d20328g	yali0d20350g

caal_a	caal_a_19.7337	caal_a_19.7339	caal_a_19.7340	caal_a_19.7341	caal_a_19.7341.1	caal_a_19.7342	caal_a_19.7343	caal_a_19.7344	caal_a_19.7345	caal_a_19.6612	caal_a_19.6610	caal_a_19.6608	caal_a_19.6607	caal_a_19.6606	caal_a_19.6605
caal_a	caal_a_19.7556	caal_a_19.7557	caal_a_19.7558	caal_a_19.7561	caal_a_19.7563	caal_a_19.7564	caal_a_19.7565	caal_a_19.7566	caal_a_19.7567	caal_a_19.7568	caal_a_19.7569	caal_a_19.7570	caal_a_19.7571	caal_a_19.7572	caal_a_19.7574
caal_b	caal_b_02210	caal_b_02211	caal_b_02212	caal_b_02213	caal_b_02214	caal_b_02215	caal_b_02216	caal_b_02217	caal_b_02218	caal_b_02219	caal_b_02220	caal_b_02221	caal_b_02222	caal_b_02223	caal_b_02224
caal_b	caal_b_02249	caal_b_02250	caal_b_02251	caal_b_02252	caal_b_02253	caal_b_02254	caal_b_02255	caal_b_02256	caal_b_02257	caal_b_02258	caal_b_02259	caal_b_02260	caal_b_02261	caal_b_02262	caal_b_02263
cadu	cadu_34700	cadu_34710	cadu_34720	cadu_34730	cadu_34740	cadu_34750	cadu_34760	cadu_34765	cadu_34770	cadu_34780	cadu_34785	cadu_34790	cadu_34795	cadu_34800	cadu_34805
cadu	cadu_34965	cadu_34968	cadu_34970	cadu_34980	cadu_34990	cadu_35000	cadu_35010	cadu_35020	cadu_35030	cadu_35035	cadu_35040	cadu_35050	cadu_35055	cadu_35060	cadu_35070
catr	catr_05873	catr_05872	catr_05871	catr_05870	catr_05869	catr_05868	catr_05867	catr_05866	catr_05865	catr_05864	catr_05863	catr_05862	catr_05861	catr_05860	catr_05859
catr	catr_05924	catr_05925	catr_05926	catr_05927	catr_05928	catr_05929	catr_05930	catr_05931	catr_05932	catr_05933	catr_05934	catr_05935	catr_05936	catr_05937	catr_05938
capa	capa_02999	capa_03000	capa_03001	capa_03002	capa_03003	capa_03004	capa_03005	capa_03006	capa_03007	capa_03008	capa_03009	capa_03010	capa_03011	capa_03012	capa_03013
loel	loel_05321	loel_05320	loel_05319	loel_05318	loel_05317	loel_05316	loel_05315	loel_05314	loel_05313	loel_05312	loel_05310	loel_05311	loel_05309	loel_05308	loel_05307
cagu	cagu_00117	cagu_00115	cagu_00116	cagu_00114	cagu_00113	cagu_00112	cagu_00111	cagu_00110	cagu_00109	cagu_00108	cagu_00107	cagu_00106	cagu_00105	cagu_00104	cagu_00103
cagu	cagu_00123	cagu_00122	cagu_00121	cagu_00120	cagu_00119	cagu_00118	cagu_00117	cagu_00115	cagu_00116	cagu_00114	cagu_00113	cagu_00112	cagu_00111	cagu_00110	cagu_00109
deha	deha2e02772g	deha2e02750g	deha2e02728g	deha2e02662g	deha2e02640g	deha2e02618g	deha2e02596g	deha2e02574g	deha2e02552g	deha2e02530g	deha2e02508g	deha2e02464g	deha2e02420g	deha2e02398g	deha2e02376g
deha	deha2e02926g	deha2e02904g	deha2e02882g	deha2e02860g	deha2e02838g	deha2e02816g	deha2e02794g	deha2e02772g	deha2e02750g	deha2e02728g	deha2e02662g	deha2e02640g	deha2e02618g	deha2e02596g	deha2e02574g
pist	pist_igi16259682	pist_igi16259681	pist_igi16259680	pist_igi19901909	pist_igi19055264	pist_igi16259679	pist_igi19055263	pist_igi20749920	pist_igi20749919	pist_igi16259678	pist_igi20749918	pist_igi16259677	pist_igi19901908	pist_igi19055262	pist_igi19055261
pist	pist_igi20749920	pist_igi20749919	pist_igi16259678	pist_igi20749918	pist_igi16259677	pist_igi19901908	pist_igi19055262	pist_igi19055261	pist_igi16259676	pist_igi19901907	pist_igi16259675	pist_igi16259674	pist_igi20749917	pist_igi18206490	pist_igi18206489
pist	pist_igi16600794	pist_igi20835351	pist_igi18291850	pist_igi16600795	pist_igi16600796	pist_igi18291851	pist_igi16600797	pist_igi20835352	pist_igi18291852	pist_igi16600798	pist_igi20835353	pist_igi18291853	pist_igi16600799	pist_igi20835354	pist_igi16600800
calu	calu_02515	calu_02514	calu_02513	calu_02512	calu_02511	calu_02510	calu_02509	calu_02508	calu_02507	calu_02506	calu_02505	calu_02504	calu_02503	calu_02502	calu_02501
yali	yali0d20218g	yali0d20240g	yali0d20262g	yali0d20306g	yali0d20328g	yali0d20350g	yali0d20394g	yali0d20416g	yali0d20438g	yali0d20460g	yali0d20482g	yali0d20504g	yali0d20526g	yali0d20548g	yali0d20570g
yali	yali0d20394g	yali0d20416g	yali0d20438g	yali0d20460g	yali0d20482g	yali0d20504g	yali0d20526g	yali0d20548g	yali0d20570g	yali0d20592g	yali0d20614g	yali0d20636g	yali0d20658g	yali0d20680g	yali0d20702g

Homologs of C. albicans ORFs caal_a_19.7554 and caal_a_19.7336. The names of the adjacent genes are shown and the homologous neighbours are highlighted in the same colour.

Cluster O (Lineage 9): homologs of ARN4 gene

Sheet1

sace_a	0	0	0	0	0	0	23013	33	23014	12568	340	340	12787	15692	15767	sace0o00308g	2191	468	23015	15409	1566	199	662	2370	2360	67	4216	4217	70	2267	1717
sace_b	0	0	0	0	0	0	0	0	0	0	0	0	0	15692	15767	sace_b_1o4_0034g	2191	468	23256	15409	1260	199	662	17139	2370	2360	67	4216	4217	70	2267
sace_d	0	0	0	0	0	0	0	0	0	0	0	0	0	15692	15767	sace_d_01250	2191	468	15409	1260	662	2370	2360	67	4216	4217	70	2267	1717	1716	1192
sace_e	0	0	0	0	0	0	0	0	0	0	0	0	12787	15692	15767	sace_e_4920	896	2191	468	23717	0	0	0	0	0	0	0	0	0	0	0
sace_e	0	0	0	0	0	0	0	0	0	0	0	12787	15692	15767	896	sace_e_4921	2191	468	23717	0	0	0	0	0	0	0	0	0	0	0	0
sapa	0	0	0	0	0	0	0	0	0	0	0	0	0	0	15767	sapa_c415_24456	0	0	0	0	0	0	0	0	0	0	0	0	0	0	0
sapa	0	0	0	0	0	0	0	0	0	0	0	0	18089	18076	15767	sapa_c416_24437	0	0	0	0	0	0	0	0	0	0	0	0	0	0	0
sami_a	0	0	0	0	0	0	0	0	0	0	0	696	17891	16907	405	sami_a_c633_10615	2191	24175	0	0	0	0	0	0	0	0	0	0	0	0	0
saba_a	0	0	0	0	0	0	0	0	0	0	0	696	15322	11132	12787	saba_a_479.6	0	0	0	0	0	0	0	0	0	0	0	0	0	0	0
saba_a	0	0	0	0	0	0	0	0	0	0	0	15692	15322	11132	12787	saba_a_673.6	2191	468	2191	468	1449	1448	4372	199	22384	22385	199	12270	1023	5194	2686

Homologs of ARN4 gene. The protein family classification of the adjacent genes is shown and the homologous neighbours are highlighted in the same colour.

sace_a	Null	Null	Null	Null	Null	Null	sace0o00110g	sace0o00132g	sace0o00154g	sace0o00176g	sace0o00198g	sace0o00220g	sace0o00242g	sace0o00264g	sace0o00286g	sace0o00308g	sace0o00330g	sace0o00352g	sace0o00374g	sace0o00396g	sace0o00418g	sace0o00440g	sace0o00462g	sace0o00484g	sace0o00506g	sace0o00528g	sace0o00550g	sace0o00572g	sace0o00594g	sace0o00616g	sace0o00638g
sace_b	Null	Null	Null	Null	Null	Null	Null	Null	Null	Null	Null	Null	Null	sace_b_1o4_0012g	sace_b_1o4_0023g	sace_b_1o4_0034g	sace_b_1o4_0045g	sace_b_1o4_0056g	sace_b_1o4_0067g	sace_b_1o4_0078g	sace_b_1o4_0100g	sace_b_1o4_0111g	sace_b_1o4_0122g	sace_b_1o4_0133g	sace_b_1o4_0144g	sace_b_1o4_0155g	sace_b_1o4_0166g	sace_b_1o4_0177g	sace_b_1o4_0188g	sace_b_1o4_0199g	sace_b_1o4_0210g
sace_d	Null	Null	Null	Null	Null	Null	Null	Null	Null	Null	Null	Null	Null	sace_d_01248	sace_d_01249	sace_d_01250	sace_d_01251	sace_d_01252	sace_d_01253	sace_d_01255	sace_d_01257	sace_d_01258	sace_d_01259	sace_d_01260	sace_d_01261	sace_d_01262	sace_d_01263	sace_d_01264	sace_d_01265	sace_d_01266	sace_d_01267
sace_e	Null	Null	Null	Null	Null	Null	Null	Null	Null	Null	Null	Null	sace_e_4917	sace_e_4918	sace_e_4919	sace_e_4920	sace_e_4921	sace_e_4922	sace_e_4923	sace_e_4924	Null	Null	Null	Null	Null	Null	Null	Null	Null	Null	Null
sace_e	Null	Null	Null	Null	Null	Null	Null	Null	Null	Null	Null	sace_e_4917	sace_e_4918	sace_e_4919	sace_e_4920	sace_e_4921	sace_e_4922	sace_e_4923	sace_e_4924	Null	Null	Null	Null	Null	Null	Null	Null	Null	Null	Null	Null
sapa	Null	Null	Null	Null	Null	Null	Null	Null	Null	Null	Null	Null	Null	Null	sapa_c415_24459	sapa_c415_24456	Null	Null	Null	Null	Null	Null	Null	Null	Null	Null	Null	Null	Null	Null	Null
sapa	Null	Null	Null	Null	Null	Null	Null	Null	Null	Null	Null	Null	sapa_c416_24454	sapa_c416_24450	sapa_c416_24441	sapa_c416_24437	Null	Null	Null	Null	Null	Null	Null	Null	Null	Null	Null	Null	Null	Null	Null
sami_a	Null	Null	Null	Null	Null	Null	Null	Null	Null	Null	Null	sami_a_c633_10642	sami_a_c633_10641	sami_a_c633_10637	sami_a_c633_10628	sami_a_c633_10615	sami_a_c633_10609	sami_a_c633_10608	Null	Null	Null	Null	Null	Null	Null	Null	Null	Null	Null	Null	Null
saba_a	Null	Null	Null	Null	Null	Null	Null	Null	Null	Null	Null	saba_a_479.1	saba_a_479.3	saba_a_479.4	saba_a_479.5	saba_a_479.6	Null	Null	Null	Null	Null	Null	Null	Null	Null	Null	Null	Null	Null	Null	Null
saba_a	Null	Null	Null	Null	Null	Null	Null	Null	Null	Null	Null	saba_a_673.1	saba_a_673.2	saba_a_673.4	saba_a_673.5	saba_a_673.6	saba_a_673.7	saba_a_673.8	saba_a_673.9	saba_a_673.10	saba_a_673.11	saba_a_673.12	saba_a_673.13	saba_a_673.14	saba_a_673.15	saba_a_673.16	saba_a_673.17	saba_a_673.18	saba_a_673.19	saba_a_673.20	saba_a_673.22

Homologs of ARN4 gene. The names of the adjacent genes are shown and the homologous neighbours are highlighted in the same colour.

Cluster R (lineage 11): homologs of C. albicans CaARN1 gene

caal_a	832	458	909	20922	908	907	906	905	904	903	902	901	899	900	897	caal_a_19.2179	20921	895	90	894	893	892	891	262	241	889	888	707	887	886	93
caaL_b	832	458	909	908	4815	907	906	905	904	903	902	901	899	900	897	caal_b_05897	895	90	894	893	892	891	262	241	889	888	707	887	886	93	930
cadu	912	911	832	458	909	908	907	906	905	904	903	902	901	899	897	cadu_22250	895	90	893	892	891	262	241	889	888	707	887	886	93	21146	931
catr	3223	3224	1596	168	167	147	4603	223	222	9026	1127	1197	902	901	899	catr_01806	895	90	893	892	891	262	241	889	887	886	93	9027	931	1240	274
capa	618	612	613	615	616	506	607	608	609	611	57	57	902	901	899	capa_02454	896	895	90	8549	1082	753	1127	1197	8550	345	301	418	3130	3131	3132
capa	612	613	615	616	506	607	608	609	611	57	57	902	901	899	896	capa_02455	895	90	8549	1082	753	1127	1197	8550	345	301	418	3130	3131	3132	1516
loel	614	13032	618	616	615	609	607	506	612	613	611	57	902	901	899	loel_02353	13031	895	90	13030	1082	13029	753	1127	1197	12929	13028	345	301	1040	1040
pist	6884	1930	1929	1928	1506	1925	15293	15235	190	1923	1922	90	895	899	897	pist_igi16606996	901	902	14827	1197	1127	753	1082	7692	1934	1933	2250	2249	2248	2247	2243

Homologs of C. albicans CaARN1 gene. The protein family classification of the adjacent genes is shown and the homologous neighbours are highlighted in the same colour.

caal_a	caal_a_19.2193	caal_a_19.2192	caal_a_19.2191.1	caal_a_19.2191	caal_a_19.2190	caal_a_19.2187	caal_a_19.2186	caal_a_19.2185	caal_a_19.2184	caal_a_19.2183
caaL_b	caal_b_05883	caal_b_05884	caal_b_05885	caal_b_05887	caal_b_05886	caal_b_05888	caal_b_05889	caal_b_05890	caal_b_05891	caal_b_05892
cadu	cadu_22110	cadu_22120	cadu_22130	cadu_22140	cadu_22150	cadu_22160	cadu_22170	cadu_22180	cadu_22190	cadu_22200
catr	catr_01792	catr_01793	catr_01794	catr_01795	catr_01796	catr_01797	catr_01798	catr_01799	catr_01800	catr_01801
capa	capa_02440	capa_02441	capa_02442	capa_02443	capa_02444	capa_02445	capa_02446	capa_02447	capa_02448	capa_02449
capa	capa_02441	capa_02442	capa_02443	capa_02444	capa_02445	capa_02446	capa_02447	capa_02448	capa_02449	capa_02450
loel	loel_02367	loel_02366	loel_02365	loel_02364	loel_02363	loel_02362	loel_02361	loel_02360	loel_02359	loel_02358
pist	pist_igi19141798	pist_igi19141799	pist_igi18293360	pist_igi19988270	pist_igi18293361	pist_igi20836968	pist_igi19988271	pist_igi19988272	pist_igi16606992	pist_igi16606993

caal_a	caal_a_19.2182	caal_a_19.2180	caal_a_19.2181	caal_a_19.2179.2	caal_a_19.2179	caal_a_19.2178.1	caal_a_19.2178	caal_a_19.2176	caal_a_19.2177	caal_a_19.2175
caaL_b	caal_b_05893	caal_b_05894	caal_b_05895	caal_b_05896	caal_b_05897	caal_b_05898	caal_b_05899	caal_b_05900	caal_b_05901	caal_b_05902
cadu	cadu_22210	cadu_22220	cadu_22230	cadu_22240	cadu_22250	cadu_22260	cadu_22270	cadu_22280	cadu_22290	cadu_22300
catr	catr_01802	catr_01803	catr_01804	catr_01805	catr_01806	catr_01807	catr_01808	catr_01809	catr_01810	catr_01811
capa	capa_02450	capa_02451	capa_02452	capa_02453	capa_02454	capa_02455	capa_02456	capa_02457	capa_02458	capa_02459
capa	capa_02451	capa_02452	capa_02453	capa_02454	capa_02455	capa_02456	capa_02457	capa_02458	capa_02459	capa_02460
loel	loel_02357	loel_02356	loel_02355	loel_02354	loel_02353	loel_02352	loel_02351	loel_02350	loel_02349	loel_02348
pist	pist_igi16606994	pist_igi20836969	pist_igi18293362	pist_igi16606995	pist_igi16606996	pist_igi16606997	pist_igi19141800	pist_igi16606998	pist_igi16606999	pist_igi20836970

caal_a	caal_a_19.2174	caal_a_19.2173	caal_a_19.2172	caal_a_19.2170	caal_a_19.2169	caal_a_19.2168.3	caal_a_19.2168	caal_a_19.2167	caal_a_19.2166	caal_a_19.2165
caaL_b	caal_b_05903	caal_b_05904	caal_b_05905	caal_b_05906	caal_b_05907	caal_b_05908	caal_b_05909	caal_b_05910	caal_b_05911	caal_b_05912
cadu	cadu_22310	cadu_22320	cadu_22330	cadu_22340	cadu_22350	cadu_22360	cadu_22370	cadu_22380	cadu_22390	cadu_22400
catr	catr_01812	catr_01813	catr_01814	catr_01815	catr_01816	catr_01817	catr_01818	catr_01819	catr_01820	catr_01821
capa	capa_02460	capa_02461	capa_02462	capa_02463	capa_02464	capa_02465	capa_02466	capa_02467	capa_02468	capa_02469
capa	capa_02461	capa_02462	capa_02463	capa_02464	capa_02465	capa_02466	capa_02467	capa_02468	capa_02469	capa_02470
loel	loel_02347	loel_02346	loel_02345	loel_02344	loel_02343	loel_02342	loel_02341	loel_02340	loel_02339	loel_02338
pist	pist_igi20836971	pist_igi20836972	pist_igi20836973	pist_igi19988273	pist_igi20836974	pist_igi19988274	pist_igi19141801	pist_igi16607000	pist_igi19988275	pist_igi18293363

Homologs of C. albicans CaARN1 gene. The names of the adjacent genes are shown and the homologous neighbours are highlighted in the same colour.

Cluster P (lineage 10): homologs of ARN3 gene

Sheet1

sace_a	0	0	0	0	0	11717	11717	11717	11717	15657	16641	1487	12790	468	3732	sace0e00330g	2295	82	1693	591	944	15707	2911	15706	876	3298	509	499	1407	1410	3173
sace_b	0	0	0	0	0	0	0	0	12787	16602	6077	465	468	16855	3732	sace_b_1e8_0089g	2295	82	1693	591	944	15707	16856	2911	15706	876	3298	509	16844	499	1407
sace_c	0	0	0	0	0	0	0	0	0	0	0	0	12790	468	3732	sace_c_05195	2295	82	1693	591	944	15707	2911	15706	876	3298	509	499	1407	1410	3173
sace_d	0	0	0	0	0	0	0	0	0	15506	16641	1487	12790	468	3732	sace_d_04406	2295	82	1693	591	944	15707	2911	15706	876	3298	509	499	1410	3173	12787
sace_e	0	0	0	0	0	0	0	0	0	15657	16641	1487	12790	468	3732	sace_e_1433	2295	82	1693	591	944	15707	2911	15706	876	3298	509	499	1407	1410	3173
sapa	0	0	0	0	0	0	0	0	0	0	0	0	0	16855	3732	sapa_c356_5984	2295	82	1693	591	944	15707	2911	15706	876	3298	509	16844	499	1407	1410
sami_a	0	0	0	0	0	0	0	0	0	0	0	468	24009	17820	3732	sami_a_c202_5644	2295	82	1693	591	944	15707	17819	2911	15706	876	3298	24008	509	16844	499
saba_a	0	0	0	22387	11328	90	3373	3374	468	4372	1448	1449	12790	468	3732	saba_a_676.52	2295	82	1693	591	944	15707	2911	15706	876	3298	509	499	1407	1410	3173
saba_b	0	0	0	0	0	0	0	0	0	0	0	0	0	468	3732	saba_b_c280_5966	2295	82	1693	591	944	15707	2911	15706	876	3298	509	22498	499	1407	1410
saca	945	22795	10807	10808	16122	5947	174	57	662	370	2466	616	618	1125	1995	saca_514.3	2594	6343	402	1857	6342	2583	0	0	0	0	0	0	0	0	0
saca	4260	5178	3278	354	354	775	181	10197	1523	4078	222	515	147	1902	5044	saca_705.26	4348	4097	1594	4096	939	938	937	16472	3092	2866	1452	3312	5974	935	16474
klpo	0	0	0	0	0	0	0	0	0	0	0	0	0	280	75	klpo_358.4	82	12001	21959	0	0	0	0	0	0	0	0	0	0	0	0
klpo	0	0	0	0	0	0	0	0	0	0	0	75	11977	11977	75	klpo_416.5	896	662	12022	473	2850	0	0	0	0	0	0	0	0	0	0
klpo	0	0	0	0	0	0	0	0	0	0	75	11977	11977	75	896	klpo_416.6	662	12022	473	2850	0	0	0	0	0	0	0	0	0	0	0
klpo	0	0	0	0	0	0	0	0	0	0	0	0	11659	940	1293	klpo_513.33	402	12076	2594	6342	2637	2961	618	616	2466	57	3657	3655	2189	190	5377
sakl	17404	2121	5046	242	2859	291	3018	4373	868	11886	4525	4293	17405	17406	1233	sakl0c12122g	790	1640	731	732	5222	182	1616	6217	2860	147	5221	2414	5220	23754	5219
sakl	3831	2878	5949	4669	4667	1748	147	1014	5950	17588	2125	12510	3046	151	3591	sakl0g06600g	4546	4545	17589	5246	3400	3401	23774	4306	4662	5589	10470	10471	147	716	6280
klwa	559	5475	418	1027	12520	991	1954	280	280	280	1820	2894	2898	2907	2929	klwa_018-snap.54	3500	921	12521	2254	2322	3640	29	0	0	0	0	0	0	0	0
klwa	4335	1881	242	5046	2859	291	3018	4373	868	11886	4525	4293	22039	12140	1233	klwa_021-snap.20	790	391	731	732	5222	182	1616	6217	2860	147	2414	5220	22040	5219	2379
klth	1977	1975	242	5046	2859	291	3018	4373	868	11886	4525	4293	21987	12140	1233	klth0a01562g	790	1640	731	732	5222	182	1616	6217	2860	147	5221	2414	5220	21986	5219
klth	559	5475	418	1027	12520	991	3307	1954	280	280	1820	2894	2898	2907	2929	klth0g09504g	3500	921	12521	2254	2322	3640	29	325	2807	2697	2724	2725	2699	10365	2700
catr	2681	2683	1988	8875	2306	2308	2309	482	2310	2311	2312	8876	8877	2314	2317	catr_00077	896	323	1659	2337	2336	2334	2333	2332	2331	2330	2329	2334	1638	3346	2326

catr	2683	1988	8875	2306	2308	2309	482	2310	2311	2312	8876	8877	2314	2317	896	catr_00078	323	1659	2337	2336	2334	2333	2332	2331	2330	2329	2334	1638	3346	2326	2327
capa	0	0	0	0	0	0	0	0	0	0	0	0	1157	21399	1028	capa_00064	0	0	0	0	0	0	0	0	0	0	0	0	0	0	0
capa	1668	1661	8568	1654	1655	1300	1648	1650	1652	1651	1647	1156	1157	1159	1160	capa_02689	896	0	0	0	0	0	0	0	0	0	0	0	0	0	0
capa	1661	8568	1654	1655	1300	1648	1650	1652	1651	1647	1156	1157	1159	1160	896	capa_02690	0	0	0	0	0	0	0	0	0	0	0	0	0	0	0
cagu	6906	6907	468	2257	6373	2987	6908	2739	579	468	6909	6910	6911	6912	340	cagu_03941	6524	4369	6913	468	6524	0	0	0	0	0	0	0	0	0	0
cagu	1902	1897	7130	1898	1898	1900	7129	1647	1651	1652	1894	1895	1896	1028	1028	cagu_05301	280	1145	1146	1148	1370	1369	1893	468	1880	525	1882	1883	1586	1586	1586
deha	1192	9599	1903	1902	1897	1898	1900	9598	1647	1651	1652	1894	1895	1896	1028	deha2c05390g	1145	1146	1148	7286	1370	1369	1893	468	1880	525	1882	1883	1586	1586	147
pist	2473	2480	2478	1666	1667	1654	1655	1300	1648	15023	1650	1652	1651	1647	1028	pist_igi18293386	280	1145	1146	1148	7286	1150	1151	1152	1153	1154	1156	1157	1158	1159	1160
calu	4037	4037	525	1823	3956	3957	3958	7490	3961	1063	3969	3966	1063	3965	3964	calu_01670	1192	2899	82	1092	3564	4356	7491	4355	147	7492	2025	2023	1206	1365	7493
pipa	118	3180	3527	1579	781	3923	2042	1661	4682	4681	145	14378	2090	662	3931	pipa_3g03640	4400	14379	750	2382	4684	7014	14380	14381	14382	54	14383	1487	754	2397	14384

Homologs of ARN3 gene. The protein family classification of the adjacent genes is shown and the homologous neighbours are highlighted in the same colour.

sace_a	Null	Null	Null	Null	Null	sace0e00110g	sace0e00132g	sace0e00154g	sace0e00176g	sace0e00198g	sace0e00220g	sace0e00242g	sace0e00264g	sace0e00286g	sace0e00308g	sace0e00330g	sace0e00352g	sace0e00374g	sace0e00396g	sace0e00418g	sace0e00440g	sace0e00462g	sace0e00484g	sace0e00506g	sace0e00528g	sace0e00550g	sace0e00594g	sace0e00616g	sace0e00638g	sace0e00660g	sace0e00682g
sace_b	Null	Null	Null	Null	Null	Null	Null	Null	sace_b_1e8_0001g	sace_b_1e8_0012g	sace_b_1e8_0023g	sace_b_1e8_0034g	sace_b_1e8_0045g	sace_b_1e8_0056g	sace_b_1e8_0078g	sace_b_1e8_0089g	sace_b_1e8_0100g	sace_b_1e8_0111g	sace_b_1e8_0122g	sace_b_1e8_0133g	sace_b_1e8_0144g	sace_b_1e8_0155g	sace_b_1e8_0166g	sace_b_1e8_0177g	sace_b_1e8_0188g	sace_b_1e8_0199g	sace_b_1e8_0210g	sace_b_1e8_0221g	sace_b_1e8_0232g	sace_b_1e8_0243g	sace_b_1e8_0254g
sace_c	Null	Null	Null	Null	Null	Null	Null	Null	Null	Null	Null	Null	sace_c_05194	sace_c_05197	sace_c_05210	sace_c_05195	sace_c_05202	sace_c_05196	sace_c_05198	sace_c_05207	sace_c_05201	sace_c_05213	sace_c_05205	sace_c_05211	sace_c_05209	sace_c_05200	sace_c_05203	sace_c_05199	sace_c_05206	sace_c_05208	sace_c_05204
sace_d	Null	Null	Null	Null	Null	Null	Null	Null	Null	sace_d_04399	sace_d_04401	sace_d_04402	sace_d_04403	sace_d_04404	sace_d_04405	sace_d_04406	sace_d_04407	sace_d_04408	sace_d_04409	sace_d_04410	sace_d_04411	sace_d_04412	sace_d_04413	sace_d_04414	sace_d_04415	sace_d_04416	sace_d_04417	sace_d_04418	sace_d_04420	sace_d_04421	sace_d_04422
sace_e	Null	Null	Null	Null	Null	Null	Null	Null	Null	sace_e_1427	sace_e_1428	sace_e_1429	sace_e_1430	sace_e_1431	sace_e_1432	sace_e_1433	sace_e_1434	sace_e_1435	sace_e_1436	sace_e_1437	sace_e_1438	sace_e_1439	sace_e_1440	sace_e_1441	sace_e_1442	sace_e_1443	sace_e_1444	sace_e_1445	sace_e_1446	sace_e_1447	sace_e_1448
sapa	Null	Null	Null	Null	Null	Null	Null	Null	Null	Null	Null	Null	Null	sapa_c356_5991	sapa_c356_5989	sapa_c356_5984	sapa_c356_5980	sapa_c356_5976	sapa_c356_5973	sapa_c356_5969	sapa_c356_5962	sapa_c356_5959	sapa_c356_5949	sapa_c356_5947	sapa_c356_5943	sapa_c356_5936	sapa_c356_5934	sapa_c356_5933	sapa_c356_5930	sapa_c356_5924	sapa_c356_5921
sami_a	Null	Null	Null	Null	Null	Null	Null	Null	Null	Null	Null	sami_a_c202_5657	sami_a_c202_5652	sami_a_c202_5648	sami_a_c202_5647	sami_a_c202_5644	sami_a_c202_5641	sami_a_c202_5637	sami_a_c202_5633	sami_a_c202_5629	sami_a_c202_5620	sami_a_c202_5618	sami_a_c202_5617	sami_a_c202_5610	sami_a_c202_5607	sami_a_c202_5603	sami_a_c202_5596	sami_a_c202_5594	sami_a_c202_5592	sami_a_c202_5591	sami_a_c202_5587
saba_a	Null	Null	Null	saba_a_676.66	saba_a_676.64	saba_a_676.63	saba_a_676.62	saba_a_676.61	saba_a_676.59	saba_a_676.58	saba_a_676.57	saba_a_676.56	saba_a_676.55	saba_a_676.54	saba_a_676.53	saba_a_676.52	saba_a_676.51	saba_a_676.50	saba_a_676.49	saba_a_676.48	saba_a_676.47	saba_a_676.46	saba_a_676.45	saba_a_676.44	saba_a_676.43	saba_a_676.42	saba_a_676.41	saba_a_676.40	saba_a_676.39	saba_a_676.38	saba_a_676.37
saba_b	Null	Null	Null	Null	Null	Null	Null	Null	Null	Null	Null	Null	Null	saba_b_c280_5958	saba_b_c280_5965	saba_b_c280_5966	saba_b_c280_5970	saba_b_c280_5973	saba_b_c280_5977	saba_b_c280_5984	saba_b_c280_5990	saba_b_c280_5998	saba_b_c280_6002	saba_b_c280_6007	saba_b_c280_6014	saba_b_c280_6018	saba_b_c280_6022	saba_b_c280_6023	saba_b_c280_6026	saba_b_c280_6030	saba_b_c280_6033
saca	saca_574.2	saca_574.3	saca_574.4	saca_574.5	saca_574.5d	saca_574.6	saca_574.7	saca_574.8	saca_574.9	saca_574.9d	saca_464.3d	saca_464.3	saca_464.2	saca_464.1	saca_514.2	saca_514.3	saca_514.4	saca_514.5	saca_514.6	saca_514.7	saca_514.8	saca_514.9	Null	Null	Null	Null	Null	Null	Null	Null	Null
saca	saca_705.12	saca_705.13	saca_705.14	saca_705.15	saca_705.15d	saca_705.16	saca_705.17	saca_705.18	saca_705.19	saca_705.20	saca_705.21	saca_705.22	saca_705.23	saca_705.24	saca_705.25	saca_705.26	saca_705.27	saca_705.28	saca_705.29	saca_705.30	saca_705.31	saca_705.32	saca_705.33	saca_705.34	saca_705.35	saca_705.36	saca_705.37	saca_705.38	saca_705.39	saca_705.40	saca_705.41
klpo	Null	Null	Null	Null	Null	Null	Null	Null	Null	Null	Null	Null	Null	klpo_358.7	klpo_358.5	klpo_358.4	klpo_358.3	klpo_358.2	klpo_358.1	Null	Null	Null	Null	Null	Null	Null	Null	Null	Null	Null	Null
klpo	Null	Null	Null	Null	Null	Null	Null	Null	Null	Null	Null	klpo_416.1	klpo_416.2	klpo_416.3	klpo_416.4	klpo_416.5	klpo_416.6	klpo_416.7	klpo_416.8	klpo_416.9	klpo_416.10	Null	Null	Null	Null	Null	Null	Null	Null	Null	Null
klpo	Null	Null	Null	Null	Null	Null	Null	Null	Null	Null	klpo_416.1	klpo_416.2	klpo_416.3	klpo_416.4	klpo_416.5	klpo_416.6	klpo_416.7	klpo_416.8	klpo_416.9	klpo_416.10	Null	Null	Null	Null	Null	Null	Null	Null	Null	Null	Null
klpo	Null	Null	Null	Null	Null	Null	Null	Null	Null	Null	Null	Null	klpo_513.36	klpo_513.35	klpo_513.34	klpo_513.33	klpo_513.32	klpo_513.31	klpo_513.30	klpo_513.29	klpo_513.28	klpo_513.27	klpo_513.26	klpo_513.25	klpo_513.24	klpo_513.23	klpo_513.22	klpo_513.21	klpo_513.20	klpo_513.19	klpo_513.18
sakl	sakl0c11638g	sakl0c11660g	sakl0c11682g	sakl0c11704g	sakl0c11726g	sakl0c11748g	sakl0c11770g	sakl0c11792g	sakl0c11814g	sakl0c11968g	sakl0c11990g	sakl0c12012g	sakl0c12034g	sakl0c12056g	sakl0c12100g	sakl0c12122g	sakl0c12144g	sakl0c12210g	sakl0c12232g	sakl0c12254g	sakl0c12276g	sakl0c12298g	sakl0c12320g	sakl0c12342g	sakl0c12364g	sakl0c12386g	sakl0c12408g	sakl0c12430g	sakl0c12452g	sakl0c12474g	sakl0c12496g
sakl	sakl0g06270g	sakl0g06292g	sakl0g06314g	sakl0g06336g	sakl0g06358g	sakl0g06380g	sakl0g06402g	sakl0g06424g	sakl0g06446g	sakl0g06468g	sakl0g06490g	sakl0g06512g	sakl0g06534g	sakl0g06556g	sakl0g06578g	sakl0g06600g	sakl0g06644g	sakl0g06666g	sakl0g06688g	sakl0g06754g	sakl0g06776g	sakl0g06798g	sakl0g06820g	sakl0g06842g	sakl0g06864g	sakl0g06886g	sakl0g06908g	sakl0g06930g	sakl0g06952g	sakl0g06974g	sakl0g06996g
klwa	klwa_018-snap.39	klwa_018-snap.40	klwa_018-snap.41	klwa_018-snap.42	klwa_018-snap.43	klwa_018-snap.44	klwa_018-snap.45	klwa_018-snap.46	klwa_018-snap.47	klwa_018-snap.48	klwa_018-snap.49	klwa_018-snap.50	klwa_018-snap.51	klwa_018-snap.52	klwa_018-snap.53	klwa_018-snap.54	klwa_018-snap.55	klwa_018-snap.56	klwa_018-snap.57	klwa_018-snap.58	klwa_018-snap.59	klwa_018-snap.60	klwa_018-snap.61	Null	Null	Null	Null	Null	Null	Null	Null
klwa	klwa_021-snap.5	klwa_021-snap.6	klwa_021-snap.7	klwa_021-snap.8	klwa_021-snap.9	klwa_021-snap.10	klwa_021-snap.11	klwa_021-snap.12	klwa_021-snap.13	klwa_021-snap.14	klwa_021-snap.15	klwa_021-snap.16	klwa_021-snap.17	klwa_021-snap.18	klwa_021-snap.19	klwa_021-snap.20	klwa_021-snap.21	klwa_021-snap.22	klwa_021-snap.23	klwa_021-snap.24	klwa_021-snap.25	klwa_021-snap.26	klwa_021-snap.27	klwa_021-snap.28	klwa_021-snap.29	klwa_021-snap.30	klwa_021-snap.31	klwa_021-snap.32	klwa_021-snap.33	klwa_021-snap.34	klwa_021-snap.35
klth	klth0a01958g	klth0a01936g	klth0a01914g	klth0a01892g	klth0a01870g	klth0a01848g	klth0a01826g	klth0a01804g	klth0a01782g	klth0a01716g	klth0a01694g	klth0a01672g	klth0a01650g	klth0a01628g	klth0a01584g	klth0a01562g	klth0a01540g	klth0a01496g	klth0a01474g	klth0a01452g	klth0a01430g	klth0a01408g	klth0a01386g	klth0a01364g	klth0a01342g	klth0a01320g	klth0a01298g	klth0a01276g	klth0a01254g	klth0a01232g	klth0a01210g
klth	klth0g09152g	klth0g09174g	klth0g09196g	klth0g09218g	klth0g09240g	klth0g09262g	klth0g09284g	klth0g09306g	klth0g09350g	klth0g09372g	klth0g09394g	klth0g09416g	klth0g09438g	klth0g09460g	klth0g09482g	klth0g09504g	klth0g09526g	klth0g09548g	klth0g09570g	klth0g09592g	klth0g09614g	klth0g09636g	klth0g09658g	klth0g09680g	klth0g09702g	klth0g09724g	klth0g09746g	klth0g09768g	klth0g09790g	klth0g09812g	klth0g09834g
catr	catr_00062	catr_00063	catr_00064	catr_00065	catr_00066	catr_00067	catr_00068	catr_00069	catr_00070	catr_00071	catr_00072	catr_00073	catr_00074	catr_00075	catr_00076	catr_00077	catr_00078	catr_00079	catr_00080	catr_00081	catr_00082	catr_00083	catr_00084	catr_00085	catr_00086	catr_00087	catr_00088	catr_00089	catr_00090	catr_00091	catr_00092
catr	catr_00063	catr_00064	catr_00065	catr_00066	catr_00067	catr_00068	catr_00069	catr_00070	catr_00071	catr_00072	catr_00073	catr_00074	catr_00075	catr_00076	catr_00077	catr_00078	catr_00079	catr_00080	catr_00081	catr_00082	catr_00083	catr_00084	catr_00085	catr_00086	catr_00087	catr_00088	catr_00089	catr_00090	catr_00091	catr_00092	catr_00093
capa	Null	Null	Null	Null	Null	Null	Null	Null	Null	Null	Null	Null	capa_00067	capa_00066	capa_00065	capa_00064	Null	Null	Null	Null	Null	Null	Null	Null	Null	Null	Null	Null	Null	Null	Null
capa	capa_02674	capa_02675	capa_02676	capa_02677	capa_02678	capa_02679	capa_02680	capa_02681	capa_02682	capa_02683	capa_02684	capa_02685	capa_02686	capa_02687	capa_02688	capa_02689	capa_02690	Null	Null	Null	Null	Null	Null	Null	Null	Null	Null	Null	Null	Null	Null
capa	capa_02675	capa_02676	capa_02677	capa_02678	capa_02679	capa_02680	capa_02681	capa_02682	capa_02683	capa_02684	capa_02685	capa_02686	capa_02687	capa_02688	capa_02689	capa_02690	Null	Null	Null	Null	Null	Null	Null	Null	Null	Null	Null	Null	Null	Null	Null
cagu	cagu_03926	cagu_03927	cagu_03928	cagu_03929	cagu_03930	cagu_03931	cagu_03932	cagu_03933	cagu_03934	cagu_03935	cagu_03936	cagu_03937	cagu_03938	cagu_03939	cagu_03940	cagu_03941	cagu_03942	cagu_03943	cagu_03944	cagu_03945	cagu_03946	Null	Null	Null	Null	Null	Null	Null	Null	Null	Null
cagu	cagu_05316	cagu_05315	cagu_05314	cagu_05313	cagu_05312	cagu_05311	cagu_05310	cagu_05309	cagu_05308	cagu_05307	cagu_05306	cagu_05305	cagu_05304	cagu_05303	cagu_05302	cagu_05301	cagu_05300	cagu_05299	cagu_05298	cagu_05297	cagu_05296	cagu_05295	cagu_05294	cagu_05293	cagu_05292	cagu_05291	cagu_05290	cagu_05289	cagu_05288	cagu_05287	cagu_05286
deha	deha2c05720g	deha2c05698g	deha2c05676g	deha2c05654g	deha2c05632g	deha2c05610g	deha2c05588g	deha2c05566g	deha2c05544g	deha2c05522g	deha2c05500g	deha2c05478g	deha2c05456g	deha2c05434g	deha2c05412g	deha2c05390g	deha2c05368g	deha2c05346g	deha2c05324g	deha2c05302g	deha2c05280g	deha2c05258g	deha2c05236g	deha2c05214g	deha2c05192g	deha2c05170g	deha2c05148g	deha2c05126g	deha2c05104g	deha2c05082g	deha2c05060g
pist	pist_igi19141817	pist_igi16607065	pist_igi16607066	pist_igi16607067	pist_igi20836992	pist_igi19988291	pist_igi16607068	pist_igi16607069	pist_igi19988292	pist_igi16607070	pist_igi16607071	pist_igi20836993	pist_igi20836994	pist_igi18293385	pist_igi19141818	pist_igi18293386	pist_igi16607072	pist_igi19988293	pist_igi18293387	pist_igi16607073	pist_igi16607074	pist_igi19141819	pist_igi20836995	pist_igi16607075	pist_igi18293388	pist_igi16607076	pist_igi16607077	pist_igi16607078	pist_igi16607079	pist_igi16607080	pist_igi18293389
calu	calu_01655	calu_01656	calu_01657	calu_01658	calu_01659	calu_01660	calu_01661	calu_01662	calu_01663	calu_01664	calu_01665	calu_01666	calu_01667	calu_01668	calu_01669	calu_01670	calu_01671	calu_01672	calu_01673	calu_01674	calu_01675	calu_01676	calu_01677	calu_01678	calu_01679	calu_01680	calu_01681	calu_01682	calu_01683	calu_01684	calu_01685
pipa	pipa_3g03560	pipa_3g03565	pipa_3g03570	pipa_3g03575	pipa_3g03580	pipa_3g03585	pipa_3g03590	pipa_3g03595	pipa_3g03600	pipa_3g03605	pipa_3g03610	pipa_3g03620	pipa_3g03625	pipa_3g03630	pipa_3g03635	pipa_3g03640	pipa_3g03645	pipa_3g03650	pipa_3g03655	pipa_3g03660	pipa_3g03665	pipa_3g03675	pipa_3g03680	pipa_3g03685	pipa_3g03690	pipa_3g03695	pipa_3g03700	pipa_3g03705	pipa_3g03715	pipa_3g03720	pipa_3g03725

Homologs of ARN3 gene. The names of the adjacent genes are shown and the homologous neighbours are highlighted in the same colour.

Cluster T (lineage 13): homologs of ARN1 and ARN2 genes

sace_a	0	0	0	0	0	0	0	0	0	0	0	11717	11717	22933	15506	sace0h00198g	12787	16671	15506	15506	896	4374	5240	1638	57	5239	996	3290	3874	4015	4012
sace_a	0	0	0	0	0	0	11717	11717	22933	15506	896	12787	16671	15506	15506	sace0h00308g	4374	5240	1638	57	5239	996	3290	3874	4015	4012	15905	4326	5237	15335	1778
sace_b	0	0	0	0	0	0	0	0	0	0	0	0	0	0	15506	sace_b_1h21_0023g	16962	12787	16963	16671	15506	896	4374	5240	1638	57	16964	5239	996	3290	3874
sace_b	0	0	0	0	0	0	0	0	15506	896	16962	12787	16963	16671	15506	sace_b_1h21_0100g	4374	5240	1638	57	16964	5239	996	3290	3874	16965	4015	4012	15905	4326	5237
sace_c	0	0	0	0	0	0	0	0	0	0	0	0	0	16671	15506	sace_c_03075	4374	5240	1638	57	5239	0	0	0	0	0	0	0	0	0	0
sace_d	0	0	0	0	0	0	0	0	0	0	0	0	0	16671	15506	sace_d_04665	4374	1638	57	5239	996	3290	3874	4015	4012	15905	4326	5237	15335	3810	5093
sace_e	0	0	0	0	0	0	0	0	0	0	0	0	0	16671	15506	sace_e_2345	4374	5240	1638	57	5239	996	3290	3874	4015	4012	15905	4326	5237	15335	17241
sapa	0	0	0	0	0	0	0	0	0	0	0	0	0	16671	15506	sapa_c38_10565	4374	5240	1638	57	5239	996	3290	3874	4015	4012	15905	4326	5237	15335	1778
sami_a	0	0	0	0	0	0	0	0	0	0	0	0	0	0	0	sami_a_c322_9452	4374	5240	1638	57	5239	996	3290	3874	4015	4012	15905	4326	5237	15335	1778
saba_a	0	0	0	0	0	0	0	0	0	0	0	605	1566	15448	1137	saba_a_602.6	4374	5240	1638	57	5239	996	3290	3874	4015	4012	0	0	0	0	0
saba_b	0	0	0	0	0	0	0	0	0	0	0	605	1566	15448	1137	saba_b_c633_10089	4374	5240	1638	57	5239	996	3290	3874	4015	4012	15905	0	0	0	0
saku	0	0	0	0	0	0	0	0	0	0	0	0	0	12787	15506	saku_c1947.4	4374	5240	0	0	0	0	0	0	0	0	0	0	0	0	0
saca	2619	1857	3898	1467	16312	748	2977	3445	6084	982	6055	4355	2904	2859	5446	saca_663.28	16313	0	0	0	0	0	0	0	0	0	0	0	0	0	0
cagl	4546	3591	151	1014	1748	4669	2878	3831	5244	5243	5242	5241	4000	499	1407	cagl0e04092g	4374	4690	5240	1638	57	5239	996	3290	5098	3874	3766	4015	5238	4012	4326
zyro	4658	4655	2570	11411	82	2571	2572	5230	548	5231	3602	3601	20831	20832	199	zyro0g07414g	2682	2357	10019	4374	4690	20833	5240	1638	57	5239	996	3290	5098	3874	20834
sakl	0	0	0	0	0	0	0	0	0	0	0	0	0	0	17294	sakl0e00132g	17462	291	2042	12353	33	17463	17464	2121	124	2908	10316	3237	4949	57	3298
sakl	0	0	0	0	0	0	0	0	0	0	0	0	0	0	0	sakl0f00110g	291	2042	236	5582	1579	2682	465	17516	17517	4522	12627	11088	881	870	4996
sakl	6734	1099	314	2944	2937	17519	170	171	2941	2940	17518	4234	4249	4250	4202	sakl0f00792g	1600	4210	3675	262	6948	4995	2376	2371	2377	12075	5583	1555	4996	870	881
sakl	17582	2605	11222	2604	11223	3475	663	10338	3479	17583	3484	314	23772	17584	167	sakl0g03300g	167	5700	1751	348	2354	468	165	4252	2356	1151	1644	5699	2574	1766	1765
klwa	0	0	0	0	0	0	0	0	0	0	0	0	11717	12628	10910	klwa_001-snap.4	11132	8021	465	3496	82	2899	1192	1092	4241	104	190	2501	4567	2088	2537
klwa	0	0	0	0	0	0	0	0	0	0	0	0	0	0	0	klwa_265-snap.3	12218	468	0	0	0	0	0	0	0	0	0	0	0	0	0
klwa	0	0	0	0	0	0	0	0	0	0	0	0	0	11717	12772	klwa_331-snap.3	0	0	0	0	0	0	0	0	0	0	0	0	0	0	0

klth	63	1883	64	12168	4442	3064	5100	3375	1429	1604	466	2619	3496	12169	340	klth0a07964g	5146	1275	33	0	0	0	0	0	0	0	0	0	0	0	0
klth	262	340	6964	7616	236	9576	2611	12215	12215	12216	12217	11717	3888	340	11717	klth0b10450g	12218	6991	0	0	0	0	0	0	0	0	0	0	0	0	0
klth	0	0	0	0	0	0	0	0	0	0	0	0	0	0	0	klth0d00110g	11717	1212	12276	12277	277	1566	12205	465	663	12278	468	896	12279	2686	468
klth	0	0	0	896	11717	1212	12276	12277	277	1566	12205	465	663	12278	468	klth0d00506g	12279	2686	468	2388	2412	5121	12280	677	4026	5408	5823	4020	4022	1966	5821
klth	1479	3444	6258	10518	24	21	22003	819	820	12346	12347	2920	322	10931	2027	klth0d18150g	75	0	0	0	0	0	0	0	0	0	0	0	0	0	0
klla	2173	82	3583	10497	5477	428	525	10894	151	10227	957	5605	10893	2302	4241	klla0a10439g	6734	6365	5582	10892	3496	4170	2560	5374	747	4699	6343	402	1857	57	174
klla	0	0	0	0	0	0	0	0	0	234	465	10828	10829	10993	10994	klla0c00220g	10995	1566	286	4296	10996	10997	10998	764	4999	3894	3918	21862	2050	146	10999
klla	2914	11088	6964	262	11089	340	7616	11090	4372	6680	11091	11092	11093	11094	11095	klla0c19272g	75	11096	1212	11097	11098	75	11099	0	0	0	0	0	0	0	0
klla	771	6135	2473	513	4586	3714	3715	525	2305	1594	124	199	5266	75	456	klla0e14609g	527	468	407	1659	2201	2206	3681	147	231	2197	5252	2013	708	1954	694
cagu	0	0	0	0	0	0	0	0	0	0	0	0	468	6524	6672	cagu_02298	75	6673	6673	75	57	147	4465	1184	1560	1561	1562	1564	6674	1861	4013

Homologs of ARN1 and ARN2 genes. The protein family classification of the adjacent genes is shown and the homologous neighbours are highlighted in the same colour.

sace_a	Null	Null	Null	Null	Null	Null	Null	Null	Null	Null	sace0h00110g	sace0h00132g	sace0h00154g	sace0h00176g	sace0h00198g	sace0h00220g	sace0h00242g	sace0h00264g	sace0h00286g	sace0h00308g	sace0h00330g	sace0h00352g	sace0h00374g	sace0h00396g	sace0h00418g	sace0h00440g	sace0h00462g	sace0h00484g	sace0h00506g	sace0h00528g
sace_a	Null	Null	Null	Null	Null	sace0h00110g	sace0h00132g	sace0h00154g	sace0h00176g	sace0h00198g	sace0h00220g	sace0h00242g	sace0h00264g	sace0h00286g	sace0h00308g	sace0h00330g	sace0h00352g	sace0h00374g	sace0h00396g	sace0h00418g	sace0h00440g	sace0h00462g	sace0h00484g	sace0h00506g	sace0h00528g	sace0h00550g	sace0h00572g	sace0h00594g	sace0h00616g	sace0h00638g
sace_b	Null	Null	Null	Null	Null	Null	Null	Null	Null	Null	Null	Null	Null	sace_b_1h21_0012g	sace_b_1h21_0023g	sace_b_1h21_0034g	sace_b_1h21_0045g	sace_b_1h21_0056g	sace_b_1h21_0067g	sace_b_1h21_0078g	sace_b_1h21_0100g	sace_b_1h21_0111g	sace_b_1h21_0122g	sace_b_1h21_0144g	sace_b_1h21_0155g	sace_b_1h21_0166g	sace_b_1h21_0177g	sace_b_1h21_0188g	sace_b_1h21_0199g	sace_b_1h21_0210g
sace_b	Null	Null	Null	Null	Null	Null	Null	sace_b_1h21_0012g	sace_b_1h21_0023g	sace_b_1h21_0034g	sace_b_1h21_0045g	sace_b_1h21_0056g	sace_b_1h21_0067g	sace_b_1h21_0078g	sace_b_1h21_0100g	sace_b_1h21_0111g	sace_b_1h21_0122g	sace_b_1h21_0144g	sace_b_1h21_0155g	sace_b_1h21_0166g	sace_b_1h21_0177g	sace_b_1h21_0188g	sace_b_1h21_0199g	sace_b_1h21_0210g	sace_b_1h21_0221g	sace_b_1h21_0232g	sace_b_1h21_0243g	sace_b_1h21_0254g	sace_b_1h21_0265g	sace_b_1h21_0276g
sace_c	Null	Null	Null	Null	Null	Null	Null	Null	Null	Null	Null	Null	sace_c_03076	sace_c_03070	sace_c_03075	sace_c_03077	sace_c_03074	sace_c_03069	sace_c_03071	sace_c_03073	Null	Null	Null	Null	Null	Null	Null	Null	Null	Null
sace_d	Null	Null	Null	Null	Null	Null	Null	Null	Null	Null	Null	Null	sace_d_04663	sace_d_04664	sace_d_04665	sace_d_04666	sace_d_04668	sace_d_04669	sace_d_04670	sace_d_04671	sace_d_04672	sace_d_04673	sace_d_04674	sace_d_04675	sace_d_04676	sace_d_04677	sace_d_04678	sace_d_04679	sace_d_04681	sace_d_04682
sace_e	Null	Null	Null	Null	Null	Null	Null	Null	Null	Null	Null	Null	sace_e_2343	sace_e_2344	sace_e_2345	sace_e_2346	sace_e_2347	sace_e_2348	sace_e_2349	sace_e_2350	sace_e_2351	sace_e_2352	sace_e_2353	sace_e_2354	sace_e_2355	sace_e_2356	sace_e_2357	sace_e_2358	sace_e_2360	sace_e_2359
sapa	Null	Null	Null	Null	Null	Null	Null	Null	Null	Null	Null	Null	sapa_c38_10580	sapa_c38_10576	sapa_c38_10565	sapa_c38_10561	sapa_c38_10556	sapa_c38_10552	sapa_c38_10546	sapa_c38_10543	sapa_c38_10537	sapa_c38_10528	sapa_c38_10526	sapa_c38_10516	sapa_c38_10511	sapa_c38_10505	sapa_c38_10503	sapa_c38_10501	sapa_c38_10497	sapa_c38_10487
sami_a	Null	Null	Null	Null	Null	Null	Null	Null	Null	Null	Null	Null	Null	Null	sami_a_c322_9452	sami_a_c322_9460	sami_a_c322_9464	sami_a_c322_9469	sami_a_c322_9471	sami_a_c322_9478	sami_a_c322_9483	sami_a_c322_9487	sami_a_c322_9494	sami_a_c322_9496	sami_a_c322_9504	sami_a_c322_9513	sami_a_c322_9516	sami_a_c322_9522	sami_a_c322_9526	sami_a_c322_9532
saba_a	Null	Null	Null	Null	Null	Null	Null	Null	Null	Null	saba_a_602.1	saba_a_602.2	saba_a_602.4	saba_a_602.5	saba_a_602.6	saba_a_602.7	saba_a_602.8	saba_a_602.9	saba_a_602.10	saba_a_602.11	saba_a_602.12	saba_a_602.13	saba_a_602.14	saba_a_602.15	saba_a_602.16	Null	Null	Null	Null	Null
saba_b	Null	Null	Null	Null	Null	Null	Null	Null	Null	Null	saba_b_c633_10043	saba_b_c633_10048	saba_b_c633_10058	saba_b_c633_10074	saba_b_c633_10089	saba_b_c633_10095	saba_b_c633_10097	saba_b_c633_10102	saba_b_c633_10104	saba_b_c633_10114	saba_b_c633_10119	saba_b_c633_10121	saba_b_c633_10128	saba_b_c633_10134	saba_b_c633_10144	saba_b_c633_10159	Null	Null	Null	Null
saku	Null	Null	Null	Null	Null	Null	Null	Null	Null	Null	Null	Null	saku_c1947.1	saku_c1947.2	saku_c1947.4	saku_c1947.5	saku_c1947.6	Null	Null	Null	Null	Null	Null	Null	Null	Null	Null	Null	Null	Null
saca	saca_663.14	saca_663.15	saca_663.16	saca_663.17	saca_663.18	saca_663.19	saca_663.20	saca_663.21	saca_663.22	saca_663.23	saca_663.24	saca_663.25	saca_663.26	saca_663.27	saca_663.28	saca_663.29	Null	Null	Null	Null	Null	Null	Null	Null	Null	Null	Null	Null	Null	Null
cagl	cagl0e04422g	cagl0e04400g	cagl0e04378g	cagl0e04356g	cagl0e04334g	cagl0e04312g	cagl0e04290g	cagl0e04268g	cagl0e04246g	cagl0e04224g	cagl0e04180g	cagl0e04158g	cagl0e04136g	cagl0e04114g	cagl0e04092g	cagl0e04070g	cagl0e04048g	cagl0e04026g	cagl0e04004g	cagl0e03982g	cagl0e03960g	cagl0e03938g	cagl0e03916g	cagl0e03894g	cagl0e03872g	cagl0e03850g	cagl0e03828g	cagl0e03806g	cagl0e03784g	cagl0e03762g
zyro	zyro0g07106g	zyro0g07128g	zyro0g07150g	zyro0g07172g	zyro0g07194g	zyro0g07216g	zyro0g07238g	zyro0g07260g	zyro0g07282g	zyro0g07304g	zyro0g07326g	zyro0g07348g	zyro0g07370g	zyro0g07392g	zyro0g07414g	zyro0g07436g	zyro0g07458g	zyro0g07480g	zyro0g07502g	zyro0g07524g	zyro0g07546g	zyro0g07568g	zyro0g07590g	zyro0g07612g	zyro0g07634g	zyro0g07656g	zyro0g07700g	zyro0g07722g	zyro0g07744g	zyro0g07766g
sakl	Null	Null	Null	Null	Null	Null	Null	Null	Null	Null	Null	Null	Null	sakl0e00110g	sakl0e00132g	sakl0e00154g	sakl0e00176g	sakl0e00242g	sakl0e00264g	sakl0e00286g	sakl0e00396g	sakl0e00528g	sakl0e00616g	sakl0e00638g	sakl0e00660g	sakl0e00682g	sakl0e00704g	sakl0e00726g	sakl0e00748g	sakl0e00770g
sakl	Null	Null	Null	Null	Null	Null	Null	Null	Null	Null	Null	Null	Null	Null	sakl0f00110g	sakl0f00132g	sakl0f00176g	sakl0f00198g	sakl0f00242g	sakl0f00286g	sakl0f00308g	sakl0f00330g	sakl0f00352g	sakl0f00374g	sakl0f00396g	sakl0f00418g	sakl0f00440g	sakl0f00462g	sakl0f00484g	sakl0f00506g
sakl	sakl0f01100g	sakl0f01078g	sakl0f01056g	sakl0f01034g	sakl0f01012g	sakl0f00990g	sakl0f00968g	sakl0f00946g	sakl0f00924g	sakl0f00902g	sakl0f00880g	sakl0f00858g	sakl0f00836g	sakl0f00814g	sakl0f00792g	sakl0f00770g	sakl0f00748g	sakl0f00726g	sakl0f00704g	sakl0f00682g	sakl0f00660g	sakl0f00638g	sakl0f00616g	sakl0f00594g	sakl0f00572g	sakl0f00550g	sakl0f00528g	sakl0f00506g	sakl0f00484g	sakl0f00462g
sakl	sakl0g02926g	sakl0g02948g	sakl0g02970g	sakl0g02992g	sakl0g03014g	sakl0g03036g	sakl0g03058g	sakl0g03080g	sakl0g03102g	sakl0g03124g	sakl0g03146g	sakl0g03168g	sakl0g03190g	sakl0g03278g	sakl0g03300g	sakl0g03322g	sakl0g03344g	sakl0g03366g	sakl0g03388g	sakl0g03410g	sakl0g03432g	sakl0g03454g	sakl0g03476g	sakl0g03498g	sakl0g03520g	sakl0g03542g	sakl0g03564g	sakl0g03586g	sakl0g03608g	sakl0g03630g
klwa	Null	Null	Null	Null	Null	Null	Null	Null	Null	Null	Null	klwa_001-snap.1	klwa_001-snap.2	klwa_001-snap.3	klwa_001-snap.4	klwa_001-snap.5	klwa_001-snap.6	klwa_001-snap.7	klwa_001-snap.8	klwa_001-snap.9	klwa_001-snap.10	klwa_001-snap.11	klwa_001-snap.12	klwa_001-snap.13	klwa_001-snap.14	klwa_001-snap.15	klwa_001-snap.16	klwa_001-snap.17	klwa_001-snap.18	klwa_001-snap.19
klwa	Null	Null	Null	Null	Null	Null	Null	Null	Null	Null	Null	Null	Null	Null	klwa_265-snap.3	klwa_265-snap.2	klwa_265-snap.1	Null	Null	Null	Null	Null	Null	Null	Null	Null	Null	Null	Null	Null
klwa	Null	Null	Null	Null	Null	Null	Null	Null	Null	Null	Null	Null	klwa_331-snap.1	klwa_331-snap.2	klwa_331-snap.3	Null	Null	Null	Null	Null	Null	Null	Null	Null	Null	Null	Null	Null	Null	Null
klth	klth0a07524g	klth0a07546g	klth0a07568g	klth0a07590g	klth0a07612g	klth0a07634g	klth0a07656g	klth0a07678g	klth0a07700g	klth0a07722g	klth0a07744g	klth0a07766g	klth0a07810g	klth0a07832g	klth0a07964g	klth0a07986g	klth0a08008g	klth0a08052g	Null	Null	Null	Null	Null	Null	Null	Null	Null	Null	Null	Null
klth	klth0b10054g	klth0b10076g	klth0b10098g	klth0b10142g	klth0b10164g	klth0b10186g	klth0b10230g	klth0b10252g	klth0b10274g	klth0b10296g	klth0b10318g	klth0b10340g	klth0b10384g	klth0b10428g	klth0b10450g	klth0b10472g	klth0b10494g	Null	Null	Null	Null	Null	Null	Null	Null	Null	Null	Null	Null	Null
klth	Null	Null	Null	Null	Null	Null	Null	Null	Null	Null	Null	Null	Null	Null	klth0d00110g	klth0d00132g	klth0d00198g	klth0d00220g	klth0d00242g	klth0d00264g	klth0d00308g	klth0d00374g	klth0d00396g	klth0d00418g	klth0d00440g	klth0d00484g	klth0d00506g	klth0d00528g	klth0d00550g	klth0d00572g
klth	Null	Null	klth0d00110g	klth0d00132g	klth0d00198g	klth0d00220g	klth0d00242g	klth0d00264g	klth0d00308g	klth0d00374g	klth0d00396g	klth0d00418g	klth0d00440g	klth0d00484g	klth0d00506g	klth0d00528g	klth0d00550g	klth0d00572g	klth0d00594g	klth0d00616g	klth0d00638g	klth0d00660g	klth0d00682g	klth0d00704g	klth0d00726g	klth0d00748g	klth0d00770g	klth0d00792g	klth0d00814g	klth0d00836g
klth	klth0d17842g	klth0d17864g	klth0d17886g	klth0d17908g	klth0d17930g	klth0d17952g	klth0d17974g	klth0d17996g	klth0d18018g	klth0d18040g	klth0d18062g	klth0d18084g	klth0d18106g	klth0d18128g	klth0d18150g	klth0d18194g	Null	Null	Null	Null	Null	Null	Null	Null	Null	Null	Null	Null	Null	Null
klla	klla0a10813g	klla0a10791g	klla0a10769g	klla0a10703g	klla0a10681g	klla0a10659g	klla0a10637g	klla0a10593g	klla0a10571g	klla0a10549g	klla0a10527g	klla0a10505g	klla0a10483g	klla0a10461g	klla0a10439g	klla0a10417g	klla0a10395g	klla0a10373g	klla0a10351g	klla0a10329g	klla0a10307g	klla0a10285g	klla0a10263g	klla0a10241g	klla0a10219g	klla0a10197g	klla0a10175g	klla0a10153g	klla0a10131g	klla0a10109g
klla	Null	Null	Null	Null	Null	Null	Null	Null	klla0c00110g	klla0c00132g	klla0c00154g	klla0c00176g	klla0c00190g	klla0c00198g	klla0c00220g	klla0c00242g	klla0c00264g	klla0c00286g	klla0c00330g	klla0c00352g	klla0c00374g	klla0c00396g	klla0c00440g	klla0c00462g	klla0c00484g	klla0c00506g	klla0c00528g	klla0c00550g	klla0c00572g	klla0c00594g
klla	klla0c18931g	klla0c18953g	klla0c18975g	klla0c18997g	klla0c19019g	klla0c19041g	klla0c19063g	klla0c19085g	klla0c19107g	klla0c19132g	klla0c19184g	klla0c19206g	klla0c19228g	klla0c19250g	klla0c19272g	klla0c19316g	klla0c19327g	klla0c19338g	klla0c19360g	klla0c19382g	klla0c19437g	klla0c19547g	Null	Null	Null	Null	Null	Null	Null	Null
klla	klla0e14213g	klla0e14235g	klla0e14257g	klla0e14301g	klla0e14323g	klla0e14345g	klla0e14367g	klla0e14389g	klla0e14411g	klla0e14433g	klla0e14477g	klla0e14499g	klla0e14543g	klla0e14587g	klla0e14609g	klla0e14631g	klla0e14653g	klla0e14675g	klla0e14697g	klla0e14719g	klla0e14741g	klla0e14763g	klla0e14785g	klla0e14807g	klla0e14829g	klla0e14851g	klla0e14895g	klla0e14917g	klla0e14939g	klla0e14961g
cagu	Null	Null	Null	Null	Null	Null	Null	Null	Null	Null	Null	cagu_02295	cagu_02296	cagu_02297	cagu_02298	cagu_02299	cagu_02300	cagu_02301	cagu_02302	cagu_02303	cagu_02304	cagu_02305	cagu_02306	cagu_02307	cagu_02308	cagu_02309	cagu_02310	cagu_02311	cagu_02312	cagu_02313

Homologs of ARN1 and ARN2 genes. The names of the adjacent genes are shown and the homologous neighbours are highlighted in the same colour.

Cluster N (lineage 8): homologs of ORF 05665 (S. cerevisiae strain JAY291)

sace_c	0	0	0	0	0	0	0	0	0	0	0	33	5098	2737	4369	sace_c_05665	12627	11088	484	6028	4363	4351	1445	1443	1439	2496	23424	2357	468	945	178
sapa	0	0	0	0	33	82	5098	2737	4369	24589	2191	2191	2191	18051	468	sapa_c345_7143	11088	18050	75	484	6028	4363	4351	1445	1443	1439	2496	2412	2357	468	0
sami_a	0	0	0	0	0	0	0	0	0	0	0	0	0	0	0	sami_a_c1152_6761	11088	15320	17776	0	0	0	0	0	0	0	0	0	0	0	0
sami_a	0	0	0	0	0	0	0	0	0	0	0	0	0	0	468	sami_a_c1153_6763	0	0	0	0	0	0	0	0	0	0	0	0	0	0	0
saba_a	0	0	0	0	0	0	0	0	0	0	0	0	0	15446	21	saba_a_601.3	11088	124	12755	340	75	2987	1374	199	15447	0	0	0	0	0	0
saba_b	0	0	0	0	0	0	0	0	0	0	0	0	0	15446	21	saba_b_c68_15461	11088	124	12755	340	22663	75	2987	1374	199	15447	57	0	0	0	0
saku	0	0	0	0	0	0	0	0	0	0	0	0	4369	2191	468	saku_c1886.4	0	0	0	0	0	0	0	0	0	0	0	0	0	0	0
cagu	3890	3885	3886	3887	3888	3888	4190	6377	4192	6378	3275	3276	3278	3310	1748	cagu_00173	3309	3365	3364	3361	3362	3363	3360	1411	3388	6379	3389	3390	469	3411	471
cagu	942	939	938	937	6921	935	934	933	1592	1593	1594	846	846	767	631	cagu_04002	3953	662	1	468	3382	1398	262	856	3312	1429	3375	6923	6922	1883	65
cagu	2914	2914	82	6993	2194	6994	2899	677	677	4369	468	6995	6996	1504	1505	cagu_04513	876	4286	4285	4284	6997	50	1857	334	335	336	337	6373	338	475	2934
deha	525	2811	9420	2729	2737	6386	2736	2735	4218	3376	535	242	3383	4165	21584	deha2a03806g	3408	3469	2242	1796	407	9421	3235	3236	3237	3238	828	4293	3017	3018	157
deha	7139	468	1411	1411	1938	1938	912	9461	9462	33	280	605	21595	9463	340	deha2a14696g	7178	9464	346	21596	9465	9466	0	0	0	0	0	0	0	0	0
deha	4411	466	1566	3674	1377	9559	468	21625	9560	9561	473	9562	6450	9563	601	deha2b16478g	468	21626	21627	21628	21629	242	9564	9565	9393	9392	9393	9393	0	0	0
deha	3804	9782	57	2090	193	192	1363	4382	4381	3916	3278	6500	9783	21699	346	deha2e02596g	6366	1596	9784	9785	4378	527	236	9786	4508	2934	9787	190	9788	236	202
pipa	0	0	0	0	0	13871	22199	1746	13872	468	13873	1706	1746	13874	902	pipa_2g00050	13875	124	22200	1787	1354	22201	13876	13877	946	1114	4371	3384	1112	2735	1206
pipa	696	321	3604	14169	696	22235	1849	1995	1994	1435	1341	2502	14280	4591	4592	pipa_3g01745	1937	1936	14281	1142	1138	14282	1393	1703	1730	14283	2042	14284	99	1106	2391

Homologs of ORF 05665 (S. cerevisiae strain JAY291). The protein family classification of the adjacent genes is shown and the homologous neighbours are highlighted in the same colour.

sace_c	Null	Null	Null	Null	Null	Null	Null	Null	Null	Null	Null	sace_c_05497	sace_c_05500	sace_c_05483	sace_c_05486	sace_c_05665
sapa	Null	Null	Null	Null	sapa_c345_7189	sapa_c345_7185	sapa_c345_7178	sapa_c345_7176	sapa_c345_7166	sapa_c345_7162	sapa_c345_7161	sapa_c345_7159	sapa_c345_7158	sapa_c345_7157	sapa_c345_7151	sapa_c345_7143
sami_a	Null	Null	Null	Null	Null	Null	Null	Null	Null	Null	Null	Null	Null	Null	Null	sami_a_c1152_6761
sami_a	Null	Null	Null	Null	Null	Null	Null	Null	Null	Null	Null	Null	Null	Null	sami_a_c1153_6769	sami_a_c1153_6763
saba_a	Null	Null	Null	Null	Null	Null	Null	Null	Null	Null	Null	Null	Null	saba_a_601.1	saba_a_601.2	saba_a_601.3
saba_b	Null	Null	Null	Null	Null	Null	Null	Null	Null	Null	Null	Null	Null	saba_b_c68_15483	saba_b_c68_15469	saba_b_c68_15461
saku	Null	Null	Null	Null	Null	Null	Null	Null	Null	Null	Null	Null	saku_c1886.1	saku_c1886.2	saku_c1886.3	saku_c1886.4
cagu	cagu_00158	cagu_00159	cagu_00160	cagu_00161	cagu_00162	cagu_00163	cagu_00164	cagu_00165	cagu_00166	cagu_00168	cagu_00167	cagu_00169	cagu_00170	cagu_00171	cagu_00172	cagu_00173
cagu	cagu_03987	cagu_03988	cagu_03989	cagu_03990	cagu_03991	cagu_03992	cagu_03993	cagu_03994	cagu_03995	cagu_03996	cagu_03997	cagu_03998	cagu_03999	cagu_04000	cagu_04001	cagu_04002
cagu	cagu_04498	cagu_04499	cagu_04500	cagu_04501	cagu_04502	cagu_04503	cagu_04504	cagu_04505	cagu_04506	cagu_04507	cagu_04508	cagu_04509	cagu_04510	cagu_04511	cagu_04512	cagu_04513
deha	deha2a03454g	deha2a03476g	deha2a03498g	deha2a03520g	deha2a03542g	deha2a03564g	deha2a03586g	deha2a03608g	deha2a03630g	deha2a03652g	deha2a03674g	deha2a03696g	deha2a03718g	deha2a03740g	deha2a03784g	deha2a03806g
deha	deha2a14278g	deha2a14300g	deha2a14322g	deha2a14344g	deha2a14366g	deha2a14388g	deha2a14410g	deha2a14498g	deha2a14520g	deha2a14542g	deha2a14586g	deha2a14608g	deha2a14630g	deha2a14652g	deha2a14674g	deha2a14696g
deha	deha2b16148g	deha2b16170g	deha2b16192g	deha2b16214g	deha2b16236g	deha2b16258g	deha2b16280g	deha2b16302g	deha2b16324g	deha2b16346g	deha2b16368g	deha2b16390g	deha2b16412g	deha2b16434g	deha2b16456g	deha2b16478g
deha	deha2e02200g	deha2e02222g	deha2e02244g	deha2e02266g	deha2e02288g	deha2e02332g	deha2e02354g	deha2e02376g	deha2e02398g	deha2e02420g	deha2e02464g	deha2e02508g	deha2e02530g	deha2e02552g	deha2e02574g	deha2e02596g
pipa	Null	Null	Null	Null	Null	pipa_2g00000	pipa_2g00005	pipa_2g00010	pipa_2g00015	pipa_2g00020	pipa_2g00025	pipa_2g00030	pipa_2g00035	pipa_2g00040	pipa_2g00045	pipa_2g00050
pipa	pipa_3g01670	pipa_3g01675	pipa_3g01680	pipa_3g01685	pipa_3g01690	pipa_3g01695	pipa_3g01700	pipa_3g01705	pipa_3g01710	pipa_3g01715	pipa_3g01720	pipa_3g01725	pipa_3g01730	pipa_3g01735	pipa_3g01740	pipa_3g01745

sace_c	sace_c_05666	sace_c_05667	sace_c_05510	sace_c_05513	sace_c_05493	sace_c_05506	sace_c_05505	sace_c_05485	sace_c_05492	sace_c_05508	sace_c_05516	sace_c_05503	sace_c_05494	sace_c_05491	sace_c_05514
sapa	sapa_c345_7127	sapa_c345_7124	sapa_c345_7119	sapa_c345_7108	sapa_c345_7103	sapa_c345_7099	sapa_c345_7095	sapa_c345_7093	sapa_c345_7089	sapa_c345_7085	sapa_c345_7078	sapa_c345_7076	sapa_c345_7073	sapa_c345_7068	Null
sami_a	sami_a_c1152_6745	sami_b_c1152.1	sami_a_c1152_6739	Null	Null	Null	Null	Null	Null	Null	Null	Null	Null	Null	Null
sami_a	Null	Null	Null	Null	Null	Null	Null	Null	Null	Null	Null	Null	Null	Null	Null
saba_a	saba_a_601.4	saba_a_601.5	saba_a_601.6	saba_a_601.7	saba_a_601.8	saba_a_601.9	saba_a_601.10	saba_a_601.11	saba_a_601.13	Null	Null	Null	Null	Null	Null
saba_b	saba_b_c68_15452	saba_b_c68_15444	saba_b_c68_15440	saba_b_c68_15430	saba_b_c68_15426	saba_b_c68_15419	saba_b_c68_15411	saba_b_c68_15408	saba_b_c68_15399	saba_b_c68_15395	saba_b_c68_15393	Null	Null	Null	Null
saku	Null	Null	Null	Null	Null	Null	Null	Null	Null	Null	Null	Null	Null	Null	Null
cagu	cagu_00174	cagu_00175	cagu_00176	cagu_00177	cagu_00178	cagu_00179	cagu_00180	cagu_00181	cagu_00182	cagu_00183	cagu_00184	cagu_00185	cagu_00186	cagu_00187	cagu_00188
cagu	cagu_04003	cagu_04004	cagu_04005	cagu_04006	cagu_04007	cagu_04008	cagu_04009	cagu_04010	cagu_04011	cagu_04012	cagu_04013	cagu_04015	cagu_04014	cagu_04016	cagu_04017
cagu	cagu_04514	cagu_04515	cagu_04516	cagu_04517	cagu_04518	cagu_04519	cagu_04520	cagu_04521	cagu_04522	cagu_04523	cagu_04524	cagu_04525	cagu_04526	cagu_04527	cagu_04528
deha	deha2a04334g	deha2a04356g	deha2a04378g	deha2a04400g	deha2a04422g	deha2a04444g	deha2a04466g	deha2a04488g	deha2a04510g	deha2a04532g	deha2a04554g	deha2a04576g	deha2a04620g	deha2a04642g	deha2a04664g
deha	deha2a14718g	deha2a14740g	deha2a14762g	deha2a14784g	deha2a14806g	deha2a14828g	Null	Null	Null	Null	Null	Null	Null	Null	Null
deha	deha2b16500g	deha2b16522g	deha2b16544g	deha2b16566g	deha2b16588g	deha2b16610g	deha2b16632g	deha2b16654g	deha2b16676g	deha2b16698g	deha2b16720g	deha2b16742g	Null	Null	Null
deha	deha2e02618g	deha2e02640g	deha2e02662g	deha2e02728g	deha2e02750g	deha2e02772g	deha2e02794g	deha2e02816g	deha2e02838g	deha2e02860g	deha2e02882g	deha2e02904g	deha2e02926g	deha2e02948g	deha2e02970g
pipa	pipa_2g00055	pipa_2g00060	pipa_2g00065	pipa_2g00070	pipa_2g00075	pipa_2g00080	pipa_2g00085	pipa_2g00090	pipa_2g00095	pipa_2g00100	pipa_2g00105	pipa_2g00110	pipa_2g00120	pipa_2g00125	pipa_2g00130
pipa	pipa_3g01750	pipa_3g01755	pipa_3g01760	pipa_3g01765	pipa_3g01770	pipa_3g01775	pipa_3g01780	pipa_3g01785	pipa_3g01790	pipa_3g01795	pipa_3g01800	pipa_3g01805	pipa_3g01810	pipa_3g01815	pipa_3g01820

Homologs of ORF 05665 (S. cerevisiae strain JAY291). The names of the adjacent genes are shown and the homologous neighbours are highlighted in the same colour.

Cluster S (lineage 12): homologs of GEX1 and GEX2 genes

sace_a	0	0	0	0	0	0	0	0	0	0	0	0	0	0	0	sace0c00110g	236	662	4998	15495	764	4999	3894	3918	15744	15494	146	15493	2025	4003	5003
sace_a	0	0	0	0	0	0	0	0	0	0	0	0	0	0	0	sace0k07414g	236	57	57	75	15388	6057	2069	607	4486	2672	16693	2670	1549	1187	1381
sace_b	0	0	0	0	0	0	0	0	0	0	0	0	0	0	0	sace_b_1k5_3763g	57	57	75	15388	6057	2069	607	4486	16693	1549	1381	15395	1858	402	3262
sace_c	0	0	0	0	0	0	0	0	0	0	0	0	0	0	1040	sace_c_04015	662	0	0	0	0	0	0	0	0	0	0	0	0	0	0
sace_d	0	0	0	0	0	0	0	0	0	0	0	0	0	0	1040	sace_d_05343	236	662	4998	15495	764	4999	3894	3918	15744	15494	146	15493	2025	4003	5003
sace_e	0	0	0	0	0	0	0	0	0	0	0	0	0	0	0	sace_e_0517	236	662	4998	0	0	0	0	0	0	0	0	0	0	0	0
sapa	0	0	0	0	0	0	0	0	0	0	0	0	0	0	0	sapa_c403_13053	236	57	75	0	0	0	0	0	0	0	0	0	0	0	0
sapa	0	0	0	0	0	0	0	0	0	0	0	0	0	0	24697	sapa_c471_13051	0	0	0	0	0	0	0	0	0	0	0	0	0	0	0
sapa	0	0	0	0	0	0	0	0	0	0	0	0	0	0	24707	sapa_c498_2182	0	0	0	0	0	0	0	0	0	0	0	0	0	0	0
sapa	0	0	0	0	0	0	0	0	0	0	0	0	0	0	0	sapa_c84_2183	236	662	4998	16623	18044	764	4999	3894	3918	15494	146	15493	2025	4003	5003
sami_a	0	0	0	0	0	0	0	0	0	0	0	0	0	0	896	sami_a_c124_14126	236	57	15962	75	0	0	0	0	0	0	0	0	0	0	0
sami_a	0	0	0	0	0	0	0	0	0	0	0	0	0	0	0	sami_a_c124_14128	896	236	57	15962	75	0	0	0	0	0	0	0	0	0	0
sami_a	0	0	0	0	0	0	0	0	0	0	0	0	0	0	0	sami_a_c1599_23100	2725	0	0	0	0	0	0	0	0	0	0	0	0	0	0
sami_a	0	0	0	0	0	0	0	0	0	0	0	0	0	0	0	sami_a_c637_2273	662	4998	0	0	0	0	0	0	0	0	0	0	0	0	0
sami_b	0	0	0	0	0	0	0	0	0	0	0	0	0	0	0	sami_b_c2470.2	236	0	0	0	0	0	0	0	0	0	0	0	0	0	0
klwa	0	0	0	0	0	0	0	0	0	0	0	0	0	11717	12204	klwa_298-snap.3	236	12416	199	6068	1040	1040	1040	1040	1040	12755	6964	340	262	21	286
klth	0	0	0	0	0	0	0	0	0	0	0	0	0	0	0	klth0b00110g	236	82	134	1192	12169	4088	2191	468	2987	11328	57	2737	5098	912	468
klth	0	0	0	0	0	0	0	0	0	0	0	0	0	0	11717	klth0g00198g	11717	21	881	870	4996	5583	2377	2371	2376	4995	6948	262	3675	4210	1600
klla	0	0	0	0	0	0	0	0	234	465	10828	10829	2191	468	11208	klla0d19945g	236	11207	2412	1566	2280	4469	2719	2722	2720	2721	264	2522	5826	12	5825

Homologs of GEX1 and GEX2 genes. The protein family classification of the adjacent genes is shown and the homologous neighbours are highlighted in the same colour.

sace_a	Null	Null	Null	Null	Null	Null	Null	Null	Null	Null	Null	Null	Null	Null	Null	sace0c00110g	sace0c00132g	sace0c00154g	sace0c00176g	sace0c00198g	sace0c00220g	sace0c00242g	sace0c00264g	sace0c00286g	sace0c00308g	sace0c00330g	sace0c00352g	sace0c00374g	sace0c00396g	sace0c00418g	sace0c00440g
sace_a	Null	Null	Null	Null	Null	Null	Null	Null	Null	Null	Null	Null	Null	Null	Null	sace0k07414g	sace0k07392g	sace0k07370g	sace0k07348g	sace0k07326g	sace0k07304g	sace0k07282g	sace0k07260g	sace0k07238g	sace0k07216g	sace0k07194g	sace0k07172g	sace0k07150g	sace0k07128g	sace0k07106g	sace0k07084g
sace_b	Null	Null	Null	Null	Null	Null	Null	Null	Null	Null	Null	Null	Null	Null	Null	sace_b_1k5_3763g	sace_b_1k5_3741g	sace_b_1k5_3730g	sace_b_1k5_3719g	sace_b_1k5_3708g	sace_b_1k5_3697g	sace_b_1k5_3686g	sace_b_1k5_3675g	sace_b_1k5_3664g	sace_b_1k5_3642g	sace_b_1k5_3620g	sace_b_1k5_3598g	sace_b_1k5_3587g	sace_b_1k5_3576g	sace_b_1k5_3565g	sace_b_1k5_3554g
sace_c	Null	Null	Null	Null	Null	Null	Null	Null	Null	Null	Null	Null	Null	Null	sace_c_05661	sace_c_04015	sace_c_04017	Null	Null	Null	Null	Null	Null	Null	Null	Null	Null	Null	Null	Null	Null
sace_d	Null	Null	Null	Null	Null	Null	Null	Null	Null	Null	Null	Null	Null	Null	sace_d_05342	sace_d_05343	sace_d_05344	sace_d_05345	sace_d_05346	sace_d_05347	sace_d_05348	sace_d_05349	sace_d_05350	sace_d_05351	sace_d_05352	sace_d_05353	sace_d_05354	sace_d_05355	sace_d_05356	sace_d_05357	sace_d_05358
sace_e	Null	Null	Null	Null	Null	Null	Null	Null	Null	Null	Null	Null	Null	Null	Null	sace_e_0517	sace_e_0518	sace_e_0519	sace_e_0520	Null	Null	Null	Null	Null	Null	Null	Null	Null	Null	Null	Null
sapa	Null	Null	Null	Null	Null	Null	Null	Null	Null	Null	Null	Null	Null	Null	Null	sapa_c403_13053	sapa_c403_13057	sapa_c403_13063	sapa_c403_13072	Null	Null	Null	Null	Null	Null	Null	Null	Null	Null	Null	Null
sapa	Null	Null	Null	Null	Null	Null	Null	Null	Null	Null	Null	Null	Null	Null	sapa_c471_13047	sapa_c471_13051	Null	Null	Null	Null	Null	Null	Null	Null	Null	Null	Null	Null	Null	Null	Null
sapa	Null	Null	Null	Null	Null	Null	Null	Null	Null	Null	Null	Null	Null	Null	sapa_c498_2177	sapa_c498_2182	Null	Null	Null	Null	Null	Null	Null	Null	Null	Null	Null	Null	Null	Null	Null
sapa	Null	Null	Null	Null	Null	Null	Null	Null	Null	Null	Null	Null	Null	Null	Null	sapa_c84_2183	sapa_c84_2187	sapa_c84_2195	sapa_c84_2197	sapa_c84_2199	sapa_c84_2201	sapa_c84_2205	sapa_c84_2209	sapa_c84_2210	sapa_c84_2217	sapa_c84_2222	sapa_c84_2224	sapa_c84_2229	sapa_c84_2230	sapa_c84_2238	sapa_c84_2243
sami_a	Null	Null	Null	Null	Null	Null	Null	Null	Null	Null	Null	Null	Null	Null	sami_a_c124_14128	sami_a_c124_14126	sami_a_c124_14118	sami_a_c124_14112	sami_a_c124_14109	sami_a_c124_14108	Null	Null	Null	Null	Null	Null	Null	Null	Null	Null	Null
sami_a	Null	Null	Null	Null	Null	Null	Null	Null	Null	Null	Null	Null	Null	Null	Null	sami_a_c124_14128	sami_a_c124_14126	sami_a_c124_14118	sami_a_c124_14112	sami_a_c124_14109	sami_a_c124_14108	Null	Null	Null	Null	Null	Null	Null	Null	Null	Null
sami_a	Null	Null	Null	Null	Null	Null	Null	Null	Null	Null	Null	Null	Null	Null	Null	sami_a_c1599_23100	sami_b_c1599.3	Null	Null	Null	Null	Null	Null	Null	Null	Null	Null	Null	Null	Null	Null
sami_a	Null	Null	Null	Null	Null	Null	Null	Null	Null	Null	Null	Null	Null	Null	Null	sami_a_c637_2273	sami_a_c637_2270	sami_a_c637_2269	Null	Null	Null	Null	Null	Null	Null	Null	Null	Null	Null	Null	Null
sami_b	Null	Null	Null	Null	Null	Null	Null	Null	Null	Null	Null	Null	Null	Null	Null	sami_b_c2470.2	sami_b_c2470.1	Null	Null	Null	Null	Null	Null	Null	Null	Null	Null	Null	Null	Null	Null
klwa	Null	Null	Null	Null	Null	Null	Null	Null	Null	Null	Null	Null	Null	klwa_298-snap.1	klwa_298-snap.2	klwa_298-snap.3	klwa_298-snap.4	klwa_298-snap.5	klwa_298-snap.6	klwa_298-snap.7	klwa_298-snap.8	klwa_298-snap.9	klwa_298-snap.10	klwa_298-snap.11	klwa_298-snap.12	klwa_298-snap.13	klwa_298-snap.14	klwa_298-snap.15	klwa_298-snap.16	klwa_298-snap.17	klwa_298-snap.18
klth	Null	Null	Null	Null	Null	Null	Null	Null	Null	Null	Null	Null	Null	Null	Null	klth0b00110g	klth0b00132g	klth0b00154g	klth0b00176g	klth0b00220g	klth0b00264g	klth0b00286g	klth0b00308g	klth0b00330g	klth0b00352g	klth0b00374g	klth0b00396g	klth0b00418g	klth0b00440g	klth0b00462g	klth0b00484g
klth	Null	Null	Null	Null	Null	Null	Null	Null	Null	Null	Null	Null	Null	Null	klth0g00132g	klth0g00198g	klth0g00396g	klth0g00418g	klth0g00440g	klth0g00462g	klth0g00484g	klth0g00506g	klth0g00528g	klth0g00550g	klth0g00572g	klth0g00594g	klth0g00616g	klth0g00638g	klth0g00660g	klth0g00682g	klth0g00704g
klla	Null	Null	Null	Null	Null	Null	Null	Null	klla0d20031g	klla0d20009g	klla0d19987g	klla0d19965g	klla0d19957g	klla0d19953g	klla0d19949g	klla0d19945g	klla0d19941g	klla0d19933g	klla0d19929g	klla0d19921g	klla0d19899g	klla0d19877g	klla0d19855g	klla0d19833g	klla0d19811g	klla0d19789g	klla0d19767g	klla0d19745g	klla0d19723g	klla0d19701g	klla0d19646g

Homologs of GEX1 and GEX2 genes. The names of the adjacent genes are shown and the homologous neighbours are highlighted in the same colour.
