# Supplementary material for: The drug:H+ antiporters of family 2 (DHA2), siderophore transporters (ARN) and glutathione:H+ antiporters (GEX) have a common evolutionary origin in hemiascomycete yeasts
Source: BMC Genomics. 2013 Dec 18;14:901. doi: 10.1186/1471-2164-14-901 (PMC3890622; doi:10.1186/1471-2164-14-901)
Supplement: Additional file 10 — Analysis of the DNA upstream regions of S. cerevisiae VBA3 gene and ORF sace_e_3474. [file 1471-2164-14-901-S10.doc]

Analysis of the upstream region of VBA3 gene

DNA upstream region of VBA3 gene:

AATCAGAAAGTATTAATGTCAGGAGCAGATTTGTTCCGACGTATCCACAGTTATAGAAAA

CCGATCCTGCTGCATACATTGTGAGACGGGTCGCCTGTGATTGAATGATGGTTCCCATTA

TATAAAAAATAGTTGCAACTAAAAAAAGCCTTAGTCTTCCGAAGTGGTCAGAGAGTCTGG

AGTAGACAACTTGGGATCCGACACTTACAACAGCATTGATAACTTGGACAGTTGAAAGTA

AGGAGTGTTCTGAATATGAGTTCGTCGCATAGCCCGTATAGGTCGATCTAAGTGTGTAGT

CTAAACTAATTCCAAACCCACATACAAACGCGGTACTTATCAGTAGAATTTTATATTTCA

AGGAATCAAACTGTGCAGACATAATTTCGTTTTCTTTGAGCTTAAAAAAGGTCGATGTCA

TAGAGTACGTGTCATCGTTTGAGTGACGTTCTCGCTCAATAATCTCACAGGACTGCCTAA

TTCCACTTTTTTTATTACTTGAGGCACCAACAACACTAGAACTCATCCGATAATTTATTT

ACTTGATTATTCCTTTTTTTTTTTTTTCTCTTTCCAGTCCATAAAACTATTTTACTTTTC

TTATTTATGTAAATAGTATTTAAGATTCTACGGTCGCTACATCTGCTAGAAATGGAATTA

CTTTAATACATTCCATGTTCTCATATATACTTTTACTGACACCTTTCGTCACTATTTTTT

GTTTAACTTTTTTTTTGGAAGTACTAAAATTATATGCTCGTAATTATTGAGTATCAAAAA

AAAAAAAAAAAAAAATTGCTGTGACACCCCTTCAATGTGGTGTCTATACACCTGGTGACT

TTAACTTTAACTTTGACCTGTCGTCGTACAATCAACTTAGTTTCCGAAAATCCACGACGA

ACCAGTTATACCGAAGACTTTTGTACGACAAGAATACTGTACCGTCGAAACTATCTCTTT

TCAAACTGATGAGTGAAGCTTGTGTTTATGTCTGTTGAAACAAGGAAAAAAATGCGGTGC

CTGTCGACAGGATATGCGCACAAGTGCAAAATCATCAAAAAATTGAGTACCTAGATCCGG

TGGTGTAGGACGAGCAAAGTTCCTTCATAAACAAACCGTAAGGGTTACTGATACACAATT

TCCTTTTTGTAAAGAGTATTTGAGCAAATTAGTCAGCGATTGAAAACAACACCCTTTTTT

CTATATATAGTAGGTGAAAGTTAACATGCGAGTAAAAGTAGTGCCACATTTCCTTTGCAG

CAGGTGCAAATTGGTACATATTTAACAGATAGCGCCGCACCTCATATGCAGCGCGTACAT

GATACGAGACGACAAGATATGCAAAAGATAATAGTGTCATCACACCTTTATGAGAAGCGA

ATTTTTTTTTTTTTTTGGTTTAAATATATATATATACATATATATAGATATAGATATACA

CATGTATAGATGTATTCTAGTTATGCTTATACCTAGAACTGATCAGACCAGATCATACCT

GGTATAGAGCTTAATCGATAGAAATTCAAAAATATGGAGGAAACTAAGTACTCTTCGCAG

CAGGAGATAGAAGAAGCATGTGGTTCAGACGCTTCATTGAATGCTAGAGGTAGCAATGAT

TCTCCAATGGGACTTTCCTTGTACCTCTGCCTGGCTTCGTGAACTCTTGTACTATTCATA

ACTGCACTGGATATTTTGATAGTGGGAACTATTATTGACGTGGTCGCAGAACAGTTCGGA

AACTACTCCAAAACAGGTTGGCTCGTTACAGGCTACAGTTTACCAAATGCTATTCTGAGT

CTCATTTGGGGAAGATTCGCATCTATCATAGGTTTCCAGCATAGTCTCATTTTAGCAATA

CTTATTTTTGAAGCCGGATCCCTAATTGCTGCCCTTGCCTCTTCA

Coding sequence of VBA3 gene:

ATGAATATGCTCATT

GTCGGTAGAGTTGTTGCTAGTGTTGGGGGAAGCGGACTTCAAACGCTTTGCTTTGTTATT

GGTTGTACGATGGTTGGTGAAAGGTCACGTCCATTGGTGATTTCCATCCTAAGTTGTGCA

TTTGCTGTAGCTGCTATCGTTGGTCCTATAATCGGAGGTGCCTTTACAACCCATGTTACC

TGGAGGTGGTGCTTCTATATCAATCTTCCTATCGGTGGTCTTGCCATTATTATGTTTTTA

CTCACATATAAGGCCGAGAATAAGGGTATACTTCAACAAATTAAAGATGCTATAGGAACA

ATCTCGAGCTTTACTTTTAGTAAGTTCAGACACCAAGTTAATTTTAAAAGACTTATGAAT

GGCATAATCTTCAAGTTTGACTTCTTTGGTTTTGCCCTCTGCTCTGCAGGGCTGGTCCTT

TTCCTACTGGGGCTAACCTTTGGTGGTAATAAATATAGTTGGAACTCTGGCCAAGTCATC

GCATATTTGGTTTTGGGTGTCTTACTTTTTATTTTTTCATTGGTGTACGATTTCTTCTTA

TTCGATAAATTCAACCCGGAACCTGATAATATATCCTACAGGCCTCTCCTTCTAAGAAGA

TTGGTAGCAAAACCAGCCATAATAATAATAAACATGGTAACATTTCTATTATGTACCGGT

TACAATGGGCAAATGATATACTCTGTCCAGTTTTTCCAACTTATATTTGCGTCGAGTGCA

TGGAAAGCCGGTCTTCACTTGATACCAATCGTTATTACCAACGTTATTGCGGCCATTGCA

AGTGGTGTGATTACCAAAAAGCTCGGTTTAGTTAAACCACTCTTAATATTTGGAGGCGTT

CTTGGGGTAATTGGAGCAGGGCTTATGACACTTATGACAAATACGTCCACGAAGTCAACT

CAAATTGGTGTTTTGCTATTACCGGGGTTTTCCCTTGGATTTGCTCTACAAGCATCGCTC

ATGAGTGCACAGCTTCAAATTACCAAAGATCGTCCAGAAGCTGCTATGGACTTTATTGAA

GTAACAGCTTTCAATACATTCATGAAGTCATTAGGTACAACTCTTGGTGGTGTGCTTTCA

ACCACTGTTTTTTCCGCCTCCTTTCACAACAAAGTATCACGAGCTCATCTAGAGCCTTAC

GAAGGAAAAACGGTTGATGACATGATTTTGTATCGTCTTCAAAACTACGACGGTTCTCAT

TCGACTATTGGAAACATTTTAAGCGACTCCATTAAGAACGTATTTTGGATGGATCTAGGG

TTTTATGCCTTAGGATTTTTGTTTTGTAGTTTTTCATCCAATAAGAAATTAATCATACCA

AAAAAGGACGAGACACCAGAAGATAATTTAGAAGACAAGTAG

1) Translation of upstream sequence of VBA3 using transeq function of EMBOSS (in the three different frames):

Output file outseq

>_1

NQKVLMSGADLFRRIHSYRKPILLHTL*DGSPVIE*WFPLYKK*LQLKKALVFRSGQRVW

SRQLGIRHLQQH**LGQLKVRSVLNMSSSHSPYRSI*VCSLN*FQTHIQTRYLSVEFYIS

RNQTVQT*FRFL*A*KRSMS*STCHRLSDVLAQ*SHRTA*FHFFYYLRHQQH*NSSDNLF

T*LFLFFFFLFPVHKTILLFLFM*IVFKILRSLHLLEMELL*YIPCSHIYFY*HLSSLFF

V*LFFWKY*NYMLVIIEYQKKKKKKLL*HPFNVVSIHLVTLTLTLTCRRTINLVSENPRR

TSYTEDFCTTRILYRRNYLFSN**VKLVFMSVETRKKMRCLSTGYAHKCKIIKKLST*IR

WCRTSKVPS*TNRKGY*YTISFL*RVFEQISQRLKTTPFFLYIVGES*HASKSSATFPLQ

QVQIGTYLTDSAAPHMQRVHDTRRQDMQKIIVSSHLYEKRIFFFFWFKYIYIHIYRYRYT

HV*MYSSYAYT*N*SDQIIPGIELNR*KFKNMEETKYSSQQEIEEACGSDASLNARGSND

SPMGLSLYLCLAS*TLVLFITALDILIVGTIIDVVAEQFGNYSKTGWLVTGYSLPNAILS

LIWGRFASIIGFQHSLILAILIFEAGSLIAALASS

>_2

IRKY*CQEQICSDVSTVIENRSCCIHCETGRL*LNDGSHYIKNSCN*KKP*SSEVVRESG

VDNLGSDTYNSIDNLDS*K*GVF*I*VRRIARIGRSKCVV*TNSKPTYKRGTYQ*NFIFQ

GIKLCRHNFVFFELKKGRCHRVRVIV*VTFSLNNLTGLPNSTFFIT*GTNNTRTHPIIYL

LDYSFFFFFSFQSIKLFYFSYLCK*YLRFYGRYIC*KWNYFNTFHVLIYTFTDTFRHYFL

FNFFFGSTKIICS*LLSIKKKKKKNCCDTPSMWCLYTW*L*L*L*PVVVQST*FPKIHDE

PVIPKTFVRQEYCTVETISFQTDE*SLCLCLLKQGKKCGACRQDMRTSAKSSKN*VPRSG

GVGRAKFLHKQTVRVTDTQFPFCKEYLSKLVSD*KQHPFFYI**VKVNMRVKVVPHFLCS

RCKLVHI*QIAPHLICSAYMIRDDKICKR**CHHTFMRSEFFFFFGLNIYIYIYIDIDIH

MYRCILVMLIPRTDQTRSYLV*SLIDRNSKIWRKLSTLRSRR*KKHVVQTLH*MLEVAMI

LQWDFPCTSAWLRELLYYS*LHWIF**WELLLTWSQNSSETTPKQVGSLQATVYQMLF*V

SFGEDSHLS*VSSIVSF*QYLFLKPDP*LLPLPLX

>_3

SESINVRSRFVPTYPQL*KTDPAAYIVRRVACD*MMVPII*KIVATKKSLSLPKWSESLE

*TTWDPTLTTALITWTVESKECSEYEFVA*PV*VDLSV*SKLIPNPHTNAVLISRILYFK

ESNCADIISFSLSLKKVDVIEYVSSFE*RSRSIISQDCLIPLFLLLEAPTTLELIR*FIY

LIIPFFFFSLSSP*NYFTFLIYVNSI*DSTVATSARNGITLIHSMFSYILLLTPFVTIFC

LTFFLEVLKLYARNY*VSKKKKKKIAVTPLQCGVYTPGDFNFNFDLSSYNQLSFRKSTTN

QLYRRLLYDKNTVPSKLSLFKLMSEACVYVC*NKEKNAVPVDRICAQVQNHQKIEYLDPV

V*DEQSSFINKP*GLLIHNFLFVKSI*AN*SAIENNTLFSIYSR*KLTCE*K*CHISFAA

GANWYIFNR*RRTSYAART*YETTRYAKDNSVITPL*EANFFFFLV*IYIYTYI*I*IYT

CIDVF*LCLYLELIRPDHTWYRA*SIEIQKYGGN*VLFAAGDRRSMWFRRFIEC*R*Q*F

SNGTFLVPLPGFVNSCTIHNCTGYFDSGNYY*RGRRTVRKLLQNRLARYRLQFTKCYSES

HLGKIRIYHRFPA*SHFSNTYF*SRIPNCCPCLFX

2) The immediate sequence of reading frame 1 (until first stop codon) was selected as query sequence against the S. cerevisiae reference genome:

TLVLFITALDILIVGTIIDVVAEQFGNYSKTGWLVTGYSLPNAILSLIWGRFASIIGFQHSLILAILIFEAGSLIAALASS

3) Using the WU-BLASTP available in SGD as pairwise comparison tool, the followed output is obtained:

YKR105C VBA5 SGDID:S000001813 Chr XI from 660464-658716, reverse complement,

Uncharacterized ORF, "Putative transporter of the Major Facilitator Superfamily (MFS); proposed role as a basic amino acid permease based on phylogeny"

[ Retrieve Sequence / ORF Map / Genome Browser / SGD Locus page ]

Length = 583

Score = 143.8 bits (394), Expect = 3.4e-38, P = 3.4e-38

Identities = 81/81 (100%), Positives = 81/81 (100%)

Query: 1 TLVLFITALDILIVGTIIDVVAEQFGNYSKTGWLVTGYSLPNAILSLIWGRFASIIGFQH 60

TLVLFITALDILIVGTIIDVVAEQFGNYSKTGWLVTGYSLPNAILSLIWGRFASIIGFQH

Sbjct: 44 TLVLFITALDILIVGTIIDVVAEQFGNYSKTGWLVTGYSLPNAILSLIWGRFASIIGFQH 103

Query: 61 SLILAILIFEAGSLIAALASS 81

SLILAILIFEAGSLIAALASS

Sbjct: 104 SLILAILIFEAGSLIAALASS 124

4) Comparison between the translated DNA upstream sequence of the VBA3 gene and the N-terminal peptide of VBA5 gene and ORF sace_e_0518 (a VBA3 ortholog):

A - Reading frame 1 of DNA upstream sequence of VBA3 gene (sequence with a stop codon):

MEETKYSSQQEIEEACGSDASLNARGSNDSPMGLSLYLCLAS*TLVLFITALDILIVGTIIDVVAEQFGNYSKTGWLVTGYSLPNAILSLIWGRFASIIGFQHSLILAILIFEAGSLIAALASS

B – N-terminal amino acid sequence of ORF sace_e_0518:

MEETKYSSQQEIEEACGSDASLNARGSNDSPMGLSLYLCLASLTLVLFITALDILIVGTIIDVVAEQFGNYSKTGWLVTGYSLPNAILSLIWGRFASIIGFQHSLILAILIFEAGSLIAALASS

C - N-terminal amino acid sequence of Vba5p:

MEETKYSSQQEIEGACGSDASLNARGSNDSPMGLSLYLCLASLTLVLFITALDILIVGTIIDVVAEQFGNYSKTGWLVTGYSLPNAILSLIWGRFASIIGFQHSLILAILIFEAGSLIAALASS

Output (residue differences indicated by asterisk):

* *

A - MEETKYSSQQEIEEACGSDASLNARGSNDSPMGLSLYLCLAS*TLVLFITALDILIVGTIIDVVAEQFGNYSKTGWLVTGYSLPNAILSLIWGRFASIIGFQHSLILAILIFEAGSLIAALASS

B - MEETKYSSQQEIEEACGSDASLNARGSNDSPMGLSLYLCLASLTLVLFITALDILIVGTIIDVVAEQFGNYSKTGWLVTGYSLPNAILSLIWGRFASIIGFQHSLILAILIFEAGSLIAALASS

C - MEETKYSSQQEIEGACGSDASLNARGSNDSPMGLSLYLCLASLTLVLFITALDILIVGTIIDVVAEQFGNYSKTGWLVTGYSLPNAILSLIWGRFASIIGFQHSLILAILIFEAGSLIAALASS

Analysis of the upstream region of ORF sace_e_3474

DNA upstream region of ORF sace_e_3474 (upstream sequence ends in contig extremity):

TCATGGACAGAGTTCTCACCATACAGGTTGGCTCGTTACAGGCTACAGTTTACCAAATGC

TATTCTGAGTCTCATTTGGGGAAGATTCGCATCTATCATAGGTTTCCAGCATAGTCTCAT

TTTAGCAATACTTATTTTTGAAGCTGGATCCTTAATTGCTGCCCTTGCCTCTTCA

Coding sequence of ORF sace_e_3474:

ATGAA

TATGCTCATTGTCGGTAGAGTTGTTGCTGGTGTTGGGGGAAGCGGACTTCAAACGCTTTG

CTTTGTTATTGGTTGTACGATGGTTGGTGAAAGGTCGCGTCCATTGGTGATTTCCATCCT

AAGTTGTGCATTTGCTGTAGCTGCTATCGTCGGTCCTATAATCGGAGGTGCCTTTACAAC

CCATGTTACCTGGAGGTGGTGCTTCTATATCAATCTTCCTATCGGTGGTCTTGCCATTAT

TATGTTTTTACTCACCTATAAGGCCGAGAATAAGGGTATACTTCAACAAATTAAAGATGC

TATAGGAACAATCTCGAGCTTTACTTTTAGTAAGTTCAGACACCAAGTTAATTTTAAAAG

ACTTATGAATGGCATAATCTTCAAGTTTGACTTCTTTGGTTTTGCCCTCTGCTCTGCAGG

GCTGGTCCTTTTCCTACTGGGGCTAACCTTTGGCGGTAATAAATATAGTTGGAACTCTGG

CCAAGTCATCGCATATTTGGTTTTGGGTGTCTTACTTTTTATTTTTTCATTGGTGTACGA

TTTCTTCCTATTCGATAAATTCAACCCGGAACCTGATAATATATCCTACAGGCCTCTCCT

TCTAAGAAGATTGGTAGCAAAACCAGCCATAATAATAGTAAACATGGTAACATTTCTATT

ATGTACCGGTTACAATGGGCAAATGATATACTCTGTCCAGTTTTTCCAACTTATATTTGC

GTCGAGTGCATGGAAAGCCGGTCTTCACTTGATACCAATCGTTATTACCAACGTTATTGC

GGCCATTGCAAGTGGTGTGATTACCAAAAAGCTCGGTTTAGTTAAACCACTCTTAATATT

TGGAGGCGTTCTTGGGGTAATTGGAGCAGGGCTTATGACACTTATGACAAATACGTCCAC

GAAGTCAACTCAAATTGGTGTTTTGTTATTACCGGGGTTTTCCCTTGGATTTGCTCTACA

AGCATCGCTCATGAGTGCACAGCTTCAAATTACCAAAGATCGTCCAGAAGCTGCTATGGA

CTTTATTGAAGTAACAGCTTTCAATACATTCATGAAGTCATTAGGTACAACTCTTGGTGG

TGTGCTTTCAACCACTGTTTTTTCCGCCTCCTTTCACAACAAAGTATCACGAGCTCATCT

AGAGCCTTACGAAGGAAAAACGGTTGATGACATGATTTTGTATCGTCTTCAAAACTACGA

CGGTTCTCATTCGACTATTGGAAACATTTTAAGCGACTCCATTAAGAACGTATTTTGGAT

GGACCTAGGGTTTTATGCCTTAGGATTTTTGTTTTGTAGTTTTTCATCCAATAAGAAATT

AATCATACCAAAAAAGGACGAGACACCAGAAGATAATTTAGAAGACAAG

1) Translation of upstream sequence of ORF sace_e_3474 using transeq function of EMBOSS:

HGQSSHHTGWLVTGYSLPNAILSLIWGRFASIIGFQHSLILAILIFEAGSLIAALASS

2) This amino acid sequence was selected as query sequence against the S. cerevisiae reference genome. Using the WU-BLASTP available in SGD as pairwise comparison tool, the followed output is obtained:

YKR105C VBA5 SGDID:S000001813 Chr XI from 660464-658716, reverse complement,

Uncharacterized ORF, "Putative transporter of the Major Facilitator Superfamily (MFS); proposed role as a basic amino acid permease based on phylogeny"

[ Retrieve Sequence / ORF Map / Genome Browser / SGD Locus page ]

Length = 583

Score = 93.4 bits (251), Expect = 1.4e-21, P = 1.4e-21

Identities = 51/51 (100%), Positives = 51/51 (100%), Frame = +2

Query: 23 TGWLVTGYSLPNAILSLIWGRFASIIGFQHSLILAILIFEAGSLIAALASS 175

TGWLVTGYSLPNAILSLIWGRFASIIGFQHSLILAILIFEAGSLIAALASS

Sbjct: 74 TGWLVTGYSLPNAILSLIWGRFASIIGFQHSLILAILIFEAGSLIAALASS 124

3) Comparison between the translated DNA upstream sequence of the VBA3 gene and of ORF sace_e_3474 and the N-terminal peptide of VBA5 gene and of ORF sace_e_0518 (a VBA3 ortholog):

A - Reading frame 1 of DNA upstream sequence of VBA3 gene (sequence with a stop codon):

MEETKYSSQQEIEEACGSDASLNARGSNDSPMGLSLYLCLAS*TLVLFITALDILIVGTIIDVVAEQFGNYSKTGWLVTGYSLPNAILSLIWGRFASIIGFQHSLILAILIFEAGSLIAALASS

B – N-terminal amino acid sequence of ORF sace_e_0518:

MEETKYSSQQEIEEACGSDASLNARGSNDSPMGLSLYLCLASLTLVLFITALDILIVGTIIDVVAEQFGNYSKTGWLVTGYSLPNAILSLIWGRFASIIGFQHSLILAILIFEAGSLIAALASS

C - N-terminal amino acid sequence of Vba5p:

MEETKYSSQQEIEGACGSDASLNARGSNDSPMGLSLYLCLASLTLVLFITALDILIVGTIIDVVAEQFGNYSKTGWLVTGYSLPNAILSLIWGRFASIIGFQHSLILAILIFEAGSLIAALASS

D – Translated DNA upstream sequence of ORF sace_e_3474:

HGQSSHHTGWLVTGYSLPNAILSLIWGRFASIIGFQHSLILAILIFEAGSLIAALASS

Output (residue differences indicated by asterisk):

* * *******

1 - MEETKYSSQQEIEEACGSDASLNARGSNDSPMGLSLYLCLAS*TLVLFITALDILIVGTIIDVVAEQFGNYSKTGWLVTGYSLPNAILSLIWGRFASIIGFQHSLILAILIFEAGSLIAALASS

2 - MEETKYSSQQEIEEACGSDASLNARGSNDSPMGLSLYLCLASLTLVLFITALDILIVGTIIDVVAEQFGNYSKTGWLVTGYSLPNAILSLIWGRFASIIGFQHSLILAILIFEAGSLIAALASS

3 - MEETKYSSQQEIEGACGSDASLNARGSNDSPMGLSLYLCLASLTLVLFITALDILIVGTIIDVVAEQFGNYSKTGWLVTGYSLPNAILSLIWGRFASIIGFQHSLILAILIFEAGSLIAALASS

4 - HGQSSHHTGWLVTGYSLPNAILSLIWGRFASIIGFQHSLILAILIFEAGSLIAALASS
